# Taxonomy of the ant genus *Carebara* Westwood (Formicidae, Myrmicinae) in the Malagasy Region

**DOI:** 10.3897/zookeys.767.21105

**Published:** 2018-06-18

**Authors:** Frank Azorsa, Brian L. Fisher

**Affiliations:** 1 Entomology Department, California Academy of Sciences, San Francisco, California, U.S.A.; 2 División de Entomologia, Centro de Ecologia y Biodiversidad, CEBIO, Lima, PERU.

**Keywords:** *Carebara*, Crematogastrini, Intercastes, Intermediates, Madagascar, Malagasy region, *Pheidologeton*, Polymorphic, Taxonomy

## Abstract

The genus *Carebara* is revised for the Malagasy region, and based on the examination of over 10,000 specimens, twenty-three species are recognized. Twenty one of these are described as new (*C. bara* **sp. n.**, *C. berivelo* **sp. n.**, *C. betsi* **sp. n.**, *C. creolei* **sp. n.**, *C. demeter* **sp. n.**, *C. dota* **sp. n.**, *C. hainteny* **sp. n.**, *C. hiragasy* **sp. n.**, *C. jajoby* **sp. n.**, *C. kabosy* **sp. n.**, *C. lova* **sp. n.**, *C. mahafaly* **sp. n.**, *C. malagasy* **sp. n.**, *C. omasi* **sp. n.**, *C. placida* **sp. n.**, *C. raberi* **sp. n.**, *C. salegi* **sp. n.**, *C. sampi* **sp. n.**, *C. tana* **sp. n.**, *C. tanana* **sp. n.**, *C. vazimba* **sp. n.**), and two are redescribed, *C. grandidieri* Forel (= *C. voeltzkowi* Forel **n. syn.**) and *C. nosindambo* Forel. A lectotype is designated for *C. nosindambo. C. creolei* **sp. n.** is known only from Mauritius and Seychelles, *C. grandidieri* Forel is distributed in Comoros, Madagascar and Mayotte, and the other twenty-one species are endemic to Madagascar. Most of the *Carebara* species recorded in this work are endemic to a specific habitat (ecoregion), but some of them (*C. bara* **sp. n.**, *C. grandidieri* Forel, *C. jajoby* **sp. n.**, *C. kabosy* **sp. n.**, and *C. nosindambo* Forel) are widespread within Madagascar across all major habitats. The worker caste of *Carebara* can be differentiated from other genera in the Myrmicinae subfamily by the presence of the following combination of characters: antennae of eight to eleven segments, with a two-segmented club; anterior clypeal margin without central isolated seta (rarely present in some species or specimens), and usually with four distinct setae; mandibles with four to seven teeth (except in one species from Ghana - *C. crigensis* with three teeth); and palp formula 2,2 or 1,2. We report that almost all *Carebara* species found in the Malagasy region have intermediates (distinct forms) in the major worker subcaste, with the largest major workers showing remnants of queen flight sclerites and ocelli. The widespread presence of intermediates in the major worker subcaste expands the morphological boundaries of *Carebara*. We present an overview of the natural history of *Carebara* in the Malagasy region, an illustrated key for the identification of the known Malagasy species of *Carebara*, as well as high-resolution images and distribution maps. Unique identifiers are used for all specimens studied, including type material, and the raw data that forms the basis of this study are available on www.antweb.org (open access).

## Introduction

The Malagasy region includes the island of Madagascar, as well as the smaller islands of the western Indian Ocean (Comoros, Mauritius, Mayotte, Reunion, Seychelles, and others). The greatest concentration of biodiversity and endemism in the region is found in Madagascar (Goodman and Benstead 2003). Ants in particular have been well studied and systematically collected in the Malagasy region since 1992. These collections were made across the Malagasy region using a variety of different standardized methods (see Fisher 2005), resulting in the identification of many new species and genera. As of September 2017, there are approximately 656 species of ants described from the Malagasy region, nearly half of which have been described since 2000 (Bolton 2017). The total ant fauna in the Malagasy region is estimated to be greater than 1200 species.

The genus *Carebara* is distributed worldwide, present mainly in tropical and subtropical regions, and contains approximately 253 described taxa (222 valid species, including the newly described species in this work, 23 valid subspecies, and 8 fossil species) (Bolton 2017; Fischer et al. 2014, 2015; Bharti and Kumar 2013). The taxonomy and limits of *Carebara* have not been well defined, although several genera were recently synonymized with *Carebara* based on morphological and molecular information, for example: Fernández (2004, 2006 and 2010) placed *Afroxyidris*, *Oligomyrmex*, *Paedalgus* and *Parvimyrma* as junior synonyms of *Carebara*, and indicated similarities between *C. villiersi* and *Pheidologeton*, expanding the boundaries of the genus. Fischer et al. (2014) placed *Pheidologeton* as a junior synonym of *Carebara*, based on the morphological similarities between former *Pheidologeton* and the *C. polita* group, (*C. brevipilosa*, *C. madibai*, *C. perpusilla*, *C. polita*, *C. nicotianae*, *C. silvestrii*, *C. urichi*, and *C. villiersi*), and the prescence of intermediates (distinct forms) in the major worker subcaste of the *C. polita* group. In addition to the morphological similarities, molecular studies have revealed a close relationship between *Carebara*, *Oligomyrmex* and *Pheidologeton* (Moreau et al. 2006; Moreau and Bell 2013), and a recent molecular study provided additional support for the synonymization of *Pheidologeton* under *Carebara*, revealing that the former is nested within the latter (Ward et al. 2015). The *Carebara* taxa included in the Ward et al. (2015) study included: *C. affinis* (formerly *Pheidologeton*), *C. urichi* (*C. polita* group), *C. alperti* (morphologically similar to former *Pheidologeton*), *C. nosindambo* (formerly *Oligomyrmex*) and *C. vidua* (*Carebara* s. str.); no samples of former *Paedalgus* were included in this work. Future studies including morphological and molecular data and sampling including former *Paedalgus* taxa will help to understand the evolutionary biology of this group, as well as the monophyly of *Carebara*.

After all of the recent synonymizations were made, *Carebara* species (not including former *Pheidologeton*) were provisionally placed in five species groups: *C. concinna* (*Oligomyrmex* and *Carebara* s. str.), *C. alperti* (*alperti* group), *C. crigensis* (*Afroxyidris*), *C. escherichi* (*Paedalgus*), and *C. polita* groups (Fernández 2004, Bharti and Kumar 2013, Fischer et al. 2014), former *Pheidologeton* species were not included in any species groups yet, but in future revisions could be splitted in two species groups (Fischer et al. 2014). Fischer et al. (2014, 2015) highlighted the need to revise all *Carebara* species to better delimit the boundaries of the species groups proposed by Fernández (2004) and Bharti and Kumar (2013). A preliminary morphological study suggests that all *Carebara* species can be placed in ten species groups (Azorsa et al. in preparation); future molecular, morphological and biogeographic studies will help to clarify and better understand the taxonomy of *Carebara* and their limits. Before this work only three species of *Carebara* were reported from Madagascar: *C. grandidieri* (Forel, 1891) (q, m), *C. nosindambo* (Forel, 1891) (w, m, q), and *C. voeltzkowi* (Forel, 1907) (q). We describe 21 new species and propose *C. voeltzkowi* as a junior synonym of *C. grandidieri*, increasing the diversity of *Carebara* to 23 species for the Malagasy region.

## Methods

### Samples

This project is based on the study of more than 10,000 specimens collected from 1992 to 2016. Collecting methods included more than 6,000 leaf litter samples, 4,000 pitfall traps, and 9,000 additional hand collecting events (for more information see Fisher 2005). All the material studied in this work is deposited in the Entomology Collection of the California Academy of Sciences, San Francisco CA, U.S.A. We also examined type material of the three described species of *Carebara* (*C. grandidieri*, *C. nosindambo* and *C. voeltzkowi* syn. n. of *C. grandidieri*), deposited in the Muséum d’Histoire Naturelle de la Ville de Genève, Geneva, Switzerland, and the Museum of Natural History, Humboldt University, Berlin. Additional specimens were revised from the P. S. Ward Collection, University of California, Davis CA, U.S.A., and the Museum of Comparative Zoology, Harvard University, Cambridge, Massachusetts, U.S.A.

### Terminology

For morphological characters we followed Bolton (1994), Bolton and Belshaw (1993), Belshaw and Bolton (1994), Bolton (2003), Ettershank (1966), Fernández (2004, 2006 and 2010), and Fischer et al. (2014, 2015). For sculpture characters we followed Harris (1979).

### Images

We present high-resolution images from all species and type material examined, holotype images from all the new species, lectotype images of *C. nosindambo* (queen), as well as images of the queen of *C. voeltzkowi* (syn. n. of *C. grandidieri*). We also present images of the *intermediates* of each species. All images were created using a JVC KY-F75 digital camera and Syncroscopy Auto-Montage (version 5.0) software. Images of all species were edited using Adobe Illustrator and Photoshop, and uploaded to AntWeb, where anyone can access and download the images of all the species presented in this work (www.antweb.org). The illustrations showing the measurements and morphological characters used in this work were made using Adobe Illustrator. All imaged specimens (type and non-type material) are uniquely identified with specimen-level codes (e.g. CASENT0483010).

### Distribution maps

We mapped the distribution of all species using ArcGIS v10.2.1 (ESRI, Redlands, CA) and R (R Core Team 2017).

### Measurements and indices

We measured more than 500 specimens (at least 10 specimens per caste/species, and more in the species with intermediates in the major worker subcaste). Values are presented in mm, with measurements of holotype specimens in parentheses. All measurements were taken with a Leica MZ 12.5 equipped with an orthogonal pair of micrometers at a magnification of 100×.

**Figure 1.**
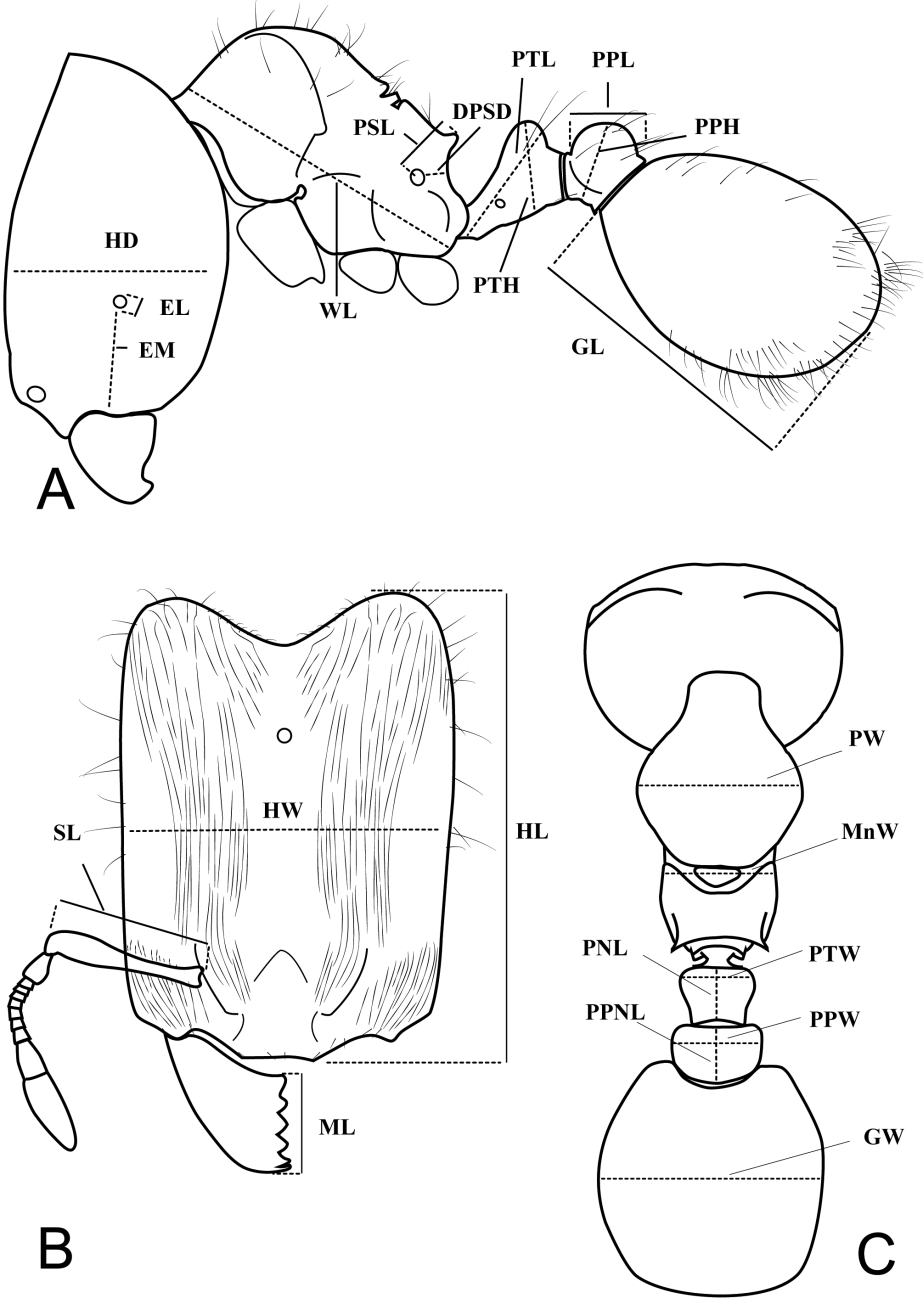
Standard measurements of *Carebara* minor and major workers. **A** Head depth (HD), eye length (EL), eye to mandible (EM), Weber’s length (WL), propodeal spine length (PSL), petiolar node height (PTH), petiole length (PTL), postpetiole height (PPH), postpetiole length (PPL), gaster Length (GL) **B** Head length (HL), head width (HW), scape length (SL), mandible length (ML) **C** Pronotal width (PW), petiolar node width (PTW), petiolar node length (PNL), postpetiole width (PPW), postpetiole node length (PPNL), gaster width (GW). *Carebara jajoby* (CASENT0037498).

The following terminology and abbreviations are used (Figure 1).

**HL** Head length: maximum length of head in full-face view between lines drawn across anterior margin of clypeus and lines drawn across the posterolateral corners of head.

**HW** Head width: width of head directly behind the eyes (if present) measured in full-face view.

**SL** Scape length: maximum scape length excluding basal condyle and neck.

**ML** Mandible length: the straight-line length of the mandible at full closure from the mandibular apex to the clypeal margin.

**EL** Eye length: maximum diameter of compound eye measured in oblique profile view.

**EM** Eye to mandible: distance from base of compound eye to the mandibular insertion, measured in profile view.

**HD** Head depth: maximum depth of head in profile view measured perpendicular to full-face view plane.

**WL** Weber’s length: diagonal length of mesosoma in profile view from the postero-ventral margin of the propodeal lobe to the anterior-most point of the pronotal slope, excluding the neck.

**PSL** Propodeal spine length: length of propodeal spine or posterodorsal corner of propodeum, measured from center of the propodeal spiracle to the tip of the spine or the posterodorsal corner of propodeum.

**PW** Pronotal width: maximum width of pronotum measured in dorsal view.

**MFL** Femur length: length of the profemur measured along its long axis in posterior view. Taken only from hind leg.

**MFW** Femur width: maximum width of the hind femur.

**MTL** Tibia length: maximum length of hind tibia.

**PTL** Petiole length: maximum length of petiole, measured in profile view.

**PTH** Petiolar node height: maximum height of petiolar node measured in profile view from the highest (median) point of the node to the ventral outline.

**PNL** Petiolar node length: maximun length of petiolar node measured in dorsal view.

**PTW** Petiolar node width: maximum width of dorsal face of petiolar node measured in dorsal view.

**PSD** Propodeal spiracle diameter: maximum length of propodeal spiracle in profile view.

**DPSD** Distance from propodeal spiracle to declivity: minimum distance from posterior border of propodeal spiracle to declivity of propodeum.

**PPL** Postpetiole length: maximum length of postpetiole measured in profile view.

**PPNL** Postpetiole node length: maximum length of postpetiole measured in dorsal view.

**PPH** Postpetiole height: maximum height of the postpetiole measured in profile view from the highest (median) point of the node to the ventral outline. The measuring line is placed at an orthogonal angle to the ventral outline of the node.

**PPW** Postpetiole width: maximum width of postpetiole measured in dorsal view.

**GL** Gaster length: maximum length of the gaster measured in profile view.

**GW** Gaster width: maximum width of the gaster measured in dorsal view.

**CI** Cephalic index: 100*HW/HL.

**MI** Mandibular index: 100*ML/HL.

**SI** Scape index: 100*SL/HL.

**MLI** Metafemur length index: 100*FL/HL.

**PPLI** Postpetiole length index: 100*PPL/PTL.

**PPI** Postpetiole width index: 100*PPW/PTW.

**PSI** Propodeal spine index: 100*PSL/HW.

### Morphological diagnosis of the worker caste of *Carebara* (adapted from, and with additions to Fischer et al. 2014).

1 Workers polymorphic, dimorphic or monomorphic, allowing a division of the worker caste into smaller workers (minor worker) and larger workers (major worker). The major worker subcaste shows size differences, with *intermediates* between small and large major workers. Some species of the Old World with phragmotic heads in the major worker subcaste.

2 Eyes either absent or present (when present, small to medium sized, from 1–75 ommatidia). Major workers of some Malagasy species present one or more ommatidia in specimens from the same colony.

3 One to three ocelli occasionally developed in the major worker subcaste (*intermediates*). The number of ocelli is related to the size of the major worker.

4 Antennae with 8–11 segments, with a two-segmented club. In some Malagasy species (e.g. *C. jajoby*, *C. nosindambo*), the largest major worker (intermediate) may have one additional segment (11 segments) than medium, smaller major workers, and minor workers in the same nest series (10 segments).

5 Palpal formula 2,2 and 1,2.

6 Mandible subtriangular or triangular with four to seven teeth or denticles (three teeth in *C. crigensis* (Belshaw and Bolton 1994)).

7 Clypeus with median area well defined, inserted between the frontal carinae, with rugae on the frontal carinae continuing to the anterior margin of the clypeus.

8 Clypeus of minor workers usually with four distinct setae (some species or specimens, e.g. *C. carinata*, *C. sangi*, *C. lignata*, with a central, isolated seta).

9 Frontal carinae covering the antennal sockets and often bases of the scapes.

10 Short antennal scrobe present on phragmotic major workers.

11 Head of major worker rarely bearing a pair of short, forwardly-directed horns or transverse ridges on the posterolateral lobes, or with posterolateral corners well developed.

12 Promesonotal suture usually distinct only laterally to the height of the spiracle, absent on dorsum, but usually present in larger major workers (intermediates).

13 Metanotal groove distinct and deeply or shallowly impressed on the dorsum.

14 Propodeum with posterodorsal corner rounded, sharply angulate or dentate, propodeal carinae lamellar.

15 Propodeal spiracle usually round.

16 Petiole peduncle relatively short or long, node distinct, subpetiolar process usually stoutly dentate.

17 Postpetiole with distinct node but lower than petiole in profile view.

18 Sculpture variable, smooth and shiny, punctate, striate, rugose, reticulate or costate.

19 Color yellow to deep brown.

### Morphological characters

The following characters consistently showed variation among species: a) pilosity of the head, mesosoma and gaster: erect, suberect, subdecumbent, decumbent, and appressed, b) sculpture of the head: areolate, areolate-rugose, costate, punctate, rugose, and striate, c) cephalic index, d) form of the anterior and posterior margin of the petiole: flat, concave or convex, e) form of the dorsum of the petiole: flat, rounded or convex, f) form of the dorsum, anterodorsal and posterodorsal corners of the propodeum: rounded or convex, g) distance from propodeal spiracle to declivity of propodeum: we use the diameter of the propodeal spiracle for comparison (for example, same as half the diameter of the spiracle, or slightly longer than the diameter of the spiracle), h) abundance of pilosity on head and body, hair inclination, i) in intermediate castes, form and size of the head, mesosoma and petiole (see under intermediates), and j) head longer or slightly longer than wide: we use the following terms, subquadrate when head length/head width varies between 1 and 1.1, subrectangular when it varies between 1.2 and 1.4, and rectangular when is close to 1.5. These characters are more noticeable in the major worker subcaste; nevertheless, their variations provide important distinctions in both minor and major worker castes.

### Pilosity

The terminology we used to describe the inclination of pilosity follows Wilson (1955), who described five types of hair inclination. **In profile view**. a) **erect**: hairs that are vertical or nearly vertical to the cuticular surface (fully erect - 90°); b) **suberect**: hairs with an inclination of 70 degrees from the cuticular surface; c) **subdecumbent**: hairs with an inclination of 50 degrees from the cuticular surface; d) **decumbent**: hairs with an inclination of 30 degrees from the cuticular surface; and e) **appressed**: hairs with an inclination of 10 degrees or nearly parallel to the cuticular surface. The hairs described in this study may be of different sizes, in most cases the larger hairs are usually 2 to 3 times larger than the smaller hairs and have a different inclination than their smaller counterparts. Generally, the smaller hairs are more abundant than the larger ones. Hair inclination and abundance varies in larger major workers, (intermediates), usually in intermediates where ocellus and flight sclerites are present. Most of the *Carebara* species, especially minor workers due to the lack of easy characters, can only be separated from other species by the variation of pilosity in the scape, mesosoma and the gaster.

### Sculpture

The terminology we used to describe the sculpture follows Harris (1979): a) **areolate**: raised ridges that connect to form a number of small polygons; b) **areolate-rugose**: areolate with nonlinear raised ridges; c) **costate**: longitudinal parallel ridges; d) **punctate**: fine, impressed points or punctures appearing as pinpricks; e) **rugose**: nonlinear raised ridges; and f) **striate**: fine, parallel and longitudinal impressed lines or furrows. This character system is very useful to differentiate species of *Carebara* based on major workers. In the Malagasy region, the minor worker of *C. creolei* is the only one with the head and mesosoma completely sculptured.

### Petiole

The petiole varies greatly in shape and combined with pilosity and form of the posterodorsal corner of the propodeum, is one of the most useful characters for differentiating species in *Carebara*. **In profile view**, the main variations present among species are: a) ventral face: medially flat, convex or slightly concave; b) dorsum: convex, angulate, rounded, or almost flat; and c) combined outline of dorsal surface of peduncle and anterior face of node, and posterior face of petiolar node: concave or straight.

### Mesosoma

In profile view, a) promesonotum: rounded, convex, slightly convex to nearly flat; b) metanotal groove: present, deeply or weakly impressed; c) dorsum of propodeum: flat, concave, slightly concave, convex or slightly convex; d) posterodorsal corner of propodeum: armed or unarmed, rounded, convex, angulate, or with a pair of triangular teeth; e) declivity of propodeum: flat, concave, or slightly concave; f) propodeal lobe: rounded, convex or triangular; g) propodeal spiracle: rounded, oval, closer to mid-height and mid-length of lateropropodeum in larger major workers, distance from posterior border of spiracle to declivity varies between species. In dorsal view, g) dorsum of propodeum: smooth and shiny or sculptured.

### Intermediates

The presence of intermediates (major workers with distinct forms, larger major workers with flight sclerites or ocelli) in the major worker subcaste has been reported previously for *C. bruchi* (formerly *Oligomyrmex*), *C. diversa* (formerly *Pheidologeton*) (Kusnezov, 1951), *C. corniger* (Ettershank, 1966), *C. coeca*, *C. tenua*, and *C. coqueta* (Fernández 2004, 2006). In this work, we report that most *Carebara* species from the Malagasy region present a complex variation in the major worker subcaste (some species with four distinct forms, with the largest major worker morphologically similar to queens). Larger major workers can be thus considered intercastes, because of their similarities to queens (Peeters 1991). The presence of *intermediates* is similar to that reported for the *C. polita* species group (Fischer et al. 2014), further supporting the synonymization of *Pheidologeton* under *Carebara*. The ant genera *Carebara*, *Oligomyrmex*, and *Pheidologeton*, where separated mainly because they were monomorphic, dimorphic and polymorphic, respectively, but recent discoveries show that *Carebara* present a complex morphological variation, so this character was not longer useful to split those genera.

In addition to the variation in the major worker caste, a recent study showed that some *Carebara* species from the Old World, including *C. elmenteitae*, *C. butteli*, *C. lilith*, *C. nayana*, and *C. phragmotica*, possess major workers with phragmotic heads (Fischer et al. 2015). However, despite all of the intensive collections carried out in the Malagasy region, none of the Malagasy *Carebara* species has been found to have major workers with phragmotic heads. The discoveries reported by Fischer et al. (2014, 2015), and in this work, indicate that the use of standardized methods to collect ants (see Fisher 2005) may help to discover other *Carebara* species with *intermediates* or with phragmotic heads in the major worker caste. Future studies could help to better understand the evolution of caste intergradations (Baroni Urbani and Passera 1996, Ward 1997, Molet et al. 2012).

Most *Carebara* species from the Malagasy Region show a remarkable range of morphological diversity, and this is especially noticeable in the major worker subcaste. In some cases, like *C. jajoby* sp. n., and *C. nosindambo* Forel, there are four recognizable “intermediates” in the major worker subcaste (from the smallest, intermediate 1, to the largest, intermediate 4) in the same colony (Figures 44, 53). Morphologically these intermediates are mosaics between major workers and queens. Intermediates are reported for the following Malagasy species: *C. bara* sp. n., *C. grandidieri* Forel, *C. jajoby* sp. n., *C. kabosy* sp. n., *C. lova* sp. n., *C. malagasy* sp. n., *C. nosindambo* Forel, *C. omasi* sp. n., *C. placida* sp. n., *C. raberi* sp. n., *C. sampi* sp. n., and *C. vazimba* sp. n. The main variations present in the intermediates from the smallest to the largest are: a) posterolateral corners of head: moderately to strongly expanding posteriorly; b) posterior margin of head: weakly to strongly concave; c) size of the eyes: in some species from one to 75 ommatidia; d) number of ocelli: from one to three; e) form of the mesosoma: showing remnants of queen flight sclerites; f) propodeal spiracle: distance of propodeal spiracle to declivity of propodeum closer to midlength of propodeal sclerite in larger intermediates; g) hair abundance; and h) sculpture: weakly to strongly sculptured. For the species with intermediates, we did not present a separate description for each intermediate form, but instead only a general discussion of all the forms present in the species. All the variations present in the intermediates were included in the identification key, and can be separated using the major worker key. In some cases, larger intermediates are similar in the form of the head, but they differ in the form of the propodeum, petiole, as well as in the pilosity, so the user should check all characters mentioned on each part of the key.

### Biology

**Figure 2.**
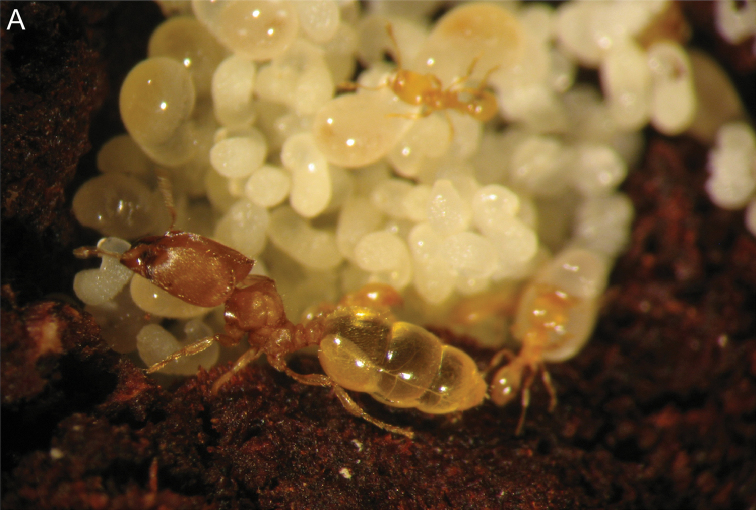
**A** Major worker (Intermediate 4) of *Carebara jajoby* (Image by Christian Peeters).

*Carebara* characteristically have both very small minor workers and extremely large major workers. The genus contains one of the smallest ants in the world (minor worker of Neotropical *C. minuta* has a head width of 0.21 mm, and total length near 1 mm, while in the Malagasy region the smallest *Carebara* species is the minor worker of *C. creolei* sp.n. with a head width of 0.26 mm and a total length near 1.2 mm). The largest major worker in the genus *Carebara* (major worker of *C. diversus* – formerly *Pheidologeton*) can reach a total length of 16 mm, while in the Malagasy region the largest *Carebara* is the major worker of *C. jajoby* sp.n. with a total length of 4.53 mm (Figures 2, 44). Workers may be monomorphic, dimorphic or polymorphic. Polymorphism is more noticeable in the major worker caste, with some species having four sizes of major workers (e.g. *C. jajoby* sp. n. and *C. nosindambo*). We did not report any major worker smaller than the minor worker, but this phenomenon may occur in other species of *Carebara* (Baroni Urbani 1998). The main differences in the morphology of these intermediates include: size differences in the head, posterolateral corners of head, and thorax; larger major workers with one to three ocelli (ocelli are reduced compared to the queen caste); reduced flight sclerites; and eye size differences. It is possible that the intermediates in the major worker caste are trophic specialists like the intermediates of the ant genus *Crematogaster* (see Peeters et al. 2013). Colonies can be large, e.g. colonies of *C. overbecki* and *C. urichi* contain up to 1000 individuals (minor and major workers), with the proportion of major workers approaching ten percent (Moffett 1986; Wilson 1962). Minor workers nurse the brood, and major workers defend the nest. The diet of *Carebara* includes mites, entomobryid collembolans and arthropod eggs (Wilson 1962, 1986), and according to Fischer (2012), six *Carebara* species from Kenya (Kakamega forest) have specialized predatory diets.

### Distribution

**Figure 3.**
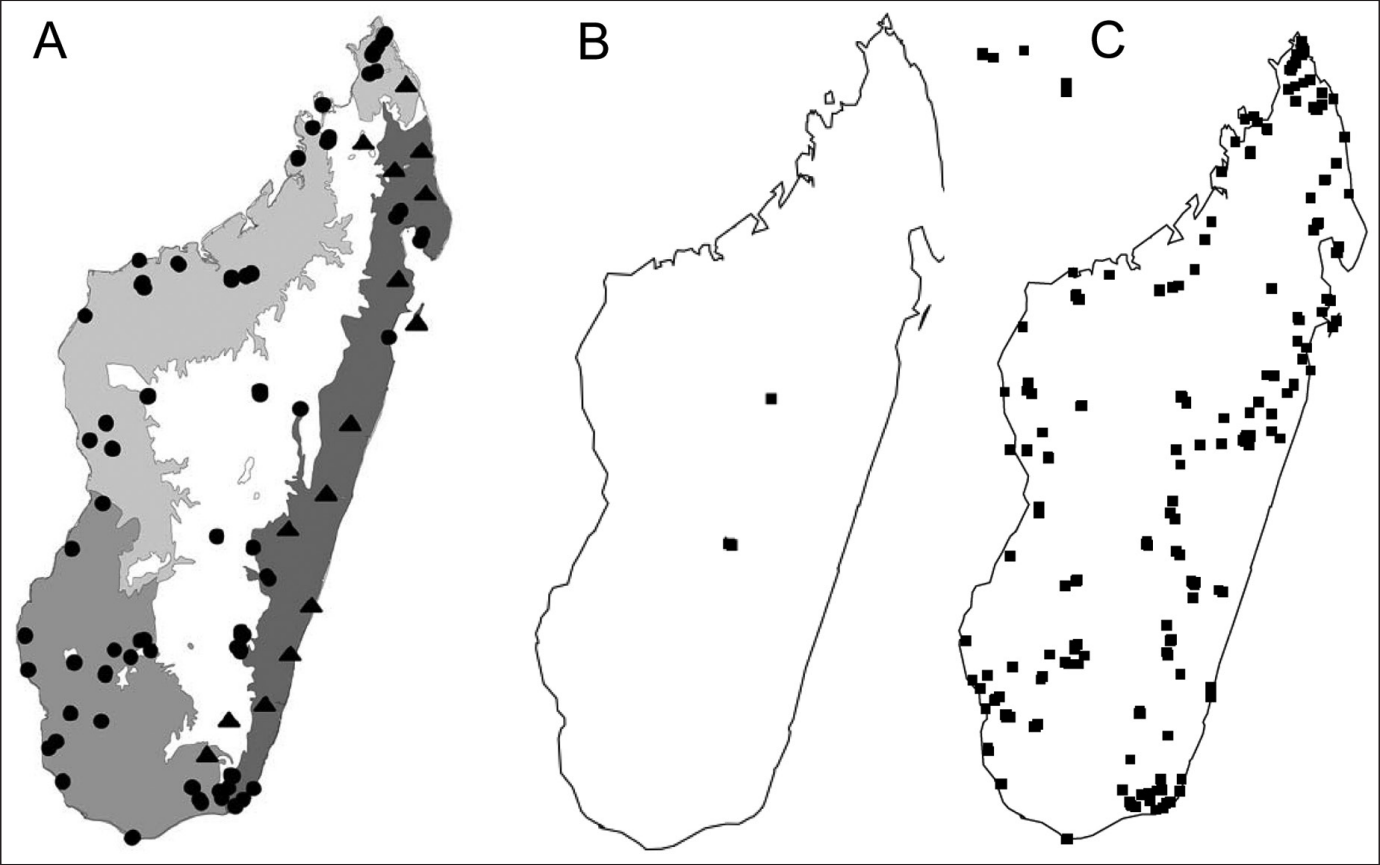
**A** Localities for field collecting **B** *Carebara* records in 2012 **C** Distribution of *Carebara* in the Malagasy region in 2016.

The ant genus *Carebara* is distributed worldwide, present mainly in tropical and subtropical regions, with approximately 253 described taxa (230 species and 23 subspecies), and the Indomalaya region seems to have more species of *Carebara* than the other biogeographic regions: 71 species and 14 subspecies (Bolton, 2017). Before this work only three species were recorded for the Malagasy region; these records were reported in the provinces of Fianarantsoa (*C. nosindambo*), Antananarivo (*C. grandidieri*) and Toamasina (*C. voeltzkowi*). Since 1992 and using a variety of standardized methods for ant collections, ants were collected across all Malagasy region (see Fisher, 2005), and based on the revision of specimens collected since 1992, we report 23 species. *Carebara* was recorded in almost every habitat in the Malagasy region where an ant collection was made (Figure 3). Most of the *Carebara* species recorded in this work are endemic to a specific habitat (ecoregion): for example, *C. creolei* sp. n. is known only from Mauritius and Seychelles, *C. grandidieri* Forel, which is widely distributed in Madagascar, is also present in Comoros and Mayotte. The other *Carebara* species are restricted to Madagascar. Five of them are confined to a specific province, ecoregion or habitat: *C. berivelo* sp. n., *C. betsi* sp. n., *C. mahafaly* sp. n., *C. malagasy* sp. n., and *C. vazimba* sp. n. Six species are known only from their type locality (*C. demeter* sp. n., *C. placida* sp. n., *C. raberi* sp. n., *C. salegi* sp. n., *C. tana* sp. n., *C. tanana* sp.n.). Six species were collected in more than one province or habitat: *C. dota* sp. n., *C. hainteny* sp. n., *C. hiragasy* sp. n., *C. lova* sp. n., *C. omasi* sp. n., *C. sampi* sp. n., while other five species are widespread and were collected across all major habitats of Madagascar: *C. bara* sp. n., *C. grandidieri* Forel, *C. jajoby* sp. n., *C. kabosy* sp. n., and *C. nosindambo* Forel (Figures 68, 69). Three species were collected only above 1000 m (*C. demeter* sp. n., and *C. omasi* sp. n., and *C. tanana* sp.n.), seven species were recorded below 600 m (*C. berivelo* sp. n., *C. creolei* sp. n., *C. lova* sp. n., *C. malagasy* sp. n., *C. placida* sp. n., *C. sampi* sp. n., and *C. vazimba* sp. n.), while some species were recorded below 600 m and above 1000 m (*C. grandidieri* Forel, *C. betsi* sp. n., *C. dota* sp. n., *C. jajoby* sp. n., and *C. nosindambo* Forel).

### Abbreviations of depositories

**BMNH** The Natural History Museum (British Museum, Natural History), London, U.K.

**CASC** California Academy of Sciences, San Francisco, California, U.S.A.

**MCZ** Museum of Comparative Zoology, Harvard University, Cambridge, Massachusetts, U.S.A.

**MHNG** Muséum d’Histoire Naturelle de la Ville de Genève, Geneva, Switzerland.

**MNHB** Museum of Natural History of Humboldt University in Berlin, Germany.

**PBTZ** Parc Botanique et Zoologique de Tsimbazaza, Antananarivo, Madagascar.

### Synopsis of the *Carebara* species of the Malagasy region

*Carebara bara* Azorsa & Fisher, **sp. n.**

*Carebara berivelo* Azorsa & Fisher, **sp. n.**

*Carebara betsi* Azorsa & Fisher, **sp. n.**

*Carebara creolei* Azorsa & Fisher, **sp. n.**

*Carebara demeter* Azorsa & Fisher, **sp. n.**

*Carebara dota* Azorsa & Fisher, **sp. n.**

*Carebara grandidieri* (Forel, 1891)

= *Oligomyrmex voeltzkowi* Forel, 1907, **syn. n.**

*Carebara hainteny* Azorsa & Fisher, **sp. n.**

*Carebara hiragasy* Azorsa & Fisher, **sp. n.**

*Carebara jajoby* Azorsa & Fisher, **sp. n.**

*Carebara kabosy* Azorsa & Fisher, **sp. n.**

*Carebara lova* Azorsa & Fisher, **sp. n**.

*Carebara mahafaly* Azorsa & Fisher, **sp. n.**

*Carebara malagasy* Azorsa & Fisher, **sp. n.**

*Carebara nosindambo* (Forel, 1891)

*Carebara omasi* Azorsa & Fisher, **sp. n.**

*Carebara placida* Azorsa & Fisher, **sp. n.**

*Carebara raberi* Azorsa & Fisher, **sp. n.**

*Carebara salegi* Azorsa & Fisher, **sp. n.**

*Carebara sampi* Azorsa & Fisher, **sp. n.**

*Carebara tana* Azorsa & Fisher, **sp. n.**

*Carebara tanana* Azorsa & Fisher, **sp. n.**

*Carebara vazimba* Azorsa & Fisher, **sp. n.**

### Key to Malagasy species of *Carebara* based on major worker

**Table:** 

1	Antennae 9-segmented	**2**
–	Antennae 10- or 11-segmented	**3**
2	Mandible with six teeth; dorsum of head and mesosoma smooth and shiny, genae and frontal lobes anteriorly with short longitudinal rugulae, not surpassing mid-length of head; head relatively long (HL 0.56–0.84) (Fig. 4A)	***C. grandidieri***
–	Mandible with five teeth, dorsum of head and mesosoma areolate-rugose, genae and frontal lobes with long longitudinal rugulae, surpassing mid-length of head, head relatively short (HL 0.45–0.53) (Fig. 4B)	***C. creolei***
3	Posterodorsal corner of propodeum angulate or convex, without a triangular tooth, or armed with at least a dentiform angle (Fig. 5A, B)	**4**
–	Posterodorsal corner of propodeum with a triangular tooth (Fig. 5C, D)	**8**
4	Dorsum of head with well-defined longitudinal rugae on the frons, extending to the posterior margin (Fig. 6A); head laterally with longitudinal rugae extending from mandibular insertion to posterolateral corner of head	***C. placida***
–	Dorsum of head with at least the frons smooth and shiny (Fig. 6B, C, D); head laterally with fine longitudinal rugulae extending from mandibular insertions to about eye level or slightly beyond	**5**
5	In full-face view posterolateral corners of head narrowing posteriorly and their prominent appearance enhanced by the relatively deep median impression of the posterior margin (Fig. 6D)	***C. malagasy***
–	In full-face view posterolateral corners of head evenly rounded, not prominent, their short, broad appearance enhanced by the relatively shallow median impression of the posterior margin (Fig. 6B, C)	**6**
6	In full-face view, mandible with six teeth; in profile, combined outline of dorsal surface of peduncle and anterior face of node medially concave; anterodorsal corner of node more broadly rounded, petiole node longer (PNL 0.12–0.15); petiole and postpetiole with all hairs suberect to subdecumbent (Fig. 7C); dorsum of propodeum with more than ten long erect to suberect hairs	***C. hiragasy***
–	In full-face view, mandible with five teeth; in profile, combined outline of dorsal surface of peduncle and anterior face of node nearly flat (Fig. 7A, B); anterodorsal corner of node convex or narrowly rounded, petiole node short (PNL 0.08–0.11); dorsum of propodeum with fewer than ten erect or suberect hairs	**7**
7	In full-face view, dorsum of head with well defined longitudinal rugae laterally, and smooth and shiny middle area; in profile gaster with abundant long suberect hairs; distance from propodeal spiracle to declivity less or same as the diameter of the spiracle (Fig. 7A)	***C. tanana***
–	In full-face view, dorsum of head smooth and shiny or with fine longitudinal rugae laterally, smooth and shiny frons; in profile gaster with short decumbent to subdecumbent hairs and long subdecumbent to suberect hairs; distance from propodeal spiracle to declivity same or greater than the diameter of the spiracle (Fig. 7B)	***C. bara***
8	In full-face view, lateral margins of head straight or weakly convex, when weakly convex posterolateral corners nearly rounded (Fig. 8A, B, C, D)	**9**
–	In full-face view, lateral margins of head convex or nearly straight, when nearly straight or weakly convex posterolateral corners of head more prominent and narrowing posteriorly (Fig. 8E, F, G H), when lateral margins are convex like (Fig. 8E) then posterolateral corners rounded	**16**
9	Dorsal surface of head not completely sculptured, frons smooth and shiny, dorsolateral face of head with longitudinal rugae, mandible with five or six teeth (Fig. 9A, B)	**10**
–	Dorsal surface of head sculptured, when median area of head not completely sculptured or smooth and shiny, head with longitudinal and irregular rugae and reticulate-rugose near the posterior margin, mandible with five teeth (Fig. 9C, D)	**13**
10	In full-face view, head without transverse rugae near posterior margin of head, mandible with six teeth (Fig. 9A)	**11**
–	In full-face view, head with transverse rugae near posterior margin of head, mandible with five teeth (Fig. 9B)	**12**
11	In full-face view, head with longitudinal rugae, not extending to posterior margin of head, posterolateral lobes weakly sculptured, almost smooth and shiny; in profile, combined outline of dorsal surface of peduncle and anterior face of node nearly flat; posterior face of petiolar node nearly flat (Fig. 10A); distance from propodeal spiracle to declivity twice the diameter of the spiracle; posterolateral portion of cephalic dorsum lacking sculpture	***C. omasi***
–	In full-face view, head with longitudinal rugae, extending to posterior margin of head, posterolateral lobes sculptured; in profile, combined outline of dorsal surface of peduncle and anterior face of node medially concave; posterior face of petiolar node convex (Fig. 10B); distance from propodeal spiracle to declivity less than twice the diameter of the spiracle; posterolateral portion of cephalic dorsum sculptured	***C. nosindambo***
12	In profile, posterior face of petiolar node vertical to weakly concave; petiolar node short (PNL 0.07–0.11) (Fig. 11A); distance from propodeal spiracle to declivity equal to the diameter of the spiracle; declivity of propodeum flat	***C. berivelo***
–	In profile, posterior face of petiolar node steeply sloping and weakly convex; petiolar node long (PNL 0.11–0.12) (Fig. 11B); distance from propodeal spiracle to declivity less than the diameter of the spiracle; declivity of propodeum concave	***C. tana***
13	In profile, length of propodeal tooth less than the diameter of the propodeal spiracle; dorsum of propodeum slightly convex	**14**
–	In profile, length of propodeal tooth equal to, or slightly greater than the diameter of the propodeal spiracle; dorsum of propodeum flat	**15**
14	Dorsum of head with well-defined longitudinal rugae; irregular and transverse rugae present close to posterolateral corners and medially on posterior margin of head (Fig. 12A)	***C. dota***
–	Dorsum of head with fine longitudinal and irregular rugae laterally, rugae weakly defined on frons, rugoreticulum present close to posterior margin of head (Fig. 12 B)	***C. demeter***
15	In full-face view, posterolateral corners of head with a small triangular, tooth-like horn; dorsum of head longitudinally rugose laterally, becoming rugoreticulate medially, with a strong median longitudinal ruga reaching almost to the posterior margin of the head (Fig. 13A)	***C. hainteny***
–	In full-face view, posterolateral corners of head without teeth; dorsum of head with longitudinal rugae directed to posterolateral corners of head, without a distinctly stronger median ruga (Fig. 13B)	***C. betsi***
16	Lateral margins of head convex; occipital margin shallowly to moderately concave medially and posterolateral corners not prominent, rounded and not narrowed posteriorly (Fig. 14A, B)	**17**
–	Lateral margins of head weakly convex, straight or nearly so medially; occipital margin deeply concave medially and posterolateral corners prominent, narrowed posteriorly (Fig. 14C, D)	**18**
17	Posterodorsal corner of propodeum with a laminate triangular tooth that is slightly longer than the maximum propodeal spiracle diameter (PSL 0.08–0.09); propodeal spiracle oval and separated by almost twice the diameter of the spiracle from the propodeal declivity (Fig. 15A)	***C. mahafaly***
–	Posterodorsal corner of propodeum with a triangular tooth that is shorter than the propodeal spiracle diameter (PSL 0.05); propodeal spiracle round and separated by less than half the diameter of the spiracle from the propodeal declivity (Fig. 15B)	***C. salegi***
18	In full face-view, dorsum of head smooth and shiny, or with fine longitudinal rugae laterally, extending from frontal lobes to midlenght of head, but never reaching to apices of frontal carinae and smooth and shiny frons (Fig. 16A, B, C)	**19**
–	In full face-view, dorsum of head with well-defined longitudinal rugae, extending from frontal lobes to apices of frontal carinae, except for smooth and shiny frons (Fig. 18A, B, C)	**21**
19	In profile, propodeal dorsum flat and slopes at about 30 degrees compared to the ventral margin of the metapleura, gaster with abundant short decumbent hairs; in dorsal view, anterior margin of pronotum sculptured, weakly areolate (Fig. 16E)	***C. raberi***
–	In profile, propodeal dorsum flat and slopes at about 45 degrees compared to the ventral margin of the metapleura; gaster with few short decumbent or appressed hairs; in dorsal view, anterior margin of pronotum smooth and shiny, in some cases weakly sculptured with short longitudinal rugae (Fig. 16D, F)	**20**
20	In full face-view, posterolateral corners of head narrowed posteriorly (Fig. 16 A); propodeal tooth triangular and barely longer than propodeal spiracle, distance from posterior border of propodeal spiracle to declivity less than two times the diameter of the spiracle; combined outline of dorsal surface of peduncle and anterior face of node weakly concave (Fig. 17A)	***C. sampi***
–	In full-face view, posterolateral corners of head not as narrowed as above (Fig. 16C); propodeal tooth triangular and short, rarely longer than propodeal spiracle, distance from posterior border of propodeal spiracle to declivity same as or less than the diameter of the spiracle; combined outline of dorsal surface of peduncle and anterior face of node concave medially (Fig. 17B)	***C. lova***
21	In profile, propodeal tooh triangular, well defined, and longer than the diameter of the propodeal spiracle; narrow petiolar peduncle, anterodorsal face of petiolar node weakly convex or straight; in full-face view head usually heart shaped, posterolateral corners of head narrowed anteriorly, head weakly narrowed anteriorly (Fig. 18A, D)	***C. jajoby***
–	In profile, propodeal tooth triangular, and shorter or barely longer than the diameter of the propodeal spiracle; broad petiolar peduncle, anterodorsal face of petiolar node, convex or rounded; in full-face view head less heart shaped, posterolateral corners of head nearly rounded, head not narrowed anteriorly (Fig. 18B, C, E, F)	**22**
22	In profile, petiolar peduncle tapering evenly from the node toward its anterior articulation, appearing slender anteriorly, dorsal margin of peduncle curving evenly into anterior margin of node, anterodorsal margin of petiole convex; propodeal lobes short and weakly convex, propodeal tooth shorter than propodeal spiracle diameter (Fig. 19A)	***C. vazimba***
–	In profile, petiolar peduncle not tapering, remaining deep almost to the anterior articulation and here narrowing abruptly, junction of dorsal margin of peduncle and anterior margin of node distinctly inflected, anterodorsal margin of petiole rounded; propodeal lobes short and weakly triangular, propodeal tooth slightly longer than propodeal spiracle diameter (Fig. 19B)	***C. kabosy***

**Figure 4.**
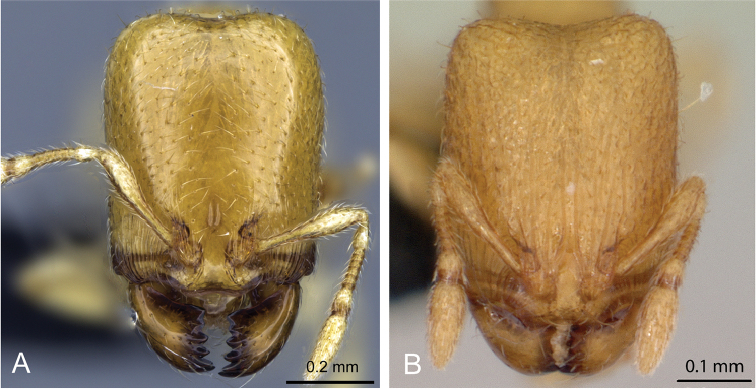
Head in full-face view of **A** *Carebara grandidieri* (CASENT0444653) **B** *Carebara creolei* (CASENT0060366).

**Figure 5.**
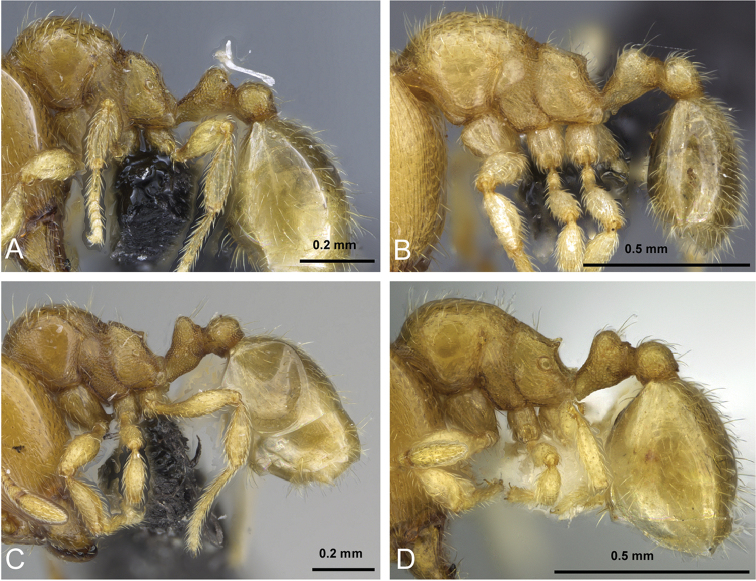
Body in profile view of **A** *Carebara malagasy* (CASENT0042186) **B** *Carebara placida* (CASENT0070817) **C** *Carebara omasi* (CASENT0027898) **D** *Carebara berivelo* (CASENT0438281).

**Figure 6.**
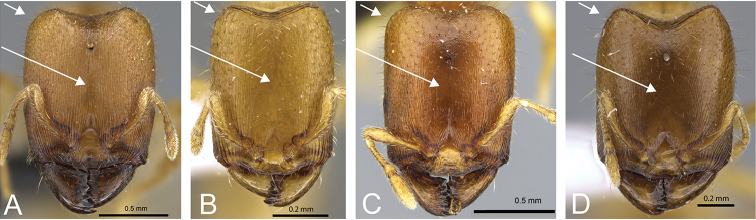
Head in full-face view of **A** *Carebara placida* (CASENT0070818) **B** *Carebara bara* (CASENT0439907) **C** *Carebara hiragasy* (CASENT0496886) **D** *Carebara malagasy* (CASENT0438154).

**Figure 7.**
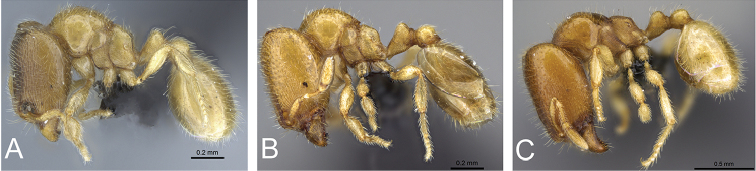
Petiole and postpetiole in profile view of **A** *Carebara tanana* (CASENT0211708) **B** *Carebara bara* (CASENT0483010) **C** *Carebara hiragasy* (CASENT0496886)

**Figure 8.**
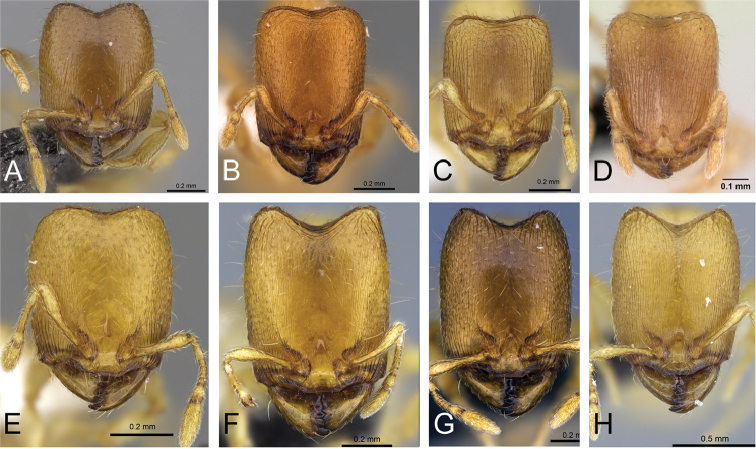
Head in full-face view of **A** *Carebara omasi* (CASENT0027898) **B** *Carebara berivelo* (CASENT0438188) **C** *Carebara dota* (CASENT0127649) **D** *Carebara betsi* (CASENT0479444) **E** *Carebara salegi* (CASENT0438150) **F** *Carebara sampi* (CASENT0030489) **G** *Carebara vazimba* (CASENT0055639) **H** *Carebara jajoby* (CASENT0037505).

**Figure 9.**
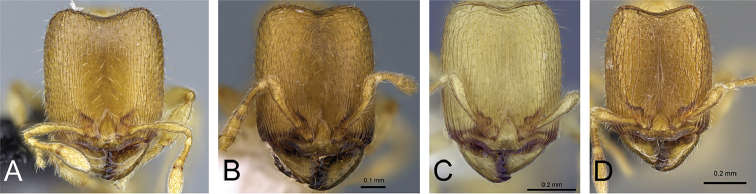
Head in full face view of **A** *Carebara nosindambo* (CASENT0072626) **B** *Carebara tana* (CASENT0437819) **C** *Carebara dota* (CASENT0192623) **D** *Carebara hainteny* (CASENT0039833).

**Figure 10.**
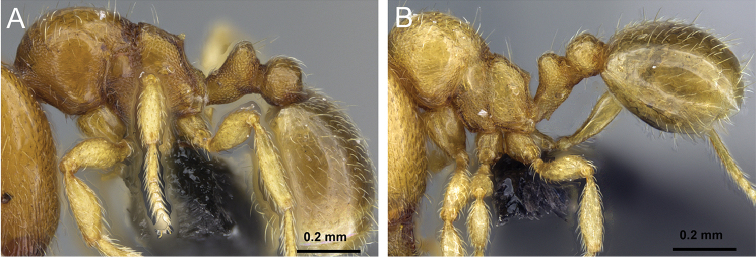
Petiole and postpetiole in profile view of **A** *Carebara omasi* (CASENT0028862) **B** *Carebara nosindambo* (CASENT0073096).

**Figure 11.**
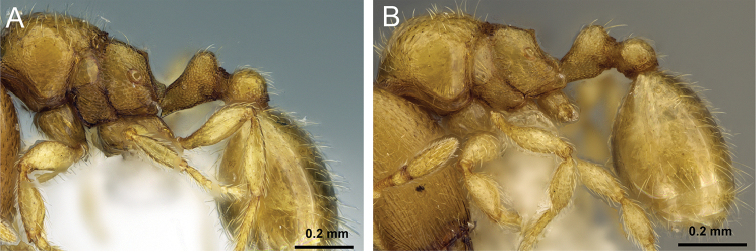
Petiole and postpetiole in profile view of **A** *Carebara berivelo* (CASENT0438188) **B** *Carebara tana* (CASENT0437819).

**Figure 12.**
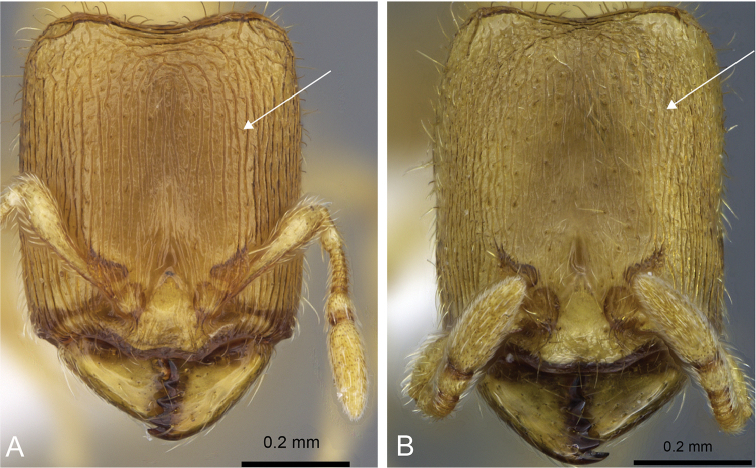
Head in full-face view of **A** *Carebara demeter* (CASENT0192622) **B** *Carebara dota* (CASENT0127649).

**Figure 13.**
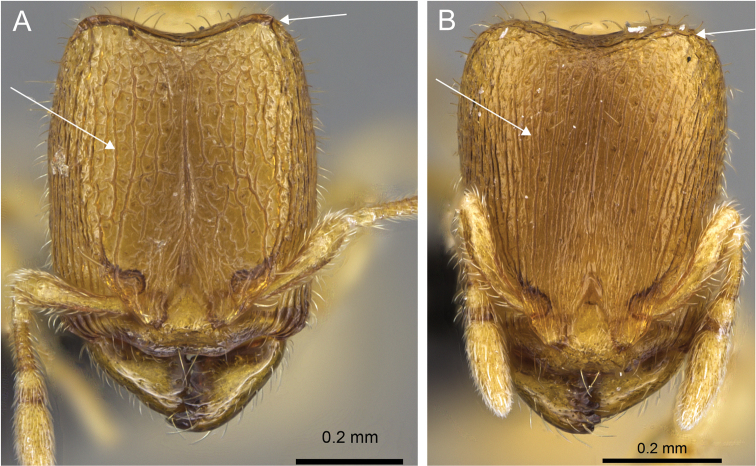
Head in full-face view of **A** *Carebara hainteny* (CASENT0039833) **B** *Carebara betsi* (CASENT0479444).

**Figure 14.**
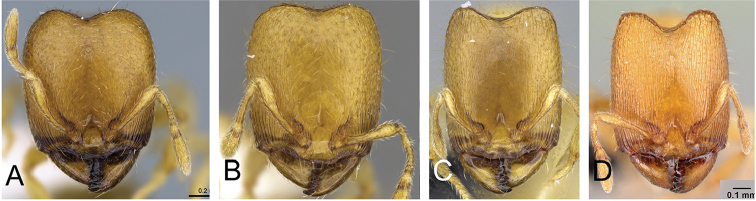
Head in full-face view of **A** *Carebara mahafaly* (CASENT0410502) **B** *Carebara salegi* (CASENT0438150) **C** *Carebara lova* (CASENT0036019) **D** *Carebara kabosy* (CASENT0042428).

**Figure 15.**
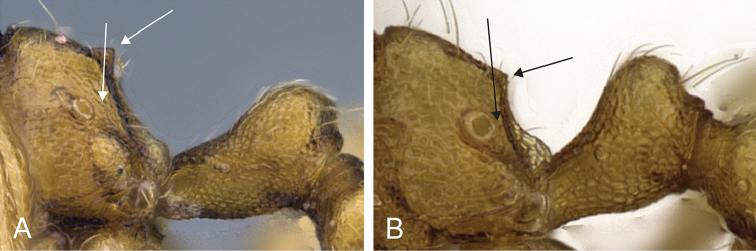
Propodeum and petiole in profile view of **A** *Carebara mahafaly* (CASENT0410502) **B** *Carebara salegi* (CASENT0438150).

**Figure 16.**
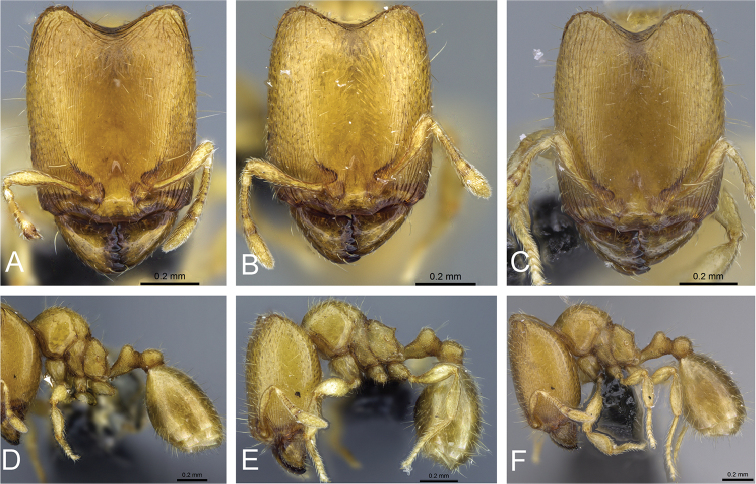
Head in full face view of **A, D** *Carebara sampi* (CASENT0030489) **B, E** *Carebara raberi* (CASENT0056921) **C, F** *Carebara lova* (CASENT0035923).

**Figure 17.**
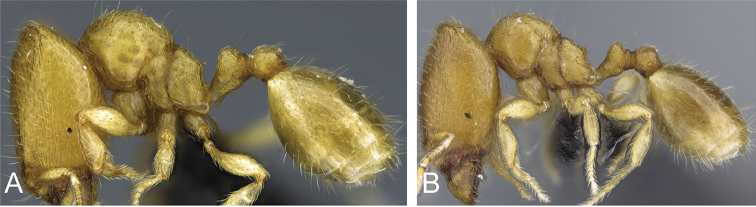
Mesosoma, petiole and postpetiole in profile view of **A** *Carebara sampi* (CASENT0030374) **B** *Carebara lova* (CASENT0470762).

**Figure 18.**
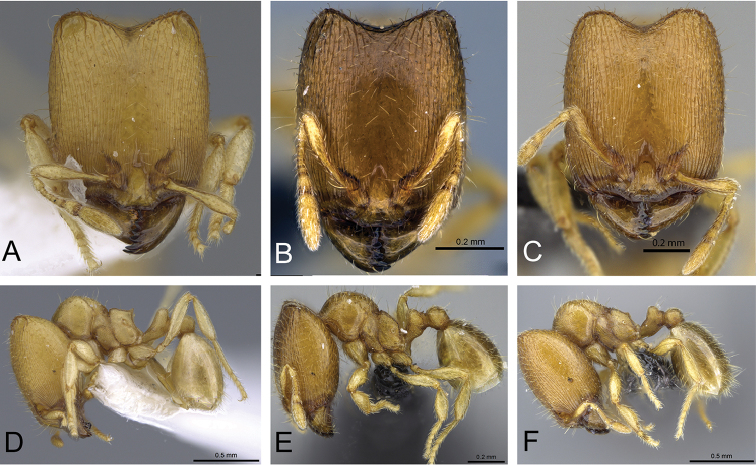
Head in full face-view of **A, D** *Carebara jajoby* (CASENT0192567) **B, E** *Carebara vazimba* (CASENT0055650) **C, F** *Carebara kabosy* (CASENT0162282).

**Figure 19.**
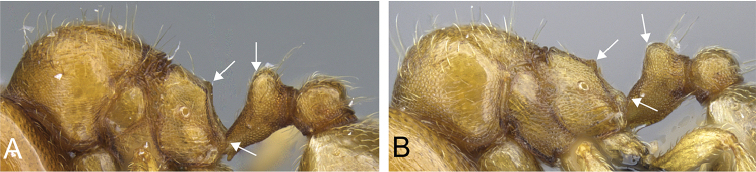
Mesosoma, petiole and postpetiole in profile view of **A** *Carebara vazimba* (CASENT0055532) **B** *Carebara kabosy* (CASENT0162282).

### Key to Malagasy species of *Carebara* based on minor worker

**Table:** 

1	Antennae 9-segmented	**2**
–	Antennae 10- or 11-segmented	**3**
2	Head somewhat longer (HL 0.39–0.49), with the dorsum smooth (Fig. 20A)	***Carebara grandidieri***
–	Head somewhat shorter (HL 0.31–0.33), with the dorsum sculptured (Fig. 20B)	***C. creolei***
3	Head and mesosoma with abundant erect and suberect, long and short hairs, more than 25 hairs on posterior margin of head, and more than 32 hairs on dorsal face of mesosoma; in profile, posterodorsal corner of propodeum convex and unarmed; petiolar node thick, petiole and postpetiole with abundant erect and suberect hairs (Fig. 21B, C)	**4**
–	Head and mesosoma with few decumbent to erect, long and short hairs, with fewer than 20 hairs on posterior margin of head, and fewer than 25 hairs on dorsal face of mesosoma; in profile, posterodorsal corner of propodeum armed with at least a dentiform angle or with a distinct triangular tooth, or if propodeum convex and unarmed then with less than 6 long erect hairs on mesosoma; petiole and postpetiole with few subdecumbent to apressed hairs (Fig. 21A)	**5**
4	Dorsum of propodeum nearly flat; mesosoma somewhat longer (WL 0.50–0.57); distance from center of propodeal spiracle to posterodorsal corner of propodeum relatively large (PSL 0.03–0.04); mandible length relatively large (MI 27–31) (Fig. 22A)	***C. hiragasy***
–	Dorsum of propodeum slightly convex; mesosoma somewhat shorter (WL 0.45–0.47); distance from center propodeal spiracle to posterodorsal corner of propodeum short (PSL 0.02–0.03); mandible length relatively short (MI 15–24) (Fig. 22B)	***C. placida***
5	In profile, posterodorsal corner of propodeum evenly convex and unarmed, without trace of a prominent dentiform angle or tooth (Fig. 23A)	***C. malagasy***
–	In profile, posterodorsal corner of propodeum armed with at least a dentiform angle, or with a distinct triangular tooth (Fig. 23B, C, D)	**6**
6	In profile, posterodorsal corner of propodeum at least with a dentiform angle, more usually the corner with a pair of small, low, angulate teeth (Fig. 23B, C)	**7**
–	In profile, posterodorsal corner of propodeum armed with a conspicuous pair of triangular teeth (Fig. 23D)	**11**
7	In profile, combined outline of dorsal surface of peduncle and anterior face of node weakly concave, and outline of dorsal surface of propodeum weakly concave (Fig. 24B)	***C. demeter***
–	In profile, combined outline of dorsal surface of peduncle and anterior face of node flat; outline of dorsal surface of propodeum flat, or weakly convex and declining posteriorly (Fig. 24C, D, E, F)	**8**
8	In profile, outline of dorsal surface of propodeum flat and declining posteriorly at about 40 degrees compared to the ventral margin of the metapleura; posterodorsal corner of propodeum angulate or with a pair of laminate triangular teeth, directed upward (Fig. 24D)	***C. dota***
–	In profile, outline of dorsal surface of propodeum convex or weakly convex and declining posteriorly at about 30 degrees compared to the ventral margin of the metapleura, posterodorsal corner of propodeum at least with a dentiform angle (Fig. 24C, E, F)	**9**
9	In profile, gaster with few short decumbent to subdecumbent hairs and long subdecumbent hairs; petiolar node relatively low (PTH 0.09–0.10) (Fig. 24E)	***C. bara***
–	In profile, gaster with abundant subdecumbent hairs; petiolar node relatively high (PTH 0.11–0.12) (Fig. 24C, F)	**10**
10	In profile, distance from propodeal spiracle nearly close to declivity, less than half the diameter of the spiracle; petiolar node relatively narrow; declivity of propodeum weakly concave; gaster with more than 30 subdecumbent hairs (Fig. 24C)	***C. betsi***
–	In profile, distance from propodeal spiracle to declivity about half the diameter of the spiracle; petiolar node not narrowed; declivity of propodeum weakly convex; gaster with fewer than 25 subdecumbent hairs (Fig. 24F)	***C. tanana***
11	In profile, dorsum of propodeum and declivity convex or weakly convex (Fig. 25A, B, C, D)	**12**
–	In profile, dorsum of propodeum and declivity flat or concave (Fig. 25E, F, G, H)	**16**
12	In profile, combined outline of dorsal surface of peduncle and anterior face of node nearly straight; gaster with short appressed hairs, and without longer decumbent, suberect or erect hairs; propodeal teeth acutely triangular and slightly longer than propodeal spiracle diameter; petiolar node smooth and shiny (Fig. 26A)	***C. mahafaly***
–	In profile, combined outline of dorsal surface of peduncle and anterior face of node medially concave; gaster with long subdecumbent to suberect hairs, and short appressed or decumbent hairs; propodeal teeth short, weakly triangular, and not as long as propodeal spiracle diameter; petiolar node sculptured (Fig. 26B)	**13**
13	Hairs of the scape, posterior margin and lateral margins of head, abundant and subdecumbent; dorsum of promesonotum with abundant short suberect hairs (20–30 hairs); distance from center of the propodeal spiracle to declivity same or slightly longer than the diameter of the spiracle (Fig. 27A)	***C. salegi***
–	Hairs of the scape, posterior margin and lateral margins of head, decumbent or appressed; dorsum of promesonotum with short subdecumbent to suberect hairs (fewer than 20 hairs); distance from center of the propodeal spiracle to declivity less than the diameter of the spiracle (Fig. 27B, C, D)	**14**
14	Dorsum of propodeum weakly convex; posterodorsal corner with a pair of very short triangular teeth, length less than propodeal spiracle diameter (Fig. 28A)	***C. lova***
–	Dorsum of propodeum convex; posterodorsal corner of propodeum with a pair of triangular and laminate teeth, length same as propodeal spiracle diameter	**15**
15	Anterodorsal corner of petiole convex; side of propodeum areolate-rugose, propodeal lobes subtriangular and obtusely rounded; gaster with short decumbent to appressed hairs and long subdecumbent hairs (Fig. 28B)	***C. omasi***
–	Anterodorsal corner of petiole rounded; median area of side of propodeum smooth and shining, propodeal lobes short and convex; gaster with short decumbent to subdecumbent hairs and long subdecumbent to suberect hairs (Fig. 28C)	***C. nosindambo***
16	In profile, declivity of propodeum concave to weakly concave (Fig. 28D, F)	**17**
–	In profile, declivity of propodeum flat (Fig. 28G)	**20**
17	Gaster with short decumbent to subdecumbent hairs and long subdecumbent to suberect hairs, lateropropodeum almost smooth and shiny (Fig. 28D)	**18**
–	Gaster with short appressed to decumbent hairs and long subdecumbent hairs, lateropropodeum sculptured (Fig. 28F)	**19**
18	Gaster with short subdecumbent to decumbent hairs, and more than ten longer subdecumbent or suberect hairs; distance from propodeal spiracle to declivity less than the diameter of the spiracle (Fig. 28H)	***C. tana***
–	Gaster with abundant subdecumbent hairs, and with fewer than ten longer suberect hairs; distance from propodeal spiracle to declivity same as the diameter of the spiracle (Fig. 28E)	***C. jajoby***
19	Gaster with short decumbent or appressed hairs and longer subdecumbent hairs; distance from propodeal spiracle to declivity same as the diameter of the spiracle (Fig. 28D)	***C. kabosy***
–	Gaster with appressed hairs only; distance from propodeal spiracle to declivity same as half the diameter of the spiracle (Fig. 28F)	***C. berivelo***
20	Posterior margin of head nearly straight (Fig. 29A); gaster with short decumbent hairs and fewer than ten long suberect hairs	***C. raberi***
–	Posterior margin of head weakly concave in the middle (Fig. 29B); gaster with more than ten suberect hairs	**21**
21	Gaster with abundant and long suberect to subdecumbent hairs (Fig. 30A)	***C. hainteny***
–	Gaster with short decumbent hairs and long subdecumbent to suberect hairs (Fig. 30B, C)	**22**
22	Ventral face of petiole medially convex, propodeal spiracle round and small; distance from propodeal spiracle to declivity same as the diameter of the spiracle (Fig. 30B)	***C. vazimba***
–	Ventral face of petiole nearly flat, propodeal spiracle round and larger; distance from propodeal spiracle to declivity less than half the diameter of the spiracle (Fig. 30C)	***C. sampi***

**Figure 20.**
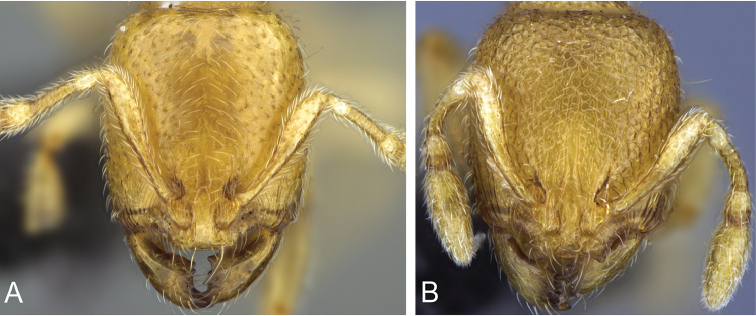
Head in full-face view of **A** *Carebara grandidieri* (CASENT0036011) **B** *Carebara creolei* (CASENT0060069).

**Figure 21.**
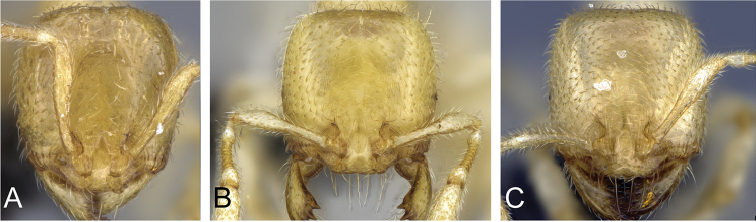
Head in full-face view of **A** *Carebara malagasy* (CASENT0044172) **B** *Carebara hiragasy* (CASENT0496888) **C** *Carebara placida* (CASENT0070817).

**Figure 22.**
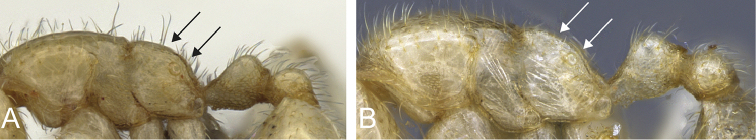
Mesosoma, petiole and postpetiole in profile view of **A** *Carebara hiragasy* (CASENT0496888) **B** *Carebara placida* (CASENT0070817).

**Figure 23.**
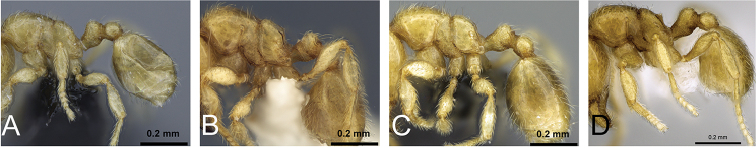
Body in profile view of **A** *Carebara malagasy* (CASENT0044172) **B** *Carebara dota* (CASENT0192570) **C** *Carebara bara* (CASENT0016716) **D** *Carebara mahafaly* (CASENT0410535).

**Figure 24.**
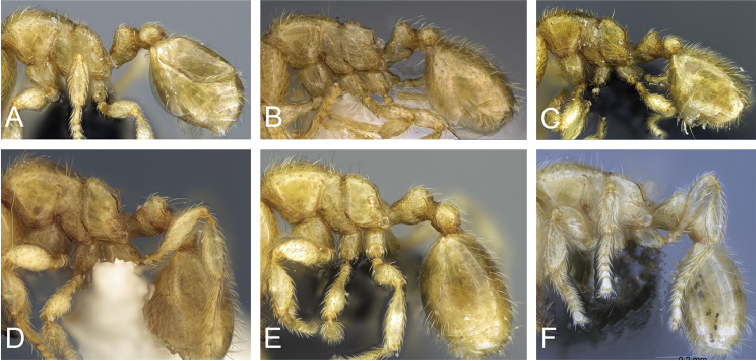
Mesosoma, petiole, postpetiole and gaster in profile view of **A** *Carebara malagasy* (CASENT0044172) **B** *Carebara demeter* (CASENT0127688) **C** *Carebara betsi* (CASENT0479432) **D** *Carebara dota* (CASENT0192570) **E** *Carebara bara* (CASENT0016716) **F** *Carebara tanana* (CASENT0211714).

**Figure 25.**
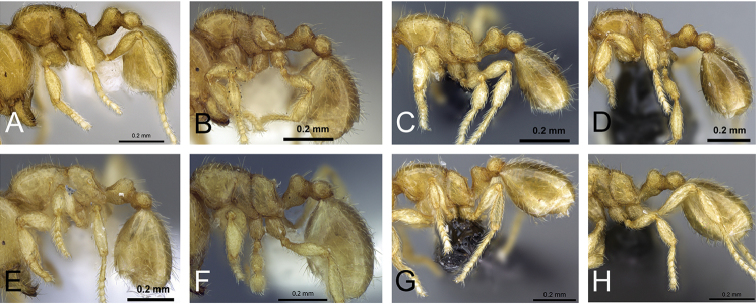
Body in profile view of **A** *Carebara mahafaly* (CASENT0410535) **B** *Carebara salegi* (CASENT0438115) **C** *Carebara lova* (CASENT0468562) **D** *Carebara omasi* (CASENT0028947) **E** *Carebara tana* (CASENT0438084) **F** *Carebara kabosy* (CASENT0437739) **G** *Carebara raberi* (CASENT0140575) **H** *Carebara vazimba* (CASENT0055533).

**Figure 26.**
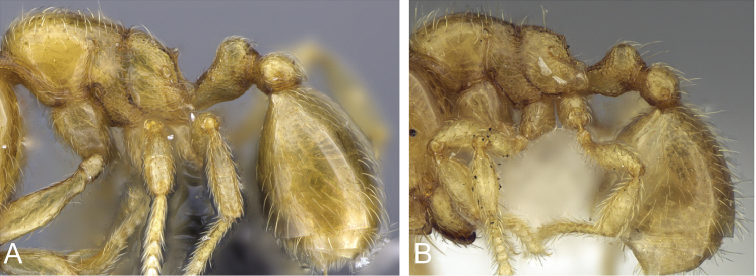
Mesosoma, petiole, postpetiole and gaster in profile view of **A** *Carebara mahafaly* (CASENT0151798) **B** *Carebara salegi* (CASENT0438115).

**Figure 27.**
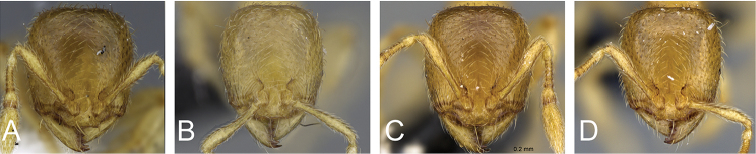
Head in full-face view of **A** *Carebara salegi* (CASENT0438115) **B** *Carebara lova* (CASENT0468562) **C** *Carebara omasi* (CASENT0028947) **D** *Carebara nosindambo* (CASENT0073105).

**Figure 28.**
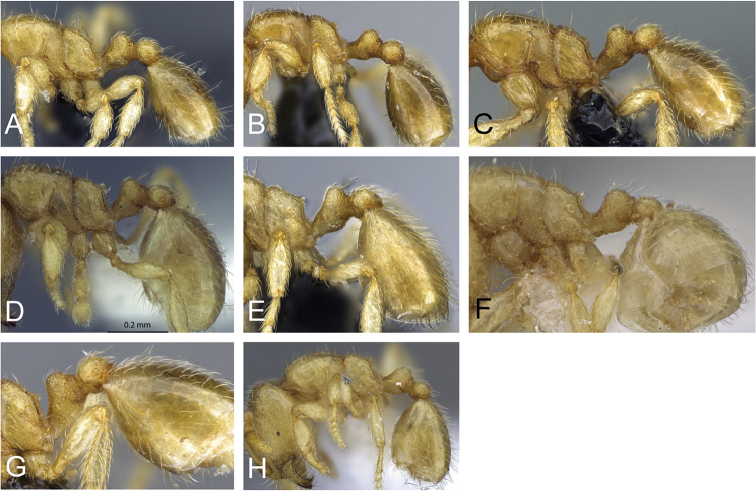
Mesosoma, petiole, postpetiole and gaster in profile view of **A** *Carebara lova* (CASENT0468562) **B** *Carebara omasi* (CASENT0028947) **C** *Carebara nosindambo* (CASENT0073105) **D** *Carebara kabosy* (CASENT0437739) **E** *Carebara jajoby* (CASENT0037501) **F** *Carebara berivelo* (CASENT0439909) **G** *Carebara raberi* (CASENT0140575) **H** *Carebara tana* (CASENT0438084).

**Figure 29.**
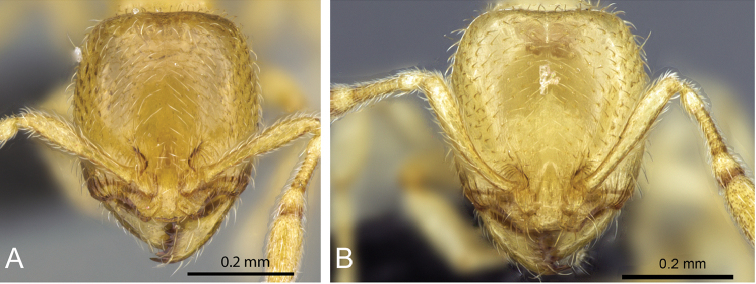
Head in full-face view of **A** *Carebara raberi* (CASENT0140575) **B** *Carebara jajoby* (CASENT0037501).

**Figure 30.**
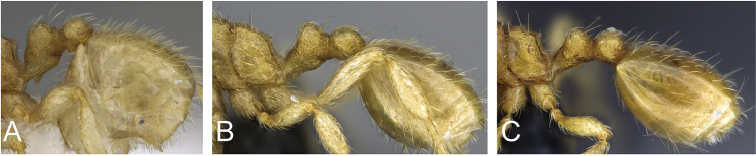
Mesosoma, petiole, postpetiole and gaster in profile view of **A** *Carebara hainteny* (CASENT0410542) **B** *Carebara vazimba* (CASENT0055533) **C** *Carebara sampi* (CASENT0031214).

## Species accounts

### Carebara bara

Taxon classificationAnimaliaHymenopteraFormicidae

Azorsa & Fisher sp. n.

http://zoobank.org/5FC4DF24-163E-4225-8C7B-43FE0AAA8A61

#### Holotype.

(major worker), MADAGASCAR, Mahajanga, Forêt de Tsimembo, 11.0 km 346° NNW Soatana, -18.99528, 44.4435, 50 m, tropical dry forest, 21–25.xi.2001, (*Fisher*, *Griswold et al.*.). Collection code BLF04508, (CASC: CASENT0483010). **Paratypes.** (16 major workers and 22 minor workers), with same data as holotype, 15 major workers (BMNH: CASENT0482934, CASC: CASENT0483093, CASENT0483092, CASENT0483094, CASENT0482845, CASENT0483091, CASENT0483058, CASENT0482822, CASENT0482813, CASENT0482823, CASENT0483057, CASENT0482984, MCZ: CASENT0482830, MHNG: CASENT0483009, NHMB: CASENT0482821), and 22 minor workers (BMNH: CASENT0482990, CASC: CASENT0482991, CASENT0482983, CASENT0483028, CASENT0483023, CASENT0482978, CASENT0482977, CASENT0482994, CASENT0482985, CASENT0482999, CASENT0483033, CASENT0482992, CASENT0482993, CASENT0483011, CASENT0482996, CASENT0482998, CASENT0482987, CASENT0483021, CASENT0483022, MCZ: CASENT0482995, NHMB: CASENT0483037, BMNH: CASENT0482986). 1 major worker, Madagascar, Mahajanga, Forêt de Tsimembo, 8.7 km 336° NNW Soatana, -19.02139, 44.44067, 20 m, tropical dry forest, 21–25.xi.2001, (*Fisher*, *Griswold et al.*) with collection code BLF04511, (CASC: CASENT0077891).

#### Diagnosis.

Antennae ten-segmented. **Major**: Head nearly rectangular, longer than wide, head longitudinally rugose laterally, weakly reticulate near posterior margin of head, frons smooth and shiny, posterior margin of head medially concave, posterolateral corners rounded; posterodorsal corner of propodeum angulate, never with a pair of triangular teeth; dorsum of propodeum nearly flat; combined outline of dorsal surface of peduncle and anterior face of node nearly straight, dorsum rounded; gaster with sparse, long subdecumbent hairs and abundant, short decumbent hairs. **Minor**: Head longer than wide; posterodorsal corners of propodeum each armed with a weakly developed angular tooth, dorsum of propodeum slightly convex and declining posteriorly; combined outline of dorsal surface of peduncle and anterior face of node nearly straight, posterior margin convex, dorsum rounded; gaster covered by abundant decumbent to subdecumbent hairs, and with sparse and slightly longer subdecumbent hairs.

**Figure 31.**
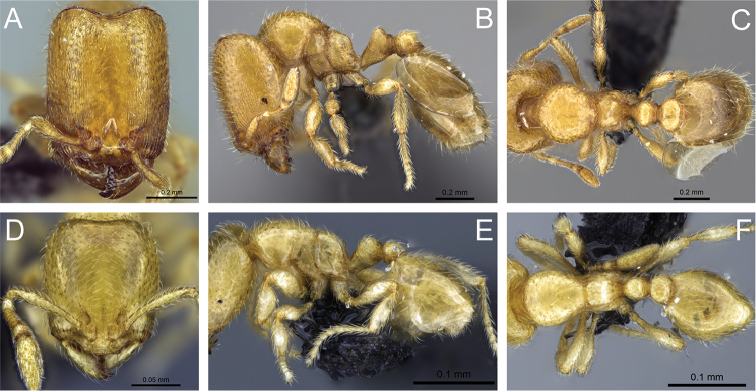
*Carebara bara*–holotype. Major worker, CASENT0483010. **A** head in full-face view **B** body in profile view **C** body in dorsal view. Minor worker, CASENT0482985 **D** head in full-face view **E** body in profile view **F** body in dorsal view.

#### Description of major workers.

**Measurements** (n=25): HL 0.54–0.71 (0.64); HW 0.41–0.56 (0.49); SL 0.21–0.27 (0.25); ML 0.11–0.18 (0.15); EL 0.01–0.03 (0.03); EM 0.15–0.21 (0.18); HD 0.28–0.40 (0.36); WL 0.46–0.65 (0.56); PSL 0.04–0.07 (0.05); PW 0.28–0.40 (0.33); MFL 0.25–0.36 (0.30); MFW 0.07–0.09 (0.08), MTL 0.15–0.29 (0.23); PTL 0.15–0.24 (0.23); PNL 0.08–0.11 (0.11); PTH 0.14–0.20 (0.16); PTW 0.12–0.17 (0.15); PPL 0.11–0.16 (0.13); PPNL 0.09–0.14 (0.12); PPH 0.11–0.17 (0.14); PPW 0.16–0.23 (0.19); GL 0.44–0.77 (0.59); GW 0.34–0.63 (0.40); CI 71–79 (77); MI 20–26 (23); SI 34–43 (39); MLI 57–65 (61); PPLI 57–82 (57); PPI 125–143 (127); PSI 10–14 (10).

Head longer than wide (CI 71–79), in full-face view nearly rectangular, about 1.5 times longer than wide. Posterior margin of head medially concave, posterolateral corners rounded, lateral margins straight to weakly convex. Mandibles with five teeth. Anterior margin of clypeus medially concave and laterally convex. Frons without median ocellus. Antennae with ten segments. Scapes short (HL 0.54–0.71, SL 0.21–0.27, SI 34–43) not surpassing cephalic midlength. Eyes present, consisting of one to three ommatidia (EL 0.01–0.03). Supraclypeal area well defined and triangular.

In profile, posterolateral corner of head with (larger major workers) or without (smaller major workers) a small, obtuse tooth resembling a horn. Promesonotum high and convex, metanotal groove deep. Propodeum about 1.5 times higher than long, dorsal face of propodeum flat or weakly concave, declining posteriorly, propodeum unarmed, posterodorsal corners of propodeum convex or angulate, declivity of propodeum flat to slightly concave in direction of propodeal lobe. Propodeal lobes triangular with blunt apices. Propodeal spiracle rounded and situated above mid-height of sclerite and beyond mid-length of sclerite, by about the diameter of the spiracle; distance from propodeal spiracle to posterodorsal corner of propodeum almost twice the diameter of the spiracle, (PSL 0.04–0.07) and distance to declivity same as the diameter of the spiracle. In dorsal view, promesonotum about as long as wide, anterior margin and sides rounded; sides of propodeum convex.

Petiole approximately as high as long (PTH 0.14–0.20, PTL 0.15–0.24) and with a short peduncle, ventral face medially convex. Anterodorsal and posterodorsal faces of petiolar node sloping, combined outline of dorsal surface of peduncle and anterior face of node flat, posterior face of node vertical, straight to slightly convex, dorsum rounded. Subpetiolar process slightly shorter than the diameter of the propodeal spiracle. Postpetiolar node rounded and lower than petiolar node. In dorsal view, postpetiolar node wider than petiolar node (PPW 0.16–0.23, PTW 0.12–0.17), petiolar node wider than long (PTW 0.12–0.17, PNL 0.08–0.11), anterior and posterior margins of petiole and postpetiole nearly straight, sides rounded in petiole and postpetiole.

Dorsal surface of mandibles, clypeus and frons smooth and shiny, with scattered piligerous punctae on head and mandibles. Dorsolateral faces of head longitudinally rugose, gena with well-developed longitudinal reticulations, weakly marked rugae on frontal lobes, reticulate-rugose sculpture near posterior margin of head. In profile, posterolateral portion of cephalic dorsum smooth and shiny. Mesosoma smooth and shiny, except for katepisternum and propodeal lobe (areolate-rugose), and metapleuron (areolate and with longitudinal rugae). Petiole and ventral face of postpetiole areolate-rugose. In dorsal view, mesosoma, petiole, postpetiole and gaster smooth and shiny.

Lateral margins, and posterolateral corners of head with short subdecumbent hairs, and longer suberect hairs. Scapes with appressed hairs. Outer margin of mandibles with short subdecumbent hairs. Mesosoma with short and long suberect hairs. Petiole, postpetiole and gaster with short decumbent hairs and sparse and slightly longer subdecumbent hairs. Tibia with short appressed hairs. Color yellowish ferruginous, antennae, legs and parts of gaster, slightly lighter.

#### Description of minor workers.

**Measurements** (n=9): HL 0.33–0.37; HW 0.28–0.34; SL 0.20–0.23; ML 0.08–0.10; EL 0.01–0.02; EM 0.09–0.10; HD 0.19–0.22; WL 0.33–0.38; PSL 0.02–0.04; PW 0.16–0.20; MFL 0.20–0.22; MFW 0.05; MTL 0.15–0.17; PTL 0.11–0.12; PNL 0.06–0.07; PTH 0.09–0.10; PTW 0.08–0.09; PPL 0.07–0.08; PPNL 0.06–0.08; PPH 0.07–0.08; PPW 0.09–0.12; GL 0.26–0.34; GW 0.20–0.25; CI 82–92; MI 24–27; SI 59–64; MLI 65–76; PPLI 58–67; PPI 113–138; PSI 7–14.

Head longer than wide (CI 82–92), in full-face view weakly subrectangular, about 1.2 times longer than wide, and slightly narrowed anteriorly. Posterior margin of head slightly concave in the middle, posterolateral corners rounded, lateral margins slightly convex, nearly straight. Mandibles with five teeth. Anterior margin of clypeus more or less straight, and laterally angulate. Antennae with ten segments. Scape fails to reach the posterior margin of head (HL 0.33–0.37, SL 0.20–0.23, SI 59–64). Eyes present, consisting of one ommatidium (EL 0.01–0.02). Supraclypeal area triangular, in some specimens poorly defined, and appears as a short longitudinal depression.

In profile view, promesonotum weakly convex, metanotal groove slightly impressed. Propodeum about 1.5 times higher than long, dorsal face convex and declining posteriorly, posterodorsal corners of propodeum each armed with a small angulate tooth, declivity of propodeum slightly concave. Propodeal lobes triangular. Propodeal spiracle rounded and situated slightly above mid-height of sclerite by about half the diameter of the spiracle, and beyond mid-length of the sclerite by about the diameter of the spiracle; distance from propodeal spiracle to posterodorsal corner of propodeum same as the diameter of the spiracle (PSL 0.02–0.04), and distance to declivity less than the diameter of the spiracle. In dorsal view, promesonotum longer than wide, anterior margin rounded, sides convex; sides of propodeum weakly convex.

Petiole slightly longer than high (PTL 0.11–0.12, PTH 0.09–0.10) with a short peduncle, ventral face weakly convex in the middle. Combined outline of dorsal surface of peduncle and anterior face of node nearly straight, posterior margin slightly convex, dorsum rounded. Subpetiolar process small, almost the same as the diameter of the propodeal spiracle. Postpetiolar node convex and lower than petiolar node. In dorsal view, postpetiolar node slightly wider than petiolar node (PPW 0.09–0.12, PTW 0.08–0.0.9), petiolar node wider than long (PTW 0.09–0.12, PNL 0.06–0.07), anterior margin of petiole convex, posterior margin slightly concave, anterior and posterior margin of postpetiole nearly straight, sides moderately convex in petiole and postpetiole.

Dorsal surface of mandibles, clypeus and frons smooth and shiny, with abundant piligerous punctae on mandibles and head. Head with fine and short longitudinal rugae on malar space and frontal lobes. Mesosoma smooth and shiny, except for katepisternum, below mid-height of metapleuron, and propodeal lobe, which are areolate-rugose. Sides of petiole, and ventral face of postpetiole areolate-rugose. In dorsal view, mesosoma, petiole, postpetiole and gaster smooth and shiny.

Lateral margins, scapes and posterior margin of head with subdecumbent hairs. Hairs in the posterior margin shorter than others. Outer margin of mandible with decumbent hairs. Mesosoma with suberect hairs. Petiole, postpetiole, tibia and gaster with short decumbent to subdecumbent hairs, and with sparse and slightly longer subdecumbent hairs. Tibia with abundant to sparse decumbent hairs. Color yellowish ferruginous.

#### Distribution and biology.

*Carebara bara* is an endemic and widespread species, found across Madagascar (Figure 68), mainly in the tropical dry forest in western Madagascar and the desert spiny bush thicket in the southwest. In the center of Madagascar (montane forest) there is only one record. *C. bara* occurs in the dry forest, gallery forest, littoral rainforest, montane rainforest, spiny forest/dry forest transition, spiny forest/thicket, spiny thicket, spiny thicket gallery forest transition, and tropical dry forest, and has been collected at elevations ranging from 10–1410 m. Individuals and nest series were collected from rotten logs, in the leaf litter, in leaf mold, and rotten wood.

#### Comments.

*Carebara bara* can be confused with *C. berivelo* but can be separated by the form of the propodeum. *C. berivelo* is armed with a pair of small triangular teeth while *C. bara* is unarmed in major workers and armed with a small angulate tooth in minor workers. This species can also be confused with *C. tana*, but in *C. tana* the combined outline of dorsal surface of peduncle and anterior face of node is medially concave. *C. bara* is widespread throughout most of Madagascar, with only one record in the center of the island and no records in the east, while *C. berivelo* and *C. tana* are restricted to the north of Madagascar. These three species occur in sympatry in the north of Madagascar, other four species were recorded at the same localities: *C. grandidieri*, *C. kabosy*, *C. malagasy*, and *C. salegi*, which can be separated from *C. bara* by the characters described above.

**Figure 32.**
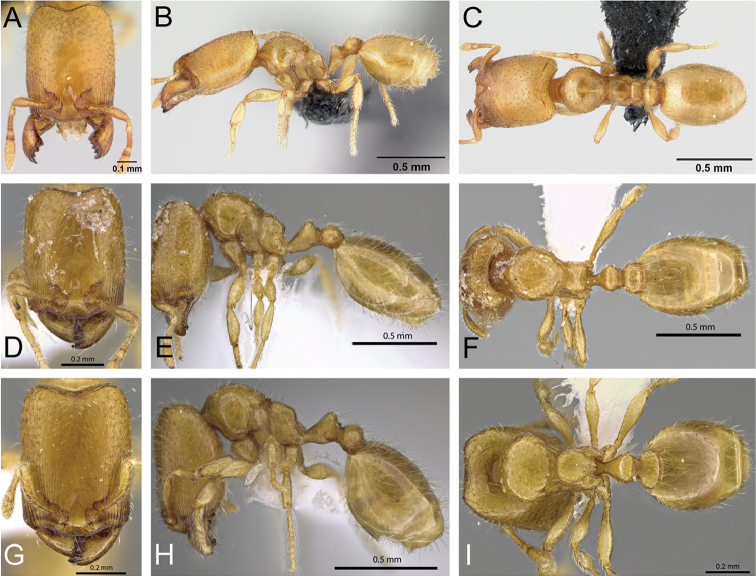
Intermediates of *Carebara bara*. Major workers, CASENT0482934. **A** head in full-face view **B** body in profile view **C** body in dorsal view. CASENT0127693 **D** head in full-face view **E** body in profile view **F** body in dorsal view. CASENT0439907 **G** head in full-face view **H** body in profile view **I** body in dorsal view.

*C. bara* have three intermediates in the major worker subcaste (Figure 32) with only minor variations between them. The shape of the posterolateral corner of the head and the posterior margin of the head do not vary much. Intermediate 3 has an increase in the size of the eyes, but only one ommatidium. Ocelli are absent in intermediates 1 and 2, and sometimes present in intermediate 3 (one ocellus). Intermediate 3 has reduced flight sclerites. In addition, the dorsum of the mesosoma of intermediate 3 is anteriorly convex and gradually slopes to a declivity. By comparison, in intermediates 1 and 2 the dorsum of the mesosoma is anteriorly convex, and the propodeum is below the promesonotum, which is flat and declining posteriorly to a declivity. Pilosity is nearly identical in all intermediates. The longitudinal rugae of the head are more finely marked in intermediate 1. The sculpture of the mesosoma, petiole and postpetiole is nearly identical in all the intermediates.

#### Additional material examined.

**MADAGASCAR**: ***Antananarivo***: Réserve Spéciale d’Ambohitantely, Forêt d’ Ambohitantely, 20.9 km 72° NE d’ Ankazobe, -18.22528, 47.28683, 1410 m, montane rainforest, 17–22.iv.2001, (*Fisher, Griswold et al.*); ***Antsiranana***: Réserve Spéciale d’Ambre, 3.5 km 235° SW Sakaramy, -12.46889, 49.24217, 325 m, tropical dry forest, 26–31.i.2001, (*Fisher*, *Griswold et al.*); Antsiranana, Réserve Spéciale de l’Ankarana, 13.6 km 192° SSW Anivorano Nord, -12.86361, 49.22583, 210 m, tropical dry forest, 16–21.ii.2001, (*Fisher, Griswold et al.*); ***Fianarantsoa***: Parc National d’Isalo, Sahanafa River, 29.2 km 351° N Ranohira, -22.31333, 45.29167, 500 m, gallery forest, 10–13.ii.2003, (*Fisher, Griswold et al.*); Fianarantsoa, Forêt d’Analalava, 29.6 km 280° W Ranohira, -22.59167, 45.12833, 700 m, tropical dry forest, 1–5.ii.2003, (*Fisher, Griswold et al.*); Fianarantsoa, Parc National d’Isalo, 9.1 km 354° N Ranohira, -22.48167, 45.46167, 725 m, gallery forest, 27–31.i.2003, (*Fisher, Griswold et al.*); ***Mahajanga***: Réserve Spéciale de Bemarivo, 23.8 km 223° SW Besalampy, -16.925, 44.36833, 30 m, tropical dry forest, 19–23.xi.2002, (*Fisher, Griswold et al.*); Mahajanga, Parc National de Baie de Baly, 12.4 km 337° NNW Soalala, -16.01, 45.265, 10 m, tropical dry forest, 26–30.xi.2002, (*Fisher, Griswold et al.*); Mahajanga, Forêt Ambohimanga, 26.1 km 314° Mampikony, -15.96267, 47.43817, 250 m, tropical dry forest, 13.xii.2004, (*B.L. Fisher*); Mahajanga, Forêt de Tsimembo, 8.7 km 336° NNW Soatana, -19.02139, 44.44067, 20 m, tropical dry forest, 21–25.xi.2001, (*Fisher, Griswold et al.*); Mahajanga, Réserve forestière Beanka, 50.2 km E Maintirano, -18.02649, 44.05051, 250 m, tropical dry forest on tsingy, 19–22.x.2009, (*B.L. Fisher et al*.); Mahajanga, Parc National d’Ankarafantsika, Forêt de Tsimaloto, 18.3 km 46° NE de Tsaramandroso, -16.22806, 47.14361, 135 m, tropical dry forest, 2–8.iv.2001, (*Fisher, Griswold et al.*); Mahajanga, Parc National tsingy de Bemaraha, 3.4 km 93° E Bekopaka, Tombeau Vazimba, -19.14194, 44.828, 50 m, tropical dry forest, 6–10.xi.2001, (*Fisher, Griswold et al.*); Mahajanga, Parc National tsingy de Bemaraha, 2.5 km 62° ENE Bekopaka, Ankidrodroa River, -19.13222, 44.81467, 100 m, tropical dry forest on tsingy, 11–15.xi.2001, (*Fisher, Griswold et al.*); Mahajanga, Parc National d’Ankarafantsika, Ampijoroa Station Forestière, 5.4 km 331° NW Andranofasika, -16.29889, 46.813, 70 m, tropical dry forest, 26.iii.-1.iv.2001, (*Fisher, Griswold et al.*); Mahajanga, Parc National d’Ankarafantsika, Ampijoroa Station Forestière, 40 km 306° NW Andranofasika, -16.32083, 46.81067, 130 m, tropical dry forest, 26.iii.-1.iv.2001, (*Fisher, Griswold et al.*); Mahajanga, Réserve d’Ankoririka, 10.6 km 13° NE de Tsaramandroso, -16.26722, 47.04861, 210 m, tropical dry forest, 9–14.iv.2001, (*Fisher, Griswold et al.*); Mahajanga, Parc National tsingy de Bemaraha, 10.6 km ESE 123° Antsalova, -18.70944, 44.71817, 150 m, tropical dry forest on tsingy, 16–20.xi.2001, (*Fisher, Griswold et al.*); Mahajanga, Parc National de Namoroka, 17.8 km 329° WNW Vilanandro, -16.37667, 45.32667, 100 m, tropical dry forest, 8–12.xi.2002, (*Fisher, Griswold et al.*); ***Toliara***: Forêt de Beroboka, 5.9 km 131° SE Ankidranoka, -22.23306, 43.36633, 80 m, tropical dry forest, 12–16.iii.2003, (*Fisher, Griswold et al.*); Toliara, Parc National de Kirindy Mite, 16.3 km 127° SE Belo sur Mer, -20.79528, 44.147 80 m, tropical dry forest, 6–10.xii.2001, (*Fisher, Griswold et al.*); Toliara, Réserve Privé Berenty, Forêt de Malaza, Mandraré River, 8.6 km 314° NW Amboasary, -25.00778, 46.306, 40 m, gallery forest, 6.ii.2002, (*Fisher, Griswold et al.*); Toliara, Parc National d’Andohahela, Forêt d’Ambohibory, 1.7 km 61° ENE Tsimelahy, 36.1 km 308° NW Tolagnaro, -24.93, 46.6455, 300 m, tropical dry forest, 16–20.i.2002, (*Fisher, Griswold et al.*); Toliara, Forêt de Mite, 20.7 km 29° WNW Tongobory, -23.52417, 44.12133, 75 m, gallery forest, 27.ii.-3.iii.2002, (*Fisher, Griswold et al.*); Toliara, Réserve Privé Berenty, Forêt de Bealoka, Mandraré River, 14.6 km 329° NNW Amboasary, -24.95694, 46.2715, 35 m, gallery forest, 3–8.ii.2002, (*Fisher, Griswold et al.*); Toliara, Réserve Spéciale de Cap Sainte Marie, 12.3 km 262° W Marovato, -25.58167, 45.16833, 200 m, spiny forest/thicket, 11–15.ii.2002, (*Fisher, Griswold et al.*); Toliara, Parc National d’Andohahela, Forêt de Manantalinjo, 33.6 km 63° ENE Amboasary, 7.6 km 99° E Hazofotsy, -24.81694, 46.61, 150 m, spiny forest/thicket, 12–16.i.2002, (*Fisher, Griswold et al.*); Toliara, Parc National de Zombitse, 19.8 km 84° E Sakaraha, -22.84333, 44.71, 770 m, tropical dry forest, 5–9.ii.2003, (*Fisher, Griswold et al.*); Toliara, Antafoky, -23.47917, 44.06611, 60 m, gallery forest, 26.i.2002, (*Frontier Project*); Toliara, Fiherenana, -23.17694, 43.96083, 100 m, gallery forest, 21–24.x.2002, (*Frontier Project*); Toliara, Ranobe, -23.0342, 43.61185, 30 m, spiny forest/thicket, 5–9.ii.2003, (*Frontier Project*); Toliara, Manderano, -23.52333, 44.09444, 80 m, spiny thicket, 8.vi.2002, (*Frontier Project*); Toliara, Sept Lacs, -23.52472, 44.15917, 160 m, spiny thicket gallery forest transition, 10.iii.2002, (*Frontier Project*); Toliara, Ranobe, -23.04067, 43.60973, 20 m, gallery forest, degraded, 17–21.v.2003, (*Frontier Wilderness Project*); Toliara, Sept, Lacs, -23.52833, 44.15556, 80 m, gallery forest, 8.iii.2002, (*Frontier Project*); Toliara, Manderano, -23.52722, 44.0875, 70 m, gallery forest, 10.v.2002, (*Frontier Project*); Toliara, Manombo, -22.8123, 43.73932, 165 m, gallery forest, TS3, 22–24.v.2004, (*Frontier Wilderness Project*); Toliara, Forêt de Kirindy, 15.5 km 64° ENE Marofandilia, -20.045, 44.66222, 100 m, tropical dry forest, 28.xi.-3.xii.2001, *(Fisher, Griswold et al.*); Toliara, Beza-Mahafaly, 27 km E Betioky, -23.65, 44.63333, 135 m, tropical dry forest, 24.iv.1997, (*B.L. Fisher*); Toliara, Beza-Mahafaly, 27 km E Betioky, -23.65, 44.63333, 135 m, tropical dry forest, 23.iv.1997, (*B.L. Fisher*); Toliara, southern Isoky-Vohimena Forest, 59 km NE Sakaraha, -22.46667, 44.85, 730 m, tropical dry forest, 21.i.1996, (*B.L. Fisher*); Toliara, Forêt Vohidava 88.9 km N Amboasary, -24.24067, 46.28783, 500 m, spiny forest/dry forest transition, 6–8.xii.2006, (*B.L. Fisher et al*.); Toliara, Forêt de Petriky, 12.5 km W 272° Tolagnaro, -25.06167, 46.87, 10 m, littoral rainforest, 22.xi.1998, (*B.L. Fisher*); Toliara, Makay Mts., -21.21836, 45.3106, 510 m, gallery forest on sandy soil, 24–27.xi.2010, (*B.L. Fisher et al*.); Toliara, Makay Mts., -21.20978, 45.34184, 525 m, gallery forest on sandy soil, 27.xi-2.xii.2010, (*B.L. Fisher et al*.); Toliara, Mandena, 8.4 km NNE 30° Tolagnaro, -24.95167, 47.00167, 20 m, littoral rainforest, 20.xi.1998, (*B.L. Fisher*); Toliara, Forêt de Mahavelo, Isantoria River, 5.5 km 37° NE Ifotaka, -24.75361, 46.1515, 115 m, spiny forest/thicket, 31.i.2002, (*Fisher, Griswold et al.*); Toliara, Parc National d’Andohahela, Forêt d’Ambohibory, 1.7 km 61° ENE Tsimelahy, 36.1 km 308° NW Tolagnaro, -24.93, 46.6455, 300 m, tropical dry forest, 16–21.i.2002, (*Fisher*, *Griswold et al.*); Toliara, Fiherenana, -23.17698, 43.96142, gallery forest, 18–19.viii.2003, (*Frontier Wilderness Project*); Toliara, Makay Mts., -21.30997, 45.12946, 590 m, dry forest on sandy soil, 3–6.xii.2010, (*B.L. Fisher*).

### Carebara berivelo

Taxon classificationAnimaliaHymenopteraFormicidae

Azorsa & Fisher sp. n.

http://zoobank.org/AC17530D-9B3B-47DE-9D70-1F92769ACB7E

#### Holotype.

(major worker), MADAGASCAR, Antsiranana, Réserve Spéciale d’Ambre, 3.5 km 235° SW Sakaramy, -12.46889, 49.24217, 325 m, tropical dry forest, 26–31.i.2001, (*Fisher, Griswold et al.*). Collection code BLF02654, (CASC: CASENT0438188). **Paratypes**: (10 major workers and 20 minor workers), with same data as holotype, 6 major workers (BMNH: CASENT0438182, CASC: CASENT0438184, CASENT0438198, CASENT0439906, CASENT0438197, MCZ: CASENT0438281), and 18 minor workers (BMNH: CASENT0438185, CASC: CASENT0438222, CASENT0438221, CASENT0438133, CASENT0438209, CASENT0438219, CASENT0438131, CASENT0439901, CASENT0438192, CASENT0438220, CASENT0438194, CASENT0438193, CASENT0438206, CASENT0438190, CASENT0438204, MCZ: CASENT0438196, MHNG: CASENT0438187, NHMB: CASENT0439903). 3 major workers and 2 minor workers with same data as holotype except collected from pitfall trap and collection code BLF2655, 3 major workers (CASC: CASENT0439911, MHNG: CASENT0439915, NHMB: CASENT0439914), 2 minor workers (CASC: CASENT0439917, CASENT0439916). 1 major worker with different data from holotype: Madagascar, Antsiranana, Montagne des Français, 7.2 km 142° SE Antsiranana, (=Diego Suarez), -12.32278, 49.33817, 180 m, tropical dry forest, 22–28.ii.2001, (*Fisher*, *Griswold et al.*). Collection code BLF03128, (CASC: CASENT0460943).

#### Diagnosis.

Antennae ten-segmented. **Major**: Head subrectangular, posterior margin medially concave, and posterolateral corners rounded; promesonotum flat in profile view, quite similar to *C. bara*, but the propodeum of *C. berivelo* has a pair of acute teeth, dorsum of propodeum is flat; combined outline of dorsal surface of peduncle and anterior face of node slightly medially concave, anterodorsal corner convex, posterior face of node vertical and nearly straight, dorsum slightly convex. **Minor**: Head slightly longer than wide, posterior margin nearly straight; promesonotum weakly convex, similar to *C. bara* but easily differentiated by the presence of a pair of small acute teeth on the propodeum, anterodorsal corner moderately convex, dorsum flat and declining posteriorly; gaster with decumbent hairs.

**Figure 33.**
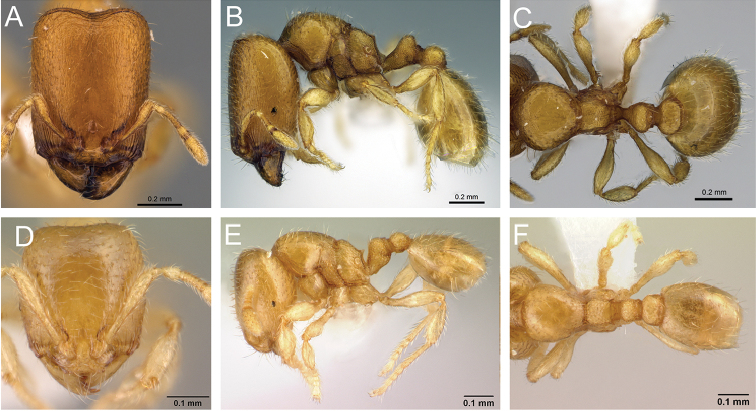
*Carebara berivelo*–holotype. Major worker, CASENT0438188. **A** head in full-face view **B** body in profile view **C** body in dorsal view. Minor worker, CASEN0438184 **D** head in full-face view **E** body in profile view **F** body in dorsal view.

#### Description of major workers.

**Measurements** (n=11): HL 0.56–0.71 (0.71); HW 0.42–0.54 (0.53); SL 0.24–0.27 (0.27); ML 0.10–0.15 (0.15); EL 0.02–0.04 (0.04); EM 0.15–0.20 (0.20); HD 0.31–0.39 (0.39); WL 0.47–0.60 (0.60); PSL 0.06–0.09 (0.08); PW 0.28–0.37 (0.37); MFL 0.31–0.37 (0.35); MFW 0.07–0.10 (0.09); MTL 0.22–0.28 (0.28); PTL 0.16–0.25 (0.25); PNL 0.07–0.11 (0.11); PTH 0.13–0.17 (0.17); PTW 0.12–0.17 (0.17); PPL 0.11–0.14 (0.14); PPNL 0.10–0.13 (0.12); PPH 0.11–0.16 (0.16); PPW 0.15–0.20 (0.20); GL 0.46–0.86 (0.57); GW 0.40–0.55 (0.54), CI 73–77 (75); MI 16–21 (21); SI 37–43 (38); MLI 65–76 (66); PPLI 56–72 (56); PPI 118–143 (118); PSI 13–18 (15).

Head longer than wide (CI 73–77), in full-face view nearly subrectangular, about 1.3 times longer than wide. Posterior margin of head medially concave, posterolateral corners rounded, lateral margins nearly straight. Mandibles with five teeth. Anterior margin of clypeus slightly concave medially and laterally convex. Antennae with ten segments. Scapes short (HL 0.56–0.71, SL 0.24–0.27, SI 37–43) not surpassing cephalic midlength. Eyes present, consisting of three ommatidia (EL 0.02–0.04). Supraclypeal area as a triangular depression but poorly defined.

In profile, posterolateral corner of head with (larger major workers) or without (smaller major workers) a small, obtuse tooth resembling a horn. Promesonotum high and convex, metanotal groove present. Propodeum about 1.4 times higher than long, dorsal face of propodeum flat, declining posteriorly, posterodorsal corner of propodeum with a pair of short, triangular teeth, declivity of propodeum flat to slightly concave with thin lateral laminae connecting propodeal lobe. Propodeal lobes triangular with blunt apices. Propodeal spiracle rounded and situated slightly above mid-height of sclerite, and beyond mid-length of sclerite, by about half the diameter of the spiracle, distance from propodeal spiracle to posterodorsal corner of propodeum more than twice but less than three times the diameter of the spiracle (PSL 0.06–0.09), and distance to declivity same as the diameter of the spiracle. In dorsal view, promesonotum about as long as wide, anterior margin and sides rounded; sides of propodeum nearly straight.

Petiole longer than high (PTH 0.13–0.17, PTL 0.16–0.25) with a relatively short peduncle, ventral face nearly flat but slightly convex in the middle. Combined outline of dorsal surface of peduncle and anterior face of node medially concave, posterior margin nearly vertical and slightly convex, anterodorsal corner convex, and posterodorsal corner rounded, dorsum rounded. Subpetiolar process small, acutely produced, and at about the same as the diameter of the propodeal spiracle. Postpetiolar node rounded and lower than petiolar node. In dorsal view, postpetiolar node slightly wider than petiolar node (PPW 0.16–0.23, PTW 0.12–0.17), petiolar node wider than long (PTW 0.12–0.17, PNL 0.07–0.11), anterior and posterior margins of petiole nearly straight, anterior margin of postpetiole concave and posterior margin straight, sides rounded in petiole and postpetiole.

Dorsal surface of mandibles, clypeus and frons smooth and shiny, with scattered piligerous punctae on head and mandibles. Dorsolateral faces of head with fine longitudinal rugae, reticulate-rugose sculpture near posterior margin of head, gena with longitudinal carinae, weakly marked rugae on frontal lobes. In profile, posterolateral portion of cephalic dorsum smooth and shiny. Sides of pronotum with fine longitudinal and parallel striations, smooth and shiny medially and dorsally, anepisternum smooth and shiny, katepisternum, metapleuron, propodeum, propodeal lobes, petiole and ventral face of postpetiole areolate-rugose. In dorsal view, promesonotum smooth and shiny except for anterior margin which is weakly areolate, propodeum, declivity and petiole areolate-rugose, postpetiole and gaster smooth and shiny.

Lateral margins and posterior margin of head with suberect hairs, and sparse short decumbent hairs. Scapes with appressed hairs. Outer margin of mandible with sparse decumbent hairs. Mesosoma with suberect hairs. Petiole, postpetiole and gaster with short decumbent hairs and sparse long suberect hairs. Tibia with sparse appressed hairs. Color yellowish ferruginous, antennae, legs and parts of gaster, slightly lighter.

#### Description of minor workers.

**Measurements** (n=5): HL 0.35–0.36; HW 0.30–0.33; SL 0.20–0.23; ML 0.08–0.10; EL 0.01–0.02; EM 0.10–0.11; HD 0.20–0.23; WL 0.33–0.38; PSL 0.04–0.05; PW 0.19–0.21; MFL 0.20–0.24, MFW 0.05–0.06; MTL 0.16–0.18; PTL 0.11–0.13; PNL 0.06–0.07; PTH 0.09–0.10; PTW 0.08; PPL 0.07–0.08; PPNL 0.06–0.07; PPH 0.07–0.08; PPW 0.09–0.11; GL 0.27–0.36; GW 0.21–0.27; CI 84–92; MI 23–28; SI 57–64; MLI 67–73; PPLI 58–64; PPI 113–138; PSI 12–15.

Head longer than wide (CI 84–92), in full-face view weakly subrectangular, about 1.2 times longer than wide, and slightly narrowed anteriorly. Posterior margin of head slightly concave in the middle, posterolateral corners convex, lateral margins convex. Mandibles with five teeth. Anterior margin of clypeus more or less straight, sides with small, angulate, forwardly directed teeth. Antennae with ten segments. Scape fails to reach the posterior margin of head (HL 0.35–0.36, SL 0.20–0.23, SI 57–64). Eyes present, consisting of one ommatidium (EL 0.01–0.02). Supraclypeal area triangular but poorly defined and appears as a small oblong depression.

In profile view, promesonotum weakly convex, metanotal groove deeply impressed. Propodeum about 1.45 times higher than long, dorsal face nearly flat and declining posteriorly, anterodorsal corner convex, posterodorsal corners each armed with a triangular tooth, declivity of propodeum concave and with thin lateral laminae. Propodeal lobes triangular. Propodeal spiracle rounded and situated slightly above mid-height of sclerite by about half the diameter of the spiracle, and beyond mid-length of sclerite by about 1.5 times the diameter of the spiracle, distance from propodeal spiracle to posterodorsal corner of propodeum barely larger than the diameter of the spiracle (PSL 0.04–0.05), and distance to declivity less than half the diameter of the spiracle. In dorsal view, promesonotum longer than wide, anterior margin weakly convex, sides convex and narrowed posteriorly; propodeum about as long as wide, sides convex.

Petiole approximately as high as long (PTH 0.09–0.10, PTL 0.11–0.13) with a short peduncle, ventral face convex in the middle. Combined outline of dorsal surface of peduncle and anterior face of node nearly straight, sometimes weakly concave, posterior margin slightly convex, dorsum weakly convex. Subpetiolar process reduced to a small convexity. Postpetiolar node strongly convex and slightly lower than petiolar node, postpetiolar ventral process present, small and convex. In dorsal view, postpetiolar node wider than petiolar node (PPW 0.09–0.11, PTW 0.08), petiolar node wider than long (PTW 0.08, PNL 0.06–0.07), anterior and posterior margins of petiole nearly straight, anterior margin of postpetiole concave and posterior margin nearly straight, sides of petiole slightly convex and sides of postpetiole strongly convex.

Dorsal surface of mandibles, clypeus and frons smooth and shiny, with scattered piligerous punctae on head and mandibles. Dorsolateral faces of head, gena and frontal lobes with fine longitudinal rugae. Mesosoma smooth and shiny, except for katepisternum and posterior border of anepisternum (areolate), metapleuron (finely areolate), and dorsum of propodeum (finely areolate). Petiole and ventral face of postpetiole areolate. In dorsal view, promesonotum postpetiole and gaster smoth and shiny, propodeum and petiole areolate.

Lateral margins and posterior margin of head with subdecumbent hairs. Scapes with abundant decumbent hairs. Outer margin of mandible with decumbent and appressed hairs. Mesosoma with suberect hairs. Petiole, postpetiole, and gaster with subdecumbent to decumbent hairs, and some longer and subdecumbent hairs. Tibia with appressed hairs. Color yellowish ferruginous.

#### Distribution and biology.

*Carebara berivelo* is known only from the north of Madagascar and was found in littoral rainforest and tropical dry forest (Figure 68). The specimens were found at three elevations: 90 m, 180 m, and 325 m. *C. berivelo* was collected using maxi-Winkler and pitfall traps. Individuals and colonies were found in sifted litter, leaf mold, and rotten wood.

#### Comments.

*Carebara berivelo* is endemic to the north of Madagascar. It can be confused with *C. bara* but can be separated from that species by the propodeum, which is armed with a pair of small triangular teeth, while *C. bara* is unarmed in major workers, and armed with a small angulate tooth in minor workers. Both species occur in sympatry in the north of Madagascar. Five additional species are present in this area (*C. grandidieri*, *C. kabosy*, *C. malagasy*, *C. salegi*, and *C. tana*).

*C. berivelo* does not present intermediates in the major worker subcaste.

#### Additional material examined.

**MADAGASCAR**: ***Antsiranana***: Forêt d’Orangea, 3.6 km 128° SE Remena, -12.25889, 49.37467, 90 m, littoral rainforest, 22–28.ii.2001, (*Fisher, Griswold et al.*); Antsiranana, Montagne des Français, 7.2 km 142° SE Antsiranana (=Diego Suarez), -12.32278, 49.33817, 180 m, tropical dry forest, 22–28.ii.2001, (*Fisher, Griswold et al.*).

### Carebara betsi

Taxon classificationAnimaliaHymenopteraFormicidae

Azorsa & Fisher sp. n.

http://zoobank.org/71F716F5-EAD5-40AF-AEA1-1E6200EBA28C

#### Holotype.

(major worker), MADAGASCAR, Antananarivo, Réserve Spéciale d’Ambohitantely, Forêt d’ Ambohitantely, 20.9 km 72° NE d’ Ankazobe, -18.22528, 47.28683, 1410 m, montane rainforest, 17–22.iv.2001, (*Fisher, Griswold et al.*). Collection code BLF03694, (CASC: CASENT0479444). **Paratypes**: (5 major workers and 26 minor workers), with same data as holotype, 5 major workers, (BMNH: CASENT0479447, CASC: CASENT0479429, CASENT0479534, CASENT0479472, MCZ: CASENT0479509), and 26 minor workers (BMNH: CASENT0007713, CASC: CASENT0479452, CASENT0474433, CASENT0479555, CASENT0479492, CASENT0479495, CASENT0479538, CASENT0479493, CASENT0479430, CASENT0479432, CASENT0479476, CASENT0479539, CASENT0479552, CASENT0479561, CASENT0479431, CASENT0479518, CASENT0479516, CASENT0479498, CASENT0479510, CASENT0479497, CASENT0479512, CASENT0479505, CASENT0479496, MCZ: CASENT0479519, MHNG: CASENT0479520, NHMB: CASENT0479521).

#### Diagnosis.

Antennae ten-segmented. **Major**: Head subrectangular, longer than wide, lateral margins straight and parallel, dorsal face of head longitudinally rugose-rugulose, rugae extend from frontal lobes and posterior margin of supraclypeal area to the posterior margin of the head; combined outline of dorsal surface of peduncle and anterior face of node flat, posterior face node vertical and nearly straight; dorsum of propodeum flat and declining posteriorly; gaster with abundant subdecumbent hairs. **Minor**: Head longer than wide, weakly subrectangular, posterior margin of head medially concave; combined outline of dorsal surface of peduncle and anterior face of node straight, posterior face vertical and slightly convex; dorsum of propodeum flat and declining posteriorly; gaster with abundant subdecumbent hairs.

**Figure 34.**
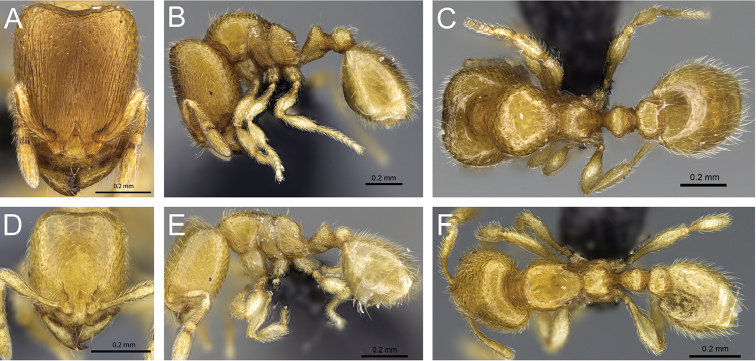
*Carebara betsi*–holotype. Major worker, CASENT0479444. **A** head in full-face view **B** body in profile view **C** body in dorsal view. Minor worker, CASENT0479432 **D** head in full-face view **E** body in profile view **F** body in dorsal view.

#### Description of major workers.

**Measurements** (n=7): HL 0.51–0.63 (0.58); HW 0.40–0.50 (0.45); SL 0.20–0.27 (0.24); ML 0.09–0.13 (0.10); EL 0.02; EM 0.14–0.18 (0.17); HD 0.28–0.38 (0.28); WL 0.44–0.57 (0.52); PSL 0.05–0.08 (0.07); PW 0.27–0.33 (0.33); MFL 0.24–0.32 (0.32); MFW 0.07–0.09 (0.08); MTL 0.17–0.25 (0.21); PTL 0.19–0.22 (0.21); PNL 0.08–0.09 (0.09); PTH 0.15–0.19 (0.17); PTW 0.13–0.15 (0.15); PPL 0.11–0.16 (0.13); PPNL 0.10–0.13 (0.11); PPH 0.12–0.15 (0.13); PPW 0.17–0.20 (0.19); GL 0.43–0.58 (0.46); GW 0.35–0.42 (0.39); CI 75–79 (78); MI 15–22 (17); SI 37–44 (41); MLI 60–71 (71), PPLI 55–76 (62); PPI 121–133 (127); PSI 11–19 (16).

Head longer than wide (CI 75–79), in full-face view nearly subrectangular, about 1.4 times longer than wide. Posterior margin of head medially concave, posterolateral corners rounded, lateral margins straight. Mandible with five teeth. Anterior margin of clypeus slightly concave, nearly straight, and laterally convex. Antennae with ten segments. Scapes short (HL 0.51–0.63, SL 0.20–0.27, SI 37–44). Eyes present, consisting of two ommatidia (EL 0.02). Ocelli absent (weakly present in some major workers). Supraclypeal area well defined and triangular.

In profile view, promesonotum high and convex, metanotal groove deeply impressed. Propodeum about 1.6 times higher than long, dorsal face of propodeum flat, declining posteriorly, propodeum armed, posterodorsal corners each armed with a small triangular tooth, anterodorsal corners angulate, declivity of propodeum concave with thin lateral laminae. Propodeal lobes short and convex. Propodeal spiracle rounded and situated above mid-height of sclerite, and beyond mid-length of sclerite, by about the diameter of the spiracle; distance from propodeal spiracle to posterodorsal corner of propodeum almost three times the diameter of the spiracle (PSL 0.05–0.08), and distance to declivity almost the same as the diameter of the spiracle. In dorsal view, promesonotum about as long as wide, anterior margin and sides rounded; sides of propodeum weakly convex.

Petiole slightly longer than high (PTH 0.15–0.19, PTL 0.19–0.22) with short peduncle, ventral face medially convex. Combined outline of dorsal surface of peduncle and anterior face of node flat, posterior face of node vertical and slightly concave, dorsum weakly rounded, anterodorsal corner convex, and posterodorsal corner weakly rounded. Petiolar node slightly narrowed at its dorsum. Subpetiolar process small, weakly triangular, and shorter than the diameter of the propodeal spiracle, at about half the diameter of the spirale. Postpetiolar node rounded and lower than petiolar node. In dorsal view, postpetiolar node wider than petiolar node (PPW 0.17–0.20, PTW 0.13–0.15), petiolar node wider than long (PTW 0.13–0.15, PNL 0.08–0.09), anterior and posterior margins of petiole and postpetiole straight, sides rounded in petiole and strongly convex in postpetiole.

Dorsal surface of mandibles, lower median portion of clypeus and supraclypeal area smooth and shiny, with scattered piligerous punctae on head and mandibles. Head with longitudinal, well-defined rugae, which extend from propodeal lobes and posterior margin of supraclypeal area to posterior margin of head, with a few, weak rugulae on upper median portion of clypeus. In profile, posterolateral portion of cephalic dorsum usually smooth and shiny, sometimes with short longitudinal rugulations. Sides of pronotum smooth and shiny, while mesopleuron, metapleuron, petiole and ventral face of postpetiole are areolate. In dorsal view, promesonotum smooth and shiny medially, as well as postpetiole and gaster, finely areolate on propodeum, petiole and near anterior and posterior margins of promesonotum.

Lateral margins and posterior margin of head with short suberect hairs, scapes with appressed hairs, and outer margin of mandible with decumbent hairs. Mesosoma with short and long suberect hairs. Petiole and postpetiole with subdecumbent hairs, and longer suberect hairs, gaster with abundant subdecumbent hairs. Tibia with abundant subdecumbent and decumbent hairs. Color yellowish ferruginous.

#### Description of minor workers.

**Measurements** (n=7): HL 0.36–0.39; HW 0.29–0.33; SL 0.22–0.23; ML 0.09–0.11; EL 0.01; EM 0.11–0.12; HD 0.22–0.25; WL 0.38–0.42; PSL 0.03–0.04; PW 0.18–0.21; MFL 0.22–0.25; MFW 0.05–0.07; MTL 0.14–0.19; PTL 0.13–0.14; PNL 0.06–0.07; PTH 0.11–0.12; PTW 0.08–0.10; PPL 0.08–0.09; PPNL 0.07–0.09; PPH 0.08–0.09; PPW 0.12–0.13; GL 0.29–0.36; GW 0.24–0.28; CI 81–85; MI 24–28; SI 58–64, MLI 71–79; PPLI 57–64; PPI 130–150; PSI 9–14.

Head longer than wide (CI 81–85), in full-face view weakly subrectangular, about 1.2 times longer than wide. Posterior margin of head slightly concave medially, posterolateral corners rounded, lateral margins slightly convex, nearly straight. Mandibles with five teeth. Anterior margin of clypeus nearly straight to slightly concave medially, laterally angulate. Antennae with ten segments. Scape fails to reach the posterior margin of head (HL 0.36–0.39, SL 0.22–0.23, SI 58–64). Eyes present, consisting of one ommatidium (EL 0.01). Supraclypeal area almost triangular but poorly defined, and appears as an oblong depression.

In profile view, promesonotum weakly convex, nearly flat, metanotal groove deeply impressed. Propodeum about 1.5 times higher than long, dorsal face of propodeum nearly flat and declining posteriorly, posterodorsal corners each armed with a small triangular, (sometines angulate), tooth, anterodorsal corner nearly convex and higher than posterodorsal corner of promesonotum, declivity medially concave and with thin lateral laminae. Propodeal lobes triangular. Propodeal spiracle rounded and situated slightly above mid-height of sclerite by about half the diameter of the spiracle, and beyond mid-length of sclerite by about 1.5 times the diameter of the spiracle; distance from propodeal spiracle to posterodorsal corner of propodeum almost twice the diameter of the spiracle (PSL 0.03–0.04), and distance to declivity less than half the diameter of the spiracle. In dorsal view, promesonotum about 1.27 times longer than wide, anterior margin rounded, sides convex; sides of propodeum same as promesonotum.

Petiole slightly longer than high (PTL 0.13–0.14, PTH 0.11–0.12) with a short peduncle, ventral face medially convex. Combined outline of dorsal surface of peduncle and anterior face of node straight to slightly concave medially, posterior face of node vertical and slightly convex, anterodorsal and posterodorsal corners of petiolar node sloping, dorsum narrowed and rounded. Subpetiolar process weakly triangular, and shorter than the diameter of the propodeal spiracle. Postpetiolar node rounded and lower than petiolar node. Postpetiolar ventral process produced as a small triangular denticle. In dorsal view, petiolar node not as wide as postpetiolar node (PTW 0.08–0.10, PPW 0.12–0.13), and petiolar node wider than long (PNL 0.06–0.07, PTW 0.08–0.10), anterior and posterior margin of petiole and postpetiole straight, sides rounded in petiole and convex in postpetiole.

Dorsal surface of mandibles, clypeus and head smooth and shiny, with scattered piligerous punctae on head and mandibles. Gena and frontal lobes with fine linear rugulae. Mesosoma smooth and shiny, except for katepisternum and metapleuron which are areolate. Petiole and ventral face of postpetiole areolate-rugose. In dorsal view, mesosoma, postpetiole and gaster smooth and shiny, declivity and petiole finely areolate.

Lateral margins and posterior margin of head with abundant short subdecumbent hairs. Scapes with abundant decumbent hairs. Outer margin of mandible with appressed hairs. Mesosoma with suberect hairs. Petiole and postpetiole with short decumbent hairs and longer subdecumbent hairs. Tibia with abundant decumbent hairs. Gaster with abundant subdecumbent hairs. Color yellowish ferruginous.

#### Distribution and biology.

*Carebara betsi* is known only from the center of Madagascar (Figure 68), in montane rainforest with an elevation of 1410 m, and was collected in sifted litter using Winkler traps on a 50 sample transect. The microhabitats of this species are leaf mold and rotten wood.

#### Comments.

*Carebara betsi* is endemic to the montane rainforest on the high plateau of Madagascar and can be easily separated from the other species by the following combination of characters: combined outline of dorsal surface of peduncle and anterior face of node flat, and hairs on the gaster abundant and subdecumbent. Also major workers present well-marked longitudinal and parallel rugae. Other seven species are present in this area: *C. grandidieri*, *C. hainteny*, *C. jajoby*, *C. kabosy*, *C. mahafaly*, *C. nosindambo*, and *C. omasi*, which can be differentiated from *C. betsi* by the combination of characters mentioned above.

*C. betsi* does not have intermediates in the major worker subcaste.

#### Additional material examined.

**MADAGASCAR**: ***Antananarivo***: 3 km 41° NE Andranomay, 11.5 km 147° SSE Anjozorobe, -18.47333, 47.96, 1300 m, montane rainforest, 5–13.xii.2000, (*Fisher, Griswold et al.*).

### Carebara creolei

Taxon classificationAnimaliaHymenopteraFormicidae

Azorsa & Fisher sp. n.

http://zoobank.org/43A5D98C-9AA6-4798-B426-885D1560421A

#### Holotype.

(major worker), MAURITIUS, Brise Mt., Bambous, -20.3455, 57.75467, 200 m, rainforest, 27.v.2005, (*B.L. Fisher et al*.). Collection code BLF12072, (CASC: CASENT0060366). **Paratypes**: (6 major workers and 25 minor workers), with same data as holotype, 3 major workers (CASC: CASENT0060524, CASENT0060071, CASENT0060663), and 20 minor workers (BMNH: CASENT0060067, CASC: CASENT0060069, CASENT0060070, CASENT0060525, CASENT0060527, CASENT0060528, CASENT0060666, CASENT0060670, CASENT0060664, CASENT0060068, CASENT0060367, CASENT0060667, CASENT0060529, CASENT0060072, CASENT0060368, CASENT0060665, CASENT0060371, MCZ: CASENT0060668, MHNG: CASENT0060369, NHMB: CASENT0060370). 3 major workers and 4 minor workers from: Mauritius, Ile aux Aigrettes, -20.41883, 57.7305, 1 m, coastal scrub, 28.v.2005, (*B.L. Fisher et al*.), with collection code BLF12085, 3 major workers (BMNH: CASENT0060719, CASC: CASENT0060721, MCZ: CASENT0060720), and 4 minor workers (CASC: CASENT0060726, CASENT0060722, CASENT0060724, CASENT0060725). 2 minor workers from: Seychelles, St. Joseph Island, -5.437422, 53.363127, 22.vi.2003, (*J. Gerlach*), with collection code ANTC10036 (CASC: CASENT0189354) and ANTC10139 (CASC: CASENT0191438).

#### Diagnosis.

Antennae nine-segmented. **Major**: Head longer than wide, nearly subrectangular in full-face view, lateral margins straight and parallel; head longitudinally reticulate-rugose; mesosoma areolate-rugose; gaster smooth and shiny. **Minor**: Head sculpture areolate-rugose and mesosoma areolate-rugose.

**Figure 35.**
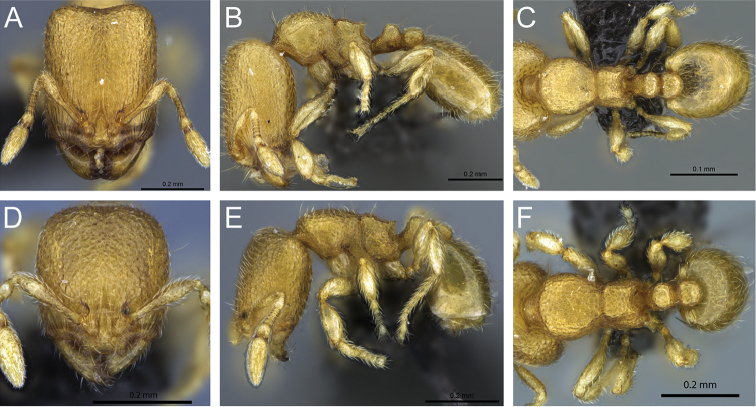
*Carebara creolei*–holotype. Major worker, CASENT0060366. **A** head in full-face view **B** body in profile view **C** body in dorsal view. Minor worker, CASENT0060670 **D** head in full-face view **E** body in profile view **F** body in dorsal view.

#### Description of major workers.

**Measurements** (n=8): HL 0.45–0.53 (0.46); HW 0.36–0.39 (0.37); SL 0.21–0.22 (0.21); ML 0.10–0.11 (0.11); EL 0.02; EM 0.10–0.11 (0.10); HD 0.19–0.24 (0.24); WL 0.34–0.39 (0.35); PSL 0.06–0.07 (0.07); PW 0.21–0.23 (0.22); MFL 0.22–0.23 (0.22); MFW 0.06–0.07 (0.06); MTL 0.19–0.20 (0.20); PTL 0.15; PNL 0.07–0.08 (0.07); PTH 0.10–0.11 (0.10); PTW 0.09–0.11 (0.10); PPL 0.08–0.09 (0.08); PPNL 0.07–0.08 (0.07); PPH 0.09; PPW 0.09–0.11(0.10); GL 0.36–0.46 (0.40), GW 0.29–0.35 (0.29); CI 73–82 (80); MI 19–24 (24); SI 40–47 (46); MLI 56–62 (59); PPLI 53–60 (53); PPI 100; PSI 15–19 (19).

Head longer than wide (CI 73–82), in full-face view nearly subrectangular, about 1.3 times longer than wide. Posterior margin of head medially concave, posterolateral corners rounded, lateral margins nearly straight. Mandibles with five teeth. Anterior margin of clypeus with a concavity flanked by a pair of triangular teeth. Antennae with nine segments. Scapes short (HL 0.45–0.53, SL 0.21–0.22, SI 40–47). Ocelli absent. Eye present, consisting of one ommatidium (EL 0.02). Supraclypeal area extends posteriorly toward antennal insertions.

In profile view, promesonotum convex, metanotal groove deeply impressed. Propodeum about 1.5 times higher than long, dorsal face of propodeum nearly concave closer to posterodorsal corner, and convex closer anterodorsal corner, propodeum armed, posterodorsal corners each armed with a triangular tooth upwardly directed, declivity of propodeum concave with lamellate lateral margination on each side. Propodeal lobes short and triangular. Propodeal spiracle rounded and situated slightly above mid-height of sclerite, and beyond mid-length of sclerite, by about half the diameter of the spiracle; distance from propodeal spiracle to posterodorsal corner of propodeum almost four times the diameter of the spiracle (PSL 0.06–0.07), and distance to declivity about 1.5 times the diameter of the spiracle. In dorsal view, promesonotum about as long as wide, anterior margin and sides rounded; sides of propodeum weakly concave in the middle.

Petiole longer than high (PTL 0.15, PTH 0.10–0.11) with relatively long peduncle, ventral face nearly flat but weakly convex medially. Combined outline of dorsal surface of peduncle and anterior face of node deeply concave medially, posterior face of node convex, anterodorsal corner rounded and posterodorsal corner convex, dorsum flat and declining posteriorly. Subpetiolar process produced as a small triangular denticle, slightly larger than propodeal spiracle. Postpetiolar node slightly convex, nearly flat, and lower than petiolar node. In dorsal view, postpetiolar node almost as wide as petiolar node (PTW 0.09–0.11, PPW 0.09–0.11), petiolar node wider than long (PTW 0.09–0.11, PNL 0.07–0.08), anterior and posterior margins of petiole straight, anterior margin of postpetiole straight, posterior margin rounded, sides of petiole rounded, sides of postpetiole convex.

Dorsal surface of mandibles, clypeus and supraclypeal area smooth and shiny, with scattered piligerous punctae on head and mandibles. Clypeus with a few poorly defined transverse rugae. Head reticulate-rugose in full-face view, with irregular longitudinal rugulae which do not reach posterior margin of head. Cephalic dorsum between frontal carinae with two longitudinal rugulae, extending from posterior clypeal margin to posterior quarter of head. In profile, posterolateral portion of cephalic dorsum areolate-rugose, except for smooth and shiny ventral area. Mesosoma areolate-rugose, except for middle sides of pronotum which are smooth and shiny. Petiole and ventral face of postpetiole areolate. In dorsal view, mesosoma areolate, petiole and postpetiole smooth and shiny, with scattered foveolae.

Lateral margins and posterior margin of head with short, sparse decumbent and appressed hairs. Scapes with appressed hairs. Outer margin of mandible with few decumbent hairs. Mesosoma with short decumbent hairs, and sparse long and erect hairs. Petiole, postpetiole and gaster with short decumbent to appressed hairs, and sparse long subdecumbent hairs. Tibia with decumbent hairs. Color yellowish ferruginous.

#### Description of minor workers.

**Measurements** (n=7): HL 0.31–0.33; HW 0.26–0.28; SL 0.18–0.20; ML 0.08–0.09; EL 0.01; EM 0.06–0.07; HD 0.14–0.21; WL 0.31–0.33; PSL 0.05–0.06; PW 0.17–0.19; MFL 0.17–0.21; MFW 0.04–0.05; MTL 0.13–0.15; PTL 0.10–0.11; PNL 0.06; PTH 0.07–0.09; PTW 0.07–0.08; PPL 0.06–0.07; PPNL 0.06; PPH 0.06–0.07; PPW 0.07; GL 0.22–0.33; GW 0.22–0.25; CI 79–88; MI 24–28; SI 58–63; MLI 61–81; PPLI 55–64; PPI 88–100; PSI 17–23.

Head a little longer than wide (CI 79–88), in full-face view nearly subquadrate, about 1.1 times longer than wide, narrowed anteriorly. Posterior margin of head nearly straight, posterolateral corners rounded, lateral margins slightly convex. Mandible with five teeth. Anterior margin of clypeus with a concavity flanked by a pair of triangular teeth. Antennae with nine segments. Scapes barely exceeding midpoint of head (HL 0.31–0.33, SL 0.18–0.20, SI 58–63). Eyes present, consisting of one ommatidium (EL 0.01). Supraclypeal area triangular and extends posteriorly toward antennal insertions.

In profile, promesonotum weakly convex, nearly flat, metanotal groove deeply impressed. Propodeum about 1.25 times higher than long, dorsal face of propodeum convex towards anterodorsal corner and medially concave, posterodorsal corners each armed with a triangular tooth, declivity of propodeum concave with lamellate lateral margination on each side. Propodeal lobes short and convex. Propodeal spiracle rounded and situated above mid-height of sclerite, and beyond mid-length of sclerite by about the diameter of the spiracle; distance from propodeal spiracle to posterodorsal corner of propodeum about four times the diameter of the spiracle (PSL 0.05–0.06), and distance to declivity about half the diameter of the spiracle. In dorsal view, promesonotum about 1.08 times longer than wide, anterior margin and sides of promesonotum rounded, sides of propodeum weakly concave near posterodorsal corner.

Petiole longer than high (PTL 0.10–0.11, PTH 0.07–0.09) with a relatively long peduncle, ventral face nearly flat to weakly convex in the middle. Combined outline of dorsal surface of peduncle and anterior face of node deeply concave, posterior face of node slightly convex, anterodorsal corner rounded, posterodorsal corner convex, dorsum flat and declining posteriorly. Subpetiolar process produced as a small denticle, usually the same as the diameter of the propodeal spiracle. Postpetiolar node nearly flat and slightly lower than petiolar node. In dorsal view, petiolar node almost as broad as postpetiolar node (PTW 0.07–0.08, PPW 0.07), petiolar node slightly wider than long (PTW 0.0.7–0.0.8, PNL 0.06), anterior margin of petiole convex, posterior margin straight, anterior margin of postpetiole nearly straight and posterior margin slightly convex, sides of petiole and postpetiole convex to rounded.

Dorsal surface of mandibles smooth and shiny with scattered piligerous punctae on head and mandibles, clypeus finely sculptured. Head longitudinally areolate-rugose, malar space with longitudinal rugae. Mesosoma, petiole and ventral face of postpetiole areolate. In dorsal view, mesosoma areolate-rugose, petiole and postpetiole weakly areolate, and gaster smooth and shiny.

Lateral margins and posterior margin of head with short decumbent to appressed hairs. Scapes with abundant decumbent hairs. Outer margin of mandible with a few decumbent hairs. Mesosoma with long suberect hairs, and short subdecumbent hairs. Petiole and postpetiole with short appressed hairs and longer subdecumbent hairs. Tibia with decumbent hairs and gaster with abundant short subdecumbent hairs and sparse longer subdecumbent hairs. Color yellowish ferruginous.

#### Distribution and biology.

*Carebara creolei* is only known from Mauritius and Seychelles (Figure 68). It occurs in the coastal scrub, mangrove, mixed forest, and rainforest. The species was collected at different elevations, ranging from 1 m to 200 m, using Winkler traps. The individuals and colonies were found in sifted litter, leaf mold, rotten wood, under coconut husks on the ground, and under rootmats/litter on rocks. The distribution of this species on two distant islands is interesting given the presence of invasive species on these islands. It is possible that *C. creolei* has been introduced to these islands, but a review of described species from outside the region, notability SE Asia, did not turn up any possible candidates. Until futher evidence is obtained, it is best to consider this species as a new species and native to the region.

#### Comments.

*Carebara creolei* is endemic to Mauritius and Seychelles and is the only species where minor and major workers have a sculptured on heads and mesosoma. This species and *C. grandidieri* are the only two species with nine antennal segments. *C. creolei* does not have intermediates in the major worker subcaste.

#### Additional material examined.

**MAURITIUS**: Ile aux Aigrettes, -20.41883, 57.7305, 1 m, coastal scrub, 28.v.2005, (*B.L. Fisher et al*.); **SEYCHELLES**: St. Joseph Island, -5.437422, 53.363127, 22.vi.2003, (*J. Gerlach*); Conception Island, -4.66311, 55.36821, 65 m, mixed forest, 12–13.ii.2010, (*B.L. Fisher et al*.)

### Carebara demeter

Taxon classificationAnimaliaHymenopteraFormicidae

Azorsa & Fisher sp. n.

http://zoobank.org/D384FB08-E21B-451F-9951-B1A752392226

#### Holotype.

(major worker), MADAGASCAR, Antsiranana, Réserve Spéciale Manongarivo, 14.5 km 220° SW Antanambao, -13.99833, 48.42833, 1175 m, montane rainforest, 20.x.1998, (*B.L. Fisher*). Collection code BLF01938, (CASC: CASENT0906155). **Paratypes**: (10 major worker and 9 minor workers), with same data as holotype, 10 major workers (BMNH: CASENT0192622, CASC: CASENT0248445, CASENT0248446, CASENT0248447, CASENT0248448, CASENT0248449, CASENT0248450, MCZ: CASENT0248451, MHNG: CASENT0248452, NHMB: CASENT0248453), and 9 minor workers (BMNH: CASENT0127683, CASC: CASENT0127688, CASENT0192568, CASENT0248454, CASENT0248455, CASENT0248456, CASENT0248457, CASENT0248458, MCZ: CASENT0248459).

#### Diagnosis.

Antennae ten-segmented. **Major**: Head longer than wide, nearly subrectangular in full-face view, lateral margins straight and parallel, head with longitudinal and irregular rugae, reticulate-rugose close to posterior margin of head; petiole triangular in profile view, combined outline of dorsal surface of peduncle and anterior face of node almost straight, ventral face medially convex, subpetiolar process present. **Minor**: Head longer than wide, slightly narrowed anteriorly, lateral margins weakly convex; dorsum of promesonotum and anterodorsal corner of propodeum at the same height, posterodorsal corner of propodeum each armed with a small triangular, angulate tooth; combined outline of dorsal surface of peduncle and anterior face of node weakly concave in the middle, posterior face slightly convex; gaster with abundant subdecumbent hairs.

**Figure 36.**
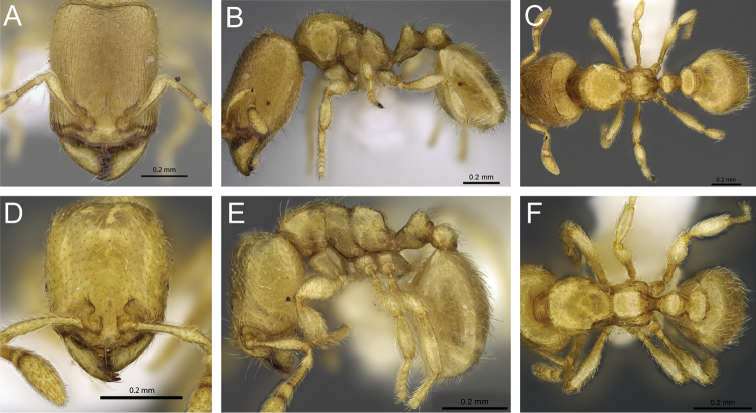
*Carebara demeter*–holotype. Major worker, CASENT0906157. **A** head in full-face view **B** body in profile view **C** body in dorsal view. Minor worker, CASENT0192568 **D** head in full-face view **E** body in profile view **F** body in dorsal view.

#### Description of major workers.

**Measurements** (n=12): HL 0.53–0.64 (0.62); HW 0.43–0.50 (0.47); SL 0.24–0.25 (0.25); ML 0.10–0.14 (0.13); EL 0.02–0.03 (0.02); EM 0.14–0.17 (0.17); HD 0.28–0.37 (0.35); WL 0.48–0.58 (0.53); PSL 0.04–0.06 (0.06); PW 0.28–0.35 (0.33); MFL 0.26–0.31 (0.29); MFW 0.07–0.08 (0.08); MTL 0.20–0.23 (0.23); PTL 0.17–0.20 (0.20); PNL 0.08–0.10 (0.09); PTH 0.14–0.17 (0.15); PTW 0.12–0.14 (0.14); PPL 0.08–0.14 (0.12); PPNL 0.10–0.13 (0.11); PPH 0.10–0.14 (0.12); PPW 0.16–0.18 (0.18); GL 0.47–0.60 (0.49); GW 0.34–0.45 (0.42); CI 74–81 (76); MI 17–22 (21); SI 39–45 (40), MLI 60–70 (62); PPLI 44–74 (60); PPI 129–150 (129); PSI 9–14 (13).

Head longer than wide (CI 74–81), in full-face view nearly subrectangular, about 1.3 times longer than wide. Posterior margin of head slightly concave in the middle, posterolateral corners rounded, lateral margins straight. Mandibles with five teeth. Anterior margin of clypeus nearly straight, laterally convex. Antennae with ten segments. Scapes short (HL 0.53–0.64, SL 0.24–0.25, SI 39–45). Eyes present, consisting of two or three ommatidia (EL 0.02–0.03). Supraclypeal area triangular and appears as a short longitudinal depression, surpassing frontal carinae.

In profile, posterolateral corner of head with (medium and larger major workers) or without (smaller major workers) a small, obtuse tooth resembling a horn. Promesonotum high and strongly convex, metanotal groove deeply impressed. Propodeum about 1.6 times higher than long, dorsal face of propodeum slightly convex, nearly flat, declining posteriorly, posterodorsal corners of propodeum each armed with a small triangular tooth, declivity of propodeum concave, nearly flat, with thin lateral laminae. Propodeal lobes triangular. Propodeal spiracle rounded and situated above mid-height of sclerite by about half the diameter of the spiracle, and beyond mid-length of sclerite by about 1.5 times the diameter of the spiracle, distance from propodeal spiracle to posterodorsal corner of propodeum about twice the diameter of the spiracle (PSL 0.04–0.06), and distance to declivity same as the diameter of the spiracle. In dorsal view, promesonotum about as long as wide, anterior margin and sides rounded; sides of propodeum straight.

Petiole slightly longer than high (PTL 0.17–0.20, PTH 0.14–0.17) with short peduncle, ventral face medially convex. Combined outline of dorsal surface of peduncle and anterior face of node nearly straight, posterior face of node vertical and slightly concave, anterodorsal corner convex, posterodorsal corner rounded, dorsum convex. Subpetiolar process produced as denticle, slightly larger than the diameter of the propodeal spiracle. Postpetiolar node convex and lower than petiolar node. In dorsal view, petiolar node not as wide as postpetiolar node (PTW 0.12–0.14, PPW 0.16–0.18), petiolar node wider than long (PTW 0.12–0.14, PNL 0.08–0.10), anterior and posterior margins of petiole and postpetiole nearly straight, sides rounded in petiole and postpetiole.

Dorsal surface of mandibles, clypeus, supraclypeal area, and median area of head smooth and shiny; scattered piligerous punctae on mandibles and head. Head with longitudinal and irregular rugae and reticulate-rugose near the posterior margin. In profile, posterolateral portion of cephalic dorsum smooth and shiny. Mesosoma smooth and shiny, except for katepisternum (areolate), metapleuron (longitudinally and parallel areolate rugose), and propodeal lobes (areolate-rugose). Petiole and ventral face of postpetiole areolate-rugose. In dorsal view, mesosoma, petiole, postpetiole and gaster smooth and shiny, declivity of propodeum, anterior and posterior face of petiole finely areolate.

Lateral margins and posterior margin of head with short suberect hairs, short appressed hairs below eye level. Scapes with appressed hairs. Outer margin of mandible with decumbent to appressed hairs. Mesosoma with long and short suberect hairs. Petiole and postpetiole with short decumbent hairs and long subdecumbent hairs. Tibia with decumbent hairs. Gaster with abundant decumbent hairs, and longer suberect hairs. Color yellowish ferruginous.

#### Description of minor workers.

**Measurements** (n=6): HL 0.34–0.37; HW 0.29–0.32; SL 0.20–0.23; ML 0.09–0.11; EL 0.01; EM 0.10–0.11; HD 0.21–0.23; WL 0.35–0.39; PSL 0.02–0.04; PW 0.18–0.21; MFL 0.21–0.24, MFW 0.05–0.06, MTL 0.14–0.17, PTL 0.12–0.14; PNL 0.06–0.07; PTH 0.10–0.11; PTW 0.08–0.09; PPL 0.08–0.09; PPNL 0.07–0.08; PPH 0.07–0.08; PPW 0.11–0.12; GL 0.30–0.40, GW 0.24–0.28, CI 85– 89; MI 24–31; SI 59–64, MLI 72–77, PPLI 57–67, PPI 133–138, PSI 6–13.

Head longer than wide (CI 85–89), in full-face view nearly subrectangular, about 1.2 times longer than wide, and slightly narrowed anteriorly. Posterior margin of head nearly straight to slightly concave in the middle, posterolateral corners strongly convex, lateral margins slightly convex. Mandibles with five teeth. Anterior margin of clypeus nearly straight medially and laterally convex. Antennae with ten segments. Scape fails to reach the posterior margin of head (HL 0.34–0.37, SL 0.20–0.23, SI 59–64). Eye present, consisting of two ommatidia (EL 0.01). Supraclypeal area almost triangular and not well defined.

In profile view, promesonotum convex, metanotal groove deeply impressed. Propodeum about 1.3 times higher than long, dorsal face of propodeum nearly flat, declining posteriorly, anterodorsal corner convex, posterodorsal corners each armed with a small angulate tooth, declivity of propodeum slightly concave with thin lateral laminae which extend from posterodorsal corner of propodeum to propodeal lobe. Propodeal lobes triangular. Propodeal spiracle nearly oval and situated above mid-height of sclerite, and beyond mid-length of sclerite by about the diameter of the spiracle; distance from propodeal spiracle to posterodorsal corner of propodeum about 2.5 times the diameter of the spiracle (PSL 0.02–0.04), and distance to declivity about 1.4 times the diameter of the spiracle. In dorsal view, promesonotum slightly longer than wide (about 1.13 times longer than wide), anterior margin and sides weakly rounded; sides of propodeum weakly convex.

Petiole slightly longer than high (PTL 0.12–0.14, PTH 0.10–0.11) with short peduncle, ventral face medially convex. Combined outline of dorsal surface of peduncle and anterior face of node weakly concave in the middle, posterior face of node vertical and nearly straight, anterodorsal corner convex, posterodorsal corner rounded, dorsum rounded. Subpetiolar process produced as denticle, slightly larger than the diameter of the propodeal spiracle. Postpetiolar node rounded and lower than petiolar node. In dorsal view, postpetiolar node wider than petiolar node (PPW 0.11–0.12, PTW 0.08–0.09), petiolar node wider than long (PNL 0.06–0.07, PTW 0.08–0.09), anterior and posterior margins of petiole slightly convex, anterior and posterior margin of postpetiole straight, sides of petiole and postpetiole rounded.

Dorsal surface of head, mandibles and clypeus smooth and shiny, with scattered piligerous punctae on head and mandibles, except in central longitudinal area, malar area with short longitudinal rugae. Mesosoma smooth and shiny, except for katepisternum (areolate), and metapleuron (finely areolate-rugose). Petiole and ventral face of postpetiole areolate. In dorsal view, mesosoma, petiole, postpetiole, and gaster smooth and shiny.

Lateral margins of head with decumbent hairs, posterior margin with short subdecumbent hairs, scapes with abundant subdecumbent hairs. Outer margin of mandibles with decumbent hairs. Mesosoma with short suberect hairs, and long subdecumbent hairs. Petiole and postpetiole with decumbent hairs and longer subdecumbent hairs. Tibia with abundant decumbent hairs. Gaster with abundant, short subdecumbent hairs, and sparse suberect hairs. Color yellowish ferruginous.

#### Distribution and biology.

*Carebara demeter* is known only from northwestern Madagascar (Figure 68), in montane rainforest with an elevation of 1175 m. It was collected using Winkler traps, and individuals were found in leaf litter, leaf mold and rotten wood.

#### Comments.

*Carebara demeter* is endemic to the northwest of Madagascar. It can be confused with *C. tana* due to the form of the head. However, *C. demeter* has irregular longitudinal rugae and is rugoreticulate near the posterior margin of the head, while the frontal area is smooth and shiny in *C. tana*. The petiolar node of *C. tana* is thicker than *C. demeter*. The anterior margin of the petiole is nearly straight in *C. demeter*, but slightly concave in *C. tana*. The anteroventral corner of the petiole has a well-developed triangular tooth in *C. demeter*, and a small angulate triangular tooth in *C. tana*. The anterodorsal corner of the petiole is convex in *C. tana*, but rounded in *C. demeter*. The posterodorsal corner is convex in *C. demeter*, but slightly concave in *C. tana*. Only two additional species were recorded in this area: *C. grandidieri*, and *C. jajoby*.

*C. demeter* does not have intermediates in the major worker subcaste.

### Carebara dota

Taxon classificationAnimaliaHymenopteraFormicidae

Azorsa & Fisher sp. n.

http://zoobank.org/DD931F9C-69AA-4BB8-87BB-97EB147D017F

#### Holotype.

(major worker), MADAGASCAR, Fianarantsoa, 43 km S Ambalavao, Réserve Andringitra, -22.23333, 47, 825 m, rainforest, 5.x.1993, (*B.L. Fisher*). Collection code BLF00747, (CASC: CASENT0192623). **Paratypes**: (5 major workers and 19 minor workers), with same data as holotype, 5 major workers (BMNH: CASENT0248460, CASC: CASENT0281313, CASENT0127684, CASENT0127650, CASENT0127649), and 19 minor workers (BMNH: CASENT0004053, CASC: CASENT0004050, CASENT0002885, CASENT0004051, CASENT0002887, CASENT0002886, CASENT0002884, CASENT0002882, CASENT0002883, CASENT0004057, CASENT0004052, CASENT0004056, CASENT0004049, CASENT0004055, CASENT0127703, CASENT0192570, MCZ: CASENT0227028, MHNG: CASENT0227023, NHMB: CASENT0127685).

#### Diagnosis.

Antennae ten-segmented. **Major**: Head longer than wide, lateral margins straight, head with longitudinal and parallel rugae, reticulate rugose close to posterolateral corners of head; promesonotum strongly convex, dorsum of propodeum slightly convex, nearly flat, posterodorsal corners with small angulate tooth; combined outline of dorsal surface of peduncle and anterior face of node slightly concave, posterior margin vertical and nearly straight; gaster with subdecumbent hairs. **Minor**: Head longer than wide, lateral margins slightly convex; propodeum with small, triangular, angulate teeth; combined outline of dorsal surface of peduncle and anterior face of node nearly straight, posterior margin vertical and concave.

**Figure 37.**
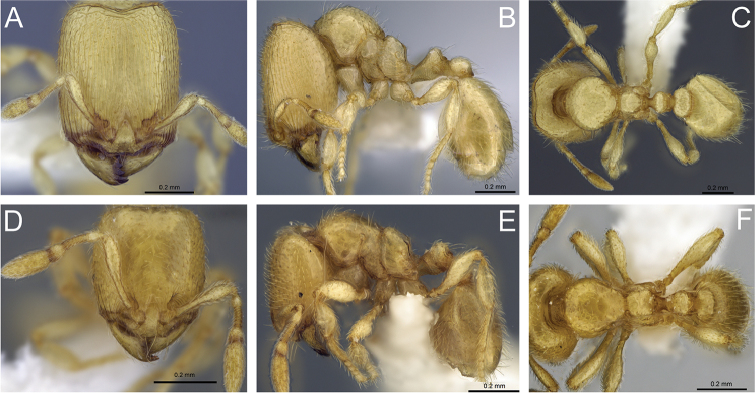
*Carebara dota*–holotype. Major worker, CASENT0192623. **A** head in full-face view **B** body in profile view **C** body in dorsal view. Minor worker, CASENT0192570 **D** head in full-face view **E** body in profile view **F** body in dorsal view.

#### Description of major workers.

**Measurements** (n=7): HL 0.61–0.71 (0.62); HW 0.47–0.56 (0.50); SL 0.22–0.28 (0.25); ML 0.12–0.15 (0.12); EL 0.02–0.03 (0.02); EM 0.15–0.19 (0.18); HD 0.35–0.41 (0.36); WL 0.53–0.62 (0.53); PSL 0.05–0.07 (0.05); PW 0.33–0.40 (0.35); MFL 0.30–0.36 (0.32); MFW 0.08–0.09 (0.08), MTL 0.20–0.27 (0.23); PTL 0.19–0.23 (0.19); PNL 0.09–0.10 (0.09); PTH 0.15–0.18 (0.15); PTW 0.13–0.16 (0.14); PPL 0.13–0.16 (0.13); PPNL 0.11–0.14 (0.12); PPH 0.13–0.16 (0.14); PPW 0.17–0.22 (0.19); GL 0.47–0.60 (0.50); GW 0.39–0.55 (0.39); CI 77–83 (81); MI 19–21 (19); SI 35–41 (40), MLI 58–64 (64); PPLI 62–74 (68); PPI 129–143 (136); PSI 9–13 (10).

Head longer than wide (CI 77–83), in full-face view nearly subrectangular, about 1.3 times longer than wide. Posterior margin of head medially concave, posterolateral corners rounded, lateral margins straight. Mandibles with five teeth. Anterior margin of clypeus slightly concave, and laterally angulate. Antennae with ten segments. Scapes short (HL 0.61–0.71, SL 0.22–0.28, SI 35–41). Eyes present, consisting of two ommatidia (EL 0.02–0.03). Supraclypeal area triangular and well defined.

In profile, posterolateral corner of head with a small, triangular tooth resembling a horn. Promesonotum high and strongly convex, metanotal groove deeply impressed. Propodeum lower than promesonotum. Propodeum about 1.8 times higher than long, dorsal face of propodeum slightly convex, nearly flat, declining posteriorly, posterodorsal corners each armed with a very small angulate tooth, declivity of propodeum vertical and concave with thin lateral laminae. Propodeal lobes triangular. Propodeal spiracle nearly oval and situated above mid-height of sclerite by about half the diameter of the spiracle, and beyond mid-length of sclerite by about the diameter of the spiracle; distance from propodeal spiracle to posterodorsal corner of propodeum about two times the diameter of the spiracle (PSL 0.05–0.07), and distance to declivity same as the diameter of the spiracle. In dorsal view, promesonotum about as long as wide (1.1 times longer than wide), anterior margin and sides of promesonotum rounded; sides of propodeum weakly convex.

Petiole approximately as high as long (PTH 0.15–0.18, PTL 0.19–0.23) and with a relatively short peduncle, ventral face slightly convex in the middle. Combined outline of dorsal surface of peduncle and anterior face of node concave, posterior face of node vertical and nearly straight, anterodorsal corner convex, posterodorsal corner rounded, dorsum convex. Subpetiolar process produced as a small denticle, almost as large as the diameter of the propodeal spiracle. Postpetiolar node strongly convex and lower than petiolar node. In dorsal view, petiolar node not as broad as postpetiolar node (PTW 0.13–0.16, PPW 0.17–0.22) and petiolar node wider than long (PNL 0.09–0.10, PTW 0.13–0.16), anterior and posterior margins of petiole straight, anterior margin of postpetiole concave, posterior margin nearly straight, sides of petiole rounded and strongly convex in postpetiole.

Dorsal surface of mandibles, clypeus and supraclypeal area smooth and shiny, with scattered piligerous punctae on mandibles and head. Head with well defined longitudinal rugae, and reticulate rugose near the posterior margin, gena with longitudinal rugae extending to the posterolateral corners. In profile, posterolateral portion of cephalic dorsum smooth and shiny but with finely longitudinal reticulations. Mesosoma smooth and shiny, except for katepisternum (areolate), and metapleuron (finely areolate and with longitudinal rugae). Petiole and ventral face of postpetiole areolate. In dorsal view mesosoma, petiole, postpetiole and gaster, smooth and shiny except for declivity, anterior face of petiole and postpetiole which are finely areolate.

Lateral margins of head with short subdecumbent hairs, posterolateral corners with scattered suberect hairs. Scapes with appressed hairs. Outer margin of mandibles with short appressed hairs. Mesosoma with short and long suberect hairs, petiole and postpetiole with decumbent hairs and longer subdecumbent hairs. Tibia with decumbent hairs. Gaster with abundant subdecumbent hairs. Color yellowish.

#### Description of minor workers.

**Measurements** (n=8): HL 0.38–0.41; HW 0.33–0.36; SL 0.23–0.25; ML 0.09–0.11; EL 0.01–0.02; EM 0.10–0.11; HD 0.20–0.25; WL 0.39–0.42; PSL 0.03–0.04; PW 0.18–0.24; MFL 0.23–0.29; MFW 0.06–0.07; MTL 0.15–0.21; PTL 0.12–0.16; PNL 0.06–0.07; PTH 0.11–0.13; PTW 0.09–0.10; PPL 0.08–0.10; PPNL 0.08–0.09; PPH 0.08–0.10; PPW 0.12–0.14; GL 0.32–0.42; GW 0.26–0.30; CI 80–92; MI 22–27; SI 58–63; MLI 69–83; PPLI 53–83; PPI 130–144; PSI 9–12.

Head longer than wide (CI 80–92), in full-face view nearly subquadrate, about 1.1 times longer than wide, weakly narrowed anteriorly. Posterior margin of head slightly concave in the middle, posterolateral corners rounded, lateral margins slightly convex. Mandibles with five teeth. Anterior margin of clypeus medially convex, and laterally angulate. Antennae with ten segments. Scape fails to reach the posterior margin of head (HL 0.38–0.41, SL 0.23–0.25, SI 58–63). Eyes present, consisting of one ommatidium (EL 0.01–0.02). Supraclypeal area triangular and well defined.

In profile view, promesonotum weakly convex, metanotal groove deeply impressed. Propodeum about 1.65 times higher than long, dorsal face of propodeum nearly flat and declining posteriorly, posterodorsal corners of propodeum each armed with a small triangular tooth, anterodorsal corner of propodeum convex, declivity vertical and concave with thin lateral laminae. Propodeal lobes triangular. Propodeal spiracle rounded and situated above mid-height of sclerite by about the diameter of the spiracle, and beyond mid-length of sclerite by about two times the diameter of the spiracle; distance from propodeal spiracle to posterodorsal corner of propodeum almost twice the diameter of the spiracle (PSL 0.03–0.04), and distance to declivity same as the diameter of the spiracle. In dorsal view, promesonotum about as long as wide (1.1 times longer than wide), anterior margin weakly convex, sides convex and narrowed posteriorly; sides of propodeum straight to weakly convex.

Petiole about as high as long (PTL 0.12–0.16, PTH 0.11–0.13) and with a short peduncle, ventral face medially convex. Combined outline of dorsal surface of peduncle and anterior face of node nearly straight, posterior face of node vertical and slightly concave, anterodorsal corner convex, posterodorsal corner rounded, dorsum slightly convex. Subpetiolar process produced as a small denticle, almost as large as the diameter of the propodeal spiracle. Postpetiolar node convex and lower than petiolar node. In dorsal view, postpetiolar node wider than petiolar node (PPW 0.12–0.14, PTW 0.09–0.10), petiolar node wider than long (PNL 0.06–0.07, PTW 0.09–0.10), anterior and posterior margin of petiole nearly straight, anterior margin of postpetiole slightly concave, posterior margin convex, sides of petiole convex and rounded in postpetiole.

Dorsal surface of mandibles, median portion of clypeus and supraclypeal area smooth and shiny, with scattered piligerous punctae on head and mandibles, except for smooth and shiny frons; malar area and frontal lobes with longitudinal rugae. Mesosoma smooth and shiny, except for katepisternum (areolate), and metapleuron (weakly areolate and with longitudinal and parallel rugae). Petiole and ventral face of postpetiole areolate. In dorsal view, mesosoma, petiole, postpetiole and gaster smooth and shiny.

Lateral margins of head with subdecumbent hairs, posterior margin of head with suberect hairs. Scapes with abundant decumbent hairs. Outer margin of mandible with decumbent hairs. Mesosoma with short and long suberect hairs. Petiole and postpetiole with short decumbent hairs and longer subdecumbent hairs. Tibia with abundant decumbent hairs. Gaster with abundant subdecumbent hairs, and sparse suberect hairs. Color yellowish ferruginous.

#### Distribution and biology.

*Carebara dota* is known only from southwestern and north-central Madagascar (Figure 68). This species was found in habitats such as gallery forest, grassland, littoral rainforest, montane rainforest, montane rainforest edge, and rainforest. *C. dota* was collected at elevations ranging from 10 m to 1670 m using maxi-Winkler and pitfall traps. Individuals and colonies were found in leaf mold and rotten wood.

#### Comments.

*Carebara dota* is endemic to Madagascar, and major workers are easily distinguished from other species by the longitudinal and parallel rugae on the dorsal surface of the head. Six species were recorded at the same places were this species was collected: *C. bara*, *C. grandidieri*, *C. hainteny*, *C. jajoby*, *C. nosindambo*, and *C. sampi*, which can be separated from *C. dota* by the characters mentioned above.

*C. dota* does not have intermediates in the major worker subcaste.

#### Additional material examined.

**MADAGASCAR**: ***Fianarantsoa***: 45 km S. Ambalavao, -22.21667, 47.01667, 785 m, rainforest, 25.ix.1993, (*B.L. Fisher*); Fianarantsoa, 28 km SSW Ambositra, Ankazomivady, -20.775, 47.16833, 1670 m, grassland, 11.i.1998, (*B.L. Fisher*); Fianarantsoa, Réserve Spéciale Ivohibe, 7.5 km ENE Ivohibe, -22.47, 46.96, 900 m, rainforest, 7–12.x.1997, (*B.L.Fisher (Sylvain*)); Fianarantsoa, 28 km SSW Ambositra, Ankazomivady, -20.775, 47.16833, 1670 m, montane rainforest edge, 14.i.1998, (*B.L. Fisher*); ***Toliara***: Manombo, -22.8123, 43.73932, 165 m, gallery forest, TS3, 22–24.v.2004, (*Frontier Wilderness Project*); Toliara, Parc National d’Andohahela, Col du Sedro, 3.8 km 113° ESE Mahamavo, 37.6 km 341° NNW Tolagnaro, -24.76389, 46.75167, 900 m, montane rainforest, 21–25.i.2002, (*Fisher, Griswold et al.*); Toliara, Réserve Spéciale Kalambatritra, Ampanihy, -23.4635, 46.4631, 1270 m, montane rainforest, 9–10.ii.2009, (*B.L. Fisher et al*.); Toliara, Forêt de Petriky, 12.5 km W 272° Tolagnaro, -25.06167, 46.87, 10 m, littoral rainforest, 22.xi.1998, (*B.L. Fisher*).

### Carebara grandidieri

Taxon classificationAnimaliaHymenopteraFormicidae

(Forel, 1891)

Oligomyrmex grandidieri Forel, 1891: 201. Holotype queen (1 queen, CASENT0101999), (MHNG) [examined]: Madagascar, Antananarivo (*Camboué*). Combination in Carebara: Fernández, 2004: 235.
Oligomyrmex voeltzkowi Forel, 1907: 77. Holotype queen (1 queen, CASENT0906665, GBIF-D/FoCol1927), (ZMHB): Madagascar, Toamasina, Tamatave (Voeltzkow). [examined] **syn. n.** Combination in Carebara: Fernández, 2004: 235.

#### Diagnosis.

Antennae nine-segmented. **Major**: Head longer than wide, nearly rectangular, margins straight and parallel; petiole with relatively long peduncle, ventral face flat, petiolar node rounded. **Minor**: Head longer than wide, nearly subrectangular; petiole with relatively long peduncle, petiolar node rounded.

**Figure 38.**
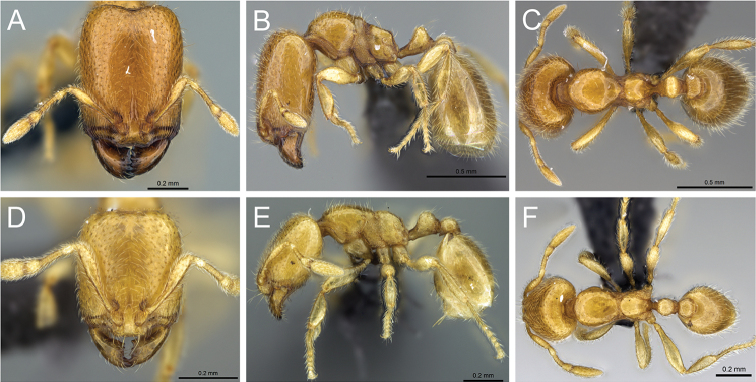
*Carebara grandidieri*. Major worker, CASENT0035958. **A** head in full-face view **B** body in profile view **C** body in dorsal view. Minor worker, CASENT0036011 **D** head in full-face view **E** body in profile view **F** body in dorsal view.

**Figure 39.**
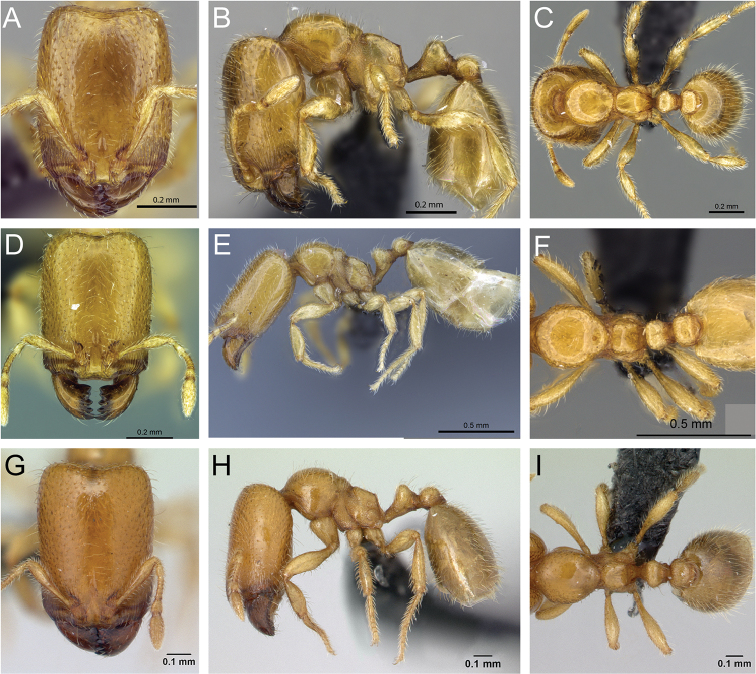
Intermediates of *Carebara grandidieri*. Major workers, CASENT0021673. **A** head in full-face view **B** body in profile view **C** body in dorsal view. CASENT0444643 **D** head in full-face view **E** body in profile view **F** body in dorsal view. CASENT0133170 **G** head in full-face view **H** body in profile view **I** body in dorsal view.

**Figure 40.**
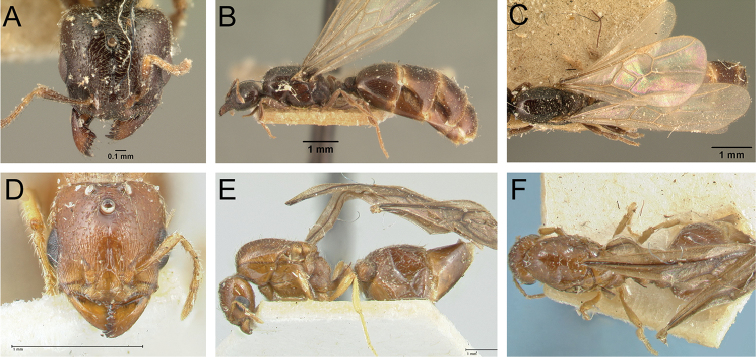
*Carebara grandidieri*-lectotype and *Carebara voeltzkowi* (= *C. grandidieri* syn. n.). Queen, CASENT0101999. **A** head in full-face view **B** body in profile view **C** body in dorsal view. Queen, CASENT0906665 **D** head in full-face view **E** body in profile view **F** body in dorsal view.

#### Description of major workers.

**Measurements** (n=22): HL 0.56–0.84; HW 0.44–0.61; SL 0.25–0.32; ML 0.12–0.21; EL 0.01–0.03; EM 0.14–0.19; HD 0.30–0.45; WL 0.51–0.74; PSL 0.05–0.09; PW 0.26–0.38; MFL 0.29–0.42; MFW 0.06–0.10; MTL 0.26–0.37; PTL 0.18–0.28; PNL 0.09–0.14; PTH 0.12–0.20; PTW 0.12–0.19; PPL 0.11–0.16; PPNL 0.09–0.14; PPH 0.10–0.17; PPW 0.13–0.21; GL 0.44–0.85; GW 0.35–0.65; CI 71–79; MI 20–27; SI 38–46; MLI 63–74; PPLI 50–67; PPI 100–123; PSI 10–15.

Head longer than wide (CI 71–79), in full-face view nearly subrectangular, about 1.3 times longer than wide. Posterior margin of head medially concave, posterolateral corners rounded, lateral margins straight. Mandibles with six teeth. Anterior margin of clypeus concave and laterally convex. Frons without median ocellus. Antennae with nine segments. Scapes short (HL 0.56–0.84, SL 0.25–0.32, SI 38–46). Eyes present, consisting of one to three ommatidia (EL 0.01–0.03). Supraclypeal area acutely triangular, extending forward beyond the antennal insertions.

In profile view, promesonotum convex, mesonotum shallowly present and lower than dorsum of promesonotum, metanotal groove present. Propodeum lower than promesonotum, about 1.2 times higher than long, dorsal face of propodeum nearly flat and declining posteriorly, posterodorsal corner angulate to dentate, declivity of propodeum slightly concave with thin lateral laminae in direction of propodeal lobe. Propodeal lobes short and convex. Propodeal spiracle rounded and situated above mid-height of sclerite, and beyond mid-length of sclerite by about half the diameter of the spiracle; distance from propodeal spiracle to posterodorsal corner of propodeum about 2.5 times the diameter of the spiracle (PSL 0.05–0.09), and distance to declivity almost twice the diameter of the spiracle. In dorsal view, promesonotum about 1.2 times longer than wide, anterior margin of promesonotum rounded, sides convex and narrowed posteriorly; sides of propodeum weakly convex or straight.

Petiole with relatively long peduncle, ventral face flat. Combined outline of dorsal surface of peduncle and anterior face of node deeply concave in the middle, posterior face of node slightly convex, anterodorsal and posterodorsal corner convex, dorsum rounded. Subpetiolar process produced as a small denticle, smaller than the diameter of the propodeal spiracle. Postpetiolar node rounded and lower than petiolar node. In dorsal view, petiolar node as broad as postpetiolar node (PTW 0.12–0.19, PPW 0.13–0.21) and petiolar node wider than long (PNL 0.09–0.14, PTW 0.12–0.19), anterior and posterior margins of petiole nearly straight, anterior margin of postpetiole concave and convex posterior margin, sides rounded in petiole and convex in postpetiole, narrowed posteriorly.

Dorsal surface of mandibles, upper median portion of clypeus and supraclypeal area smooth and shiny, with scattered piligerous punctae on head and mandibles. Head with longitudinal rugae along the gena, and frontal lobes. Lower median portion of clypeus with transverse rugae. In profile, posterolateral portion of cephalic dorsum smooth and shiny. Mesosoma smooth and shiny, except for propleuron and mesopleuron (areolate), and metapleuron (longitudinally rugulose). Ventral face of petiole and postpetiole longitudinally areolate. In dorsal view mesosoma, petiole, postpetiole and gaster smooth and shiny, except for declivity of propodeum, anterior and posterior face of petiole finely areolate.

Lateral margins and posterior margin of head with suberect to subdecumbent hairs. Scapes with subdecumbent to decumbent hairs. Outer margin of mandibles with short decumbent hairs. Mesosoma with short and long suberect hairs. Petiole and postpetiole with short subdecumbent hairs and long suberect hairs. Tibia with subdecumbent to decumbent hairs. Gaster with abundant suberect to decumbent hairs. Color yellowish ferruginous.

#### Description of minor workers.

**Measurements** (n=18): HL 0.39–0.49; HW 0.31–0.40; SL 0.23–0.30; ML 0.08–0.13; EL 0.01–0.02; EM 0.11–0.13; HD .022–0.28; WL 0.40–0.52; PSL 0.03–0.05; PW 0.18–0.24; MFL 0.24–0.31; MFW 0.06–0.08; MTL 0.19–0.27; PTL 0.16–0.19; PNL 0.09–0.11; PTH 0.10–0.13; PTW 0.09–0.11; PPL 0.08–0.11; PPNL 0.07–0.10; PPH 0.07–0.10; PPW 0.10–0.12; GL 0.31–0.55; GW 0.22–0.32; CI 79–86; MI 19–28; SI 57–65; MLI 73–82; PPLI 47–63; PPI 100–120; PSI 9–13.

Head longer than wide (CI 79–86), in full-face view nearly subrectangular, about 1.2 times longer than wide. Posterior margin of head weakly concave, nearly straight, posterolateral corners rounded, lateral margins slightly convex, nearly straight. Mandibles with five teeth. Anterior margin of clypeus concave and laterally angulate. Antennae with nine segments. Scape fails to reach the posterior margin of head (HL 0.39–0.49, SL 0.23–0.30, SI 57–65). Eyes present, consisting of one ommatidium (EL 0.01–0.02). Supraclypeal area triangular but poorly defined.

In profile view, promesonotum weakly convex, nearly flat, metanotal groove present. Propodeum about 1.3 times higher than long, dorsal face of propodeum convex, posterodorsal corners angulate, or with a small triangular tooth, anterodorsal corner convex, declivity concave with thin lateral laminae in direction of propodeal lobe. Propodeal lobes short and triangular. Propodeal spiracle rounded and situated above mid-height of sclerite by about two times the diameter of the spiracle, and beyond mid-length of sclerite by about 1.5 times the diameter of the spiracle, distance from propodeal spiracle to posterodorsal corner of propodeum almost three times the diameter of the spiracle (PSL 0.03–0.05), and distance to declivity less than twice the diameter of the spiracle. In dorsal view, promesonotum about 1.25 times longer than wide, anterior margin rounded, sides convex; sides of propodeum straight.

Petiole with a relatively long peduncle, petiole longer than high (PTL 0.16–0.19, PTH 0.10–0.13), ventral face flat. Combined outline of dorsal surface of peduncle and anterior face of node deeply concave in the middle, posterior face of node slightly convex, anterodorsal and posterodorsal corner convex, dorsum rounded. Subpetiolar process produced as a small denticle, smaller than the diameter of the propodeal spiracle. Postpetiolar node strongly convex and lower than petiolar node. In dorsal view, petiolar node almost as broad as postpetiolar node (PTW 0.09–0.11, PPW 0.10–0.12), and petiolar node as broad as long (PNL 0.09–0.11, PTW 0.09–0.11), anterior and posterior margin of petiole convex, anterior margin of postpetiole concave and convex posteriorly, sides of petiole and postpetiole strongly convex.

Dorsal surface of mandibles, clypeus, supraclypeal area, and head smooth and shiny, with scattered piligerous punctae on head and mandibles. Head with transverse rugae near the posterior margin of head, gena and frontal lobes with longitudinal rugae. Mesosoma smooth and shiny, except for propleuron and mesopleuron (areolate), and metapleuron (finely areolate-rugose). Lateral margins of petiole finely areolate-rugose, except for petiolar node and ventral face of postpetiole which are areolate. In dorsal view mesosoma, petiole, postpetiole and gaster smooth and shiny, except for declivity of propodeum, anterior and posterior face of petiole which are finely areolate.

Lateral margins of head and scape with subdecumbent to decumbent hairs. Posterior margin of head with short suberect hairs. Outer margin of mandibles with decumbent hairs. Mesosoma with short and long suberect hairs. Petiole and postpetiole with short decumbent hairs and long subdecumbent hairs. Tibia with decumbent hairs. Gaster with longer suberect to decumbent hairs. Color yellowish ferruginous.

#### Distribution and biology.

*Carebara grandidieri* is a widespread and common species in the Malagasy region, and has been recorded on Madagascar, Comoros and Mayotte (Figure 68). This species was collected in the following environments: along the roadside, coastal scrub, dry forest, gallery forest, grassland, littoral forest, montane rainforest, open secondary vegetation, rainforest, spiny forest/thicket, tropical dry forest, *Uapaca* woodland, and urban/garden. *C. grandidieri* was sampled at elevations ranging from 10 m to 1550 m. Individuals and colonies were collected in rotten logs, rotting tree stumps, soil, termite mounds, leaf litter, under moss, litter on rocks, under rotten logs, under stones, and in leaf litter.

#### Comments.

*Carebara grandidieri* is widely distributed throughout Madagascar, Comoros and Mayotte, and co-occurs with almost all *Carebara* species recorded in the Malagasy region. The morphology of this species is highly variable (Figure 39, 40). There are different populations that initially appear to be distinct species. However, we could not find consistent characters to split *C. grandidieri* into additional species. The characters that vary across populations include: a) pilosity that varies from suberect to decumbent hairs, and is more noticeable on the gaster; b) posterior corner of propodeum, which varies from a pair of small triangular and angulate upwardly directed teeth, to nearly convex (in specimens from the same colony it is possible to see the gradual change from nearly convex to slightly dentate); c) petiole, where the node is thicker in profile and dorsal view, in some populations; and d) the diameter of the propodeal spiracle, which is smaller in populations from the north of Madagascar.

Upon examining all *Carebara* workers in the Malagasy region, we found only two species with nine antennal segments, *C. grandidieri* and *C. creolei*, and *C. creolei* is present only in Comoros and the Seychelles. All other *Carebara* species present in Madagascar have 10 or 11 antennal segments. The main character present in the queens of *C. grandidieri* and *C. voeltzkowi* described by Forel, is the presence of nine antennal segments, while the morphological differences between these specimens are not sufficient to merit placement in separate species. It is possible that *C. grandidieri* represents a species complex, with two or three different species, but much more detailed studies, including molecular studies, will likely be required to confirm this.

The main variations between the queens (Figure 40) of the two species are in the form of the head and the posterodorsal corner of the propodeum. After examining queens collected in different localities we see that the shape of the head is very variable in the queen caste, with some populations with lateral margins of the head convex, and others nearly straight. In the queen caste of *C. grandidieri*, the posterodorsal corner of the propodeum is unarmed, while in *C. voeltzkowi* it has a pair of small and angulate triangular teeth, though we found that this character is highly variable in the queens and the major worker caste. For this reason, we are including *C. voeltzkowi* as a junior synonym of *C. grandidieri*. In general, the queen of *C. grandidieri* can be separated from others by the following combination of characters: head longer than wide, in full-face view nearly rectangular, or nearly subquadrate, posterior margin of head slightly concave in the middle, posterolateral corners rounded; lateral margins straight to slightly convex; mandible with six or seven teeth; anterior margin of clypeus slightly concave; antennae with nine segments; ocelli present, eyes present.

#### Material examined.

**COMOROS**: Anjouan, -12.25764, 44.38915, 20 m, along roadside, 27.i.2009, (*B.L. Fisher et al*.); Mohéli Ouallah, -12.32717, 43.65952, 10 m, coastal scrub, 17.i.2009, (*B.L. Fisher et al*.); Mohéli Ouallah, -12.30668, 43.66407, 275 m, rainforest, 21–24.i.2009, (*B.L. Fisher et al*.); Mohéli Lac Boundouni, -12.37915, 43.85165, 25 m, dry forest, 20–21.i.2009, (*B.L. Fisher et al*.); Mohéli Ouallah, -12.30353, 43.66827, 500 m, rainforest, 18–22.i.2009, (*B.L. Fisher et al*.); Mohéli Ouallah, -12.30353, 43.66827, 500 m, rainforest, 18–19.i.2009, (*B.L. Fisher et al*.). **MADAGASCAR**: ***Antananarivo***. Réserve Spéciale d’Ambohitantely, Forêt d’ Ambohitantely, 20.9 km 72° NE d’ Ankazobe, -18.22528, 47.28683, 1410 m, montane rainforest, 17–22.iv.2001, (*Fisher, Griswold et al.*); Antananarivo, [Antananarivo], (*Camboué*); Antananarivo, Ankalalahana, -19.00659, 47.1122, 1375 m, *Uapaca* Woodland, 29–31.iii.2011, (*B.L. Fisher et al*.); ***Antsiranana***. Forêt de Binara, 7.5 km 230° SW Daraina, -13.255, 49.61667, 375 m, tropical dry forest, 1.xii.2003, (*B.L. Fisher*); Antsiranana, Forêt d’ Antsahabe, 11.4 km 275° W Daraina, -13.21167, 49.55667, 550 m, tropical dry forest, 12.xii.2003, (*B.L. Fisher*); Antsiranana, Forêt d’ Andavakoera, 21.4 km 75° ENE Ambilobe; 4.6 km 356° N Betsiaka, -13.11833, 49.23, 425 m, rainforest, 15.xii.2003, (*B.L. Fisher*); Antsiranana, Forêt de Bekaraoka, 6.8 km 60° ENE Daraina, -13.16667, 49.71, 150 m, tropical dry forest, 7.xii.2003, (*B.L. Fisher*); Antsiranana, Réserve Analamerana, 16.7 km 123° Anivorano-Nord, -12.80467, 49.37383, 225 m, tropical dry forest, 3.xii.2004, (*B.L. Fisher*); Antsiranana, Réserve Analamerana, 28.4 km 99° Anivorano-Nord, -12.74667, 49.49483, 60 m, tropical dry forest, 5.xii.2004, (*B.L. Fisher*); Antsiranana, Forêt Ambato, 26.6 km 33° Ambanja, -13.4645, 48.55167, 150 m, rainforest, 8.xii.2004, (*B.L. Fisher*); Antsiranana, Ambondrobe, 41.1 km 175° Vohemar, -13.71533, 50.10167, 10 m, littoral rainforest, 29.xi.2004, (*B.L. Fisher*); Antsiranana, Forêt Ambato, 26.6 km 33° Ambanja, -13.4645, 48.55167, 150 m, rainforest, 10.xii.2004, (*B.L. Fisher*); Antsiranana, Ambondrobe, 41.1 km 175° Vohemar, -13.71533, 50.10167, 10 m, littoral rainforest, 30.xi.2004, (*B.L. Fisher*); Antsiranana, Parc National de Marojejy, Manantenina River, 28.0 km 38° NE Andapa, 8.2 km 333° NNW Manantenina, -14.43667, 49.775, 450 m, rainforest, 23.xi.2004, (*B.L. Fisher*); Antsiranana, Forêt d’Ampombofofo, -12.09949, 49.33874, 25 m, littoral forest, 21–22.xi.2007, (*B.L. Fisher et al*.); Antsiranana, Nosy Faly, -13.36435, 48.49137, 40 m, open secondary vegetation, 25.ii.2013, (*B.L. Fisher et al*.); Antsiranana, Nosy Be, Réserve Naturelle Intégrale de Lokobe, 6.3 km 112° ESE Hellville, -13.41933, 48.33117, 30 m, rainforest, 19–24.iii.2001, (*Fisher, Griswold et al.*); Antsiranana, Réserve Spéciale de l’Ankarana, 22.9 km 224° SW Anivorano Nord, -12.90889, 49.10983, 80 m, tropical dry forest, 10–16.ii.2001, (*Fisher, Griswold et al.*); Antsiranana, Forêt d’Anabohazo, 21.6 km 247° WSW Maromandia, -14.30889, 47.91433, 120 m, tropical dry forest, 11–16.iii.2001, (*Fisher, Griswold et al.*); Antsiranana, Réserve Spéciale de l’Ankarana, 13.6 km 192° SSW Anivorano Nord, -12.86361, 49.22583, 210 m, tropical dry forest, 16–21.ii.2001, (*Fisher, Griswold et al.*); Antsiranana, Réserve Spéciale de l’Ankarana, 22.9 km 224° SW Anivorano Nord, -12.90889, 49.10983, 80 m, tropical dry forest on tsingy, 10–16.ii.2001, (*Fisher, Griswold et al.*); Antsiranana, Ampasindava, Forêt d’Ambilanivy, 3.9 km 181° S Ambaliha, -13.79861, 48.16167, 600 m, rainforest, 4–9.iii.2001, (*Fisher, Griswold et al.*); Antsiranana, Forêt d’Ampondrabe, 26.3km 10° NNE Daraina, -12.97, 49.7, 175 m, tropical dry forest, 10.xii.2003, (*B.L. Fisher*); Antsiranana, Réserve Spéciale d’Ambre, 3.5 km 235° SW Sakaramy, -12.46889, 49.24217, 325 m, tropical dry forest, 26–31.i.2001, (*Fisher, Griswold et al.*). ***Fianarantsoa***. Parc National d’Isalo, Sahanafa River, 29.2 km 351° N Ranohira, -22.31333, 45.29167, 500 m, gallery forest, 10–13.ii.2003, (*Fisher, Griswold et al.*); Fianarantsoa, Parc National d’Isalo, 9.1 km 354° N Ranohira, -22.48167, 45.46167, 725 m, gallery forest, 27–31.i.2003, (*Fisher, Griswold et al.*); Fianarantsoa, Parc National d’Isalo, Ambovo Springs, 29.3 km 4° N Ranohira, -22.29833, 45.35167, 990 m, *Uapaca* woodland, 9–14.ii.2003, (*Fisher, Griswold et al.*); Fianarantsoa, Réserve Forestière d’Agnalazaha, Mahabo, 42.9 km 215° Farafangana, -23.19383, 47.723, 20 m, littoral rainforest, 19.iv.2006, (*B.L. Fisher et al*.); Fianarantsoa, Réserve Spéciale Manombo 24.5 km 228° Farafangana, -23.01583, 47.719, 30 m, rainforest, 20.iv.2006, (*B.L. Fisher et al*.); Fianarantsoa, Forêt d’Atsirakambiaty, 7.6 km 285° WNW Itremo, -20.59333, 46.56333, 1550 m, grassland, 22–26.i.2003, (*Fisher, Griswold et al.*). ***Mahajanga*** Parc National tsingy de Bemaraha, 10.6 km ESE 123° Antsalova, -18.70944, 44.71817, 150 m, tropical dry forest on tsingy, 16–20.xi.2001, (*Fisher, Griswold et al.*); Mahajanga, Mahavavy River, 6.2 km 145° SE Mitsinjo, -16.05167, 45.90833, 20 m, gallery forest, 1–5.xii.2002, (*Fisher, Griswold et al.*); Mahajanga, Parc National de Namoroka, 17.8 km 329° WNW Vilanandro, -16.37667, 45.32667, 100 m, tropical dry forest, 8–12.xi.2002, (*Fisher, Griswold et al.*); Mahajanga, Réserve Spéciale de Bemarivo, 23.8 km 223° SW Besalampy, -16.925, 44.36833, 30 m, tropical dry forest, 19–23.xi.2002, (*Fisher, Griswold et al.*); Mahajanga, Parc National de Namoroka, 9.8 km 300° WNW Vilanandro, -16.46667, 45.35, 140 m, tropical dry forest, 4–8.xi.2002, (*Fisher, Griswold et al.*); Mahajanga, Parc National de Namoroka, 16.9 km 317° NW Vilanandro, -16.40667, 45.31, 100 m, tropical dry forest, 12–16.xi.2002, (*Fisher, Griswold et al.*); Mahajanga, Forêt de Tsimembo, 8.7 km 336° NNW Soatana, -19.02139, 44.44067, 20 m, tropical dry forest, 21–25.xi.2001, (*Fisher, Griswold et al.*); Mahajanga, Parc National tsingy de Bemaraha, 2.5 km 62° ENE Bekopaka, Ankidrodroa River, -19.13222, 44.81467, 100 m, tropical dry forest on tsingy, 11–15.xi.2001, (*Fisher, Griswold et al.*); Mahajanga, Réserve Forestière Beanka, 50.7 km E Maintirano, -17.88021, 44.46877, 140 m, tropical dry forest on tsingy, 29.x.-1.xi.2009, (*B.L. Fisher et al*.); Mahajanga, Réserve Forestière Beanka, 50.2 km E Maintirano, -18.02649, 44.05051, 250 m, tropical dry forest on tsingy, 19–22.x.2009, (*B.L. Fisher et al*.); Mahajanga, Réserve Forestière Beanka, 52.7 km E Maintirano, -18.0622, 44.52587, 300 m, tropical dry forest on tsingy, 24–27.x.2009, (*B.L. Fisher et al*.); Mahajanga, Parc National d’Ankarafantsika, Forêt de Tsimaloto, 18.3 km 46° NE de Tsaramandroso, -16.22806, 47.14361, 135 m, tropical dry forest, 2–8.iv.2001, (*Fisher, Griswold et al.*); Mahajanga, Parc National tsingy de Bemaraha, 3.4 km 93° E Bekopaka, Tombeau Vazimba, -19.14194, 44.828, 50 m, tropical dry forest, 6–10.xi.2001, (*Fisher, Griswold et al.*); Mahajanga, Parc National d’Ankarafantsika, Ampijoroa Station Forestière, 40 km 306° NW Andranofasika, -16.32083, 46.81067, 130 m, tropical dry forest, 26.iii.-1.iv.2001, (*Fisher, Griswold et al.*); Mahajanga, Parc National d’Ankarafantsika, Ampijoroa Station Forestière, 5.4 km 331° NW Andranofasika, -16.29889, 46.813, 70 m, tropical dry forest, 26.iii.-1.iv.2001, (*Fisher, Griswold et al.*); Mahajanga, Réserve d’Ankoririka, 10.6 km 13° NE de Tsaramandroso, -16.26722, 47.04861, 210 m, tropical dry forest, 9–14.iv.2001, (*Fisher, Griswold et al.*); Mahajanga, Forêt de Tsimembo, 11.0 km 346° NNW Soatana, -18.99528, 44.4435, 50 m, tropical dry forest, 21–25.xi.2001, (*Fisher, Griswold et al.*). ***Toamasina***. Parcelle K7 Tampolo, -17.28333, 49.41667, 10 m, littoral forest, 20.iv.2004, (*Malagasy ant team*); Toamasina, Forêt d’Analava Mandrisy, 5.9 km 195º Antanambe, -16.48567, 49.847, 10 m, littoral rainforest, 13.xi.2005, (*B.L. Fisher et al*.); Toamasina, Mahavelona (Foulpointe), -17.66667, 49.5, 20.v.1993, (*A. Pauly*); Toamasina, Station Forestière Analamazaotra, Analamazaotra 1.3 km S Andasibe, -18.38466, 48.41271, 980 m, montane rainforest, 11–13.xii.2007, (*B.L. Fisher et al*.); Toamasina, Réserve Forestière Tampolo, 95.2 km N Toamasina, -17.27808, 49.42853, 20 m, littoral rainforest, 7.v.2008, (*B.L. Fisher et al*.); Toamasina, Tamatave, (*Voeltzkow*); ***Toliara***. Parc National de Kirindy Mite, 16.3 km 127° SE Belo sur Mer, -20.79528, 44.147, 80 m, tropical dry forest, 6–10.xii.2001, (*Fisher, Griswold et al.*); Toliara, Réserve Privé Berenty, Forêt de Malaza, Mandraré River, 8.6 km 314° NW Amboasary, -25.00778, 46.306, 40 m, gallery forest, 6.ii.2002, (*Fisher, Griswold et al.*); Toliara, Forêt de Mite, 20.7 km 29° WNW Tongobory, -23.52417, 44.12133, 75 m, gallery forest, 27.ii.-3.iii.2002, (*Fisher, Griswold et al.*); Toliara, Réserve Privé Berenty, Forêt de Bealoka, Mandraré River, 14.6 km 329° NNW Amboasary, -24.95694, 46.2715, 35 m, gallery forest, 3–8.ii.2002, (*Fisher, Griswold et al.*); Toliara, Forêt de Mahavelo, Isantoria River, -24.75833, 46.15717, 110 m, spiny forest/thicket, 28.i.-1.ii.2002, (*Fisher, Griswold et al.*); Toliara, Réserve Spéciale de Cap Sainte Marie, 12.3 km 262° W Marovato, -25.58167, 45.16833, 200 m, spiny forest/thicket, 11–15.ii.2002, (*Fisher, Griswold et al.*); Toliara, Parc National d’Andohahela, Forêt de Manantalinjo, 33.6 km 63° ENE Amboasary, 7.6 km 99° E Hazofotsy, -24.81694, 46.61, 150 m, spiny forest/thicket, 12–16.i.2002, (*Fisher, Griswold et al.*); Toliara, Parc National de Tsimanampetsotsa, 6.7 km 130° SE Efoetse, 23.0 km 175° S Beheloka, -24.10056, 43.76, 25 m, spiny forest/thicket, 18–22.iii.2002, (*Fisher, Griswold et al.*); Toliara, Réserve Spéciale d’Ambohijanahary, Forêt d’Ankazotsihitafototra, 35.2 km 312° NW Ambaravaranala, -18.26667, 45.40667, 1050 m, montane rainforest, 13–17.i.2003, (*Fisher, Griswold et al.*); Toliara, Réserve Spéciale d’Ambohijanahary, Forêt d’Ankazotsihitafototra, 34.6 km 314° NW Ambaravaranala, -18.26, 45.41833, 1100 m, montane rainforest, 16.i.2003, (*Fisher, Griswold et al.*); Toliara, Forêt de Kirindy, 15.5 km 64° ENE Marofandilia, -20.045, 44.66222, 100 m, tropical dry forest, 28.xi.-3.xii.2001, (*Fisher, Griswold et al.*); Toliara, Antafoky, -23.48778, 44.0775, 60 m, gallery forest, 9.ii.2002, (*Frontier Project*); Toliara, Manombo, -22.8123, 43.73932, 165 m, gallery forest, TS3, 22–24.v.2004, (*Frontier Wilderness Project*); Toliara, Amboasary, -25.03883, 46.3835, 25 m, urban/garden, 9.xii.2006, (*B.L. Fisher et a*l.); Toliara, Manatantely, 8.9km NW Tolagnaro, -24.9815, 46.92567, 100 m, rainforest, 27.xi.2006, (*B.L. Fisher et al*.); Toliara, Réserve Spéciale Kalambatritra, Ambinanitelo, -23.45707, 46.4473, 1300 m, grassland, 8.ii.2009, (*B.L. Fisher et al*.); Toliara, Réserve Spéciale Kalambatritra, Ambinanitelo, -23.45373, 46.45773, 1345 m, grassland, 11.ii.2009, (*B.L. Fisher et al*.); Toliara, Réserve Spéciale Kalambatritra, -23.4185, 46.4583, 1365 m, grassland, 8.ii.2009, (*B.L. Fisher et al*.); Toliara, Beza-Mahafaly, 27 km E Betioky, -23.65, 44.63333, 135 m, tropical dry forest, 23.iv.1997, (*B.L. Fisher*); Toliara, Makay Mts., -21.21985, 45.32396, 500 m, gallery forest on sandy soil, 25.xi.2010, (*B.L. Fisher et al*.); Toliara, Makay Mts, -21.22284, 45.32477, 490 m, gallery forest on sandy soil, 24.xi.-1.xii.2010, (*B.L. Fisher et al*.); Toliara, Makay Mts., -21.20978, 45.34184, 525 m, gallery forest on sandy soil, 27.xi-2.xii.2010, (*B.L. Fisher et al*.); Toliara, Mahafaly Plateau, 6.2 km 74° ENE Itampolo, -24.65361, 43.99667, 80 m, spiny forest/thicket, 21–25.ii.2002, (*Fisher, Griswold et al.*); Toliara, Réserve Spéciale de Cap Sainte Marie, 14.9 km 261° W Marovato, -25.59444, 45.14683, 160 m, spiny forest/thicket, 13–19.ii.2002, (*Fisher, Griswold et al.*); **MAYOTTE**: Dapani, -12.96279, 45.15037, 135 m, rainforest, 2–4.xii.2007, (*B.L. Fisher et al*.); MAYOTTE: Mont Benara, -12.87585, 45.15672, 425 m, rainforest, 30.xi.-2.xii.2007, (*B.L. Fisher et al*.); MAYOTTE: Mont Chongui, -12.95903, 45.13411, 380 m, rainforest, 28–30.xi.2007, (*B.L. Fisher et al*.); MAYOTTE: Mont Combani, -12.80632, 45.15314, 370 m, rainforest, 25–28.xi.2007, (*B.L. Fisher et al*.).

### Carebara hainteny

Taxon classificationAnimaliaHymenopteraFormicidae

Azorsa & Fisher sp. n.

http://zoobank.org/5C71CCE3-AECF-4BFD-99E2-0C816B243EA4

#### Holotype.

(major worker), MADAGASCAR, Antananarivo, 3 km 41° NE Andranomay, 11.5 km 147° SSE Anjozorobe, -18.47333, 47.96, 1300 m, montane rainforest, 5–13.xii.2000, (*Fisher, Griswold et al.*). Collection code BLF02378, (CASC: CASENT0410494). **Paratypes**: (7 major workers, and 26 minor workers), with same data as holotype. 21 minor workers (BMNH: CASENT0410551, CASC: CASENT0410541, CASENT0410542, CASENT0410553, CASENT0410561, CASENT0410557, CASENT0410540, CASENT0410546, CASENT0410560, CASENT0410568, CASENT0410567, CASENT0410566, CASENT0410507, CASENT0410508, CASENT0410509, CASENT0410510, CASENT0410511, CASENT0410515, MCZ: CASENT0410512, MHNG: CASENT0410513, NHMB: CASENT0410555). 2 major workers and 3 minor workers with same data as holotype but with collection code BLF02464, 2 major workers (CASC: CASENT0410495, CASENT0410496), and 3 minor workers (CASC: CASENT0410516, CASENT0410524, CASENT0410518). 3 major workers and 2 minor workers with same data as holotype but with collection code BLF02506, 3 major workers (BMNH: CASENT0410498, CASC: CASENT0410497, MCZ: CASENT0410499), and 2 minor workers (CASC: CASENT0410529, CASENT0410530). 1 major worker from: Madagascar, Fianarantsoa, Parc National de Ranomafana, Vatoharanana River, 4.1 km 231° SW Ranomafana, -21.29, 47.43333, 1100 m, montane rainforest, 27–31.iii.2003, (*Fisher*, *Griswold et al.*) and collection code BLF08400 (CASC: CASENT0039833. 1 major worker from: Madagascar, Fianarantsoa, 2 km W Andrambovato, along river Tatamaly, -21.51167, 47.41, 1075 m, montane rainforest, 3–5.vi.2005, (*B.L. Fisher et al*.) and collection code BLF12164, (CASC: CASENT0061535).

#### Diagnosis.

Antennae ten-segmented. **Major**: Head subrectangular, posterior margin of head concave, lateral margins nearly straight and parallel; propodeum with a pair of small triangular teeth, dorsum of propodeum nearly flat; head with longitudinal and parallel rugose-reticulate and with longitudinal rugae laterally; posterolateral corners of head with a small, dentiform horn; gaster with abundant, suberect hairs. **Minor**: Head slightly longer than wide, nearly subquadrate; posterodorsal corner of propodeum with a pair of triangular teeth, dorsum nearly flat; combined outline of dorsal surface of peduncle and anterior face of node nearly straight, posterior margin vertical and slightly convex, anterodorsal corner convex; gaster with abundant suberect hairs.

**Figure 41.**
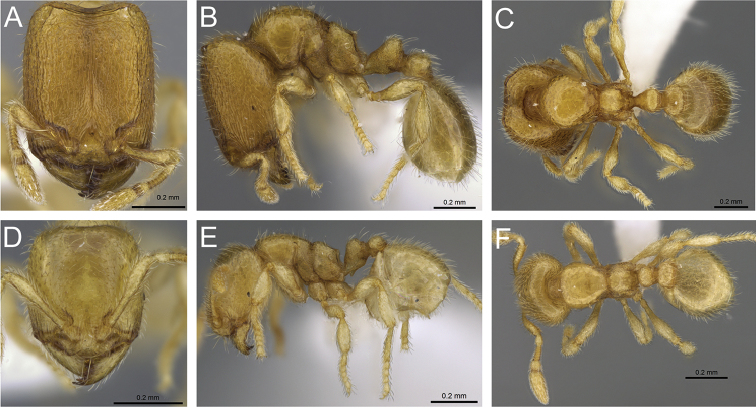
*Carebara hainteny*–holotype. Major worker, CASENT0410494: **A** head in full-face view **B** body in profile view **C** body in dorsal view. Minor worker, CASENT0410542: **D** head in full-face view **E** body in profile view **F** body in dorsal view.

#### Description of major workers.

**Measurements** (n=10): HL 0.56–0.68 (0.63); HW 0.43–0.53 (0.50); SL 0.24–0.27 (0.26); ML 0.10–0.14 (0.12); EL 0.02–0.04 (0.02); EM 0.16–0.19 (0.18); HD 0.31–0.38 (0.35); WL 0.50–0.57 (0.56); PSL 0.06–0.07 (0.06); PW 0.28–0.35 (0.32); MFL 0.28–0.36 (0.31); MFW 0.07–0.10 (0.08); MTL 0.22–0.25 (0.25); PTL 0.20–0.23 (0.23); PNL 0.08–0.10 (0.09); PTH 0.16–0.19 (0.17); PTW 0.12–0.16 (0.15); PPL 0.11–0.15 (0.14); PPNL 0.10–0.13 (0.11); PPH 0.12–0.14 (0.14); PPW 0.18–0.21 (0.20); GL 0.45–0.65 (0.48); GW 0.36–0.45 (0.42); CI 75–82 (79); MI 17–23 (19); SI 38–44 (41), MLI 60–73 (62); PPLI 55–75 (61); PPI 127–150 (133); PSI 11–14 (12).

Head longer than wide (CI 75–82), in full-face view subrectangular, about 1.3 times longer than wide. Posterior margin of head concave, posterolateral corners rounded, lateral margins slightly convex. Mandibles with five teeth. Anterior margin of clypeus medially concave and laterally angulate. Antennae with ten segments. Scapes short (HL 0.56–0.68, SL 0.24–0.27, SI 38–44). Ocelli are absent. Eyes present, consisting of one ommatidium (EL 0.02–0.04). Supraclypeal area acutely triangular, extending forward beyond the antennal insertions.

In profile view, posterolateral corner of head with a short triangular tooth resembling a horn. Promesonotum high and convex, metanotal groove deep. Propodeum about 1.65 times higher than long, dorsal face of propodeum nearly flat and declining posteriorly, posterodorsal corners of propodeum each armed with a triangular tooth, declivity of propodeum vertical, flat with thin lateral laminae. Propodeal lobes triangular. Propodeal spiracle rounded and situated above mid-height of sclerite by about half the diameter of the spiracle, and beyond mid-length of sclerite by about the diameter of the spiracle; distance from propodeal spiracle to posterodorsal corner of propodeum about 2.5 times the diameter of the spiracle (PSL 0.06–0.07), and distance to declivity same as the diameter of the spiracle. In dorsal view, promesonotum almost as long as wide (about 1.02 times longer than wide), anterior margin and sides of promesonotum rounded, narrowed posteriorly; sides of propodeum weakly convex.

Petiole slightly longer than high (PTL 0.20–0.23, PTH 0.16–0.19) and with a short peduncle, ventral face weakly convex in the middle. Combined outline of dorsal surface of peduncle and anterior face of node slightly concave, nearly straight, posterior face of node vertical, nearly straight, anterodorsal corner convex, posterodorsal corner rounded, dorsum convex. Subpetiolar process produced as denticle, slightly larger than the diameter of the propodeal spiracle. Postpetiolar node rounded and lower than petiolar node. In dorsal view, petiolar node not as broad as postpetiolar node (PTW 0.12–0.16, PPW 0.18–0.21), petiolar node wider than long (PTW 0.12–0.16, PNL 0.08–0.10), anterior and posterior margin of petiole and postpetiole nearly straight, sides rounded in petiole and postpetiole.

Dorsal surface of mandibles, clypeus and supraclypeal area smooth and shiny, with piligerous punctae on head and mandibles. Head with longitudinal irregular and parallel rugose-reticulate, transverse irregular rugae near posterior margin of head, gena with longitudinal rugae extending to posterolateral lobes, reticulate close to posterolateral corners. In profile, posterolateral portion of cephalic dorsum smooth and shiny. Mesosoma smooth and shiny, except for katepisternum (areolate) and metapleuron (areolate and with longitudinal and parallel rugae). Petiole and ventral face of postpetiole areolate. In dorsal view mesosoma, petiole, postpetiole and gaster, smooth and shiny, except for anterior and posterior faces of petiole which are areolate.

Lateral margins of head with short subdecumbent hairs, posterior margin of head with suberect hairs. Scapes with abundant appressed hairs. Outer margin of mandibles with decumbent hairs. Mesosoma with long and short erect and suberect hairs. Petiole and postpetiole with short decumbent hairs and longer subdecumbent hairs. Tibia with abundant decumbent hairs. Gaster with sparse, suberect hairs and abundant subdecumbent hairs. Color yellowish ferrugineus.

#### Description of minor workers.

**Measurements** (n=7): HL 0.35–0.39; HW 0.31–0.35; SL 0.21–0.24; ML 0.08–0.11; EL 0.01–0.02; EM 0.10–0.12; HD 0.21–0.24; WL 0.35–0.42; PSL 0.02–0.05; PW 0.18–0.21; MFL 0.21–0.25, MFW 0.05–0.06; MTL 0.15–0.18; PTL 0.12–0.15; PNL 0.06–0.07; PTH 0.10–0.12; PTW 0.08–0.10; PPL 0.07–0.10; PPNL 0.07–0.08; PPH 0.07–0.10; PPW 0.11–0.13; GL 0.35–0.39; GW 0.23–0.30; CI 87–92; MI 23–29; SI 60–64, MLI 66–75; PPLI 54–71; PPI 122–150; PSI 7–15.

Head slightly longer than wide (CI 87–92), in full-face view nearly subquadrate, about 1.1 times longer than wide. Posterior margin of head shallowly straight, posterolateral corners rounded, lateral margins weakly convex. Mandibles with five teeth. Anterior margin of clypeus almost straight and laterally angulate. Antennae with ten segments. Scape fails to reach the posterior margin of head (HL 0.35–0.39, SL 0.21–0.24, SI 60–64). Eyes present, consisting of one ommatidium (EL 0.01–0.02). Supraclypeal area triangular but poorly defined.

In profile view, promesonotum weakly convex, metanotal groove deeply impressed. Propodeum about 1.3 times higher than long, dorsal face of propodeum slightly concave and declining posteriorly, anterodorsal corner rounded, posterodorsal corners each armed with a triangular tooth, declivity slightly concave with thin lateral laminae. Propodeal lobes convex. Propodeal spiracle weakly oval and situated above mid-height of sclerite by about half the diameter of the spiracle, and beyond mid-length of sclerite by about the diameter of the spiracle, distance from propodeal spiracle to posterodorsal corner of propodeum about two times the diameter of the spiracle (PSL 0.02–0.05), and distance to declivity about 1.5 times the diameter of the spiracle. In dorsal view, promesonotum almost as long as wide (about 1.03 times longer than wide), anterior margin of promesonotum weakly convex, sides rounded and narrowed posteriorly; sides of propodeum convex.

Petiole slightly longer than high (PTL 0.12–0.15, PTH 0.10–0.12) and with a short peduncle, ventral face medially convex. Combined outline of dorsal surface of peduncle and anterior face of node straight, posterior face of node vertical and weakly concave, anterodorsal corner convex, posterodorsal corner rounded, dorsum convex. Subpetiolar process produced as denticle, slightly larger than the diameter of the propodeal spiracle. Postpetiolar node nearly rounded and lower than petiolar node. In dorsal view, postpetiolar node wider than petiolar node (PPW 0.11–0.13, PTW 0.08–0.10), petiolar node wider than long (PTW 0.0.8–0.10, PNL 0.06–0.0.7), anterior and posterior margin of petiole and postpetiole convex, sides of petiole and postpetiole rounded.

Dorsal surface of head, mandibles, clypeus and supraclypeal area smooth and shiny, with scattered piligerous punctae on head and mandibles. Gena and frontal lobes with longitudinal rugae. Mesosoma smooth and shiny, except for katepisternum (areolate) and metapleuron (areolate and with longitudinal and parallel rugae). Petiole and ventral face of postpetiole areolate. In dorsal view, mesosoma, postpetiole and gaster smooth and shiny, except for petiole which is areolate.

Lateral margins and posterior margin of head with suberect hairs. Scapes with abundant subdecumbent hairs. Outer margin of mandibles with decumbent hairs. Mesosoma with short and long suberect hairs. Petiole, and postpetiole with short subdecumbent hairs and long suberect hairs. Tibia with appressed hairs. Gaster with abundant subdecumbent hairs. Color yellowish.

#### Distribution and biology.

*Carebara hainteny* is known only from the center of Madagascar (Figure 68) from grassland, montane rainforest and rainforest and was collected once in montane grassland. The species was sampled at different elevations from 785 m to 1300 m. *C. hainteny* was collected using Winkler traps, and found in rotten logs, leaf mold and rotten wood.

#### Comments.

*Carebara hainteny* is endemic to Madagascar. This species can be easily distinguished from other species by the presence of well-defined, irregular and longitudinal rugoreticulate sculpture on the head; short, triangular, horn-like teeth on the posterolateral corners of the head; workers with a pair of triangular teeth on the propodeum, a thick petiolar node and the gaster with abundant suberect to subdecumbent hairs. Other five species, *C. betsi*, *C. jajoby*, *C. kabosy*, *C. mahafaly*, and *C. nosindambo*, were recorded at the same area, but can be separated from the others by the characters mentioned above.

*C. hainteny* does not have intermediates in the major worker subcaste.

#### Additional material examined.

**MADAGASCAR**: ***Fianarantsoa***: Parc National de Ranomafana, Vatoharanana River, 4.1 km 231° SW Ranomafana, -21.29, 47.43333, 1100 m, montane rainforest, 27–31.iii.2003, (*Fisher, Griswold et al.*); Fianarantsoa, Vohiparara Kidonavo 2, -21.22603, 47.36963, 1100 m, stomach contents of *Mantella madagascariensis*, 13.iii.2003, (*V.C.Clark*); Fianarantsoa, 2 km W Andrambovato, along river Tatamaly, -21.51167, 47.41, 1075 m, montane rainforest, 3–5.vi.2005, (*B.L. Fisher et al*.); Fianarantsoa, 43 km S Ambalavao, Réserve Andringitra, -22.23333, 47, 825 m, rainforest, 5.x.1993, (*B.L. Fisher*); Fianarantsoa, 28 km. SSW Ambositra, Ankazomivady, -20.775, 47.16833, 1670 m, grassland, 11.i.1998, (*B.L. Fisher*); Fianarantsoa, 45 km S. Ambalavao, -22.21667, 47.01667, 785 m, rainforest, 25.ix.1993, (*B.L. Fisher*); ***Toamasina***: Parc National Mantadia, -18.79167, 48.42667, 895 m, rainforest, 25–28.xi.1998, (*H.J.Ratsirarson*); Toamasina, Parc National Mantadia, -18.79167, 48.42667, 895 m, rainforest, 28.xi.-1.xii.1998, (*H.J.Ratsirarson*); Toamasina, Forêt classée Didy, -18.19833, 48.57833, 960 m, rainforest, 16.xii.-23.xii.1998, (*H.J.Ratsirarson*); Toamasina, Analamay, -18.80623, 48.33707, 1068 m, montane rainforest, 21.iii.2004, (*Malagasy ant team*).

### Carebara hiragasy

Taxon classificationAnimaliaHymenopteraFormicidae

Azorsa & Fisher sp. n.

http://zoobank.org/7F0B6310-F0FE-4F30-A9A5-DA8E3D3BE8AB

#### Holotype.

(major worker), MADAGASCAR, Toliara, Forêt Classée d’Analavelona, 29.2 km 343° NNW Mahaboboka, -22.675, 44.19, 1100 m, montane rainforest, 18–22.ii.2003, (*Fisher, Griswold et al.*). Collection code BLF07838, (CASC: CASENT0906501). **Paratypes**: (2 major workers and 6 minor workers), with same data as holotype, 2 major workers (CASC: CASSENT0906495, CASENT0906496), and 1 minor worker (CASC: CASENT0496888). 5 minor workers with same data as holotype but with different collection codes. BLF07820. (BMNH: CASENT0498240), BLF07827, (CASC: CASENT0498241, CASENT0498242), BLF07848, (MCZ: CASENT0498224, MHNG: CASENT0498223).

#### Diagnosis.

Antennae ten-segmented. **Major**: Head nearly subrectangular, posterior margin of head concave and posterolateral corners rounded; head with longitudinal rugae, not reaching posterolateral lobes, smooth and shiny frons, imbricate close to posterior margin of head; propodeum with posterodorsal corner nearly convex, dorsum flat; ventral face of petiole flat and petiolar node thick, combined outline of dorsal surface of peduncle and anterior face of node medially concave and posterior face vertical and convex. **Minor**: Head nearly subquadrate; propodeum with posterodorsal corner nearly convex, and dorsum flat; petiolar node thick, combined outline of dorsal surface of peduncle and anterior face of node slightly concave.

**Figure 42.**
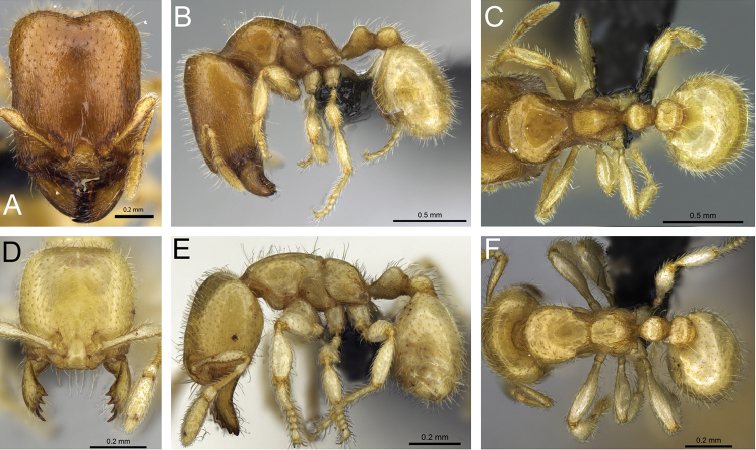
*Carebara hiragasy*–holotype. Major worker, CASENT0496886: **A** head in full-face view **B** body in profile view **C** body in dorsal view. Minor worker, CASENT0496888: **D** head in full-face view **E** body in profile view **F** body in dorsal view.

#### Description of major workers.

**Measurements** (n=3): HL 0.74–1.03 (0.87); HW 0.58–0.79 (0.66); SL 0.31–0.39 (0.35); ML 0.20–0.27 (0.22); EL 0.02–0.05 (0.02); EM 0.21–0.34 (0.30); HD 0.42–0.61 (0.50); WL 0.67–0.88 (0.80); PSL 0.04–0.07 (0.06); PW 0.39–0.53 (0.46); MFL 0.36–0.50 (0.43); MFW 0.08–0.12 (0.11); MTL 0.29–0.39 (0.34); PTL 0.22–0.30 (0.26); PNL 0.12–0.15 (0.13); PTH 0.16–0.21 (0.18); PTW 0.16–0.21 (0.18); PPL 0.16–0.20 (0.17); PPNL 0.12–0.18 (0.15); PPH 0.14–0.21 (0.17); PPW 0.18–0.24 (0.21); GL 0.52–0.75 (0.61); GW 0.47–0.75 (0.61); CI 76–78 (76); MI 25–27 (25); SI 38–42 (40), MLI 62–65 (65); PPLI 65–73 (65); PPI 113–117 (117); PSI 7–9 (9).

Head longer than wide (CI 76–78), in full-face view roughly subrectangular, about 1.3 times longer than wide. Posterior margin of head medially concave, posterolateral corners rounded, lateral margins straight. Mandibles with six teeth. Anterior margin of clypeus slightly concave, and laterally convex. Antennae with ten segments. Scapes short (HL 0.74–1.03, SL 0.31–0.39, SI 38–42), barely surpassing cephalic midlength. Ocelli present or absent. Eyes present, consisting of one ommatidium (EL 0.02–0.05). Supraclypeal area well defined, acutely triangular with a short longitudinal strip posteriorly, not reaching cephalic midlength.

In profile view, promesonotum high and strongly convex, metanotal groove deeply impressed, propodeum lower than promesonotum. Propodeum about as high as long, dorsal face of propodeum nearly flat and declining posteriorly, propodeum unarmed, posterodorsal corner bluntly angled, declivity of propodeum vertical and slightly concave. Propodeal lobes short and convex. Propodeal spiracle rounded and situated above mid-height of sclerite by about 1.5 times the diameter of the spiracle, and beyond mid-length of sclerite by about two times the diameter of the spiracle, distance from propodeal spiracle to posterodrosal corner of propodeum about 1.8 times the diameter of the spiracle (PSL 0.04–0.07), and distance to declivity same as the diameter of the spiracle. In dorsal view, promesonotum about as long as wide, anterior margin and sides of promesonotum rounded, deeply narrowed posteriorly; sides of propodeum straight.

Petiole longer than high (PTL 0.22–0.30, PTH 0.16–0.21) and with relatively short peduncle, ventral face slightly convex. Petiolar node thick. Combined outline of dorsal surface of peduncle and anterior face of node slightly concave in the middle, posterior face of node vertical and weakly convex, anterodorsal corner convex, posterodorsal corner rounded, dorsum convex. Subpetiolar process produced as denticle, about as long as the diameter of the propodeal spiracle. Postpetiolar node rounded and lower than petiolar node. In dorsal view, petiolar node not as wide as postpetiolar node (PTW 0.16–0.21, PPW 0.18–0.24), petiolar node wider than long (PTW 0.16–0.21, PNL 0.12–0.15), anterior and posterior margins of petiole slightly convex, and nearly straight in postpetiole, sides rounded in petiole and postpetiole.

Dorsal surface of mandibles and head with scattered piligerous punctae. Frontoclypeal area, frons, and posterolateral portion of cephalic dorsum smooth and shiny. Head with longitudinal and parallel carinulae, extending from gena and frontal lobes to posterolateral corners, clypeus with transverse rugae. Mandibles with longitudinal striations, weakly imbricate close to posterior margin of head. In profile, posterolateral portion of cephalic dorsum smooth and shiny medially. Mesosoma smooth and shiny, except for katepisternum, metapleuron and propodeum, which are areolate. Longitudinal and fine carinulae present on lateral side of pronotum. Petiole and ventral face of postpetiole areolate. In dorsal view, promesonotum, petiole, postpetiole and gaster smooth and shiny; propodeum weakly sculptured.

Lateral margins, posterolateral corners, and posterior margin of head with short and long erect to suberect hairs. Scapes with appressed hairs. Outer margin of mandible with a few and very short appressed hairs. Mesosoma with short and long erect hairs. Petiole and postpetiole with long suberect and subdecumbent hairs. Tibia with decumbent hairs. Gaster with short subdecumbent hairs and long erect hairs. Color yellowish ferruginous.

#### Description of minor workers.

**Measurements** (n=8): HL 0.43–0.50; HW 0.41–0.45; SL 0.29–0.32; ML 0.12–0.15; EL 0.01–0.02; EM 0.14–0.18; HD 0.27–0.31; WL 0.50–0.57; PSL 0.03–0.04; PW 0.26–0.30; MFL 0.29–0.35; MFW 0.07–0.08; MTL 0.24–0.27; PTL 0.15–0.18; PNL 0.09–0.10; PTH 0.13–0.14; PTW 0.11–0.13; PPL 0.11–0.14; PPNL 0.09–0.11; PPH 0.10–0.12; PPW 0.14–0.17; GL 0.43–0.61; GW 0.36–0.42; CI 90–100; MI 27–31; SI 64–68; MLI 67–78; PPLI 69–81; PPI 119–132; PSI 7–9.

Head slightly longer than wide (CI 90–100), in full-face view nearly subquadrate, about 1.03 times longer than wide. Posterior margin of head nearly straight, posterolateral corners convex, lateral margins convex. Mandibles with five teeth. Anterior margin of clypeus weakly concave medially and laterally angulate. Antennae with ten segments. Scape fails to reach the posterior margin of head (HL 0.43–0.50, SL 0.29–0.32, SI 64–68). Eyes present, consisting of two ommatidia (EL 0.01–0.02). Supraclypeal area triangular but poorly defined.

In profile view, promesonotum weakly convex, nearly flat, metanotal groove deeply impressed. Propodeum about as high as long, dorsal face of propodeum flat and declining posteriorly, posterodorsal corner bluntly angled, anterodorsal corner rounded, declivity vertical and slightly concave. Propodeal lobes short and convex. Propodeal spiracle rounded and situated slightly above mid-height of sclerite by about half the diameter of the spiracle, and beyond mid-length of sclerite by about the diameter of the spiracle, distance from propodeal spiracle to posterodorsal corner of propodeum and to declivity same as the diameter of the spiracle (PSL 0.03–0.04). In dorsal view, promesonotum almost as long as wide (about 1.06 times longer than wide), anterior margin of promesonotum rounded, sides convex; sides of propodeum weakly convex.

Petiole slightly longer than high (PTL 0.15–0.18, PTH 0.13–0.14) and with a short peduncle, ventral face medially convex. Combined outline of dorsal surface of peduncle and anterior face of node slightly concave, nearly straight, posterior face of node vertical and nearly straight, anterodorsal corner convex, posterodorsal corner rounded, dorsum convex. Subpetiolar process produced as a small denticle, smaller than the diameter of the propodeal spiracle. Petiolar node thick. Postpetiolar node rounded and lower than petiolar node. In dorsal view, postpetiolar node wider than petiolar node (PPW 0.14–0.17, PTW 0.11–0.13), petiolar node wider than long (PTW 0.11–0.13, PNL 0.09–0.10), anterior and posterior margin of petiole slightly convex, and nearly straight in postpetiole, sides of petiole rounded and convex in postpetiole.

Dorsal surface of head, mandibles, clypeus and supraclypeal area smooth and shiny, with scattered piligerous punctae on head and mandibles, gena and frontal lobes longitudinally carinulate. Mesosoma smooth and shiny, except for katepisternum (areolate), and metapleuron (finely and longitudinally areolate). Petiole and ventral face of postpetiole areolate. In dorsal view, mesosoma, petiole, postpetiole and gaster smooth and shiny, except for declivity of propodeum which is weakly sculptured.

Lateral margins and posterior margin of head with suberect hairs. Scapes with subdecumbent hairs. Outer margin of mandible with short decumbent hairs. Mesosoma with long and short erect hairs. Petiole, postpetiole and gaster with abundant subdecumbent hairs, and sparse and long suberect hairs. Tibia with decumbent hairs. Color yellowish ferruginous.

#### Distribution and biology.

*Carebara hiragasy* is known only from the southwestern and southeastern sections of Madagascar (Figure 68). This species was found in montane rainforest and rainforest (transition to montane forest). *C. hiragasy* was collected at 600 m and 1100 m, in soil and under stones.

#### Comments.

*Carebara hiragasy* is endemic to two localities in the southwest and southeast of Madagascar. The major workers are easily recognized by the following characters: head longer than wide, with the posterolateral corners rounded; the propodeum unarmed; and the anterodorsal corner of the petiole convex. In minor workers: the head nearly subquadrate; the propodeum unarmed; and long erect to suberect hairs on the head and body, except for those on the scape and tibia which are decumbent. Other nine species were recorded at the same localities: *C. bara*, *C. dota*, *C. grandidieri*, *C. hainteny*, *C. jajoby*, *C. nosindambo*, *C. omasi*, *C. placida* and *C. sampi*, but *C. hiragasy* can be separated from the others by the combination of characters mentioned above.

*C. hiragasy* does not have intermediates in the major worker subcaste.

#### Additional material examined.

**MADAGASCAR**: ***Fianarantsoa***: Forêt de Vevembe, 66.6 km 293° Farafangana, -22.791, 47.18183, 600 m, rainforest, transition to montane forest, 23.iv.2006, (*B.L. Fisher et al*.).

### Carebara jajoby

Taxon classificationAnimaliaHymenopteraFormicidae

Azorsa & Fisher sp. n.

http://zoobank.org/38FE8252-FF94-4791-B4C6-BF07DA2D78D5

#### Holotype.

(major worker), MADAGASCAR, Toamasina, Montagne d’Anjanaharibe, 18.0 km 21° NNE Ambinanitelo, -15.18833, 49.615, 470 m, rainforest, 8–12.iii.2003, (*Fisher, Griswold et al.*). Collection code BLF08002, (CASC: CASENT0037505). **Paratypes**: (45 major workers and 23 minor workers), with same data as holotype, 26 major workers (BMNH: CASENT0037498, CASC: CASENT0037566, CASENT0037542, CASENT0037486, CASENT0037496, CASENT0037522, CASENT0037538, CASENT0037532, CASENT0037536, CASENT0037540, CASENT0037555, CASENT0037495, CASENT0037476, CASENT0037518, CASENT0037544, CASENT0037547, CASENT0037554, CASENT0037483, CASENT0037560, CASENT0037558, CASENT0037551, CASENT0037550, CASENT0037562, MCZ: CASENT0037533, MHNG: CASENT0037524, NHMB: CASENT0037521), and 23 minor workers (BMNH: CASENT0037467, CASC: CASENT0037549, CASENT0037479, CASENT0037568, CASENT0037563, CASENT0037552, CASENT0037484, CASENT0037503, CASENT0037525, CASENT0037501, CASENT0037565, CASENT0037535, CASENT0037556, CASENT0037543, CASENT0037534, CASENT0037528, CASENT0037515, CASENT0037557, CASENT0037553, CASENT0037497, MCZ: CASENT0037499, MHNG: CASENT0037512, NHMB: CASENT0037559). With same data as holotype but with different collection codes, 4 major workers, BLF08054, (CASC: CASENT0495342, CASENT0495343), BLF08128, (CASC: CASENT0495466, CASENT0495467). 1 major worker, BLF08110, (CASC: CASENT0495483), 2 major workers, BLF08025, (CASC: CASENT0495307, CASENT0495306), and 2 major workers, BLF08070, (CASC: CASENT0495243, CASENT0495244).

#### Diagnosis.

Antennae ten or eleven-segmented (only larger major workers, minor workers always with ten segments). **Major**: Head longer than wide, slightly narrowed anteriorly and on the top, posterior margin of head deeply concave, posterolateral corners well developed and narrowed posteriorly; posterodorsal corner of propodeum with a pair of triangular teeth, dorsum flat and declining posteriorly; petiolar node thin, anterodorsal and posterodorsal corner rounded, dorsum weakly convex, combined outline of dorsal surface of peduncle and anterior face of node concave to nearly straight, posterior face vertical and nearly straight. **Minor**: Head slightly longer than wide; posterodorsal corner of propodeum with a pair of triangular teeth, dorsum flat and declining posteriorly; petiolar node thin with the top bluntly prominent, combined outline of dorsal surface of peduncle and anterior face of node weakly concave, posterior face vertical and almost straight; gaster with abundant subdecumbent hairs.

**Figure 43.**
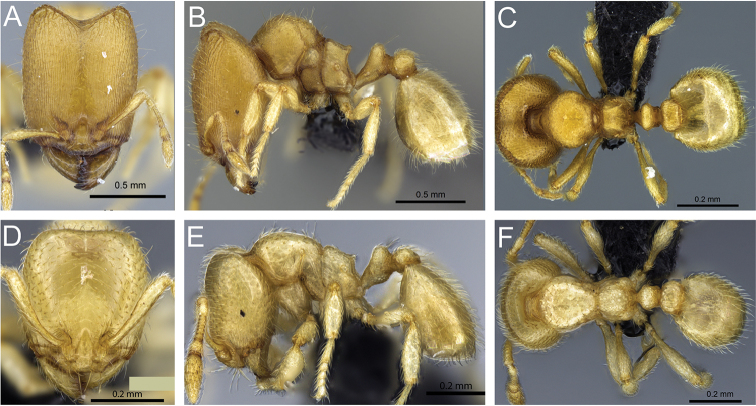
*Carebara jajoby*–holotype. Major worker, CASENT0037505: **A** head in full-face view **B** body in profile view **C** body in dorsal view. Minor worker, CASENT0037501: **D** head in full-face view **E** body in profile view **F** body in dorsal view.

**Figure 44.**
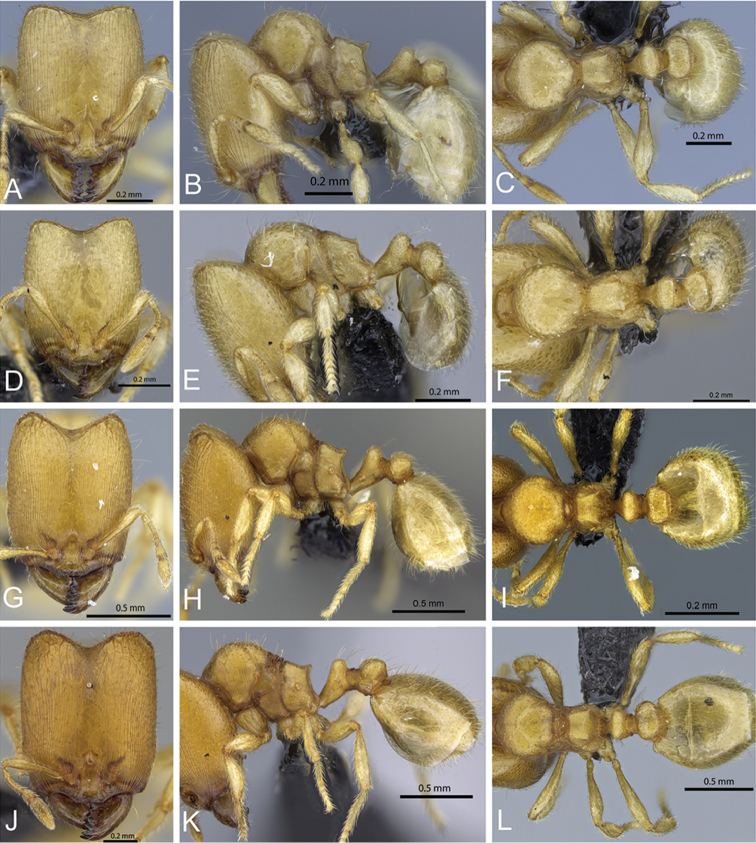
Intermediates of *Carebara jajoby*. Major workers, CASENT0496387: **A** head in full-face view **B** body in profile view **C** body in dorsal view. CASENT0068887: **D** head in full-face view **E** body in profile view **F** body in dorsal view. CASENT0037505: **G** head in full-face view **H** body in profile view **I** body in dorsal view. CASENT0070133: **J** head in full-face view **K** body in profile view **L** body in dorsal view.

#### Description of major workers.

**Measurements** (n=27): HL 0.62–1.18 (0.97); HW 0.51–0.92 (0.75); SL 0.27–0.45 (0.38); ML 0.14–0.31 (0.26); EL 0.01–0.14 (0.04); EM 0.16–0.30 (0.27); HD 0.33–0.70 (0.55); WL 0.53–1.12 (0.80); PSL 0.06–0.18 (0.15); PW 0.31–0.60 (0.48); MFL 0.32–0.63 (0.47); MFW 0.07–0.15 (0.11); MTL 0.26–0.51 (0.39); PTL 0.21–0.43 (0.36); PNL 0.08–0.15 (0.12); PTH 0.16–0.27 (0.24); PTW 0.14–0.27 (0.23); PPL 0.12–0.22 (0.18); PPNL 0.11–0.21 (0.15); PPH 0.12–0.26 (0.20); PPW 0.18–0.33 (0.27); GL 0.40–1.54 (0.66); GW 0.37–0.92 (0.66); CI 72–87 (77); MI 22–29 (27); SI 34–45 (39); MLI 58–70 (63); PPLI 45–62 (50); PPI 116–134 (117); PSI 11–26 (20).

Head longer than wide (CI 72–87), in full-face view roughly subrectangular, about 1.3 times longer than wide, narrowed anteriorly and on the top. Posterior margin of head deeply concave, V-shaped, posterolateral corners well developed and narrowed posteriorly, lateral margins moderately convex. Mandibles with six or seven teeth. Anterior margin of clypeus straight to slightly concave, and laterally angulate. Antennae with ten or eleven segments. Scapes short (HL 0.62–1.18, SL 0.27–0.45, SI 34–45). Ocelli absent or present. Eyes present, consisting of one to 50 ommatidia (EL 0.01–0.14). Supraclypeal area mostly triangular, apex varies from acute on small and medium major workers, to rounded on larger major workers.

In profile view, posterolateral corner of head with a small, obtuse tooth resembling a horn. Promesonotum high and nearly rounded, metanotal groove deeply impressed. Propodeum lower than promesonotum. Propodeum about 1.75 times higher than long, dorsal face of propodeum nearly flat, to weakly concave, declining posteriorly; posterodorsal corners of propodeum each armed with a triangular tooth; anterodorsal corner convex, declivity vertical and concave with narrow lateral laminae. Propodeal lobes short and convex. Propodeal spiracle rounded and situated above mid-height of sclerite, and beyond mid-length of sclerite by about half the diameter of the spiracle, distance from propodeal spiracle to posterodorsal corner of propodeum about four times the diameter of the spiracle (PSL 0.06–0.18), and distance to declivity almost twice the diameter of the spiracle. In dorsal view, promesonotum about as long as wide, anterior margin and sides of promesonotum rounded; sides of propodeum straight.

Petiole longer than high (PTL 0.21–0.43, PTH 0.16–0.27) and with relatively long peduncle, ventral face weakly convex in the middle. Combined outline of dorsal surface of peduncle and anterior face of node concave or nearly straight, posterior face of node vertical, anterodorsal corner convex, posterodorsal corner rounded, dorsum convex. Subpetiolar process produced as a very small denticle, same as or slightly shorter than the diameter of the propodeal spiracle. Postpetiolar node rounded and lower than petiolar node. In dorsal view, petiolar node not as broad as postpetiolar node (PPW 0.18–0.33, PTW 0.14–0.27) and petiolar node wider than long (PTW 0.14–0.27, PNL 0.08–0.15), anterior margins of petiole and postpetiole nearly straight, sides of petiole rounded, convex in postpetiole, narrowed posteriorly.

Dorsal surface of mandibles, clypeus, supraclypeal area and frons smooth and shiny, with scattered piligerous punctae on head and mandibles. Head with longitudinal rugae directed to posterior margin, gena with well-developed longitudinal rugae directed to posterolateral corners of head. In profile, posterolateral portion of cephalic dorsum smooth and shiny, larger specimens with weakly marked longitudinal rugae. Sides of pronotum with transverse and longitudinal fine rugae, median area unsculptured on smaller major workers; katepisternum areolate, metapleuron finely areolate. Petiole and ventral face of postpetiole areolate. In dorsal view, mesosoma, petiole, postpetiole and gaster smooth and shiny; propodeum weakly sculptured.

Lateral margins with long suberect hairs and short appressed hairs. Scapes with appressed hairs. Outer margin of mandible with short and sparse appressed hairs. Mesosoma with short decumbent to appressed hairs, and long suberect or subdecumbent hairs. Petiole and postpetiole with short decumbent or appressed hairs and long subdecumbent hairs. Tibia with appressed hairs. Gaster with short decumbent hairs and long subdecumbent hairs. Color yellowish ferruginous.

#### Description of minor workers.

**Measurements** (n=11): HL 0.37–0.45; HW 0.34–0.40; SL 0.24–0.28; ML 0.09–0.12; EL 0.01–0.02; EM 0.11–0.14; HD 0.21–0.28; WL 0.39–0.47; PSL 0.03–0.07; PW 0.21–0.25; MFL 0.25–0.31; MFW 0.06–0.08; MTL 0.18–0.25, PTL 0.15–0.19; PNL0.06–0.09; PTH 0.11–0.13; PTW 0.09–0.11; PPL 0.09–0.10; PPNL 0.08–0.10; PPH 0.09–0.10; PPW 0.12–0.15; GL 0.28–0.40; GW 0.26–0.34; CI 84–93; MI 22–28; SI 60–65, MLI 72–82; PPLI 50–67; PPI 127–156; PSI 9–18.

Head longer than wide (CI 84–93), in full-face view nearly subquadrate, about 1.1 times longer than wide, weakly narrowed anteriorly. Posterior margin of head slightly concave, posterolateral corners rounded, lateral margins convex. Mandibles with six teeth. Anterior margin of clypeus nearly straight, and laterally angulate. Antennae with ten segments. Scape fails to reach the posterior margin of head (HL 0.37–0.45, SL 0.24–0.28, SI 60–65). Eyes present, consisting of one ommatidium (EL 0.01–0.02). Supraclypeal area almost triangular but poorly defined.

In profile view, promesonotum weakly convex, nearly flat, metanotal groove deeply impressed. Propodeum about 1.58 times higher than long, dorsal face of propodeum flat, declining posteriorly, anterodorsal corner rounded, posterodorsal corners each armed with a triangular tooth, declivity vertical and nearly flat, with thin lateral laminae. Propodeal lobes short and convex. Propodeal spiracle nearly oval and situated above mid-height of sclerite by about half the diameter of the spiracle, and beyond mid-length of sclerite by about 1.5 times the diameter of the spiracle, distance from propodeal spiracle to posterodorsal corner of propodeum about 2.5 times the diameter of the spiracle (PSL 0.03–0.07), and distance to declivity same as the diameter of the spiracle. In dorsal view, promesonotum about 1.16 times longer than wide, anterior margin rounded, sides convex and narrowed posteriorly; sides of propodeum weakly convex.

Petiole longer than high (PTL 0.15–0.19, PTH 0.11–0.13) and with relatively short peduncle, ventral face medially convex. Combined outline of dorsal surface of peduncle and anterior face of node weakly concave, posterior face of node vertical and slightly concave, anterodorsal and posterodorsal corners rounded, dorsum convex. Subpetiolar process weakly present as denticle, shorter than the diameter of the propodeal spiracle. Postpetiolar node nearly rounded and lower than petiolar node. In dorsal view, postpetiolar node wider than petiolar node (PPW 0.12–0.15, PTW 0.09–0.11), petiolar node wider than long (PTW 0.09–0.11, PNL 0.06–0.09), anterior and posterior margins of petiole and postpetiole nearly straight, lateral margins rounded in petiole and postpetiole.

Dorsal surface of head, mandibles, clypeus and supraclypeal area smooth and shiny, with scattered piligerous punctae on head and mandibles. Gena and frontal lobes with longitudinal rugae. Mesosoma smooth and shiny, except for katepisternum (mesopleuron) and metapleuron (finely areolate-rugose). Petiole and ventral margin of postpetiole areolate. In dorsal view, promesonotum, postpetiole and gaster smooth and shiny; propodeum and petiole finely areolate.

Lateral margins and posterior margin of head with short decumbent hairs, and long suberect hairs. Scapes with abundant decumbent hairs. Outer margin of mandible with decumbent hairs. Mesosoma with short decumbent hairs and long suberect or subdecumbent hairs. Petiole and postpetiole with short decumbent hairs and longer subdecumbent hairs. Tibia with appressed hairs. Gaster with abundant decumbent hairs and long subdecumbent hairs. Color yellowish ferruginous.

#### Distribution and biology.

*Carebara jajoby* is known from the east and northwest of Madagascar (Figure 68). This species was mainly found in littoral rainforest, montane rainforest, montane shrubland, rainforest, and rainforest (transition to montane forest). *C. jajoby* is distributed in elevations ranging from 10 m to 2000 m. Specimens were collected using maxi-Winkler traps. Individuals and colonies were found in the following microhabitats: dead twigs above ground, rotten logs, rotten logs in root mat, ground nests, sifted litter, leaf mold, rotten wood, under rootmat, litter on rocks, and under stones.

#### Comments.

*Carebara jajoby* can be confused with the following species: *C. sampi*, *C. vazimba*, and *C. kabosy*, but can be separated from them by the following combination of characters. In major workers, dorsum of head of *C. sampi* is smooth and shiny or with fine longitudinal rugae laterally. In case the dorsum of head presents well-defined longitudinal rugae like *C. vazimba*, *C. kabosy*, and *C. jajoby* then the propodeal tooth of *C. jajoby* is longer than the diameter of the propodeal spiracle. In minor workers, *C. jajoby* can be separated from the others species by the prescence of abundant subdecumbent hairs in the gaster, and with fewer than ten longer suberect hairs. *C. jajoby* is distributed from the east and northwest of Madagascar, while *C. sampi* is distributed from south and southwestern, *C. vazimba* is distributed in the north, northeastern and northwestern. Other nine species were recorded at the same localities: *C. bara*, *C. betsi*, *C. grandidieri*, *C. hainteny*, *C. hiragasy*, *C. kabosy*, *C. mahafaly*, *C. nosindambo*, and *C. placida*.

*C. jajoby* have four intermediates in the major worker subcaste (Figure 44). The posterolateral corners of head of intermediates narrow posteriorly and increase in size from intermediate 1 to 4. The posterior margin of the head in intermediates 1 and 2 is less concave than intermediates 3 and 4. The lateral margins of the head are convex in all intermediates, with intermediate 4 having lateral margins nearly straight. The eyes are small and reduced to one ommatidium in intermediates 1, 2 and 3, while between 2 and 30 ommatidia are found in intermediate 4. Ocelli are absent in intermediates 1, 2 and 3, but one ocellus is present and well developed in intermediate 4. Reduced flight sclerites are present in intermediate 4. The dorsum of the mesosoma is convex anteriorly and gradually slopes to the declivity in intermediate 4. The dorsum of the promesonotum is high and nearly rounded in intermediates 1, 2 and 3. The shape of the petiole and postpetiole do not vary much in intermediates, with the exception of intermediate 4, where the combined outline of dorsal surface of peduncle and anterior face of node is nearly straight. The propodeum is armed with a pair of triangular teeth in all intermediates. The head is smooth and shiny in the frontal area and with finely longitudinal and parallel rugae in full-face view in all intermediates. Pilosity on head and body follows the same pattern in all intermediates, except for intermediate 4, which has abundant and very short appressed hairs on the gaster. Larger major workers with eleven antennal segments, and seven teeth.

#### Additional material examined.

**MADAGASCAR**: ***Antsiranana***: Parc National de Marojejy, 25.4 km 30° NNE Andapa, 10.9 km 311° NW Manantenina, -14.445, 49.735, 2000 m, montane shrubland, 23.xi.2003, (*B.L. Fisher*); Antsiranana, Parc National de Marojejy, Manantenina River, 27.6 km 35° NE Andapa, 9.6 km 327° NNW Manantenina, -14.435, 49.76, 775 m, rainforest, 15–18.xi.2003, (*B.L. Fisher et al*.); Antsiranana, Parc National de Marojejy, Manantenina River, 27.6 km 35° NE Andapa, 9.6 km 327° NNW Manantenina, -14.435, 49.76, 775 m, rainforest, 11.xii.2005, (*B.L. Fisher et al*.); Antsiranana, 6.5 km SSW Befingotra, Réserve Anjanaharibe-Sud, -14.75, 49.5, 875 m, rainforest, 21.x.1994, (*B.L. Fisher*); Antsiranana, Réserve Spéciale Manongarivo, 12.8 km 228° SW Antanambao, -13.97667, 48.42333, 780 m, rainforest, 11.x.1998, (*B.L. Fisher*); Antsiranana, Réserve Spéciale Manongarivo, 10.8 km 229° SW Antanambao, -13.96167, 48.43333, 400 m, rainforest, 8.xii.1998, (*B.L. Fisher*); Antsiranana, Réserve Spéciale Manongarivo, 12.8 km 228° SW Antanambao, -13.97667, 48.42333, 780 m, rainforest, 11–17.x.1998, (*B.L. Fisher*); Antsiranana, Réserve Spéciale Manongarivo, 14.5 km 220° SW Antanambao, -13.99833, 48.42833, 1175 m, montane rainforest, 20.x.1998, (*B.L. Fisher*); Antsiranana, Makirovana forest, -14.17066, 49.95409, 225 m, rainforest, 4–6.v.2011, (*B.L. Fisher et al*.); Antsiranana, Makirovana forest, -14.16666, 49.95, 715 m, rainforest, 2.v.2011, (*B.L. Fisher et al*.); Antsiranana, Makirovana forest, -14.16044, 49.95216, 550 m, rainforest, 1–2.v.2011, (*B.L. Fisher et al*.); Antsiranana, Makirovana forest, -14.16666, 49.95, 715 m, rainforest, 1–2.v.2011, (*B.L. Fisher et al*.); Antsiranana, Makirovana forest, -14.16506, 49.9477, 900 m, montane rainforest, 30.iv.-1.v.2011, (*B.L. Fisher et al*.); Antsiranana, Makirovana forest, -14.16044, 49.95216, 550 m, rainforest, 1.v.2011, (*B.L. Fisher et al*.); Antsiranana, Makirovana forest, -14.17066, 49.95409, 415 m, rainforest, 28–29.iv.2011, (*B.L. Fisher et al*.); Antsiranana, Réserve Spéciale Manongarivo, 10.8 km 229° SW Antanambao, -13.96167, 48.43333, 400 m, rainforest, 8.xi.1998, (*B.L. Fisher*); Antsiranana, Galoko chain, Mont Galoko, -13.58487, 48.71818, 520 m, rainforest, 19.ii.2013, (*B.L. Fisher et al*.); Antsiranana, Galoko chain, Mont Galoko, -13.58487, 48.71818, 520 m, rainforest, 17.ii.2013, (*B.L. Fisher et al*.); Antsiranana, Galoko chain, Mont Galoko, -13.59358, 48.73157, 1100 m, montane forest, 22.ii.2013, (*B.L. Fisher et al*.); Antsiranana, Parc National de Marojejy, Manantenina River, 27.6 km 35° NE Andapa, 9.6 km 327° NNW Manantenina, -14.435, 49.76, 775 m, rainforest, 16.xi.2003, (*B.L. Fisher*); ***Fianarantsoa***: Forêt de Vevembe, 66.6 km 293° Farafangana, -22.791, 47.18183, 600 m, rainforest, transition to montane forest, 24.iv.2006, (*B.L. Fisher et al*.); Fianarantsoa, Forêt de Vevembe, 66.6 km 293° Farafangana, -22.791, 47.18183, 600 m, rainforest, transition to montane forest, 23.iv.2006, (*B.L. Fisher et al*.); ***Toamasina***: Montagne d’Akirindro 7.6 km 341° NNW Ambinanitelo, -15.28833, 49.54833, 600 m, rainforest, 17–21.iii.2003, (*Fisher, Griswold et al.*); Toamasina, Forêt Ambatovy, 14.3 km 57° Moramanga, -18.85083, 48.32, 1075 m, montane rainforest, 21.iii.2004, (*Malagasy ant team*); Toamasina, Torotorofotsy, -18.87082, 48.34737, 1070 m, montane rainforest, marsh edge, 24.iii.2004, (*Malagasy ant team*); Toamasina, Forêt Ambatovy, 14.3 km 57° Moramanga, -18.85083, 48.32, 1075 m, montane rainforest, 22.iii.2004, (*Malagasy ant team*); Toamasina, Forêt Ambatovy, 14.3 km 57° Moramanga, -18.85083, 48.32, 1075 m, montane rainforest, 18.xii.2004, (*B.L. Fisher*); Toamasina, Réserve Ambodiriana, 4.8 km 306° Manompana, along Manompana river, -16.67233, 49.70117, 125 m, rainforest, 19.xi.2005, (*B.L. Fisher et al*.); Toamasina, Parc National Mananara-Nord, 7.1 km 261° Antanambe, -16.455, 49.7875, 225 m, rainforest, 14.xi.2005, (*B.L. Fisher et al*.); Toamasina, Ile Sainte Marie, Forêt Ambohidena, 22.8 km 44° Ambodifotatra, -16.82433, 49.96417, 20 m, littoral rainforest, 21.xi.2005, (*B.L. Fisher et al*.); Toamasina, Réserve Ambodiriana, 4.8 km 306° Manompana, along Manompana river, -16.67233, 49.70117, 125 m, rainforest, 18.xi.2005, (*B.L. Fisher et al*.); Toamasina, Réserve Analamazaotra, Parc National, Andasibe, -18.92778, 48.41833, 947 m, 7.i.2006, (*A.Ballerio*); Toamasina, Ambatovy, 12.4 km NE Moramanga, -18.84963, 48.2947, 1010 m, montane rainforest, 3–6.iii.2007, (*B.L. Fisher et al*.); Toamasina, Ambatovy, 12.4 km NE Moramanga, -18.83937, 48.30842, 1080 m, montane rainforest, 4–7.iii.2007, (*B.L. Fisher et al*.); Toamasina, 6.3 km S Ambanizana, Andranobe, -15.6813, 49.958, 25 m, rainforest, 23.xi.1993, (*B.L. Fisher*); Toamasina, 6.3 km S Ambanizana, Andranobe, -15.6813, 49.958, 25 m, rainforest, 14.xi.1993, (*B.L. Fisher*); Toamasina, 6.9 km NE Ambanizana, Ambohitsitondroina, -15.58506, 50.00952, 825 m, rainforest, 2.xii.1993, (*B.L. Fisher*); Toamasina, Forêt classée Andriantantely, -18.695, 48.81333, 530 m, rainforest, 4–7.xii.1998, (*H.J.Ratsirarson*); Toamasina, Forêt classée Sandranantitra, -18.04833, 49.09167, 450 m, rainforest, 18–21.i.1999, (*H.J.Ratsirarson*); Toamasina, Forêt classée Didy, -18.19833, 48.57833, 960 m, rainforest, 16–23.xii.1998, (*H.J.Ratsirarson*); Toamasina, Forêt classée Andriantantely, -18.695, 48.81333, 530 m, rainforest, 7–10.xii.1998, (*H.J.Ratsirarson*); Toamasina, Station forestière Analamazaotra, Analamazaotra 1.3km S Andasibe, -18.38466, 48.41271, 980 m, montane rainforest, (B.L. Fisher et al.); Toamasina, Corridor Forestier Analamay-Mantadia, Ambohibolakely, -18.77898, 48.36375, 918 m, rainforest, 23–28.xi.2012, (*B.L. Fisher et al*.); Toamasina, Ankerana, -18.4104, 48.8189, 855 m, rainforest, 22–27.i.2012, (*B.L. Fisher et al*.); Toamasina, Corridor Forestier Analamay-Mantadia, Ambatoharanana, -18.80388, 48.40506, 1013 m, rainforest, 12–19.xii.2012, (*B.L. Fisher et al*.); Toamasina, Corridor Forestier Analamay-Mantadia, Ambohibolakely, -18.76087, 48.37128, 1044 m, rainforest, 29.xi.2012, (*B.L. Fisher et al*.); Toamasina, Corridor Forestier Analamay-Mantadia, Ambohibolakely, -18.76131, 48.36437, 983 m, rainforest, 23.xi.2012, (*B.L. Fisher et al*.); Toamasina, Corridor Forestier Analamay-Mantadia, Ambohibolakely, -18.76131, 48.36437, 983 m, rainforest, 26.xi.2012, (*B.L. Fisher et al*.); Toamasina, Corridor Forestier Analamay-Mantadia, Tsaravoniana, -18.76465, 48.41938, 1039 m, rainforest, 4–5.xii.2012, (*B.L. Fisher et al*.); Toamasina, Corridor Forestier Analamay-Mantadia, Tsaravoniana, -18.76369, 48.4203, 984 m, rainforest, 2–7.xii.2012, (*B.L. Fisher et al*.); Toamasina, Corridor Forestier Analamay-Mantadia, Ambatoharanana, -18.80398, 48.40358, 1064 m, rainforest, 12–19.xii.2012, (B.L. Fisher et al.); Toamasina, Corridor Forestier Analamay-Mantadia, Tsaravoniana, -18.76465, 48.41938, 1039 m, rainforest, 2–7.xii.2012, (*B.L. Fisher et al*.); Toamasina, Corridor Forestier Analamay-Mantadia, Ambohibolakely, -18.76131, 48.36437, 983 m, rainforest, 23–28.xi.2012, (*B.L. Fisher et al*.); ***Toliara***: Forêt de Petriky, 12.5 km W 272° Tolagnaro, -25.06167, 46.87, 10 m, littoral rainforest, 22.xi.1998, (*B.L. Fisher*).

### Carebara kabosy

Taxon classificationAnimaliaHymenopteraFormicidae

Azorsa & Fisher sp. n.

http://zoobank.org/0FF5AEB6-5AB7-4370-9E62-962AAE1F28E2

#### Holotype.

(major worker), MADAGASCAR, Antsiranana, Parc National Montagne d’Ambre, 3.6 km 235° SW Joffreville, -12.53444, 49.1795, 925 m, montane rainforest, 20–26.i.2001, (*Fisher, Griswold et al.*). Collection code BLF02564, (CASC: CASENT0438062). **Paratypes**: (35 major workers and 17 minor workers), with same data as holotype, 34 major workers (BMNH: CASENT0438053, CASC: CASENT0437873, CASENT0437718, CASENT0437914, CASENT0437996, CASENT0438102, CASENT0437701, CASENT0437890, CASENT0437950, CASENT0438160, CASENT0437677, CASENT0437753, CASENT0437723, CASENT0437772, CASENT0437702, CASENT0437694, CASENT0437692, CASENT0437948, CASENT0437989, CASENT0437956, CASENT0437793, CASENT0438100, CASENT0437999, CASENT0437787, CASENT0437997, CASENT0438015, CASENT0437988, CASENT0437676, CASENT0437955, CASENT0437691, CASENT0437933, MCZ: CASENT0437910, MHNG: CASENT0437953, NHMB: CASENT0437958), and 17 minor workers (BMNH: CASENT0437746, CASC: CASENT0437751, CASENT0437877, CASENT0437886, CASENT0437888, CASENT0437887, CASENT0437739, CASENT0437747, CASENT0437889, CASENT0437891, CASENT0437884, CASENT0437904, CASENT0437895, CASENT0437728, MCZ: CASENT0437880, MHNG: CASENT0437731, NHMB: CASENT0437892), 1 major worker with same data as holotype but with collection code BLF02565, (CASC: CASENT0439968).

#### Diagnosis.

Antennae ten-segmented. **Major**: Head longer than wide, lateral margins convex to nearly straight, posterolateral corners well developed and narrowed posteriorly, posterior margin of head deeply concave in the middle; dorsum of propodeum flat, combined outline of dorsal surface of peduncle and anterior face of node concave to nearly straight in larger major workers, posterior face vertical and nearly straight; gaster with short decumbent hairs and longer subdecumbent hairs. **Minor**: Head longer than wide, posterior margin of head concave; dorsum of propodeum nearly flat, anterodorsal corner slightly convex, posterodorsal corner with a pair of small triangular teeth; combined outline of dorsal surface of peduncle and anterior face of node concave, posterior face weakly convex.

**Figure 45.**
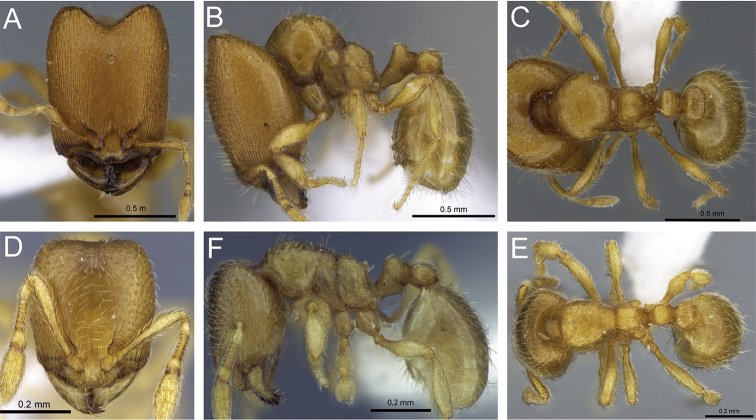
*Carebara kabosy*–holotype. Major worker, CASENT0438062: **A** head in full-face view **B** body in profile view **C** body in dorsal view. Minor worker: CASENT0437739 **D** head in full-face view **E** body in profile view **F** body in dorsal view.

**Figure 46.**
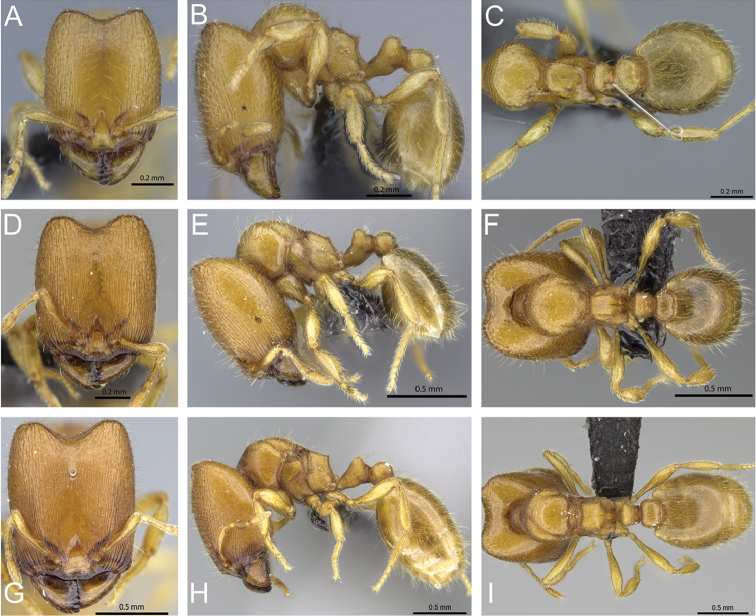
Intermediates of *Carebara kabosy*. Major workers, CASENT0043272: **A** head in full-face view **B** body in profile view **C** body in dorsal view. CASENT0134199: **D** head in full-face view **E** body in profile view **F** body in dorsal view. CASENT0162282: **G** head in full-face view **H** body in profile view **I** body in dorsal view.

#### Description of major workers.

**Measurements** (n=31): HL 0.63–1.16 (0.97); HW 0.49–0.91 (0.74); SL 0.25–0.41 (0.34); ML 0.14–0.27 (0.21); EL 0.01–0.09 (0.03); EM 0.17–0.29 (0.27); HD 0.35–0.69 (0.58); WL 0.52–0.96 (0.73); PSL 0.06–0.14 (0.09); PW 0.29–0.58 (0.46); MFL 0.28–0.58 (0.44); MFW 0.07–0.14 (0.10); MTL 0.21–0.46 (0.34); PTL 0.19–0.36 (0.30); PNL 0.09–0.15 (0.12); PTH 0.15–0.28 (0.21); PTW 0.13–0.25 (0.20); PPL 0.12–0.23 (0.18); PPNL 0.11–0.21 (0.16); PPH 0.12–0.24 (0.18); PPW 0.17–0.32 (0.24); GL 0.45–1.36 (0.70); GW 0.37–0.87 (0.63); CI 75–84 (76); MI 22–26 (22); SI 29–43 (35); MLI 57–67 (59); PPLI 53–71 (60); PPI 117–143 (120); PSI 11–16 (12).

Head longer than wide (CI 75–84), in full-face view nearly subrectangular, about 1.2 times longer than wide. Posterior margin of head deeply concave in the middle, posterolateral corners well developed, rounded and narrowed posteriorly, lateral margins convex to nearly straight. Mandibles with six teeth. Anterior margin of clypeus concave, and laterally convex. Ocelli absent or present. Antennae with ten segments. Scapes short (HL 0.63–1.16, SL 0.25–0.41, SI 29–43). Eyes present, consisting of one or more ommatidia (EL 0.01–0.09). Supraclypeal area short, triangular and well defined.

In profile view, posterolateral corner of head with a small angulate tooth. Promesonotum high and nearly rounded, metanotal groove deeply impressed. Propodeum lower than promesonotum, and about 15 times higher than long, dorsal face of propodeum flat and declining posteriorly, posterodorsal corners each armed with a triangular tooth, declivity of propodeum concave with thin lateral laminae. Propodeal lobes short and convex. Propodeal spiracle rounded and situated above mid-height of sclerite by about half the diameter of the spiracle, and beyond mid-length of sclerite by about the diameter of the spiracle, distance from propodeal spiracle to posterodorsal corner of propodeum about four times the diameter of the spiracle (PSL 0.06–0.14), and distance to declivity about 1.5 times the diameter of the spiracle. In dorsal view, anterior margin and sides of promesonotum rounded, and about as long as wide; sides of propodeum straight.

Petiole longer than high (PTL 0.19–0.36, PTH 0.15–0.28) and with relatively short peduncle, ventral face medially convex. Combined outline of dorsal surface of peduncle and anterior face of node medially concave, posterior face of node vertical and nearly straight, anterodorsal and posterodorsal corners rounded, dorsum nearly rounded. Subpetiolar process produced as small denticle, almost as large as the diameter of the propodeal spiracle. Postpetiolar node rounded and slightly lower than petiolar node. In dorsal view, postpetiolar node slightly wider than petiolar node (PTW 0.13–0.25, PPW 0.17–0.32) and petiolar node wider than long (PNL 0.09–0.15, PTW 0.13–0.25), anterior and posterior margins of petiole straight, anterior margin of postpetiole straight, and posterior margin convex, sides rounded in petiole, and postpetiole.

Dorsal surface of mandibles, clypeus, supraclypeal area and frons smooth and shiny, with scattered piligerous punctae on head and mandibles. Head with longitudinal and parallel rugae directed to posterior margin of head, gena with longitudinal rugae extending to posterolateral corners. Posterolateral portion of cephalic dorsum smooth and shiny. Sides of pronotum finely areolate, median area smooth and shiny on small and median major workers, areolate katepisternum, metapleuron with longitudinal rugae and finely areolate. Anterior margin of pronotum finely areolate. Petiole, ventral and lateral margins of postpetiole areolate. In dorsal view, promesonotum, and postpetiole smooth and shiny medially, propodeum and petiole finely areolate.

Lateral margins of head with short decumbent hairs and long suberect hairs. Posterior margin of head with short and long suberect hairs. Scapes with appressed hairs. Outer margin of mandibles with short appressed hairs. Mesosoma with short and long suberect hairs. Petiole with long subdecumbent hairs, postpetiole with short appressed hairs and long decumbent hairs. Tibia with appressed hairs. Gaster with short decumbent or appressed hairs and long suberect or sudecumbent hairs. Color yellowish ferruginous.

#### Description of minor workers.

**Measurements** (n=29): HL 0.35–0.44; HW 0.31–0.39; SL 0.22–0.28; ML 0.09–0.12; EL 0.01–0.02; EM 0.10–0.13; HD 0.22–0.28; WL 0.35–0.45; PSL 0.04–0.06; PW 0.18–0.24; MFL 0.22–0.29; MFW 0.05–0.07; MTL 0.12–0.22; PTL 0.12–0.20; PNL 0.07–0.08; PTH 0.10–0.13; PTW 0.08–0.11; PPL 0.08–0.11; PPNL 0.07–0.10; PPH 0.07–0.10; PPW 0.11–0.15; GL 0.31–0.47; GW 0.23–0.36; CI 84–95; MI 23–28; SI 61–68; MLI 64–80; PPLI 50–73; PPI 118–150; PSI 11–17.

Head longer than wide (CI 84–95), in full-face view nearly subrectangular, about 1.2 times longer than wide. Posterior margin of head slightly concave in the middle, posterolateral corners rounded, lateral margins slightly convex. Mandibles with five teeth. Anterior margin of clypeus almost straight, and laterally angulate. Antennae with ten segments. Scape fails to reach the posterior margin of head (HL 0.35–0.44, SL 0.22–0.28, SI 61–68). Eyes present, consisting of one ommatidium (EL 0.01–0.02). Supraclypeal area triangular but not well defined.

In profile view, promesonotum weakly convex, nearly flat, metanotal groove deeply impressed. Propodeum about 1.4 times higher than long, dorsal face of propodeum flat and declining posteriorly, anterodorsal corner of propodeum weakly convex, posterodorsal corners each armed with a small triangular tooth, declivity weakly concave with thin lateral laminae. Propodeal lobes short and convex. Propodeal spiracle nearly oval and situated weakly above mid-height of sclerite by about half the diameter of the spiracle, and beyond mid-length of sclerite by about 1.5 times the diameter of the spiracle, distance from propodeal spiracle to posterodorsal corner of propodeum about 2.5 times the diameter of the spiracle (PSL 0.04–0.06), and distance to declivity almost the same as the diameter of the spiracle. In dorsal view, anterior margin of promesonotum convex, sides convex and narrowed posteriorly, slightly longer than wide; sides of propodeum straight.

Petiole longer than high (PTL 0.12–0.20, PTH 0.10–0.13) and with a short peduncle, ventral face medially convex. Combined outline of dorsal surface of peduncle and anterior face of node medially concave, posterior face weakly convex, anterodorsal and posterodorsal corner rounded, dorsum nearly rounded. Subpetiolar process is reduced to a small convexity, shorter than the diameter of the propodeal spiracle. Postpetiolar node convex and lower than petiolar node. In dorsal view, petiolar node not as broad as postpetiolar node (PTW 0.08–0.11, PPW 0.11–0.15), petiolar node wider than long (PNL 0.07–0.08, PTW 0.08–0.11), anterior and posterior margins of petiole weakly convex, and nearly straight in postpetiole.

Dorsal surface of mandibles, clypeus, supraclypeal area and frons smooth and shiny, with scattered piligerous punctae on head and mandibles. Gena and frontal lobes with longitudinal rugae, and transversely rugose close to posterior margin of head. Mesosoma smooth and shiny, except for katepisternum (areolate) and metapleuron (areolate and with longitudinal rugosities). Petiole and ventral face of postpetiole areolate. In dorsal view, dorsal face of promesonotum, postpetiole and gaster smooth and shiny; anterior margin of promesonotum, dorsum of propodeum and petiole finely areolate.

Lateral margins and posterior margin of head and scape with decumbent hairs. Outer margin of mandibles with appressed hairs. Mesosoma with short subdecumbent hairs and long suberect or subdecumbent hairs. Petiole and postpetiole with short appressed hairs and long subdecumbent hairs. Tibia with appressed hairs. Gaster with short decumbent or appressed hairs and long subdecumbent hairs. Color yellowish ferruginous.

#### Distribution and biology.

*Carebara kabosy* was found in the north, northeastern and northwestern areas of Madagascar (Figure 68). The main habitats were montane forest, montane rainforest, rainforest, and tropical forest. *C. kabosy* is distributed at elevations ranging from 25 m to 1325 m. The specimens were collected using maxi-Winkler and pitfall traps. The microhabitats where it occurs include rotten logs, rotten tree stumps, sifted litter, leaf mold, rotten wood, under moss on rotten logs, and under stones.

#### Comments.

*Carebara kabosy* can be confused with *C. vazimba* but can be separated by the length of the propodeal tooth, which is shorter than propodeal spiracle diameter in *C. vazimba* while in *C. kabosy* is slightly longer. *C. kabosy* is distributed in the north, northeastern and northwestern areas of Madagascar, while *C. vazimba* is present in the north of Madagascar. Other twelve species were recorded at the same localities: *C. bara*, *C. berivelo*, *C. betsi*, *C. demeter*, *C. grandidieri*, *C. hainteny*, *C. jajoby*, *C. mahafaly*, *C. malagasy*, *C. nosindambo*, *C. raberi*, and *C. salegi*.

*C. kabosy* have three intermediates in the major worker subcaste (Figure 46). The posterolateral corner of head of intermediates 2 and 3 is nearly rounded, and slightly narrowed in intermediate 1, and the posterior margin of the head is deeply concave in all intermediates. The lateral margins of the head are nearly straight in intermediate 3, and slightly convex in intermediates 1 and 2. Eyes are small and reduced to one ommatidium in intermediates 1 and 2 but have up to 20 ommatidia in intermediate 3. Ocelli are absent in intermediate 1, shallowly marked in intermediate 2, and present and well developed in intermediate 3 (one ocellus). Reduced flight sclerites are present in intermediate 3. The dorsum of the mesosoma is convex anteriorly, gradually sloping to the declivity in intermediate 3, but the promesonotum is high and nearly rounded in intermediates 1 and 2. The petiole and postpetiole vary little in shape among all intermediates, but the anterior margin of the petiole is nearly straight in intermediate 3. The propodeum is armed with a pair of triangular teeth in all intermediates. The head is smooth and shiny in front and has longitudinal and parallel rugae in full-face view. The pilosity on the head and body follow the same pattern in all intermediates, but intermediate 3 has more abundant short decumbent to appressed hairs on the gaster.

#### Additional material examined.

**MADAGASCAR**: ***Antsiranana***: Forêt de Binara, 7.5 km 230° SW Daraina, -13.255, 49.61667, 375 m, tropical dry forest, 1.xii.2003, (*B.L. Fisher*); Antsiranana, Parc National de Marojejy, Antranohofa, 26.6 km 31° NNE Andapa, 10.7 km 318° NW Manantenina, -14.44333, 49.74333, 1325 m, montane rainforest, 18.xi.2003, (*B.L. Fisher*); Antsiranana, Forêt de Binara, 9.1 km 233° SW Daraina, -13.26333, 49.60333, 800 m, rainforest, 3.xii.2003, (*B.L. Fisher*); Antsiranana, Forêt de Binara, 9.4 km 235° SW Daraina, -13.26333, 49.6, 1100 m, montane rainforest, 5.xii.2003, (*B.L. Fisher*); Antsiranana, Parc National de Marojejy, Manantenina River, 27.6 km 35° NE Andapa, 9.6 km 327° NNW Manantenina, -14.435, 49.76, 775 m, rainforest, 15–18.xi.2003, (*B.L. Fisher et al*.); Antsiranana, Parc National de Marojejy, Manantenina River, 28.0 km 38° NE Andapa, 8.2 km 333° NNW Manantenina, -14.43667, 49.775, 450 m, rainforest, 12–15.xi.2003, (*B.L. Fisher et al*.); Antsiranana, Forêt Ambanitaza, 26.1 km 347° Antalaha, -14.67933, 50.18367, 240 m, rainforest, 26.xi.2004, (*B.L. Fisher*); Antsiranana, Forêt d’ Antsahabe, 11.4km 275° W Daraina, -13.21167, 49.55667, 550 m, tropical dry forest, 14.xii.2003, (*B.L. Fisher*); Antsiranana, Parc National Montagne d’Ambre, Ambre grand lac, -12.59656, 49.15932, 1350 m, montane rainforest, 13.xi.2007, (*B.L. Fisher et al*.); Antsiranana, Parc National Montagne d’Ambre, Mahasarika, -12.53176, 49.17662, 1135 m, montane rainforest, 19.xi.2007, (*B.L. Fisher et al*.); Antsiranana, Parc National Montagne d’Ambre, Roussettes, -12.52574, 49.17238, 1025 m, montane rainforest, 15.xi.2007, (*B.L. Fisher et al*.); Antsiranana, Parc National Montagne d’Ambre, Crête, -12.58132, 49.13368, 1110 m, montane rainforest, 13.xi.2007, (*B.L. Fisher et al*.); Antsiranana, Parc National Montagne d’Ambre, Antomboka, -12.51269, 49.17807, 970 m, montane rainforest, 17.xi.2007, (*B.L. Fisher et al*.); Antsiranana, Parc National Montagne d’Ambre, Antomboka, -12.51269, 49.17807, 970 m, montane rainforest, 16.xi.2007, (*B.L. Fisher et al*.); Antsiranana, Parc National Montagne d’Ambre, Antomboka, -12.51269, 49.17807, 970 m, montane rainforest, 19.xi.2007, (*B.L. Fisher et al*.); Antsiranana, Parc National Montagne d’Ambre, -12.51778, 49.17957, 1000 m, montane rainforest, 5.iii.2011, (*B.L. Fisher et al*.); Antsiranana, Makirovana forest, -14.17066, 49.95409, 225 m, rainforest, 4.v.2011, (*B.L. Fisher et al*.); Antsiranana, Parc National Montagne d’Ambre, -12.53417, 49.17607, 1325 m, montane rainforest, 12.iii.2011, (*B.L. Fisher et al*.); Antsiranana, Parc National Montagne d’Ambre, -12.51389, 49.17784, 984 m, montane rainforest, 23.ii.2011, (*B.L. Fisher et al*.); Antsiranana, Parc National Montagne d’Ambre, -12.51389, 49.17784, 984 m, montane rainforest, 25.ii.2011, (*B.L. Fisher et al*.); Antsiranana, Parc National Montagne d’Ambre, -12.51389, 49.17784, 984 m, montane rainforest, 26.ii.2011, (*B.L. Fisher et al*.); Antsiranana, Makirovana forest, -14.17066, 49.95409, 225 m, rainforest, 4–6.v.2011, (*B.L. Fisher et al*.); Antsiranana, Makirovana forest, -14.16044, 49.95216, 550 m, rainforest, 1–2.v.2011, (*B.L. Fisher et al*.); Antsiranana, Makirovana forest, -14.17066, 49.95409, 415 m, rainforest, 29.iv.2011, (*B.L. Fisher et al*.); Antsiranana, Makirovana forest, -14.16666, 49.95, 715 m, rainforest, 1–2.v.2011, (*B.L. Fisher et al*.); Antsiranana, Makirovana forest, -14.17066, 49.95409, 415 m, rainforest, 28–29.iv.2011, (*B.L. Fisher et al*.); Antsiranana, Galoko chain, Mont Galoko, -13.58487, 48.71818, 520 m, rainforest, 17.ii.2013, (*B.L. Fisher et al*.); Antsiranana, Galoko chain, Mont Galoko, -13.5888, 48.72864, 980 m, montane forest, 18.ii.2013, (*B.L. Fisher et al*.); Antsiranana, Galoko chain, Mont Galoko, -13.58745, 48.71419, 380 m, rainforest, 23.ii.2013, (*B.L. Fisher et al*.); Antsiranana, Galoko chain, Mont Galoko, -13.59358, 48.73157, 1100 m, montane forest, 22.ii.2013, (*B.L. Fisher et al*.); Antsiranana, Ampasindava, Forêt d’Ambilanivy, 3.9 km 181° S Ambaliha, -13.79861, 48.16167, 600 m, rainforest, 4–9.iii.2001, (*Fisher, Griswold et al.*); Antsiranana, Parc National Montagne d’Ambre, 12.2 km 211° SSW Joffreville, -12.59639, 49.1595, 1300 m, montane rainforest, 2–7.ii.2001, (*Fisher, Griswold et al.*); Antsiranana, Forêt de Binara, 9.4km 235° SW Daraina, -13.26333, 49.6, 1100 m, montane rainforest, 6.xii.2003, (*B.L. Fisher*); Antsiranana, Réserve Spéciale Manongarivo, 14.5 km 220° SW Antanambao, -13.99833, 48.42833, 1175 m, montane rainforest, 20.x.1998, (*B.L. Fisher*); ***Toamasina***: 6.9 km NE Ambanizana, Ambohitsitondroina, -15.58506, 50.00952, 825 m, rainforest, 2.xii.1993, (*B.L. Fisher*); Toamasina, Montagne d’Anjanaharibe, 18.0 km 21° NNE Ambinanitelo, -15.18833, 49.615, 470 m, rainforest, 8–12.iii.2003, (*Fisher, Griswold et al.*); Toamasina, Montagne d’Anjanaharibe, 19.5 km 27° NNE Ambinanitelo, -15.17833, 49.635, 1100 m, montane rainforest, 12–16.iii.2003, (*Fisher, Griswold et al.*); Toamasina, Montagne d’Akirindro 7.6 km 341° NNW Ambinanitelo, -15.28833, 49.54833, 600 m, rainforest, 17–21.iii.2003, (*Fisher, Griswold et al.*); Toamasina, Station Forestière Analamazaotra, Analamazaotra 1.3 km S Andasibe, -18.38466, 48.41271, 980 m, montane rainforest, 11–13.xii.2007, (*B.L. Fisher et al*.); Toamasina, Réserve Spéciale Ambatovaky, Sandrangato river, -16.77274, 49.26551, 450 m, rainforest, 20–22.ii.2010, (*B.L. Fisher et al*.); Toamasina, Réserve Spéciale Ambatovaky, Sandrangato river, -16.7633, 49.26692, 520 m, rainforest, 22–24.ii.2010, (*B.L. Fisher et al*.); Toamasina, Réserve Spéciale Ambatovaky, Sandrangato river, -16.81753, 49.29498, 360 m, rainforest, 25–27.ii.2010, (*B.L. Fisher et al*.); Toamasina, 6.3 km S Ambanizana, Andranobe, -15.6813, 49.958, 25 m, rainforest, 14.xi.1993, (*B.L. Fisher*); Toamasina, Corridor Forestier Analamay-Mantadia, Ambohibolakely, -18.77908, 48.36628, 1014 m, rainforest, 23–28.xi.2012, (*B.L. Fisher et al*.); Toamasina, Corridor Forestier Analamay-Mantadia, Ambohibolakely, -18.76131, 48.36437, 983 m, rainforest, 23–28.xi.2012, (*B.L. Fisher et al*.).

### Carebara lova

Taxon classificationAnimaliaHymenopteraFormicidae

Azorsa & Fisher sp. n.

http://zoobank.org/E900664C-C2B9-467A-A11F-7FFACA553E1F

#### Holotype.

(major worker), MADAGASCAR, Mahajanga, Parc National d’Ankarafantsika, Ampijoroa Station Forestière, 5.4 km 331° NW Andranofasika, -16.29889, 46.813, 70 m, tropical dry forest, 26.iii.01.iv.2001, (*Fisher, Griswold et al.*). Collection code BLF03571, (CASC: CASENT0468707). **Paratypes**: (12 major workers and 17 minor workers), with same data as holotype, 12 major workers (BMNH: CASENT0468552, CASC: CASENT0468675, CASENT0468639, CASENT0468605, CASENT0468494, CASENT0468503, CASENT0468578, CASENT0431674, CASENT0468476, MCZ: CASENT0468685, MHNG: CASENT0468659, NHMB: CASENT0468474), and 17 minor workers (BMNH: CASENT0468630, CASC: CASENT0468621, CASENT0468626, CASENT0468562, CASENT0468579, CASENT0468548, CASENT0468704, CASENT0468701, CASENT0468693, CASENT0468593, CASENT0468599, CASENT0468690, CASENT0468551, CASENT0468694, MCZ: CASENT0468668, MHNG: CASENT0468635, NHMB: CASENT0468673).

#### Diagnosis.

Antennae ten-segmented. **Major**: Head longer than wide, posterior margin of head deeply concave; dorsum of propodeum flat and declining posteriorly, posterodorsal corner of propodeum with small triangular teeth; combined outline of dorsal surface of peduncle and anterior face of node medially concave, posterior face vertical and slightly convex; gaster with sparse, short appressed hairs, and dispersed longer suberect hairs. **Minor**: Head longer than wide, posterior margin of head nearly straight; propodeum slightly convex and declining posteriorly, nearly flat, posterodorsal corner of propodeum with a very small triangular tooth; gaster with short decumbent to appressed hairs, and dispersed longer suberect hairs.

**Figure 47.**
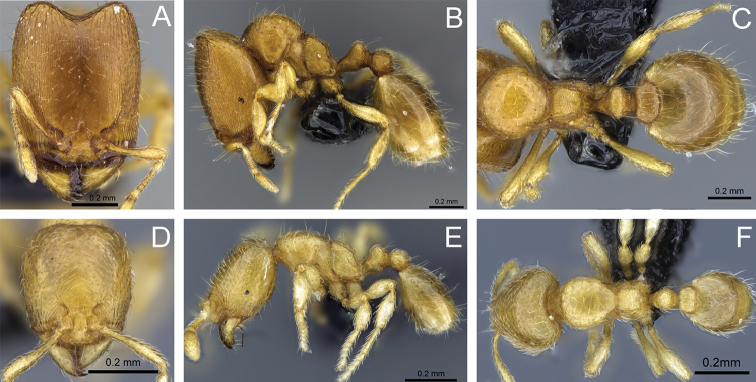
*Carebara lova*–holotype. Major worker, CASENT0468707: **A** head in full-face view **B** body in profile view **C** body in dorsal view. Minor worker, CASENT0468562: **D** head in full-face view **E** body in profile view **F** body in dorsal view.

**Figure 48.**
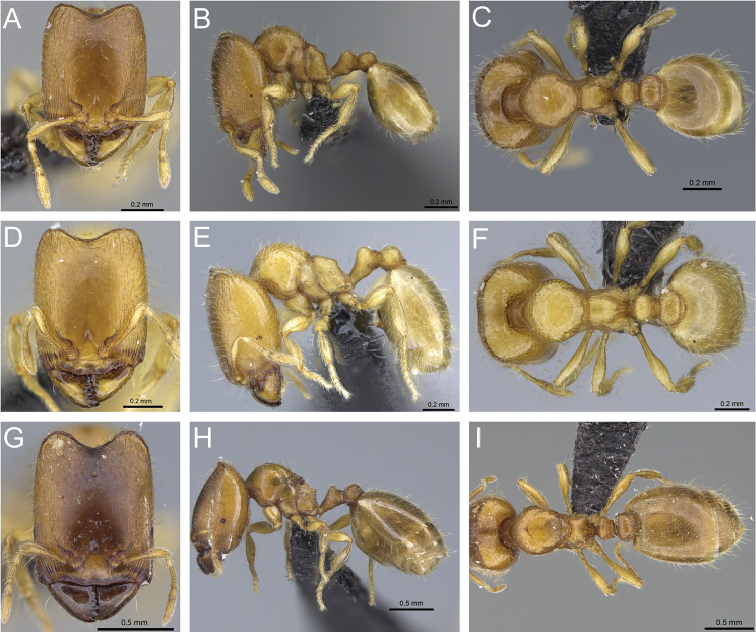
Intermediates of *Carebara lova*. Major workers, CASENT0470797: **A** head in full-face view **B** body in profile view **C** body in dorsal view. CASENT0036019: **D** head in full-face view **E** body in profile view **F** body in dorsal view. CASENT0035922: **G** head in full-face view **H** body in profile view **I** body in dorsal view.

#### Description of major workers.

**Measurements** (n=36): HL 0.60–1.05 (0.72); HW 0.49–0.85 (0.56); SL 0.25–0.37 (0.28); ML 0.14–0.26 (0.19); EL 0.01–0.08 (0.03); EM 0.17–0.32 (0.20); HD 0.33–0.63 (0.40); WL 0.51–0.96 (0.60); PSL 0.05–0.12 (0.07); PW 0.30–0.57 (0.36); MFL 0.30–0.53 (0.33); MFW 0.07–0.12 (0.08); MTL 0.22–0.41 (0.27); PTL 0.19–0.39 (0.23); PNL 0.09–0.17 (0.10); PTH 0.10–0.26 (0.17); PTW 0.13–0.25 (0.15); PPL 0.12–0.22 (0.14); PPNL 0.11–0.21 (0.12); PPH 0.12–0.27 (0.15); PPW 0.17–0.36 (0.20); GL 0.42–1.40 (0.60); GW 0.41–0.88 (0.48); CI 75–90 (78); MI 22–27 (26); SI 32–43 (39), MLI 57–65 (59); PPLI 54–90 (61); PPI 117–150 (133); PSI 10–15 (13).

Head longer than wide (CI 75–90), in full-face view nearly subrectangular, about 1.3 times longer than wide, slightly narrowed posteriorly. Posterior margin of head deeply concave, posterolateral corners well developed and rounded, lateral margins slightly convex. Mandibles with six teeth. Anterior margin of clypeus nearly straight, weakly concave, and laterally convex. Antennae with ten segments. Scapes short (HL 0.60–1.05, SL 0.25–0.37, SI 32–43) not surpassing cephalic midlength. Ocelli present or absent. Eyes present, consisting of one to three ommatidia (EL 0.01–0.08). Supraclypeal area short and triangular.

In profile view, posterolateral corner of head with a small angulate triangular tooth resembling a horn. Promesonotum high and nearly rounded, metanotal groove deeply impressed. Propodeum lower than promesonotum, and about 1.5 times higher than long, dorsal face of propodeum nearly flat, and declining posteriorly, propodeum armed, posterodorsal corners each armed with a small laminate triangular tooth, declivity concave with thin lateral laminae. Propodeal lobes short and convex. Propodeal spiracle nearly oval and situated slightly above mid-height of sclerite, and barely beyond mid-length of sclerite by about half the diameter of the spiracle, distance from propodeal spiracle to posterodorsal corner of propodeum about 2.5 times the diameter of the spiracle (PSL 0.05–0.12), and distance to declivity about 1.8 times the diameter of the spiracle. In dorsal view, promesonotum about as long as wide, anterior margin and sides rounded, slightly narrowed anteriorly and posteriorly; sides of propodeum straight.

Petiole longer than high (PTL 0.19–0.39, PTH 0.10–0.26) and with relatively short peduncle, ventral face medially convex. Combined outline of dorsal surface of peduncle and anterior face of node medially concave, posterior face of node vertical and weakly convex, anterodorsal corner convex, posterodorsal corner rounded, dorsum convex. Subpetiolar process produced as denticle, slightly larger than the diameter of the propodeal spiracle. Postpetiolar node convex and lower than petiolar node. In dorsal view, postpetiolar node wider than petiolar node (PTW 0.13–0.25, PPW 0.17–0.36) and petiolar node wider than long (PNL 0.09–0.17, PTW 0.13–0.25), anterior margin of petiole convex, posterior margin nearly straight, anterior margin of postpetiole concave, and convex posterior margin, sides rounded in petiole and postpetiole.

Dorsal surface of mandibles, clypeus, supraclypeal area and frons smooth and shiny, with scattered piligerous punctae on head and mandibles. Dorsolateral faces of head with longitudinal rugae, gena and frontal lobes with longitudinal rugae extending to posterior border of posterolateral corners. In profile, posterolateral portion of cephalic dorsum smooth and shiny. Mesosoma smooth and shiny, except for katepisternum and metapleuron, which are longitudinally areolate-rugose. Petiole, ventral face, and lateral margins of postpetiole areolate. In dorsal view, promesonotum, petiole, postpetiole and gaster smooth and shiny, except for propodeum which is finely areolate.

Lateral margins of head with short appressed hairs and long suberect hairs. Posterior margin of head with short and long suberect hairs. Scapes with appressed hairs. Outer margin of mandibles with sparse and short appressed hairs. Mesosoma with short and long suberect hairs. Petiole and postpetiole with short decumbent to appressed hairs and long subdecumbent hairs. Tibia with appressed hairs. Gaster with short decumbent to appressed hairs and dispersed, long suberect hairs. Color yellowish ferruginous.

#### Description of minor workers.

**Measurements** (n=26): HL 0.32–0.38; HW 0.28–0.35; SL 0.20–0.24; ML 0.08–0.10; EL 0.01–0.02; EM 0.09–0.12; HD 0.20–0.23; WL 0.32–0.40; PSL 0.03–0.05; PW 0.11–0.20; MFL 0.20–0.25; MFW 0.05–0.06; MTL 0.15–0.19; PTL 0.11–0.14; PNL 0.05–0.07; PTH 0.09–0.11; PTW 0.08–0.09; PPL 0.07–0.10; PPNL 0.06–0.09; PPH 0.06–0.09; PPW 0.10–0.13; GL 0.26–0.39; GW 0.19–0.29; CI 83–95; MI 21–27; SI 60–65; MLI 65–78; PPLI 57–73; PPI 122–150; PSI 9–16.

Head longer than wide (CI 83–95), in full-face view nearly subquadrate, about 1.1 times longer than wide, narrowed anteriorly and posteriorly. Posterior margin of head nearly straight to weakly concave medially, posterolateral corners rounded, lateral margins convex. Mandibles with five teeth. Anterior margin of clypeus nearly straight, and laterally convex. Antennae with ten segments. Scape fails to reach the posterior margin of head (HL 0.32–0.38, SL 0.20–0.24, SI 60–65). Eyes present, consisting of one ommatidium (EL 0.01–0.02). Supraclypeal area triangular but not well defined.

In profile, promesonotum slightly convex, nearly flat, metanotal groove present. Propodeum about 1.4 times higher than long, dorsal face of propodeum nearly flat to weakly convex, and declining posteriorly, propodeum armed, posterodorsal corners each armed with a small triangular tooth, declivity concave with thin lateral laminae. Propodeal lobes short and convex. Propodeal spiracle rounded and situated above mid-height of sclerite by about half the diameter of the spiracle, and beyond mid-length of sclerite by about the diameter of the spiracle, distance from propodeal spiracle to posterodorsal corner of propodeum almost the same as the diameter of the spiracle (PSL 0.03–0.05), and distance to declivity less than half the diameter of the spiracle. In dorsal view, anterior margin of promesonotum convex, sides rounded and narrowed posteriorly, slightly longer than wide; sides of propodeum straight.

Petiole longer than high (PTL 0.11–0.14, PTH 0.09–0.11) and with relatively short peduncle, ventral face medially convex. Combined outline of dorsal surface of peduncle and anterior face of node medially concave, posterior face of node weakly convex, anterodorsal corner convex and posterodorsal corner rounded, dorsum nearly rounded. Subpetiolar process produced as a small denticle, weakly shorter than the diameter of the propodeal spiracle. Postpetiolar node nearly rounded and lower than petiolar node. In dorsal view, postpetiolar node slightly wider than petiolar node (PTW 0.08–0.09, PPW 0.10–0.13), and petiolar node wider than long (PNL 0.05–0.07, PTW 0.08–0.09), anterior margin of petiole convex, posterior margin nearly straight, anterior and posterior margin of postpetiole nearly straight, sides of petiole and postpetiole rounded.

Dorsal surface of mandibles, clypeus, supraclypeal area and frons smooth and shiny, with scattered piligerous punctae on head and mandibles. Gena and frontal lobes with longitudinal rugae. Mesosoma smooth and shiny, except for katepisternum, metapleuron and propodeum which are areolate. Petiole and ventral face of postpetiole areolate. In dorsal view, promesonotum, postpetiole and gaster smooth and shiny; propodeum and petiole areolate.

Posterior and lateral margins of head with short decumbent hairs. Scapes with appressed hairs. Outer margin of mandibles with short appressed hairs. Mesosoma with short suberect hairs and long erect hairs. Petiole and postpetiole with short decumbent hairs and long subdecumbent hairs. Tibia with appressed hairs. Gaster with short decumbent hairs and long suberect hairs. Color yellowish ferruginous.

#### Distribution and biology.

*Carebara lova* is known only from Madagascar, distributed in the northwestern part of the island (Figure 68). This species occurs in the following ecosystems: gallery forest, gallery forest on sandy soil, rainforest and tropical dry forest. It was collected at elevations ranging from 30 m to 500 m. Individuals and colonies were found in sifted litter, rotten logs, leaf mold, and rotten wood. All specimens were collected using maxi-Winkler and pitfall traps.

#### Comments.

*Carebara lova* can be confused with *C. sampi* but can be separated by the distance from posterior border of propodeal spiracle to declivity, which is less than two times the diameter of the spiracle in *C. sampi* and is same as or less than the diameter of the spiracle in *C. lova. C. lova* is distributed in the northwestern area of Madagascar while *C. sampi* is distributed in the south and southwestern of Madagascar. Other two species were recorded at the same localities: *C. bara*, and *C. grandidieri*.

*C. lova* have three intermediates in the major worker subcaste (Figure 48). The posterolateral corner of the head of intermediates 2 and 3 is nearly rounded, and slightly narrowed posteriorly in intermediate 1; the posterior margin of the head is deeply concave in all intermediates. The lateral margins of the head are nearly straight in bigger major workers, and slightly convex in smaller and medium major workers. The eyes are small and reduced to one ommatidium in the smaller and medium intermediates,but may consist of up to four ommatidia in the bigger ones. Ocelli are absent in the smaller and medium major workers, but present and well developed in the big major workers (one ocellus). Reduced flight sclerites are present in the largest intermediate. The dorsum of the mesosoma is convex anteriorly and gradually slopes to the declivity in intermediate 3. The dorsum of the promesonotum is high and strongly convex in intermediates 1 and 2. The shape of the petiole and postpetiole vary little in all intermediates, although the anterior margin of the petiole is nearly straight in intermediate 3 and the petiolar node is thinner than that of intermediates 1 and 2. The propodeum is armed with a pair of triangular teeth in all intermediates. Frons smooth and shiny, dorsolateral faces with finely longitudinal and parallel rugae in full-face view in all intermediates. The pilosity on the head and body follows the same pattern in all intermediates, with intermediate 3 with more abundant, short decumbent hairs on the gaster.

#### Additional material examined.

**MADAGASCAR**: ***Mahajanga***: Parc National de Namoroka, 17.8 km 329° WNW Vilanandro, -16.37667, 45.32667, 100 m, tropical dry forest, 8–12.xi.2002, (*Fisher, Griswold et al.*); Mahajanga, Réserve Spéciale de Bemarivo, 23.8 km 223° SW Besalampy, -16.925, 44.36833, 30 m, tropical dry forest, 19–23.xi.2002, (*Fisher, Griswold et al.*); Mahajanga, Parc National de Namoroka, 16.9 km 317° NW Vilanandro, -16.40667, 45.31, 100 m, tropical dry forest, 12–16.xi.2002, (*Fisher, Griswold et al.*); Mahajanga, Parc National d’Ankarafantsika, Forêt de Tsimaloto, 18.3 km 46° NE de Tsaramandroso, -16.22806, 47.14361, 135 m, tropical dry forest, 2–8.iv.2001, (*Fisher, Griswold et al.*); Mahajanga, Réserve d’Ankoririka, 10.6 km 13° NE de Tsaramandroso, -16.26722, 47.04861, 210 m, tropical dry forest, 9–14.iv.2001, (*Fisher, Griswold et al.*); Mahajanga, Mahavavy River, 6.2 km 145° SE Mitsinjo, -16.05167, 45.90833, 20 m, gallery forest, 1–5.xii.2002, (*Fisher*, *Griswold et al.*).

### Carebara mahafaly

Taxon classificationAnimaliaHymenopteraFormicidae

Azorsa & Fisher sp. n.

http://zoobank.org/7989A426-4B88-4455-A593-8E113CF7C052

#### Holotype.

(major worker), MADAGASCAR, Antananarivo, 3 km 41° NE Andranomay, 11.5 km 147° SSE Anjozorobe, -18.47333, 47.96, 1300 m, montane rainforest, 5–13.xii.2000, (*Fisher, Griswold et al.*). Collection code BLF02378, (CASC: CASENT0410500). **Paratypes**: (1 major worker and 5 minor workers), with same data as holotype, 5 minor workers (BMNH: CASENT0410532, CASC: CASENT0410533, CASENT0410536, CASENT0410535, MCZ: CASENT0410537). 1 major worker with same data as holotype but with different collection code, BLF02371 (CASC: CASENT0410502).

#### Diagnosis.

Antennae ten-segmented. **Major**: Head slightly longer than wide, nearly subquadrate, lateral margins strongly convex; posterior margin of head deeply concave; dorsum of propodeum weakly convex, nearly flat; posterodorsal corner of propodeum with a pair of laminate triangular teeth; petiolar node thick, combined outline of dorsal surface of peduncle and anterior face of node concave and posterior face vertical and weakly convex; gaster with short appressed hairs. **Minor**: Head slightly longer than wide, narrowed anteriorly; lateral margins of head slightly convex; posterior margin of head straight; dorsum of propodeum medially concave, posterodorsal corner with a pair of laminate triangular teeth; petiolar node thick, combined outline of dorsal surface of peduncle and anterior face of node slightly concave in the middle, dorsum slightly convex; gaster with short appressed hairs.

**Figure 49.**
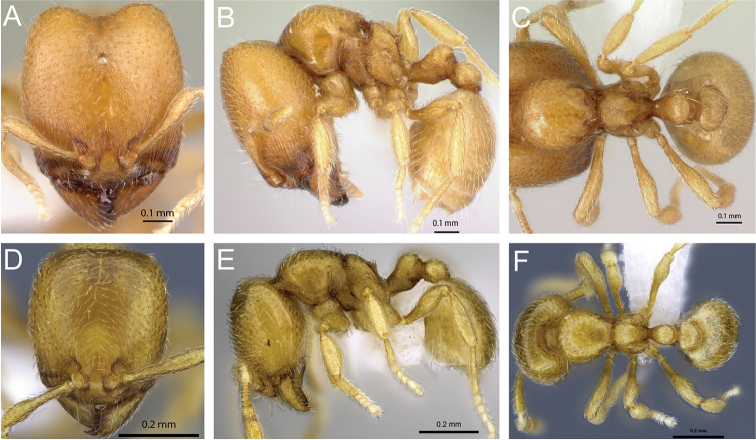
*Carebara mahafaly*–holotype. Major worker, CASENT0410500: **A** head in full-face view **B** body in profile view **C** body in dorsal view. Minor worker, CASENT0410535: **D** head in full-face view **E** body in profile view **F** body in dorsal view.

#### Description of major workers.

**Measurements** (n=2): HL 0.65–0.75 (0.65); HW 0.58–0.65 (0.58); SL 0.31–0.32 (0.32); ML 0.15; EL 0.02–0.03 (0.02); EM 0.20–0.21 (0.20); HD 0.38–0.42 (0.38); WL 0.56–0.61 (0.56); PSL 0.08–0.09 (0.09); PW 0.30–0.36 (0.30); MFL 0.34–0.37 (0.34); MFW 0.07–0.09 (0.07); MTL 0.27–0.31 (0.27); PTL 0.22–0.25 (0.22); PNL 0.09–0.11 (0.09); PTH 0.17–0.18 (0.17); PTW 0.12–0.15 (0.12); PPL 0.13–0.14 (0.14); PPNL 0.10–0.13 (0.10); PPH 0.12; PPW 0.17–0.20 (0.17); GL 0.49–0.62 (0.49); GW 0.45–0.54 (0.45); CI 87–88 (88); MI 20–23 (23); SI 41–49 (49); MLI 57–59 (59), PPLI 52–64 (64); PPI 133–142 (142); PSI 12–16 (16).

Head slightly longer than wide (CI 87–88), in full-face view nearly subquadrate, about 1.1 times longer than wide. Posterior margin of head deeply concave in the middle, posterolateral corners well developed and rounded, lateral margins strongly convex. Mandibles with six teeth. Anterior margin of clypeus nearly straight, and laterally convex. Ocelli present or absent. Antennae with ten segments. Scapes short (HL 0.65–0.75, SL 0.31–0.32, SI 41–49). Eyes present, consisting of one ommatidium (EL 0.02–0.03). Supraclypeal area acutely triangular and well defined.

In profile view, promesonotum high and strongly convex, metanotal groove deeply impressed, propodeum about 1.8 times higher than long, and lower than promesonotum, dorsal face of propodeum nearly flat and declining posteriorly, propodeum armed, posterodorsal corners each armed with a triangular laminate tooth, anterodorsal corner slightly convex, declivity weakly concave, nearly flat, with thin lateral laminae. Propodeal lobes convex. Propodeal spiracle rounded and situated above mid-height of sclerite by about the diameter of the spiracle, and beyond mid-length of sclerite by about half the diameter of the spiracle, distance from propodeal spiracle to posterodorsal corner of propodeum about 3.5 times the diameter of the spiracle (PSL 0.08–0.09), and distance to declivity about 2.2 times the diameter of the spiracle. In dorsal view, promesonotum about as long as wide, anterior margin and sides of promesonotum rounded and narrowed posteriorly; sides of propodeum straight.

Petiole longer than high (PTL 0.22–0.25, PTH 0.17–0.18) and with relatively long peduncle, ventral face medially convex. Combined outline of dorsal surface of peduncle and anterior face of node medially concave, posterior face of node vertical and nearly straight, anterodorsal corner strongly convex, posterodorsal corner rounded, dorsum convex. Subpetiolar process absent, when present, reduced to a small convexity. Postpetiolar node nearly rounded and lower than petiolar node. In dorsal view, postpetiolar node wider than petiolar node (PTW 0.12–0.15, PPW 0.17–0.20) and petiolar node wider than long (PNL 0.09–0.11, PTW 0.12–0.15), anterior margin of petiole straight, posterior margin convex, anterior margin of postpetiole concave and posterior margin convex, sides rounded in petiole and postpetiole.

Dorsal surface of mandibles, clypeus, supraclypeal area and the median portion of the frons smooth and shiny, with scattered piligerous punctae on head and mandibles. Head with finely longitudinal and parallel rugae, except for median area and near posterior margin, gena with longitudinal rugae, extending to eye level, frontal lobes with longitudinal rugae, extending to antennae level. In profile, posterolateral portion of cephalic dorsum smooth and shiny. Mesosoma smooth and shiny, except katepisternum and metapleuron (areolate-rugose), and propodeum (areolate). Petiole and ventral face of postpetiole areolate. In dorsal view, promesonotum, petiole, postpetiole and gaster smooth and shiny except for propodeum which is areolate.

Lateral margins of head with short appressed hairs, posterior margin with short and long subdecumbent hairs. Scapes with appressed hairs. Outer margin of mandibles with short and sparse appressed hairs. Mesosoma with short and long suberect hairs. Petiole and postpetiole with short appressed hairs and long subdecumbent hairs. Tibia with short appressed hairs. Gaster with short appressed hairs and sparse and long decumbent hairs. Color yellowish ferruginous.

#### Description of minor workers.

**Measurements** (n=7): HL 0.37–0.46; HW 0.32–0.42; SL 0.23–0.29; ML 0.09–0.11; EL 0.01–0.02; EM 0.11–0.15; HD 0.22–0.29; WL 0.35–0.46; PSL 0.04–0.06; PW 0.19–0.24; MFL 0.22–0.31; MFW 0.05–0.07; MTL 0.17–0.25, PTL 0.13–0.18; PNL 0.06–0.08; PTH 0.10–0.13; PTW 0.08–0.10; PPL 0.08–0.10; PPNL 0.07–0.10; PPH 0.07–0.10; PPW 0.11–0.15; GL 0.30–0.45; GW 0.23–0.35; CI 86–93; MI 22–26; SI 61–66, MLI 69–74; PPLI 56–63; PPI 130–150; PSI 11–14.

Head longer than wide (CI 86–93), in full-face view nearly subquadrate, about 1.1 times longer than wide and slightly narrowed anteriorly. Posterior margin of head nearly straight, posterolateral corners rounded, lateral margins convex. Mandibles with five teeth. Anterior margin of clypeus slightly concave, and laterally convex. Antennae with ten segments. Scape fails to reach the posterior margin of head (HL 0.37–0.46, SL 0.23–0.29, SI 61–66). Eyes present, consisting of one ommatidium (EL 0.01–0.02). Supraclypeal area triangular but poorly defined.

In profile view, promesonotum weakly convex, metanotal groove deeply impressed. Propodeum about 1.1 times higher than long, dorsal face of propodeum weakly convex and declining posteriorly, propodeum armed, posterodorsal corners each armed with a laminate triangular tooth, anterodorsal corner rounded and at same height as posterodorsal corner of promesonotum, declivity flat to slightly convex with thin lateral laminae. Propodeal lobes convex. Propodeal spiracle rounded and situated slightly above mid-height of sclerite by about half the diameter of the spiracle, and beyond mid-length of sclerite by about 1.5 times the diameter of the spiracle, distance from propodeal spiracle to posterodorsal corner of propodeum about three times the diameter of the spiracle (PSL 0.04–0.06), and distance to declivity same as the diameter of the spiracle. In dorsal view, anterior margin and sides of promesonotum rounded, narrowed posteriorly, slightly longer than wide; sides of propodeum straight.

Petiole longer than high (PTL 0.13–0.18, PTH 0.10–0.13) and with relatively long peduncle, ventral face medially convex. Combined outline of dorsal surface of peduncle and anterior face of node medially concave, posterior face of node slightly convex, anterodorsal corner convex, posterodorsal corner rounded, dorsum nearly rounded. Subpetiolar process is reduced to a small convexity, not longer than the diameter of the propodeal spiracle. Postpetiolar node rounded and lower than petiolar node. In dorsal view, postpetiolar node wider than petiolar node (PTW 0.08–0.10, PPW 0.11–0.15), and petiolar node as wide as long, anterior margin of petiole strongly convex, posterior margin rounded, anterior margin of postpetiole nearly straight, posterior margin rounded, sides convex in petiole and postpetiole, narrowed posteriorly in postpetiole.

Dorsal surface of mandibles, clypeus, supraclypeal area and frons smooth and shiny, with scattered piligerous punctae on head and mandibles. Gena and frontal lobes with longitudinal rugae. Mesosoma smooth and shiny, except for katepisternum (areolate), and metapleuron and propodeum (finely areolate). Petiole and ventral face of postpetiole areolate. In dorsal view, promesonotum, petiole, postpetiole and gaster smooth and shiny except for propodeum which is areolate.

Lateral margins of head with short appressed hairs, posterior margin with short subdecumbent hairs. Scapes with appressed hairs. Outer margin of mandibles with decumbent hairs. Mesosoma with short and long suberect to subdecumbent hairs. Petiole, postpetiole, tibia and gaster with short appressed hairs. Color yellowish ferruginous.

#### Distribution and biology.

*Carebara mahafaly* is known from two localities in the center of Madagascar (Figure 69). This species occurs in the rainforest and montane rainforest and was found at elevations of 780 m and 1300 m. Individuals and colonies were collected in sifted litter, leaf mold and rotten wood. Specimens were collected using maxi-Winkler and pitfall traps.

#### Comments.

*Carebara mahafaly* is endemic to the center of Madagascar. Other six species were recorded at the same localities: *C. betsi*, *C. grandidieri*, *C. hainteny*, *C. jajoby*, *C. kabosy*, and *C. nosindambo*. This species can be confused with *C. salegi*, especially in the shape of the head. *C. mahafaly* can be differentiated from *C. salegi* and other species by the following combination of characters: head slightly longer than wide, nearly subquadrate in full-face view, posterolateral corners rounded, lateral margins of head convex; propodeum with a pair of upwardly directed triangular teeth, and anterodorsal corner of petiole convex, additional to this *C. salegi* was recorded only in the north of Madagascar. *C. mahafaly* does not have intermediates in the major worker subcaste.

#### Additional material examined.

**MADAGASCAR**: ***Toamasina***: Parc National de Zahamena, Onibe River, -17.75908, 48.85468, 780 m, rainforest, 21–23.ii.2009, (*B.L. Fisher et al*.).

### Carebara malagasy

Taxon classificationAnimaliaHymenopteraFormicidae

Azorsa & Fisher sp. n.

http://zoobank.org/E6585384-98DE-46B5-94D7-E5C395E2C975

#### Holotype.

(major worker), MADAGASCAR, Antsiranana, Forêt de Bekaraoka, 6.8km 60° ENE Daraina, -13.16667, 49.71, 150 m, tropical dry forest, 7.xii.2003, (*B.L. Fisher*). Collection code BLF09872, (CASC: CASENT0044166). **Paratypes**: (4 major workers and 16 minor workers), with same data as holotype, 4 major workers (BMNH: CASENT0044178, CASC: CASENT0044274, CASENT0044173, CASENT0044169), and 16 minor workers (BMNH: CASENT0044161, CASC: CASENT0044167, CASENT0044168, CASENT0044275, CASENT0044165, CASENT0044179, CASENT0044175, CASENT0044174, CASENT0044162, CASENT0044176, CASENT0044277, CASENT0044276, CASENT0044180, MCZ: CASENT0044172, MHNG: CASENT0044163, NHMB: CASENT0044177).

#### Diagnosis.

Antennae ten-segmented. **Major**: Head subrectangular, longer than wide, lateral margins weakly convex and narrowed at the top, posterolateral corners well developed, posterior margin of head deeply concave; dorsum of propodeum convex, posterodorsal corner angulate; gaster with short decumbent hairs and sparse longer subdecumbent hairs. **Minor**: Head slightly longer than wide, promesonotum weakly convex; dorsum of propodeum convex, posterodorsal corner angulate; gaster with short decumbent hairs, and sparse longer subdecumbent hairs.

**Figure 50.**
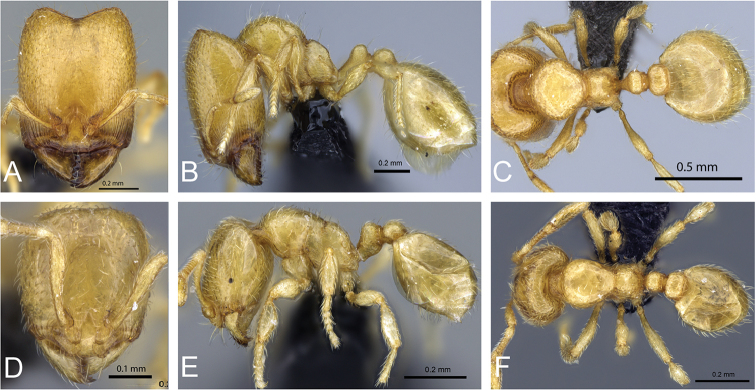
*Carebara malagasy*–holotype. Major worker, CASENT0044166: **A** head in full-face view **B** body in profile view **C** body in dorsal view. Minor worker, CASENT0044172: **D** head in full-face view **E** body in profile view **F** body in dorsal view.

**Figure 51.**
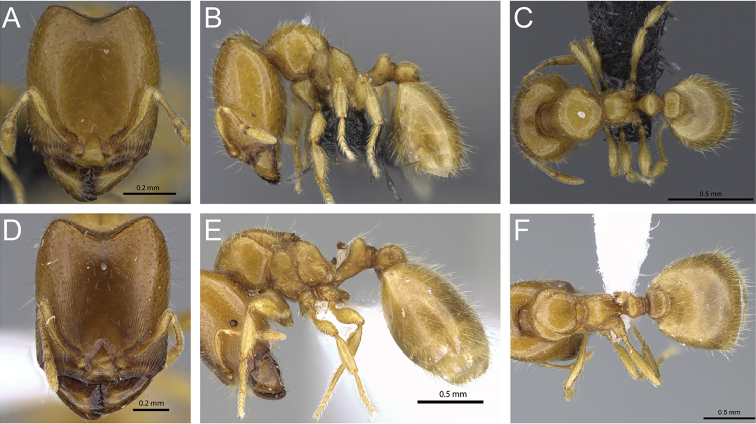
Intermediates of *Carebara malagasy*. Major workers, CASENT0141887: **A** head in full-face view **B** body in profile view **C** body in dorsal view. CASENT0438154: **D** head in full-face view **E** body in profile view **F** body in dorsal view.

#### Description of major workers.

**Measurements** (n=16): HL 0.56–1.02 (0.75); HW 0.46–0.80 (0.59); SL 0.25–0.37 (0.28); ML 0.13–0.27 (0.18); EL 0.01–0.05 (0.01); EM 0.14–0.30 (0.22); HD 0.30–0.59 (0.41); WL 0.48–0.91 (0.62); PSL 0.04–0.10 (0.05); PW 0.28–0.55 (0.38); MFL 0.27–0.53 (0.36); MFW 0.06–0.12 (0.07); MTL 0.19–0.45 (0.26); PTL 0.17–0.36 (0.25); PNL 0.08–0.14 (0.10); PTH 0.13–0.25 (0.18); PTW 0.12–0.23 (0.15); PPL 0.11–0.20 (0.14); PPNL 0.10–0.20 (0.12); PPH 0.11–0.25 (0.15); PPW 0.16–0.32 (0.20); GL 0.38–1.15 (0.62); GW 0.39–0.94 (0.57); CI 73–83 (79); MI 20–26 (24); SI 35–69 (37); MLI 58–69 (61); PPLI 55–71 (56); PPI 129–150 (133); PSI 8–13 (8).

Head longer than wide (CI 73–83), in full-face view nearly subrectangular, about 1.2 times longer than wide, narrowed at the top. Posterior margin of head deeply concave, posterolateral corners well developed and rounded, lateral margins weakly convex. Mandibles with six teeth. Anterior margin of clypeus slightly concave, and laterally convex. Ocelli present or absent. Antennae with ten segments. Scapes short (HL 0.56–1.02, SL 0.25–0.37, SI 35–69). Eyes present, consisting of one ommatidium (EL 0.01–0.05). Supraclypeal area short and triangular, but shallowly impressed.

In profile, promesonotum high and nearly rounded, metanotal groove deeply impressed. Propodeum lower than promesonotum, and about 1.6 times higher than long, dorsal face of propodeum weakly convex and declining posteriorly, propodeum unarmed, anterodorsal corner rounded, posterodorsal corner angulate, declivity weakly concave. Propodeal lobes convex. Propodeal spiracle nearly oval and situated above mid-height of sclerite, and beyond mid-length of sclerite by about half the diameter of the spiracle, distance from propodeal spiracle to posterodorsal corner of propodeum about twice the diameter of the spiracle (PSL 0.04–0.10), and distance to declivity about 1.5 times the diameter of the spiracle. In dorsal view, promesonotum about as long as wide, narrowed posteriorly, anterior margin and sides of promesonotum rounded; sides of propodeum straight.

Petiole longer than high (PTL 0.17–0.36, PTH 0.13–0.25) and with relatively long peduncle, ventral face slightly convex in the middle. Combined outline of dorsal surface of peduncle and anterior face of node concave, posterior face of node vertical and nearly straight, anterodorsal corner convex, posterodorsal corner rounded, dorsum convex. Subpetiolar process produced as denticle, slightly larger than the diameter of the propodeal spiracle. Postpetiolar node nearly rounded and lower than petiolar node. In dorsal view, postpetiolar node wider than petiolar node (PTW 0.12–0.23, PPW 0.16–0.32) and petiolar node wider than long (PNL 0.08–0.14, PTW 0.12–0.23), anterior and posterior margins of petiole and postpetiole nearly straight, sides convex in petiole and nearly rounded in postpetiole.

Dorsal surface of mandibles, clypeus, supraclypeal area and frons smooth and shiny, with scattered piligerous punctae on head and mandibles. Head with finely longitudinal rugae directed toward posterior margin of head. Gena and frontal lobes with longitudinal rugae. Mesosoma smooth and shiny, except for katepisternum (areolate-rugose) and metapleuron (areolate with longitudinal rugae). Petiole and ventral face of postpetiole areolate. In dorsal view, mesosoma, petiole, postpetiole and gaster smooth and shiny.

Lateral margins and posterior margin of head with short subdecumbent hairs and longer suberect hairs. Scapes with appressed hairs. Outer margin of mandible with short and appressed hairs. Mesosoma with short and long suberect hairs. Petiole and postpetiole with short decumbent to appressed hairs and long subdecumbent hairs. Tibia with appressed hairs. Gaster with short decumbent hairs, and long suberect to subdecumbent hairs. Color yellowish ferruginous.

#### Description of minor workers.

**Measurements** (n=15): HL 0.33–0.37; HW 0.29–0.33; SL 0.19–0.24; ML 0.08–0.09; EL 0.01–0.02; EM 0.09–0.12; HD 0.19–0.22; WL 0.33–0.37; PSL 0.02–0.04; PW 0.17–0.20; MFL 0.19–0.25; MFW 0.04–0.06; MTL 0.14–0.18; PTL 0.11–0.14; PNL 0.06–0.07; PTH 0.09–0.10; PTW 0.07–0.08; PPL 0.07–0.08; PPNL 0.06–0.08; PPH 0.06–0.08; PPW 0.10–0.12; GL 0.26–0.34; GW 0.21–0.28; CI 86–94; MI 22–26; SI 57–65; MLI 66–76; PPLI 54–73; PPI 125–150; PSI 6–13.

Head longer than wide (CI 86–94), in full-face view nearly subrectangular, about 1.2 times longer than wide, slightly narrowed anteriorly. Posterior margin slightly convex, posterolateral corners rounded, lateral margins slightly convex. Mandibles with six teeth. Anterior margin of clypeus straight, and laterally with a laminate angulate tooth. Antennae with ten segments. Scape fails to reach the posterior margin of head (HL 0.33–0.37, SL 0.19–0.24, SI 57–65). Eyes present, consisting of one ommatidium (EL 0.01–0.02). Supraclypeal area short and triangular but poorly defined.

In profile view, promesonotum weakly convex, metanotal groove weakly impressed. Propodeum about 1.6 times higher than long, dorsal face of propodeum slightly convex and declining posteriorly, propodeum unarmed, anterodorsal corner slightly convex, posterodorsal corner angulate, declivity nearly flat, with thin lateral laminae. Propodeal lobes convex. Propodeal spiracle rounded and situated slightly above mid-height of sclerite by about half the diameter of the spiracle, and beyond mid-length of sclerite by about 1.5 times the diameter of the spiracle, distance from propodeal spiracle to postedorsal corner of propodeum and to declivity almost the same as the diameter of the spiracle (PSL 0.02–0.04). In dorsal view, promesonotum about 1.1 times longer than wide, anterior margin and sides of promesonotum nearly rounded, and narrowed posteriorly; sides of propodeum weakly convex.

Petiole slightly longer than high (PTL 0.11–0.14, PTH 0.09–0.10) and with a short peduncle, ventral face medially convex. Combined outline of dorsal surface of peduncle and anterior face of node weakly concave, posterior face of node slightly convex, anterodorsal and posterodorsal corner rounded, dorsum convex. Subpetiolar process produced as a small denticle, slightly smaller than the diameter of the propodeal spiracle. Postpetiolar node nearly rounded and lower than petiolar node. In dorsal view, postpetiolar node wider than petiolar node (PTW 0.07–0.08, PPW 0.10–0.12), and petiolar node wider than long (PNL 0.06–0.07, PTW 0.07–0.08), anterior and posterior margins of petiole and postpetiole nearly straight, sides nearly rounded in petiole and postpetiole.

Dorsal surface of head, mandibles, clypeus and supraclypeal area smooth and shiny, with scattered piligerous punctae on head and mandibles. Gena and frontal lobes with short longitudinal rugae. Mesosoma smooth and shiny, except for katepisternum (areolate) and metapleuron (finely areolate-rugose). Petiole and ventral face of postpetiole areolate. In dorsal view, promesonotum, postpetiole and gaster smooth and shiny, except for propodeum and petiole which are weakly areolate.

Lateral margins and posterior margin of head with decumbent hairs. Scapes with appressed hairs. Outer margin of mandible with decumbent hairs. Mesosoma with short and long suberect to subdecumbent hairs. Petiole, and postpetiole with short decumbent hairs and longer subdecumbent hairs. Tibia with appressed hairs. Gaster with short decumbent hairs and sparse long subdecumbent hairs. Color yellowish ferruginous.

#### Distribution and biology.

*Carebara malagasy* is known from the north of Madagascar (Figure 69). This species was found in littoral rainforest and tropical dry forest and collected at elevations ranging from 25 m to 500 m. Individuals and colonies were found under stones, in leaf mold and rotten wood. Specimens were collected using maxi-Winkler and pitfall traps.

#### Comments.

*Carebara malagasy* can be confused with other species but can be separated from other species by the following combination of characters: form of the propodeum, which is unarmed or armed with at least a dentiform angle, dorsum of head with at least the frons smooth and shiny, and posterolateral corners of head narrowing posteriorly. *Carebara malagasy* is endemic to the north of Madagascar, and other five species were recorded at this area: *C. bara*, *Carebara berivelo*, *C. grandidieri*, *C. kabosy*, and *C. tana*.

*C. malagasy* have two intermediates in the major worker subcaste (Figure 51). The posterolateral corner of head in both intermediates is slightly narrowed posteriorly. The posterior margin of the head is deeply concave. The eyes are small and reduced to one ommatidium in intermediate 1, and up to 5 ommatidia in intermediate 2. Ocelli are absent in intermediate 1, but present and well developed in intermediate 2 (one ocellus). Reduced flight sclerites are present in intermediate 2. The dorsum of the promesonotum is high and nearly rounded in intermediate 1, and convex in intermediate 2. In both intermediates, the shapes of the petiole and postpetiole are similar; the propodeum is unarmed; the head is smooth and shiny, with finely longitudinal and parallel rugae; and the pilosity on the head and body follows the same pattern.

#### Additional material examined.

**MADAGASCAR**: ***Antsiranana***: Forêt d’Ampondrabe, 26.3 km 10° NNE Daraina, -12.97, 49.7, 175 m, tropical dry forest, 10.xii.2003, (*B.L. Fisher*); Antsiranana, Forêt d’Analabe, 30.0 km 72° ENE Daraina, -13.08333, 49.90833, 30 m, littoral rainforest, 27.xi.2003, (*B.L. Fisher*); Antsiranana, Forêt de Binara, 7.5 km 230° SW Daraina, -13.255, 49.61667, 375 m, tropical dry forest, 1.xii.2003, (*B.L. Fisher*); Antsiranana, Forêt d’ Antsahabe, 11.4 km 275° W Daraina, -13.21167, 49.55667, 550 m, tropical dry forest, 12.xii.2003, (*B.L. Fisher*); Antsiranana, Réserve Analamerana, 16.7 km 123° Anivorano-Nord, -12.80467, 49.37383, 225 m, tropical dry forest, 3.xii.2004, (*B.L. Fisher*); Antsiranana, Réserve Analamerana, 28.4 km 99° Anivorano-Nord, -12.74667, 49.49483, 60 m, tropical dry forest, 5.xii.2004, (*B.L. Fisher*); Antsiranana, Forêt d’Ampombofofo, -12.09949, 49.33874, 25 m, littoral forest, 21–22.xi.2007, (*B.L. Fisher et al*.); Antsiranana, Forêt d’Orangea, 3.6 km 128° SE Remena, -12.25889, 49.37467, 90 m, littoral rainforest, 22–28.ii.2001, (*Fisher, Griswold et al.*).

### Carebara nosindambo

Taxon classificationAnimaliaHymenopteraFormicidae

(Forel, 1891)

Aeromyrma nosindambo Forel, 1891: 199. Lectotype queen, paralectotype male (designated here). Madagascar: Fianarantsoa, Imérina, Camboué. Collection code: ANTC3175, (MHNG: CASENT0101937). (examined). Combination in Carebara: Fernández, 2004: 235.

#### Diagnosis.

Antennae ten-segmented. **Major**: Head nearly subrectangular, longer than wide, lateral margins straight, posterior margin of head concave to deeply concave; dorsum of propodeum flat and declining posteriorly, posterodorsal corner with a pair of triangular teeth; head with longitudinal and parallel rugae, except the frons; gaster with short decumbent or appressed hairs, and long subdecumbent hairs. **Minor**: Head nearly subquadrate, longer than wide, narrowed anteriorly, lateral margins slightly convex, posterior margin of head nearly straight; dorsum of propodeum weakly convex, posterodorsal corner with a pair of triangular teeth; gaster with short subdecumbent to decumbent hairs and dispersed long suberect or subdecumbent hairs.

**Figure 52.**
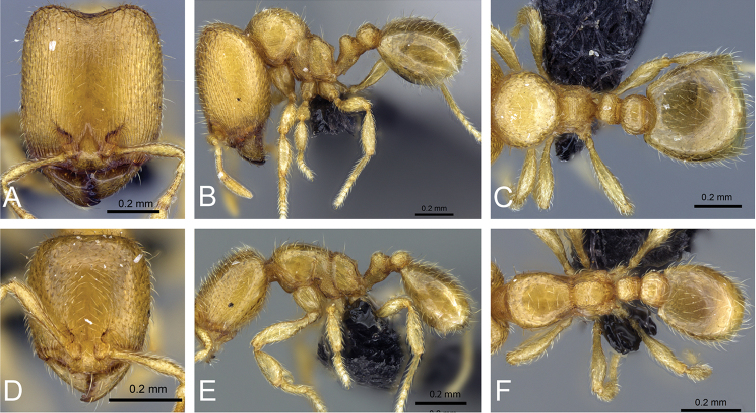
*Carebara nosindambo*, Major worker, CASENT0073096: **A** head in full-face view **B** body in profile view **C** body in dorsal view. Minor worker, CASENT0073105: **D** head in full-face view **E** body in profile view **F** body in dorsal view.

**Figure 53.**
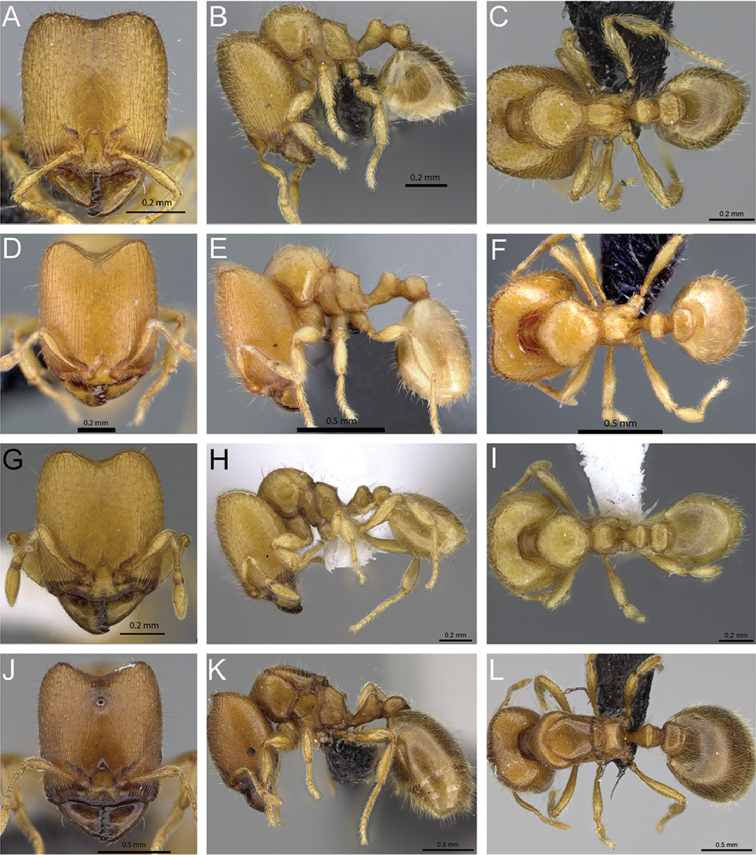
Intermediates of *Carebara nosindambo*. Major workers, CASENT0073098: **A** head in full-face view **B** body in profile view **C** body in dorsal view. CASENT0002849: **D** head in full-face view **E** body in profile view **F** body in dorsal view. CASENT0227032: **G** head in full-face view **H** body in profile view **I** body in dorsal view. CASENT0072632 **J** head in full-face view **K** body in profile view **L** body in dorsal view.

**Figure 54.**
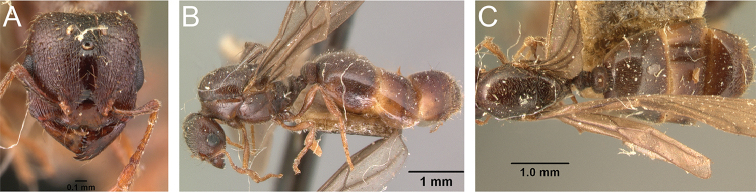
*Carebara nosindambo*-lectotype. Queen, CASENT0101937: **A** head in full-face view **B** body in profile view **C** body in dorsal view.

#### Description of major workers.

**Measurements** (n = 41): HL 0.57–1.06; HW 0.50–0.86; SL 0.26–0.40; ML 0.12–0.28; EL 0.01–0.10; EM 0.17–0.28; HD 0.33–0.63; WL 0.49–0.99; PSL 0.05–0.14; PW 0.30–0.57; MFL 0.28–0.57; MFW 0.07–0.13; MTL 0.20–0.45; PTL 0.18–0.38; PNL 0.07–0.15; PTH 0.14–0.25; PTW 0.11–0.23; PPL 0.11–0.23; PPNL 0.10–0.21; PPH 0.07–0.26; PPW 0.15–0.33; GL 0.40–1.10; GW 0.35–1; CI 78–92; MI 18–29; SI 37–49; MLI 52–70; PPLI 53–79; PPI 126–167; PSI 10–16.

Head slightly longer than wide (CI 78–92), in full-face view nearly subrectangular, about 1.2 times longer than wide. Posterior margin of head medially concave, posterolateral corners well developed, and rounded, lateral margins nearly straight. Mandibles with six teeth. Anterior margin of clypeus weakly concave in the middle, and laterally convex. Antennae with ten or eleven segments (larger major workers). Scapes short (HL 0.57–1.06, SL 0.26–0.40, SI 37–49). Ocelli present or absent. Eyes present, consisting of one to ten ommatidia (EL 0.01–0.10). Supraclypeal area triangular and well defined.

In profile view, posterolateral corner of head with (larger major workers) or without (smaller major workers) a small, obtuse tooth resembling a horn. Promesonotum high and nearly rounded, metanotal groove deeply impressed. Propodeum lower than promesonotum and about 1.8 times higher than long, dorsal face of propodeum flat, sometimes weakly concave, and declining posteriorly, propodeum armed, posterodorsal corners each armed with a laminate triangular tooth, declivity weakly concave, with thin lateral laminae. Propodeal lobes nearly rounded. Propodeal spiracle rounded and situated slightly above mid-height of sclerite by half the diameter of the spiracle, and beyond mid-length of sclerite by the diameter of the spiracle, distance from propodeal spiracle to posterodorsal corner of propodeum about 2.5 times the diameter of the spiracle (PSL 0.05–0.14), and distance to declivity same as the diameter of the spiracle. In dorsal view, promesonotum about as long as wide, anterior margin and sides of rounded; sides of propodeum straight.

Petiole longer than high (PTL 0.18–0.38, PTH 0.14–0.25) and with a relatively long peduncle, ventral face weakly convex in the middle. Combined outile of dorsal surface of peduncle and anterior face of node concave, posterior face of node vertical and weakly convex, anterodorsal and posterodorsal corner rounded, dorsum convex. Subpetiolar process produced as a small denticle, almost as large as the diameter of the propodeal spiracle. Postpetiolar node convex and slightly lower than petiolar node. In dorsal view, petiolar node not as broad as postpetiolar node (PTW 0.11–0.23, PPW 0.15–0.33) and petiolar node wider than long (PNL 0.07–0.15, PTW 0.11–0.23), anterior and posterior margins of petiole and postpetiole straight, sides rounded in petiole and postpetiole.

Dorsal surface of mandibles, clypeus, supraclypeal area and frons smooth and shiny, with scattered piligerous punctae on head and mandibles. Head with longitudinal and parallel rugae in direction of posterior margin of head, and rugoreticulate close to posterior margin of head. Mesosoma smooth and shiny, except for katepisternum (areolate), metapleuron (finely areolate and with longitudinal rugae), and propodeum (areolate). Petiole and ventral face of postpetiole areolate. In dorsal view, promesonotum smooth and shiny medially, as well as postpetiole and gaster; propodeum and petiole areolate.

Lateral margins of head with short decumbent or appressed hairs and long suberect hairs. Posterior margin of head with short subdecumbent hairs. Scapes with appressed hairs. Outer margin of mandible with short decumbent or appressed hairs. Mesosoma with short subdecumbent hairs and long erect or suberect hairs. Petiole and postpetiole with short decumbent hairs and long subdecumbent hairs. Tibia with decumbent to appressed hairs. Gaster with short decumbent or appressed hairs and long suberect or subdecumbent hairs. Color yellowish ferruginous.

#### Description of minor workers.

**Measurements** (n=17): HL 0.35–0.42; HW 0.32–0.38; SL 0.20–0.27; ML 0.08–0.11; EL 0.01–0.02; EM 0.10–0.13; HD 0.22–0.27; WL 0.35–0.44; PSL 0.03–0.06; PW 0.19–0.23; MFL 0.20–0.28; MFW 0.05–0.07; MTL 0.16–0.22, PTL 0.13–0.16; PNL 0.06–0.08; PTH 0.10–0.12; PTW 0.08–0.10; PPL 0.08–0.10; PPNL 0.07–0.09; PPH 0.08–0.09; PPW 0.11–0.14; GL 0.28–0.43; GW 0.22–0.30; CI 86–92; MI 19–28; SI 57–64; MLI 63–78; PPLI 53–77; PPI 120–150; PSI 9–17.

Head longer than wide (CI 86–92), in full-face view nearly subquadrate, about 1.1 times longer than wide, narrowed anteriorly. Posterior margin of head nearly straight, posterolateral corners rounded, lateral margins convex. Mandibles with five teeth. Anterior margin of clypeus slightly concave, and laterally angulate. Antennae with ten segments. Scape fails to reach the posterior margin of head (HL 0.35–0.42, SL 0.20–0.27, SI 57–64). Eyes present, consisting of one ommatidium (EL 0.01–0.02). Supraclypeal area triangular.

In profile, promesonotum weakly convex, metanotal groove deeply impressed. Propodeum about 1.5 times higher than long, dorsal face of propodeum nearly flat, weakly convex, and declining posteriorly, anterodorsal corner rounded, posterodorsal corners each armed with a triangular laminate tooth, declivity concave with thin lateral laminae. Propodeal lobes nearly rounded. Propodeal spiracle rounded and situated slightly above mid-height of sclerite by about half the diameter of the spiracle, and beyond mid-length of sclerite by about 1.5 times the diameter of the spiracle, distance from propodeal spiracle to posterodorsal corner of propodeum almost 1.5 times the diameter of the spiracle (PSL 0.03–0.06), and distance to declivity about half the diameter of the spiracle. In dorsal view, promesonotum about 1.2 times longer than wide, anterior margin nearly rounded and sides convex, and narrowed posteriorly; sides of propodeum weakly convex.

Petiole longer than high (PTL 0.13–0.16, PTH 0.10–0.12) and with relatively short peduncle, ventral face medially convex. Combined outline of dorsal surface of peduncle and anterior face of node medially concave, posterior face of node vertical and slightly convex, anterodorsal and posterodorsal corner rounded, dorsum convex. Subpetiolar process produced as a small denticle, almost as large as the diameter of the propodeal spiracle. Postpetiolar node nearly rounded and slightly lower than petiolar node. In dorsal view, postpetiolar node wider than petiolar node (PTW 0.08–0.10, PPW 0.11–0.14), and petiolar node wider than long (PNL 0.06–0.08, PTW 0.08–0.10), anterior and posterior margin of petiole and postpetiole nearly straight, sides rounded in petiole and postpetiole.

Dorsal surface of head, mandibles, and clypeus smooth and shiny with scattered piligerous punctae on head and mandibles. Gena and frontal lobes with short longitudinal rugae. Mesosoma smooth and shiny, except for katepisternum, metapleuron and propodeum which are areolate. Petiole and ventral face of postpetiole areolate. In dorsal view, promesonotum, postpetiole and gaster smooth and shiny, propodeum and petiole areolate.

Lateral margins of head with short decumbent or appressed hairs posterior margin of head with subdecumbent hairs. Scapes with decumbent or appressed hairs. Outer margin of mandibles with decumbent hairs. Mesosoma with short subdecumbent hairs and long suberect hairs. Petiole and postpetiole with short appressed hairs and long subdecumbent hairs. Tibia with decumbent to appressed hairs. Gaster with abundant, short, subdecumbent to decumbent hairs and dispersed, long, suberect to subdecumbent hairs. Color yellowish ferruginous.

#### Distribution and biology.

*Carebara nosindambo* is a widespread species found in the east, central and southwestern parts of Madagascar (Figure 69). Specimens were found in the following habitats: disturbed gallery, montane forest, ericoid thickets on sand, grassland, littoral forest, littoral rainforest, transition to montane forest, montane forest, montane rainforest, marsh edge, montane shrubland, park/garden, and rainforest. *C. nosindambo* was found at elevations ranging from 10 m to 1580 m, and specimens were collected using maxi-Winkler traps, Berlese funnels, and pitfall traps. Individuals and colonies were found in sifted litter, leaf mold, rotten wood, rotten logs, root matter of *Asplenium* sp. (using Berlese trap), under stones, in ground nests, sifted litter, dead tree stumps, dead twigs above ground, and leaf litter.

#### Comments.

*Carebara nosindambo* can be confused with *C. omasi* but can be separated by the sculpture of the posterolateral portion of the cephalic dorsum, which is sculptured in *C. nosindambo* and lacking sculpture in *C. omasi. C. nosindambo* is widely distributed in the center and east of Madagascar, with one record in the southwestern part of the island, while *C. omasi* is present in central and southwestern parts of Madagascar. Others thirteen species were recorded at the same localities: *C. bara*, *C. betsi*, *C. dota*, *C. grandidieri*, *C. hainteny*, *C. hiragasy*, *C. jajoby*, *C. kabosy*, *C. mahafaly*, *C. omasi*, *C. placida*, *C. raberi*, and *C. sampi*.

*C. nosindambo* have four intermediates in the major worker subcaste (Figure 53). The head is longer than wide, in full-face view nearly subquadrate, the posterolateral corners are rounded in intermediates 1 and 2, and slightly narrowed posteriorly in intermediates 3 and 4. The posterior margin of the head is concave in intermediates 1 and 2, and deeply concave in intemediates 3 and 4. The eyes are small and reduced to one ommatidium in intermediates 1 and 2, with up to twelve ommatidia in intermediate 4. Ocelli are absent in intermediates 1, 2 and 3, but present and well developed in intermediate 4 (one or two ocelli). Reduced flight sclerites are present in intermediate 4. The dorsum of the mesosoma is convex anteriorly and gradually slopes to the declivity in intermediate 4, while in intermediates 1, 2 and 3, the dorsum of the mesosoma is convex and slopes anteriorly, the propodeum is below the promesonotum, the promesonotum is nearly rounded. The propodeum is armed with a pair of small triangular teeth in all intermediates. The anterior margin of the petiole in intermediate 4 is nearly straight, but concave in the center in the other intermediates. The sculpture of the head is nearly the same in all the intermediates, with parallel and longitudinal rugae, the frontal area is smooth and shiny; the sculpture of the mesosoma is also the same in all intermediates. The pilosity on the head and body follows the same pattern, except for intermediate 4, which has more abundant, short appressed hairs on the gaster.

#### Material examined.

**MADAGASCAR: *Antananarivo***: Tsimbazaza, -18.928, 47.527, 1300 m, park/garden, 16.xii.2006, (*B.L. Fisher et al*.); Antananarivo, Forêt de galerie, Telomirahavavy, 23.4 km NNE Ankazobe, -18.12167, 47.20627, 1520 m, disturbed gallery montane forest, 3–4.vi.2008, (*B.L. Fisher et al*.); Antananarivo, Réserve Speciale d’Ambohitantely, -18.18762, 47.28576, 1580 m, montane forest, 08.iii.2012, (*B.L. Fisher et al*.); Antananarivo, Mandraka Park, -18.9019, 47.90786, 1360 m, montane shrubland, 11.iii.2012, (*B.L. Fisher et al*.); ***Fianarantsoa***: Forêt d’Atsirakambiaty, 7.6 km 285° WNW Itremo, -20.59333, 46.56333, 1550 m, montane rainforest, 22–26.i.2003, (*Fisher, Griswold et al.*); Fianarantsoa, Parc National de Ranomafana, Vatoharanana River, 4.1 km 231° SW Ranomafana, -21.29, 47.43333, 1100 m, montane rainforest, 27–31.iii.2003, (*Fisher, Griswold et al.*); Fianarantsoa, 7.6 km 122º Kianjavato, Forêt Classée Vatovavy, -21.4, 47.94, 175 m, rainforest, 6–8.vi.2005, (*B.L. Fisher et al*.); Fianarantsoa, Réserve Forestière d’Agnalazaha, Mahabo, 42.9 km 215° Farafangana, -23.19383, 47.723, 20 m, littoral rainforest, 19.iv.2006, (*B.L. Fisher et al*.); Fianarantsoa, Réserve Speciale Manombo 24.5 km 228° Farafangana, -23.01583, 47.719, 30 m, rainforest, 20.iv.2006, (*B.L. Fisher et al*.); [Imérina], (*Camboué*); Fianarantsoa, Forêt de Vevembe, 66.6 km 293° Farafangana, -22.791, 47.18183, 600 m, rainforest, transition to montane forest, 23.iv.2006, (*B.L. Fisher et al.*); Fianarantsoa, 43 km S Ambalavao, Réserve Andringitra, -22.23333, 47, 825 m, rainforest, 5.x.1993, (*B.L. Fisher*); Fianarantsoa, 45km S. Ambalavao, -22.21667, 47.01667, 785 m, rainforest, 25.ix.1993, (*B.L. Fisher*); Fianarantsoa, Réserve Spéciale Ivohibe, 7.5 km ENE Ivohibe, -22.47, 46.96, 900 m, rainforest, 7–12.x.1997, (*B.L.Fisher* (*Sylvain*)); Fianarantsoa, 9.0 km NE Ivohibe, -22.42667, 46.93833, 900 m, rainforest, 12–17.xi.1997, (*B.L.Fisher* (*Sylvain*)); Fianarantsoa, 43 km S Ambalavao, Réserve Andringitra, -22.23333, 47, 825 m, rainforest, 4.x.1993, (*B.L. Fisher*); ***Toamasina***: Station forestière Tampolo, 10 km NNE Fenoarivo Atn., -17.2825, 49.43, 10 m, littoral rainforest, 10.iv.1997, (*B.L. Fisher*); Toamasina, Analamay, -18.80623, 48.33707, 1068 m, montane rainforest, 21.iii.2004, (*Malagasy ant team*); Toamasina, Forêt Ambatovy, 14.3 km 57° Moramanga, -18.85083, 48.32, 1075 m, montane rainforest, 21.iii.2004, (*Malagasy ant team*); Toamasina, Parcelle K7 Tampolo, -17.28333, 49.41667, 10 m, littoral forest, 16.iv.2004, (*Malagasy ant team*); Toamasina, Parcelle K9 Tampolo, -17.175, 49.268, 10 m, littoral forest, 19.iv.2004, (*Malagasy ant team*); Toamasina, Parcelle E3 Tampolo, -17.28104, 49.43012, 10 m, littoral forest, 14.iv.2004, (*Malagasy ant team*); Toamasina, Torotorofotsy, -18.87082, 48.34737, 1070 m, montane rainforest, marsh edge, 24.iii.2004, (*Malagasy ant team*); Toamasina, Torotorofotsy, -18.87082, 48.34737, 1070 m, montane rainforest, marsh edge, 29.iii.2004, (*Malagasy ant team*); Toamasina, Reserve Betampona, Camp Vohitsivalana, 37.1 km 338° Toamasina, -17.88667, 49.2025, 520 m, rainforest, 1–3.xii.2005, (*B.L. Fisher et al*.); Toamasina, Ile Sainte Marie, Forêt Kalalao, 9.9 km 34° Ambodifotatra, -16.9225, 49.88733, 100 m, rainforest, 24–27.xi.2005, (*B.L. Fisher et al*.); Toamasina, Réserve Analamazaotra, Parc National, Andasibe, -18.92778, 48.41833, 947 m, 7.i.2006, (*A.Ballerio*); Toamasina, Reserve Betampona, Camp Rendrirendry 34.1 km 332° Toamasina, -17.924, 49.19967, 390 m, rainforest, 28.xi.2005, (*B.L. Fisher et al*.); Toamasina, Mahavelona (Foulpointe), -17.66667, 49.5, 1.xi.1985, (*A. Pauly*); Toamasina, Mahavelona (Foulpointe), -17.66667, 49.5, 25.xii.1993, (*A. Pauly*); [Mangoro ufer], (*Sikora*); [Mangoro ufer, 25], (*Sikora*); Toamasina, Forêt Ambatovy, 14.3 km 57° Moramanga, -18.85083, 48.32, 1075 m, montane rainforest, 18.xii.2004, (B.L. Fisher); Toamasina, Ambatovy, 12.4 km NE Moramanga, -18.84773, 48.29568, 1000 m, grassland, 5–8.iii.2007, (*B.L. Fisher et al*.); Toamasina, Ambatovy, 12.4 km NE Moramanga, -18.84963, 48.2947, 1010 m, montane rainforest, 3–6.iii.2007, (*B.L. Fisher et al*.); Toamasina, Ambatovy, 12.4 km NE Moramanga, -18.83937, 48.30842, 1080 m, montane rainforest, 4–7.iii.2007, (*B.L. Fisher et al*.); Toamasina, 6.3 km S Ambanizana, Andranobe, -15.6813, 49.958, 25 m, rainforest, 14.xi.1993, (*B.L. Fisher*); Toamasina, 5.3 km SSE Ambanizana, Andranobe, -15.67133, 49.97395, 425 m, rainforest, 21.xi.1993, (*B.L. Fisher*); Toamasina, Station forestière Tampolo, 10 km NNE Fenoarivo Atn., -17.2825, 49.43, 10 m, littoral rainforest, 4.iv.1997, (*B.L. Fisher*); Toamasina, Station forestière Tampolo, 10 km NNE Fenoarivo Atn., -17.2825, 49.43, 10 m, littoral rainforest, 6.iv.1997, (*B.L. Fisher*); Toamasina, Forêt classée Sandranantitra, -18.04833, 49.09167, 450 m, rainforest, 21–24.i.2009, (*H.J.Ratsirarson*); Toamasina, Forêt classée Sandranantitra, -18.04833, 49.09167, 450 m, rainforest, 18–21.i.1999, (*H.J.Ratsirarson*); Toamasina, Forêt classée Andriantantely, -18.695, 48.81333, 530 m, rainforest, 7–10.xii.1998, (*H.J.Ratsirarson*); Toamasina, Forêt classée Sandranantitra, -18.04833, 49.09167, 450 m, rainforest, 18–24.i.1999, (*H.J.Ratsirarson*); Toamasina, Forêt classée Andriantantely, -18.695, 48.81333, 530 m, rainforest, 4–7.xii.1998, *(H.J.Ratsirarson*); Toamasina, Sahafina forest 11.4 km W Brickaville, -18.81445, 48.96205, 140 m, rainforest, 13–14.xii.2007. (*B.L. Fisher et al*.); Toamasina, Parc National de Zahamena, Tetezambatana forest, near junction of Nosivola and Manakambahiny Rivers, -17.74298, 48.72936, 860 m, rainforest, 18–19.ii.2009, (*B.L. Fisher et al*.); Toamasina, Parc National de Zahamena, Onibe River, -17.75908, 48.85468, 780 m, rainforest, 21–23.ii.2009, (*B.L. Fisher et al*.); Toamasina, Réserve Spéciale Ambatovaky, Sandrangato river, -16.7702, 49.26638, 470 m, rainforest, 23.ii.2010, (*B.L. Fisher et al*.); Toamasina, Corridor Forestier Analamay-Mantadia, Ambatoharanana, -18.80388, 48.40506, 1013 m, rainforest, 12–19.xii.2012, (*B.L. Fisher et al*.); Toamasina, Forêt classée Sandranantitra, -18.04833, 49.09167, 450 m, rainforest, 21–24.i.1999, (*H.J.Ratsirarson*); Toamasina, Ankerana, -18.4104, 48.8189, 855 m, rainforest, 27.i.2012, (*B.L. Fisher et al*.); Toamasina, Ankerana, -18.4104, 48.8189, 855 m, rainforest, 25.i.2012, (*B.L. Fisher et al*.); Toamasina, Ankerana, -18.4104, 48.8189, 855 m, rainforest, 22–27.i.2012, (*B.L. Fisher et al*.); Toamasina, Ankerana, -18.40062, 48.81311, 865 m, rainforest, 17–18.i.2012, (*B.L. Fisher et al*.); Toamasina, Ankerana, -18.4061, 48.82029, 725 m, rainforest, 16.i.2012, (*B.L. Fisher et al*.); Toamasina, Ankerana, -18.40829, 48.82107, 750 m, rainforest, 21–22.i.2012, (*B.L. Fisher et al*.); Toamasina, Ankerana, -18.4061, 48.82029, 725 m, rainforest, 16–21.i.2012, (*B.L. Fisher et al*.); Toamasina, Ankerana, -18.40829, 48.82107, 750 m, rainforest, 21–26.i.2012, (*B.L. Fisher et al*.); Toamasina, Corridor Forestier Analamay-Mantadia, Ambatoharanana, -18.80424, 48.40081, 968 m, rainforest, 12–19.xii.2012, (*B.L. Fisher et al*.); Toamasina, Corridor Forestier Analamay-Mantadia, Ambohibolakely, -18.76087, 48.37128, 1044 m, rainforest, 29.xi.2012, (*B.L. Fisher et al*.); Toamasina, Corridor Forestier Analamay-Mantadia, Ambohibolakely, -18.76131, 48.36437, 983 m, rainforest, 26.xi.2012, (*B.L. Fisher et al*.); Toamasina, Corridor Forestier Analamay-Mantadia, Ambohibolakely, -18.77908, 48.36628, 1014 m, rainforest, 28.xi.2012, (*B.L. Fisher et al*.); Toamasina, Corridor Forestier Analamay-Mantadia, Ambatoharanana, -18.80398, 48.40358, 1064 m, rainforest, 12–19.xii.2012, (*B.L. Fisher et al*.); Toamasina, Corridor Forestier Analamay-Mantadia, Tsaravoniana, -18.76465, 48.41938, 1039 m, rainforest, 2–7.xii.2012, (*B.L. Fisher et al*.); Toamasina, Corridor Forestier Analamay-Mantadia, Tsaravoniana, -18.76124, 48.42134, 939 m, rainforest, 2–7.xii.2012, (*B.L. Fisher et al*.); Toamasina, Corridor Forestier Analamay-Mantadia, Tsaravoniana, -18.76465, 48.41938, 1039 m, rainforest, 4–5.xii.2012, (*B.L. Fisher et al*.); Toamasina, Corridor Forestier Analamay-Mantadia, Ambohibolakely, -18.76131, 48.36437, 983 m, rainforest, 23–28.xi.2012, (*B.L. Fisher et al*.); Toamasina, Corridor Forestier Analamay-Mantadia, Ambohibolakely, -18.76087, 48.37128, 1044 m, rainforest, 23–28.xi.2012, (*B.L. Fisher et al*.); ***Toliara***: Parc National Andohahela, Col de Tanatana, 33.3 km NW Tolagnaro, -24.7585, 46.85367, 275 m, rainforest, 23.xi.2006, (*B.L. Fisher et al*.); Toliara, 11 km NW Enakara, Réserve Andohahela, -24.56667, 46.83333, 800 m, rainforest, 17.xi.1992, (*B.L. Fisher*); Toliara, 10 km NW Enakara, Réserve Andohahela, -24.56667, 46.81667, 430 m, rainforest, 22.xi.1992, (*B.L. Fisher*); Toliara, Mandena, 8.4 km NNE 30° Tolagnaro, -24.95167, 47.00167, 20 m, littoral rainforest, 20.xi.1998, (*B.L. Fisher*); Toliara, 2.7 km WNW 302° Ste. Luce, -24.77167, 47.17167, 20 m, littoral rainforest, 9–11.xii.1998, (*B.L.Fisher (J.-Baptiste*)); Toliara, Forêt de Petriky, 12.5 km W 272° Tolagnaro, -25.06167, 46.87, 10 m, littoral rainforest, 22.xi.1998, (*B.L. Fisher*); Toliara, Parc National Andohahela, Col de Tanatana, 33.3 km NW Tolagnaro, -24.7585, 46.85367, 275 m, rainforest, 28.xi.2006, (*B.L. Fisher et al*.); Toliara, Manatantely, 8.9 km NW Tolagnaro, -24.9815, 46.92567, 100 m, rainforest, 27–28.xi.2006, (*B.L. Fisher et al*.); Toliara, Forêt Ivohibe 55.0km N Tolagnaro, -24.569, 47.204. 200 m, rainforest, 2–4.xii.2006, (*B.L. Fisher et al*.); Toliara, Parc National Andohahela, Col de Tanatana, 33.3km NW Tolagnaro, -24.7585, 46.85367, 275 m, rainforest, 22–24.xi.2006, (*B.L. Fisher et al*.); Toliara, Grand Lavasoa, 25.9km W Tolagnaro, -25.08767, 46.749, 450 m, rainforest, 30.xi.-2.xii.2006, (*B.L. Fisher et al*.); Toliara, Réserve Spéciale Kalambatritra, Ambinanitelo, -23.45373, 46.45773, 1345 m, grassland, 11.ii.2009, (*B.L. Fisher et al*.); Toliara, Parc National d’Andohahela, Manampanihy River, 5.4 km 113° ESE Mahamavo, 36.7 km 343° NNW Tolagnaro, -24.76389, 46.76683, 650 m, rainforest, 24.i.2002, (*Fisher, Griswold et al.*); Toliara, Forêt Classée d’Analavelona, 29.2 km 343° NNW Mahaboboka, -22.675, 44.19, 1100 m, montane rainforest, 18–22.ii.2003, (*Fisher, Griswold et al.*).

### Carebara omasi

Taxon classificationAnimaliaHymenopteraFormicidae

Azorsa & Fisher sp. n.

http://zoobank.org/0F423ABB-516D-4243-8052-AC1D681F44EC

#### Holotype.

(major worker), MADAGASCAR, Toliara, Réserve Spéciale d’Ambohijanahary, Forêt d’Ankazotsihitafototra, 34.6 km 314° NW Ambaravaranala, -18.26, 45.41833, 1100 m, montane rainforest, 16.i.2003, (*Fisher, Griswold et al.*). Collection code BLF07086, (CASC: CASENT0028870). **Paratypes**: (30 major workers and 16 minor workers), with same data as holotype, 17 major workers (BMNH: CASENT0028980, CASC: CASENT0029010, CASENT0029145, CASENT0028999, CASENT0029011, CASENT0029140, CASENT0028973, CASENT0028998, CASENT0029040, CASENT0029146, CASENT0029024, CASENT0028886, CASENT0028879, CASENT0028869, MCZ: CASENT0028945, MHNG: CASENT0028955, NHMB: CASENT0028987), and 16 minor workers (BMNH: CASENT0028987, CASC: CASENT0028928, CASENT0029047, CASENT0029035, CASENT0028947, CASENT0029042, CASENT0029043, CASENT0029045, CASENT0029046, CASENT0028936, CASENT0029031, CASENT0029087, CASENT0028940, MCZ: CASENT0028940, MHNG: CASENT0029086, NHMB: CASENT0029088). 13 major workers from: Toliara, Réserve Spéciale d’Ambohijanahary, Forêt d’Ankazotsihitafototra, 35.2 km 312° NW Ambaravaranala, -18.26667, 45.40667, 1050 m, montane rainforest, 13–17.i.2003, (*Fisher, Griswold et al.*), BLF07020, 4 major workers (CASC: CASENT0027925, CASENT0027900, CASENT0027924, CASENT0027810), BLF07132, 1 major worker (CASC: CASENT0496795), BLF07148, 2 major workers (CASC: CASENT0485919, CASENT0485920).

#### Diagnosis.

Antennae ten-segmented. **Major**: Head nearly subrectangular, longer than wide, lateral margins straight and parallel, posterolateral corners well developed and rounded, posterior margin of head deeply concave; dorsum of propodeum flat, posterodorsal corner with a pair of triangular teeth; gaster with very short decumbent or appressed hairs, and sparse, longer suberect hairs. **Minor**: Head longer than wide, posterior margin of head nearly straight; dorsum of propodeum weakly convex, posterodorsal corner with a pair of laminate, small, upwardly directed triangular teeth; gaster with short decumbent hairs and sparse, longer suberect to subdecumbent hairs.

**Figure 55.**
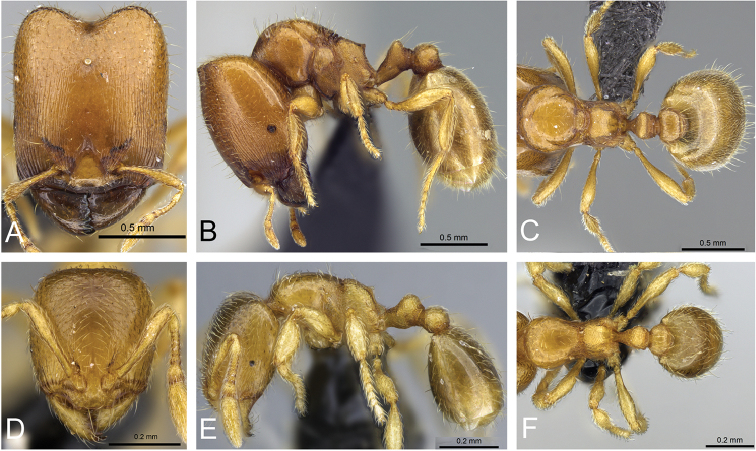
*Carebara omasi*–holotype. Major worker, CASENT0028870: **A** head in full-face view **B** body in profile view **C** body in dorsal view. Minor worker, CASENT0028947: **D** head in full-face view **E** body in profile view **F** body in dorsal view.

**Figure 56.**
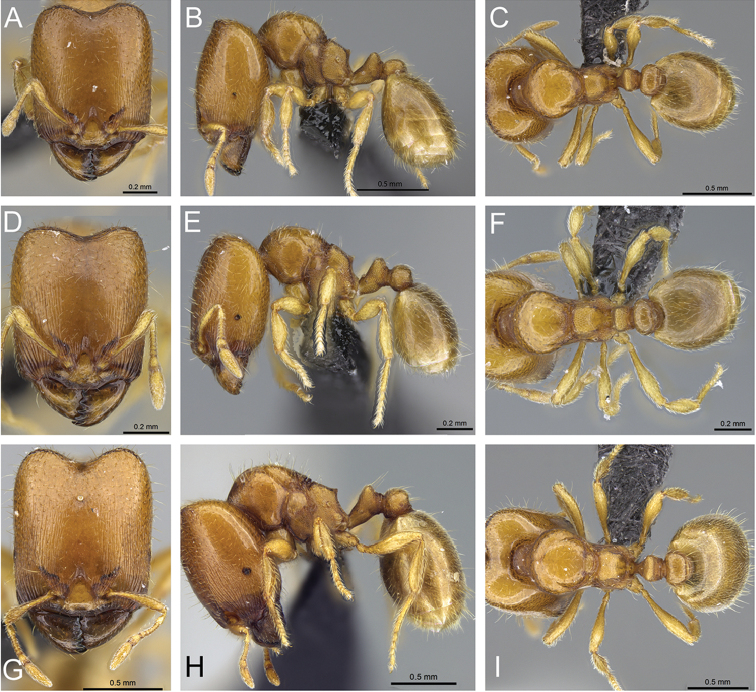
Intermediates of *Carebara omasi*. Major workers, CASENT0028955: **A** head in full-face view **B** body in profile view **C** body in dorsal view. CASENT0028862: **D** head in full-face view **E** body in profile view **F** body in dorsal view. CASENT0028870 **G** head in full-face view **H** body in profile view **I** body in dorsal view.

#### Description of major workers.

**Measurements** (n=20): HL 0.62–1.10 (1.04); HW 0.54–0.94 (0.87); SL 0.27–0.42 (0.38); ML 0.15–0.27 (0.23); EL 0.01–0.15 (0.05); EM 0.18–0.29 (0.28); HD 0.36–0.68 (0.65); WL 0.52–1.02 (0.92); PSL 0.07–0.15 (0.13); PW 0.31–0.58 (0.55); MFL 0.30–0.58 (0.54), MFW 0.08–0.13 (0.12); MTL 0.24–0.46 (0.43), PTL 0.20–0.37 (0.35); PNL 0.07–0.14 (0.12); PTH 0.15–0.28 (0.24); PTW 0.13–0.24 (0.22); PPL 0.12–0.23 (0.19); PPNL 0.11–0.20 (0.17); PPH 0.08–0.26 (0.25); PPW 0.18–0.33 (0.30); GL 0.43–1.01 (0.92); GW 0.39–0.87 (0.80); CI 82–89 (84); MI 22–27 (22); SI 37–47 (37); MLI 55–64 (62); PPLI 54–67 (54); PPI 125–143 (136); PSI 12–16 (15).

Head slightly longer than wide (CI 82–89), in full-face view nearly subrectangular, about 1.2 times longer than wide. Posterior margin of head deeply concave, posterolateral corners well developed and rounded, lateral margins straight and parallel. Mandibles with six teeth. Anterior margin of clypeus nearly straight, and laterally convex. Ocelli present or absent. Antennae with ten segments. Scapes short (HL 0.62–1.10, SL 0.27–0.42, SI 0.01–0.15). Eyes present, consisting of one to 15 ommatidia (EL 0.01–0.15). Supraclypeal area triangular and well defined.

In profile view, posterolateral corner of head with a small angulate tooth resembling a horn. Promesonotum high and nearly rounded, promesonotal suture present or absent, when present, weakly impressed on dorsum, metanotal groove deeply impressed. Propodeum lower than promesonotum, and about 1.5 times higher than long, dorsal face of propodeum flat and declining posteriorly, posterodorsal corners of propodeum each armed with an acute triangular tooth, declivity concave with thin lateral laminae. Propodeal lobes triangular. Propodeal spiracle nearly oval and situated slightly above mid-height of sclerite, and beyond mid-length of sclerite by about half the diameter of the spiracle, distance from propodeal spiracle to posterodorsal corner of propodeum almost 3.2 times the diameter of the spiracle (PSL 0.04–0.07), and distance to declivity about 2.2 times the diameter of the spiracle. In dorsal view, promesonotum about as long as wide, anterior margin and sides rounded, and narrowed posteriorly; sides of propodeum straight.

Petiole longer than high (PTL 0.20–0.37, PTH 0.15–0.28) and with relatively long peduncle, ventral face weakly convex at center. Combined outline of dorsal surface of peduncle and anterior face of node nearly straight, weakly concave, posterior face of node vertical and slightly convex, anterodorsal corner weakly convex, posterodorsal corner rounded, dorsum narrow and rounded. Subpetiolar process produced as a small denticle, slightly smaller than the diameter of the propodeal spiracle. Postpetiolar node rounded and lower than petiolar node. In dorsal view, petiolar node almost as wide as postpetiolar node (PTW 0.13–0.24, PPW 0.18–0.33) and petiolar node wider than long (PNL 0.07–0.14, PTW 0.13–0.24), anterior margin of petiole weakly concave, and concave in postpetiole, posterior margin of petiole and postpetiole straight, sides rounded in petiole and postpetiole.

Dorsal surface of mandibles, clypeus and frons smooth and shiny, with scattered piligerous punctae on head and mandibles. Head with fine longitudinal rugae directed to posterior margin of head, and rugoreticulate close to posterior margin of head. Posterolateral portion of cephalic dorsum smooth and shiny. Sides of pronotum finely areolate, mesonotum and anepisternum smooth and shiny, katepisternum and metapleuron areolate-rugose. Pronotum finely areolate. Petiole, ventral face and sides of postpetiole areolate. In dorsal view, promesonotum, postpetiole and gaster smooth and shiny, propodeum and petiole areolate.

Lateral margins of head with short appressed hairs and long erect to suberect hairs, posterior margin with subdecumbent hairs. Scapes with appressed hairs. Outer margin of mandibles with short appressed hairs. Mesosoma with short suberect hairs and long erect hairs. Petiole and postpetiole with short decumbent hairs and long subdecumbent hairs. Tibia with appressed hairs. Gaster with short to very short appressed or decumbent hairs and dispersed, long suberect hairs. Color yellowish ferruginous.

#### Description of minor workers.

**Measurements** (n=9): HL 0.35–0.43; HW 0.31– 0.38; SL 0.21–0.27; ML 0.09–0.11; EL 0.02; EM 0.09–0.12; HD 0.21–0.27; WL 0.35–0.44; PSL 0.04–0.06; PW 0.19–0.24; MFL 0.22–0.28; MFW 0.05–0.07; MTL 0.15–0.21, PTL 0.12–0.15; PNL 0.06–0.08; PTH 0.10–0.13; PTW 0.08–0.11; PPL 0.07–0.10; PPNL 0.06–0.09; PPH 0.07–0.09; PPW 0.11–0.14; GL 0.30–0.42, GW 0.22–0.32; CI 86–93; MI 23–26; SI 60–65; MLI 66–78, PPLI 57–77; PPI 120–147; PSI 11–15.

Head slightly longer than wide (CI 86–93), in full-face view nearly subquadrate, about 1.1 times longer than wide. Posterior margin of head nearly straight, weakly concave, posterolateral corners rounded, lateral margins convex. Mandibles with five teeth. Anterior margin of clypeus slightly concave, nearly straight, and laterally angulate. Antennae with ten segments. Scape fails to reach posterior margin of head (HL 0.35–0.43, SL 0.21–0.27, SI 60–65). Eyes present, consisting of one ommatidium (EL 0.02). Supraclypeal area triangular but poorly defined.

In profile, promesonotum weakly convex, nearly flat, metanotal groove deeply impressed. Propodeum about 1.4 times higher than long, dorsal face of propodeum slightly convex, and declining posteriorly, propodeum armed, posterodorsal corners each armed with a laminate triangular and small tooth, declivity weakly convex, nearly flat, with thin lateral laminae. Propodeal lobes triangular. Propodeal spiracle rounded and situated above mid-height of sclerite by about 1.5 times the diameter of the spiracle, and beyond mid-length of sclerite by about two times the diameter of the spiracle, distance from propodeal spiracle to posterodorsal corner of propodeum almost twice the diameter of the spiracle (PSL 0.04–0.06), and distance to declivity same as the diameter of the spiracle. In dorsal view, promesonotum about 1.1 times longer than wide, anterior margin weakly convex, sides convex and narrowed posteriorly; sides of propodeum weakly convex.

Petiole longer than high (PTL 0.12–0.15, PTH 0.10–0.13) and with relatively long peduncle, ventral face medially convex. Combined outline of dorsal surface of peduncle and anterior face of node medially concave, posterior face of node slightly convex, anterodorsal and posterodorsal corner rounded, dorsum convex. Subpetiolar process produced as a small denticle, almost the same as half the diameter of the propodeal spiracle. Postpetiolar node nearly rounded and lower than petiolar node. In dorsal view, postpetiolar node wider than petiolar node (PTW 0.08–0.11, PPW 0.11–0.14), and petiolar node wider than long (PNL 0.06–0.08, PTW 0.08–0.11), anterior and posterior margins of petiole and postpetiole nearly straight, sides rounded in petiole and postpetiole.

Dorsal surface of head, mandibles, and clypeus smooth and shiny with scattered piligerous punctae on head and mandibles. Gena and frontal lobes with short longitudinal rugae. Mesosoma smooth and shiny except for mesopleuron and propodeum (areolate) and metapleuron (areolate-rugose). Petiole and ventral face of postpetiole areolate. In dorsal view, promesonotum, postpetiole and gaster smooth and shiny, propodeum and petiole areolate.

Lateral margins of head with decumbent hairs, posterior margin with subdecumbent hairs. Scapes with appressed hairs. Outer margin of mandibles with decumbent hairs. Mesosoma with short and long suberect hairs. Petiole and postpetiole with short appressed hairs and longer subdecumbent hairs. Tibia with appressed hairs. Gaster with short decumbent to appressed hairs and long subdecumbent hairs. Color yellowish ferruginous.

#### Distribution and biology.

*Carebara omasi* was collected in the central and southwestern parts of Madagascar (Figure 69). This species occurs in disturbed gallery montane forest, montane rainforest and *Uapaca* woodland, and was collected at elevations ranging from 1050 m to 1491 m. Individuals and colonies were found in rotten logs, leaf mold and rotten wood. They were collected using maxi-Winkler traps.

#### Comments.

*Carebara omasi* can be confused with *C. nosindambo* but can be separated by the sculpture of the posterolateral portion of the cephalic dorsum, which is lacking sculpture in *C. omasi* and is sculptured in *C. nosindambo. C. omasi* is present only in central and southwestern areas of Madagascar while *C. nosindambo* is widespread throughout most of Madagascar. *C. omasi* is endemic to the center of Madagascar, but there are also records from southwestern Madagascar, other six species were recorded at the same localities: *C. bara*, *C. betsi*, *C. grandidieri*, *C. hiragasy*, *C. nosindambo*, and *C. sampi*.

*C. omasi* have three intermediates in the major worker subcaste (Figure 56). The shape of the posterolateral corner and posterior margin of the head varies little among all intermediates. Eyes are reduced to one to four ommatidia in all intermediates, but intermediate 3 presents up to 40 ommatidia. Ocelli are absent in intermediate 1, but present and well developed in intermediates 2 and 3. Intermediates 2 and 3 have reduced flight sclerites. The dorsum of the mesosoma is convex anteriorly and gradually slopes to the declivity in intermediate 3, but in intermediates 1 and 2 the dorsum of the mesosoma is nearly rounded anteriorly, the propodeum is below the promesonotum. The propodeum is armed with a pair of triangular teeth in all intermediates. The petiole and postpetiole do not vary much in all intermediates, the combined outline of dorsal surface of peduncle and anterior face of node is slightly concave in intermediate 1 and 2, nearly straight in intermediate 3, the petiolar node is slightly thinner in intermediate 3. Pilosity is nearly the same in all intermediates, but intermediate 3 has more abundant, short appressed hairs on the gaster. The sculpture of the head and mesosoma is almost the same in all intermediates.

#### Additional material examined.

**MADAGASCAR**: ***Antananarivo***: Forêt de galerie, Andranorovitra, 24.0 km NNE Ankazobe, -18.11243, 47.19757, 1491 m, disturbed gallery montane forest, 2–3.vi.2008, (*B.L. Fisher et al*.); Antananarivo, Ankalalahana, -19.00659, 47.1122, 1375 m, *Uapaca* woodland, 29–31.iii.2011, (*B.L. Fisher et al*.); ***Toliara***: Forêt Classée d’Analavelona, 29.4 km 343° NNW Mahaboboka, -22.675, 44.18667, 1050 m, montane rainforest, 21.ii.2003, (*Fisher, Griswold et al.*); Toliara, Réserve Spéciale d’Ambohijanahary, Forêt d’Ankazotsihitafototra, 35.2 km 312° NW Ambaravaranala, -18.26667, 45.40667, 1050 m, montane rainforest, 13–17.i.2003, (*Fisher, Griswold et al.*); Toliara, Forêt Classée d’Analavelona, 29.2 km 343° NNW Mahaboboka, -22.675, 44.19, 1100 m, montane rainforest, 18–22.ii.2003, (*Fisher, Griswold et al.*).

### Carebara placida

Taxon classificationAnimaliaHymenopteraFormicidae

Azorsa & Fisher sp. n.

http://zoobank.org/914229BC-BFC4-4CC2-9A62-60BC61051313

#### Holotype.

(major worker), MADAGASCAR, Fianarantsoa, Forêt de Vevembe, 66.6 km 293° Farafangana, -22.791, 47.18183, 600 m, rainforest, transition to montane forest, 23.iv.2006, (*B.L. Fisher et al*.). Collection code BLF14122, (CASC: CASENT0070818). **Paratypes**: (1 major worker and 1 minor worker), with same data as holotype, 1 major worker (CASC: CASENT0070817) and 1 minor worker (CASC: CASENT0070818).

#### Diagnosis.

Antennae ten-segmented. **Major**: Head subrectangular, longer than wide, lateral margins slightly convex, posterior margin of head deeply concave; dorsum of propodeum flat, posterodorsal corner of propodeum angulate; head with longitudinal rugae. **Minor**: Head nearly subquadrate, slightly longer than wide; dorsum of promesonotum and propodeum weakly convex, posterodorsal corner convex; petiolar node roundly prominent; gaster with abundant short suberect hairs.

**Figure 57.**
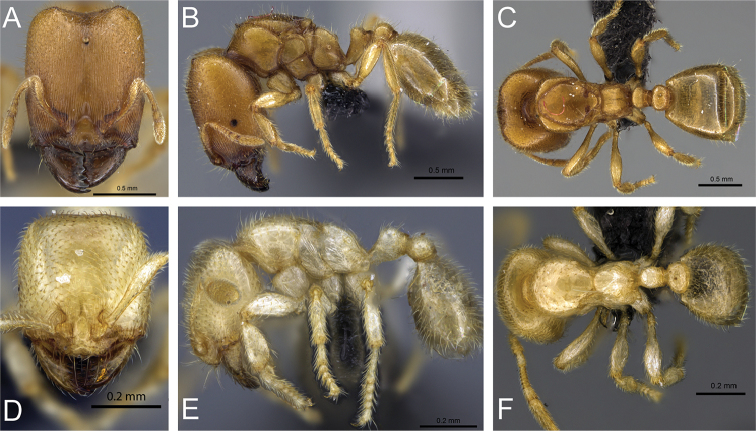
*Carebara placida*–holotype. Major worker, CASENT0070818: **A** head in full-face view **B** body in profile view **C** body in dorsal view. Minor worker, CASENT0070817: **D** head in full-face view **E** body in profile view **F** body in dorsal view.

**Figure 58.**
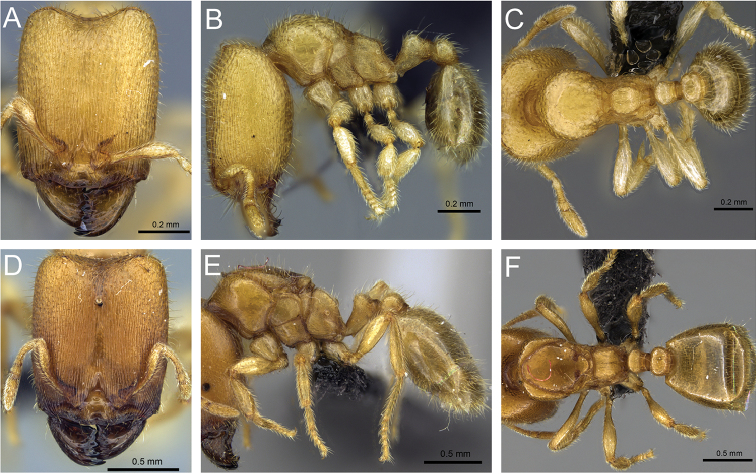
Intermediates of *Carebara placida*. Major workers, CASENT0845056: **A** head in full-face view **B** body in profile view **C** body in dorsal view. CASENT0070818: **D** head in full-face view **E** body in profile view **F** body in dorsal view.

#### Description of major workers.

**Measurements** (n=2): HL 0.73–1.20 (0.73); HW 0.55–0.95 (0.55); SL 0.29–0.44 (0.29); ML 0.14–0.29 (0.14); EL 0.01–0.05 (0.01); EM 0.24–0.39 (0.24); HD 0.38–0.68 (0.38); WL 0.62–1.16 (0.62); PSL 0.04–0.11 (0.04); PW 0.36–0.64 (0.36); MFL 0.33–0.65 (0.33); MFW 0.09–0.15 (0.09); MTL 0.25–0.54 (0.25); PTL 0.18–0.36 (0.18); PNL 0.10–0.20 (0.10); PTH 0.14–0.25 (0.14); PTW 0.12–0.24 (0.12); PPL 0.12–0.20 (0.12); PPNL 0.12–0.18 (0.12); PPH 0.12–0.24 (0.12); PPW 0.17–0.29 (0.17); GL 0.39 –1.05 (0.39); GW 0.40–0.87 (0.40); CI 76–79 (76); MI 19–24 (19); SI 37–40 (40); MLI 60–68 (60); PPLI 56–67 (67); PPI 121–142 (142); PSI 7–12 (7).

Head slightly longer than wide (CI 76–79), in full-face view nearly subrectangular, about 1.2 times longer than wide, slightly narrowed anteriorly. Posterior margin of head deeply concave, posterolateral corners rounded, lateral margins slightly convex. Mandibles with six teeth. Anterior margin of clypeus slightly concave in the middle, nearly straight, and laterally convex. Ocelli present or absent. Antennae with ten segments. Scapes short (HL 0.73–1.20, SL 0.29–0.44, SI 37–40). Eyes present, consisting of fifteen to thirty ommatidia (EL 0.01–0.05). Supraclypeal area acutely triangular and well defined.

In profile view, posterolateral corner of head with a small triangular tooth resembling a horn. Promesonotum convex, (metanotum present in larger major workers), dorsum of mesonotum slightly convex, promesonotal suture present on dorsum, metanotal groove deeply impressed, propodeum lower than promesonotum, and about 1.6 times higher than long, dorsal face of propodeum slightly convex, nearly flat, and declining posteriorly; posterodorsal corners of propodeum angulate (more noticeable in larger major workers); anterodorsal corner nearly rounded; declivity concave with thin lateral laminae. Propodeal lobes convex. Propodeal spiracle rounded and situated above mid-height of sclerite, and beyond mid-length of sclerite by about two times the diameter of the spiracle, (closer to mid-length and mid-height in larger major workers), distance from propodeal spiracle to posterodorsal corner of propodeum about twice the diameter of the spiracle (PSL 0.04–0.11), and distance to declivity less than half the diameter of the spiracle. In dorsal view, promesonotum about as long as wide, anterior margin and sides rounded, sides weakly convex in larger major workers; sides of propodeum convex, and straight in larger major workers.

Petiole longer than high (PTL 0.18–0.36, PTH 0.14–0.25) and with relatively long peduncle, ventral face slightly convex, nearly flat. Combined outline of dorsal surface of peduncle and anterior face of node medially concave, posterior face of node convex, anterodorsal and posterodorsal corner convex, dorsum rounded. Subpetiolar process produced as denticle, barely larger than the diameter of the propodeal spiracle. Postpetiolar node nearly rounded and slightly lower than petiolar node. In dorsal view, postpetiolar node slightly wider than petiolar node (PTW 0.12–0.24, PPW 0.17–0.29) and petiolar node wider than long (PNL 0.10–0.20, PTW 012–0.24), anterior margin of petiole convex, posterior margin straight, anterior and posterior margin of postpetiole straight, sides nearly rounded in petiole and postpetiole.

Dorsal surface of mandibles, clypeus and supraclypeal area smooth and shiny with scattered piligerous punctae on head and mandibles. Head with longitudinal rugae directed to the posterior margin, gena with well-developed and longitudinal rugae. Posterolateral portion of cephalic dorsum with longitudinal rugae, smooth and shiny on larger major workers. Mesosoma smooth and shiny, except for katepisternum and sides of propodeum (areolate) and metapleuron (finely areolate and with longitudinal rugae). Petiole and ventral face of postpetiole finely areolate. In dorsal view, mesosoma, petiole, postpetiole and gaster smooth and shiny.

Lateral margins of the head with short and long erect to suberect hairs, posterior margin with suberect hairs. Scapes with abundant decumbent hairs. Outer margin of mandibles with short decumbent and appressed hairs. Mesosoma with short and long erect to suberect hairs. Petiole and postpetiole with short subdecumbent hairs and longer suberect hairs. Tibia with decumbent hairs. Gaster with short decumbent hairs, and long suberect to subdecumbent hairs. Color yellowish ferruginous.

#### Description of minor workers.

**Measurements** (n=2): HL 0.40–0.41; HW 0.38; SL 0.26–0.27; ML 0.10–0.13; EL 0.01; EM 0.13; HD 0.25; WL 0.45–0.47; PSL 0.02–0.03; PW 0.22–0.24; MFL 0.26; MFW 0.07–0.08; MTL 0.21; PTL 0.13–0.14; PNL 0.08–0.09; PTH 0.11; PTW 0.08–0.09; PPL 0.09; PPNL 0.09; PPH 0.09; PPW 0.12–0.13; GL 0.32–0.34, GW 0.30–0.33; CI 93–95; MI 24–33; SI 65–66; MLI 68; PPLI 64–69; PPI 133–163; PSI 5–8.

Head slightly longer than wide (CI 93–95), in full-face view nearly subquadrate, about 1.1 times longer than wide. Posterior margin of head straight, posterolateral corners rounded, lateral margins weakly convex. Mandibles with five teeth. Anterior margin of clypeus nearly straight, and laterally angulate. Antennae with ten segments. Scape fails to reach the posterior margin of head (HL 0.40–0.41, SL 0.26–0.27, SI 65–66). Eyes present, consisting of one ommatidium (EL 0.01). Supraclypeal area triangular but poorly defined.

In profile, promesonotum weakly convex, nearly straight, metanotal groove deeply impressed. Propodeum about 1.2 times higher than long, dorsal face of propodeum weakly convex, declining posteriorly, posterodorsal corner unarmed (nearly convex), declivity slightly concave, nearly flat, with thin lateral laminae. Propodeal lobes convex. Propodeal spiracle rounded and situated above mid-height of sclerite, and beyond mid-length of sclerite by about two times the diameter of the spiracle, distance from propodeal spiracle to posterodorsal corner of propodeum and distance to declivity same as the diameter of the spiracle (PSL 0.02–0.03). In dorsal view, promesonotum slightly longer than wide, anterior margin rounded, sides convex, narrowed posteriorly; sides of propodeum convex.

Petiole longer than high (PTL 0.13–0.14, PTH 0.11) and with relatively short peduncle, ventral face medially convex. Petiolar node thick, combined outline of dorsal surface of node and anterior face of node medially concave, posterior margin slightly convex, anterodorsal corner convex and posterodorsal corner rounded, dorsum nearly rounded. Subpetiolar process produced as a small denticle, almost the same as the diameter of the propodeal spiracle. Postpetiolar node strongly convex and lower than petiolar node. In dorsal view, postpetiolar node wider than petiolar node (PTW 0.08–0.09, PPW 0.12–0.13), and petiolar node as wide as long (PNL 0.08–0.09, PTW 0.08–0.09), anterior margin of petiole convex, posterior margin nearly straight, anterior and posterior margins of postpetiole straight, sides convex in petiole and postpetiole.

Dorsal surface of head, mandibles and clypeus smooth and shiny with scattered piligerous punctae on head and mandibles. Gena and frontal lobes with short longitudinal rugae. Mesosoma smooth and shiny, except for katepisternum and metapleuron which are finely areolate. Petiole and ventral face of postpetiole finely areolate. In dorsal view, mesosoma, petiole, postpetiole and gaster smooth and shiny.

Lateral margins, posterior margin of head, scape and outer margin of mandible with suberect to subdecumbent hairs. Mesosoma with erect to suberect hairs. Petiole and postpetiole with short and long subdecumbent hairs. Tibia with decumbent hairs. Gaster with abundant suberect hairs. Color yellowish.

#### Distribution and biology.

*Carebara placida* is known only from one locality in southeastern Madagascar (Figure 69). Individuals of this species occur in rainforest and transition to montane forest at an elevation of 600 m, and were collected under stones.

#### Comments.

*Carebara placida* can be separated from other species by the next combination of characters: dorsum of head with well-defined longitudinal rugae, while others species are either unsculptured or with irregular longitudinal rugae. *C. placida* is known only from one locality in southeastern Madagascar. Other six species were recorded at the same locality: *C. dota*, *C. grandidieri*, *C. hainteny*, *C. hiragasy*, *C. jajoby*, and *C. nosindambo*.

*C. placida* have two intermediates in the major worker subcaste (Figure 58). The posterolateral corner of head in intermediates slightly narrowed posteriorly. The posterior margin of the head is weakly to deeply concave in both intermediates. Eyes are small and reduced to one ommatidium in intermediate 1, and with 14 ommatidia in intermediate 2. Ocelli are absent in intermediate 1, but present and well developed in intermediate 2 (one ocellus). Reduced flight sclerites are present in intermediate 2. The dorsum of the promesonotum is high and nearly rounded in intermediate 1, and nearly convex in intermediate 2. The combined outline of dorsal surface of peduncle and anterior face of node medially concave in intermediate 2, and slightly concave, nearly straight in intermediate 1. The sculpture of the head in both intermediates is the same. The pilosity on the head, mesosoma, petiole and postpetiole is the same in both intermediates. The pilosity on the gaster of intermediate 1 is composed of short subdecumbent hairs and sparse, long suberect hairs, while intermediate 2 has very short decumbent to appressed hairs and sparse, long suberect hairs.

### Carebara raberi

Taxon classificationAnimaliaHymenopteraFormicidae

Azorsa & Fisher sp. n.

http://zoobank.org/BE26408F-2306-4FAF-90C6-240727EBF78A

#### Holotype.

(major worker), MADAGASCAR, Mahajanga, Réserve Spéciale Marotandrano, Marotandrano 48.3 km S Mandritsara, -16.28322, 48.81443, 865 m, transition humid forest, 6–8.xii.2007, (*B.L. Fisher et al*.). Collection code BLF19156, (CASC: CASENT0140594). **Paratypes**: (14 major workers and 8 workers), with same data as holotype, 14 major workers (BMNH: CASENT0140593, CASC: CASENT0140693, CASENT0140579, CASENT0140576, CASENT0140596, CASENT0140577, CASENT0140587, CASENT0140589, CASENT0140583, CASENT0140580, CASENT0140582, MCZ: CASENT0140591, MHNG: CASENT0140585, NHMB: CASENT0140597), and 8 workers (BMNH: CASENT0140575, CASC: CASENT0140578, CASENT0140584, CASENT0140590, CASENT0140586, CASENT0140588, MCZ: CASENT0140592, MHNG: CASENT0140581).

#### Diagnosis.

Antennae ten-segmented. **Major**: Head nearly subrectangular, longer than wide, lateral margins nearly straight, posterior margin of head deeply concave; dorsum of propodeum nearly flat, slightly convex in the anterodorsal corner, posterodorsal corner with a pair of small triangular teeth; gaster with short decumbent hairs, and longer subdecumbent hairs. **Minor**: Head longer than wide, lateral margins slightly convex, posterior margin of head nearly straight; dorsum of propodeum flat and declining posteriorly, posterodorsal corner with a pair of small triangular teeth, gaster with short decumbent hairs and dispersed, longer subdecumbent hairs.

**Figure 59.**
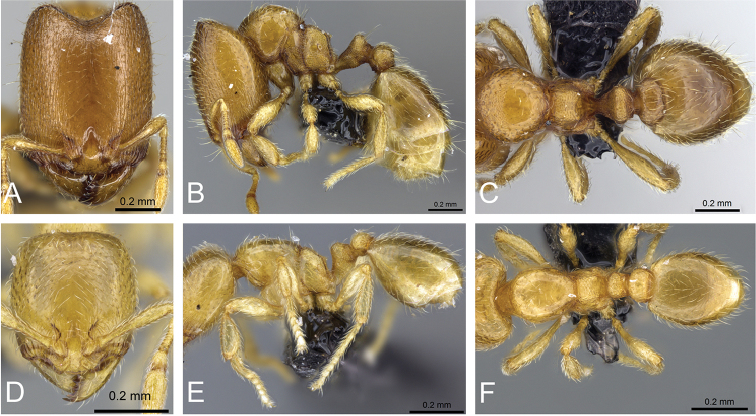
*Carebara raberi*–holotype. Major worker, CASENT0140594: **A** head in full-face view **B** body in profile view **C** body in dorsal view. Minor worker, CASENT0140575: **D** head in full-face view **E** body in profile view **F** body in dorsal view.

**Figure 60.**
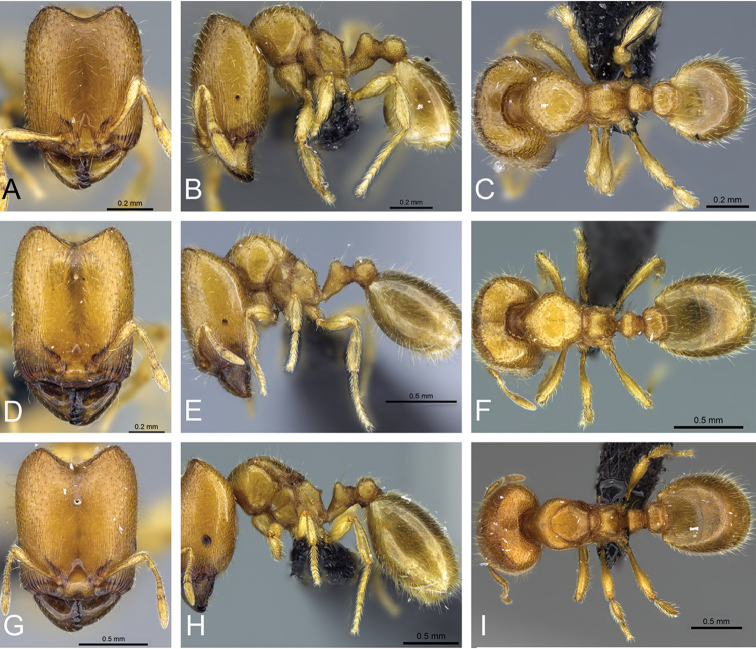
Intermediates of *Carebara raberi*. Major workers, CASENT0140591: **A** head in full-face view **B** body in profile view **C** body in dorsal view. CASENT0056920: **D** head in full-face view **E** body in profile view **F** body in dorsal view. CASENT0140693: **G** head in full-face view **H** body in profile view **I** body in dorsal view.

#### Description of major workers.

**Measurements** (n=16): HL 0.59–1.12 (0.77); HW 0.46–0.89 (0.57); SL 0.25–0.38 (0.28); ML 0.12–0.27 (0.15); EL 0.01–0.09 (0.01); EM 0.16–0.28 (0.21); HD 0.30–0.67 (0.39); WL 0.49–0.95 (0.60); PSL 0.06–0.12 (0.08); PW 0.28–0.52 (0.36); MFL 0.30–0.55 (0.37); MFW 0.08–0.13 (0.09); MTL 0.22–0.46 (0.29); PTL 0.17–0.39 (0.22); PNL 0.08–0.14 (0.09); PTH 0.15–0.26 (0.18); PTW 0.13–0.23 (0.18); PPL 0.11–0.21 (0.15); PPNL 0.10–0.19 (0.12); PPH 0.11–0.24 (0.16); PPW 0.17–0.29 (0.23); GL 0.45–1.05 (0.64); GW 0.36–0.79 (0.47); CI 74–81 (74); MI 18–25 (19); SI 34–46 (36); MLI 56–65 (65); PPLI 52–74 (68); PPI 117–138 (128); PSI 11–14 (14).

Head longer than wide (CI 74–81), in full-face view nearly subrectangular, about 1.3 times longer than wide. Posterior margin of head deeply concave, posterolateral corners well developed and nearly rounded, lateral margins slightly convex, nearly straight, slightly narrowed forward. Mandibles with six teeth. Anterior margin of clypeus straight, and laterally convex. Antennae with ten segments. Scapes short (HL 0.59–1.12, SL 0.25–0.38, SI 34–46). Ocelli present or absent. Eyes present, consisting of one ommatidium (EL 0.01–0.09). Supraclypeal area triangular and well defined.

In profile, promesonotum high and nearly rounded, metanotal groove deeply impressed. Propodeum lower than promesonotum, and about 1.7 times higher than long, dorsal face of propodeum flat and declining posteriorly, propodeum armed, posterodorsal corners each armed with a triangular tooth, anterodorsal corner convex, declivity weakly concave, with thin lateral laminae. Propodeal lobes triangular. Propodeal spiracle rounded and situated above mid-height of sclerite, and beyond mid-length of sclerite by about half the diameter of the spiracle, distance from propodeal spiracle to posterodorsal corner of propodeum about three times the diameter of the spiracle (PSL 0.06–0.12), and distance to declivity about twice the diameter of the spiracle. In dorsal view, promesonotum about as long as wide, anterior margin and sides rounded; sides of propodeum straight.

Petiole longer than high (PTL 0.17–0.39, PTH 0.15–0.26) and with relatively short peduncle, ventral face slightly convex. Combined outline of dorsal surface of peduncle and anterior face of node slightly concave, nearly straight, posterior face of node vertical and weakly convex, anterodorsal corner convex, posterodorsal corner rounded, dorsum convex. Subpetiolar process produced as a small denticle, almost the same as the diameter of the propodeal spiracle. Postpetiolar node nearly rounded and lower than petiolar node. In dorsal view, postpetiolar node wider than petiolar node (PTW 0.13–0.23, PPW 0.17–0.29) and petiolar node wider than long (PNL 0.08–0.14, PTW 0.13–0.23), anterior and posterior margins of petiole straight, anterior margin of postpetiole weakly concave, and weakly convex posterior margin, sides rounded in petiole and postpetiole.

Dorsal surface of mandibles, clypeus and frons smooth and shiny, with scattered piligerous punctae on head and mandibles. Head with longitudinal rugae directed toward posterior margin, rugoreticulate close to posterior margin of head. Posterolateral portion of cephalic dorsum smooth and shiny. Mesosoma smooth and shiny, except katepisternum, propodeum and metapleuron which are areolate. Petiole and ventral face of postpetiole areolate. In dorsal view, promesonotum, postpetiole and gaster smooth and shiny, propodeum and petiole areolate.

Lateral margins of head with short decumbent hairs and long suberect hairs, posterior margin with subdecumbent hairs. Scapes with appressed hairs. Outer margin of mandibles with sparse and short appressed hairs. Mesosoma with short subdecumbent hairs and long suberect hairs. Petiole with short decumbent hairs and long suberect hairs, postpetiole with short and long decumbent hairs. Tibia with appressed hairs. Gaster with short decumbent hairs and long subdecumbent hairs. Color yellowish ferruginous.

#### Description of minor workers.

**Measurements** (n=8): HL 0.38–0.41; HW 0.34–0.37; SL 0.23–0.25; ML 0.09–0.10; EL 0.01–0.02; EM 0.11–0.12; HD 0.24–0.25; WL 0.38–0.43; PSL 0.03–0.04; PW 0.19–0.22; MFL 0.23–0.26; MFW 0.05–0.06; MTL 0.18–0.21; PTL 0.14–0.16; PNL 0.07–0.08; PTH 0.10–0.12; PTW 0.09–0.10; PPL 0.08–0.09; PPNL 0.07–0.09; PPH 0.08–0.09; PPW 0.12–0.13; GL 0.33–0.40; GW 0.25–0.29, CI 87–90; MI 23–26; SI 59–63; MLI 68–74; PPLI 53–64; PPI 130–144; PSI 9–12.

Head longer than wide (CI 87–90), in full-face view nearly subquadrate, about 1.1 times longer than wide, slightly narrowed anteriorly. Posterior margin of head nearly straight, posterolateral corners rounded, lateral margins slightly convex. Mandibles with five teeth. Anterior margin of clypeus nearly straight, and laterally angulate. Antennae with ten segments. Scape fails to reach the posterior margin of head (HL 0.38–0.41, SL 0.23–0.25, SI 59–63). Eyes present, consisting of one ommatidium (EL 0.01–0.02). Supraclypeal area triangular but poorly defined.

In profile view, promesonotum weakly convex, metanotal groove deeply impressed. Propodeum about 1.5 times higher than long, dorsal face of propodeum flat and declining posteriorly, anterodorsal corner rounded, posterodorsal corners of propodeum each armed with a triangular tooth, declivity nearly flat, with thin lateral laminae. Propodeal lobes short and convex. Propodeal spiracle nearly oval and situated above mid-height of sclerite by about half the diameter of the spiracle, and beyond mid-length of sclerite by about the diameter of the spiracle, distance from propodeal spiracle to posterodorsal corner of propodeum about 1.5 times the diameter of the spiracle (PSL 0.03–0.04), and distance to declivity same as the diameter of the spiracle. In dorsal view, promesonotum about 1.3 times longer than wide, anterior margin rounded, sides convex, narrowed posteriorly; sides of propodeum straight.

Petiole longer than high (PTL 0.14–0.16, PTH 0.10–0.12) and with relatively short peduncle, ventral face slightly convex at center. Combined outline of dorsal surface of peduncle and anterior face of node concave, posterior face of node vertical and slightly convex, anterodorsal and posterodorsal corner rounded, dorsum convex. Subpetiolar process produced as a small denticle, slightly smaller than the diameter of the propodeal spiracle. Postpetiolar node nearly rounded and slightly lower than petiolar node. In dorsal view, postpetiolar node wider than petiolar node (PTW 0.09–0.10, PPW 0.12–0.13), and petiolar node wider than long (PNL 0.07–0.08, PTW 0.09–0.10), anterior and posterior margins of petiole weakly convex, anterior margin of postpetiole convex, and posterior margin concave, sides nearly rounded in petiole and postpetiole.

Dorsal surface of head, mandible and clypeus smooth and shiny with scattered piligerous punctae on head and mandibles. Gena and frontal lobes with short longitudinal rugae. Mesosoma smooth and shiny, except for katepisternum, propodeum and metapleuron which are areolate. Petiole and ventral face of postpetiole areolate. In dorsal view, promesonotum, postpetiole and gaster smooth and shiny, propodeum and petiole areolate.

Lateral margins of head with short decumbent hairs, posterior margin with suberect hairs. Scapes and outer margin of mandibles with decumbent hairs. Mesosoma with short and long suberect to subdecumbent hairs. Petiole and postpetiole with short decumbent hairs and longer subdecumbent hairs. Tibia with decumbent hairs. Gaster with short decumbent to appressed hairs, and long subdecumbent hairs. Color yellowish ferruginous.

#### Distribution and biology.

*Carebara raberi* is known from only one locality, in northeastern Madagascar, and only one locality (Figure 69). This species was found in the rainforest, at the transition between humid forest, and tropical dry forest, at 865 m, taken from sifted litter, rotten logs, and leaf mold. Individuals were collected using maxi-Winkler traps.

#### Comments.

*Carebara raberi* can be confused with *C. sampi* and C. *lova* but can be separated by the hair pilosity at the dorsum of the gaster, which is abundant in *C. raberi*, while in *C. sampi* and *C. lova* are fewer. *C. raberi* is known only from one locality in northeastern Madagascar, while *C. sampi* was recorded from south and southwestern Madagascar, and *C. lova* is known from the northwestern of Madagascar. *C. raberi* is the only species of *Carebara* recorded at this area.

*C. raberi* have three intermediates in the major worker subcaste (Figure 60). Posterolateral corners of the head of all intermediates are narrowed posteriorly. The posterior margin of the head is deeply concave in all of the intermediates. The eyes are reduced to one ommatidium in all intermediates. Ocelli are absent in intermediate 1, but present in intermediates 2 and 3 (one ocellus). Reduced flight sclerites are not present in any intermediate. The dorsum of the mesosoma is convex anteriorly and gradually slopes to the declivity in all intermediates. The propodeum is armed with a pair of small triangular teeth in all intermediates. The form of the petiole and postpetiole is the same among all intermediates. The sculpture in intermediate 1 and 3 is finely marked with parallel and longitudinal rugae, but the rugae are better defined in some specimens of intermediate 2. The frontal area is smooth and shiny in all intermediates. The pilosity on the head and body follows the same pattern in all intermediates.

### Carebara salegi

Taxon classificationAnimaliaHymenopteraFormicidae

Azorsa & Fisher sp. n.

http://zoobank.org/26BB897D-52C2-48E6-82D2-D6577B6F15A9

#### Holotype.

(major worker), MADAGASCAR, Antsiranana, Forêt d’Orangea, 3.6 km 128° SE Remena, -12.25889, 49.37467, 90 m, littoral rainforest, 22–28.ii.2001, (*Fisher, Griswold et al.*). Collection code BLF03200, (CASC: CASENT0438150). **Paratypes**: (22 workers), with same data as holotype (BMNH: CASENT0438112, CASC: CASENT0438113, CASENT0438115, CASENT0438132, CASENT0438142, CASENT0438149, CASENT0438120, CASENT0438122, CASENT0438189, CASENT0438121, CASENT0438148, CASENT0438152, CASENT0438119, CASENT0438144, CASENT0438126, CASENT0438110, CASENT0438117, CASENT0438151, CASENT0438186, MCZ: CASENT0438124, MHNG: CASENT0438180, NHMB: CASENT0438113).

#### Diagnosis.

Antennae ten-segmented. **Major**: Head nearly subrectangular, longer than wide, narrowed anteriorly, lateral margins of head convex; dorsum of propodeum nearly flat, posterodorsal corner with a pair of small triangular teeth; combined outline of dorsal surface of peduncle and anterior face of node concave, posterior face vertical and straight; gaster with abundant, short decumbent hairs, and long subdecumbent hairs. **Minor**: Head nearly subquadrate, slightly longer than wide, narrowed anteriorly, posterior margin of head nearly straight; dorsum of propodeum nearly flat, propodeum armed with a pair of short triangular teeth; combined outline of dorsal surface of peduncle and anterior face of node concave, posterior face convex; gaster with abundant, short decumbent hairs, and sparse, long subdecumbent hairs.

**Figure 61.**
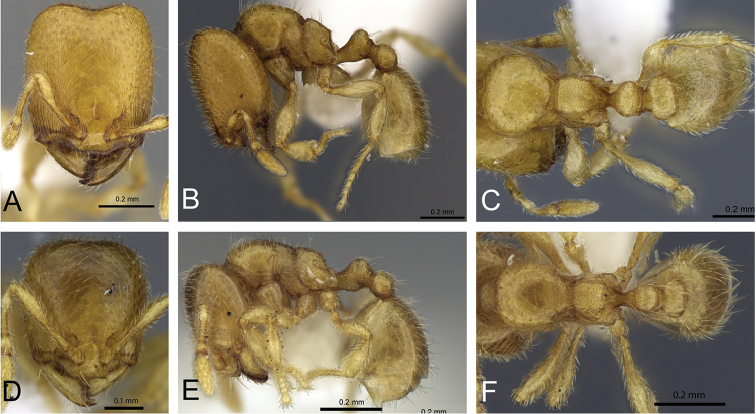
*Carebara salegi*–holotype. Major worker, CASENT0438150: **A** head in full-face view **B** body in profile view **C** body in dorsal view. Minor worker, CASENT0438115: **D** head in full-face view **E** body in profile view **F** body in dorsal view.

#### Description of major workers.

**Measurements** (n=1 (holotype)): HL 0.55; HW 0.46; SL 0.24; ML 0.11; EL 0.02; EM 0.15; HD 0.30; WL 0.47; PSL 0.05; PW 0.30; MFL 0.29; MFW 0.08, MTL 0.22; PTL 0.18; PNL 0.09; PTH 0.14; PTW 0.13; PPL 0.12; PPNL 0.11; PPH 0.11; PPW 0.17; GL0.42; GW 0.36; CI 84; MI 20; SI 44; MLI 63; PPLI 67; PPI 131; PSI 11.

Head longer than wide (CI 84), in full-face view nearly subrectangular, about 1.2 times longer tan wide, slightly narrowed anteriorly. Posterior margin of head medially concave, posterolateral corners rounded, lateral margins convex. Mandibles with six teeth. Anterior margin of clypeus slightly concave in the middle, nearly straight, and laterally convex. Antennae with ten segments. Scapes short (HL 0.55. SL 0.24, SI 44). Ocelli absent. Eyes present, consisiting of three ommatidia (EL 0.02). Supraclypeal area with a longitudinal strip, barely surpassing frontal carina.

In profile view, posterolateral corner of head with a small angulate tooth resembling a horn. Promesonotum high and convex, metanotal groove deeply impressed. Propodeum lower than promesonotum, and about 1.5 times higher than long, dorsal face of propodeum flat and declining posteriorly, posterodorsal corners of propodeum each armed with a triangular tooth, anterodorsal corner convex, declivity of propodeum concave with thin lateral laminae. Propodeal lobes triangular. Propodeal spiracle rounded and situated slightly above mid-height of sclerite by about less tan half the diameter of the spiracle, and beyond mid-length of sclerite by about the diameter of the spiracle, distance from propodeal spiracle to posterodrosal corner of propodeum about 1.8 times the diameter of the spiracle (PSL 0.05), and distance to declivity about 0.5 times the diameter of the spiracle. In dorsal view, promesonotum about as long as wide, anterior margin and sides rounded; sides of propodeum weakly convex.

Petiole longer than high (PTL 0.18, PTH 0.14) and with relatively long peduncle, ventral face weakly convex in the middle. Combined outline of dorsal surface of peduncle and anterior face of node concave, posterior face of node vertical and straight, anterodorsal corner convex, posterodorsal corner rounded, dorsum nearly rounded. Subpetiolar process produced as a small denticle, slightly smaller than the diameter of the propodeal spiracle. Postpetiolar node convex and lower than petiolar node. In dorsal view, postpetiolar node wider than petiolar node (PTW 0.13, PPW 0.17) and petiolar node wider than long (PNL 0.09, PTW 0.13), anterior margin of petiole convex, posterior margin weakly concave, anterior and posterior margins of postpetiole straight, sides rounded in petiole and postpetiole.

Dorsal surface of mandibles, clypeus and frons smooth and shiny with scattered piligerous punctae on head and mandibles. Head with finely longitudinal rugae except for middle area of head, rugoreticulate close to posterior margin of head. Posterolateral portion of cephalic dorsum smooth and shiny. Mesosoma smooth and shiny, except for katepisternum and propodeum (areolate), and metapleuron (areolate and with longitudinal rugae). Petiole and ventral face of postpetiole areolate. In dorsal view, promesonotum, postpetiole and gaster smooth and shiny; propodeum and petiole areolate.

Lateral margins of head with short decumbent hairs and long subdecumbent hairs. Scapes with appressed hairs. Outer margin of mandibles with sparse and short appressed hairs. Mesosoma with short and long suberect hairs. Petiole and postpetiole with short decumbent hairs and long subdecumbent hairs. Tibia with appressed hairs. Gaster with abundant, short decumbent hairs, and sparse, long subdecumbent hairs. Color yellowish ferruginous.

#### Description of minor workers.

**Measurements** (n=8): HL 0.34–0.38; HW 0.30–0.33; SL 0.21–0.24; ML 0.08–0.09; EL 0.01; EM 0.09–0.11; HD 0.20–0.22; WL 0.35–0.38; PSL 0.02–0.04; PW 0.19–0.22; MFL 0.21–0.25; MFW 0.05–0.06; MTL 0.16–0.19; PTL 0.13–0.14; PNL 0.07–0.08; PTH 0.10–0.11; PTW 0.07–0.09; PPL 0.08–0.09; PPNL 0.07–0.08; PPH 0.07–0.08; PPW 0.11–0.12; GL 0.29–0.40; GW 0.22–0.28; CI 85–92; MI 21–26; SI 61–67; MLI 68–78; PPLI 57–69; PPI 129–171; PSI 5–13.

Head longer than wide (CI 85–92), in full-face view nearly subquadrate, about 1.1 times longer than wide, narrowed anteriorly. Posterior margin of head nearly straight, weakly concave, posterolateral corners rounded, lateral margins convex. Mandibles with five teeth. Anterior margin of clypeus nearly straight, and laterally angulate, almost triangular. Antennae with ten segments. Scape fails to reach the posterior margin of head (HL 0.34–0.38, SL 0.21–0.24, SI 61–67). Eyes present, consisting of one ommatidium (EL 0.01). Supraclypeal area triangular but not well defined.

In profile view, promesonotum weakly convex, metanotal groove deeply impressed. Propodeum about 1.2 times higher than long, dorsal face of propodeum nearly flat, and barely declining posteriorly, posterodorsal corner usually unarmed but when armed, posterodorsal corners each armed with a small triangular tooth, declivity nearly flat with thin lateral laminae. Propodeal lobes triangular. Propodeal spiracle nearly oval and situated above mid-height of sclerite, and beyond mid-length of sclerite by about half the diameter of the spiracle, distance from propodeal spiracle to posterodorsal corner of propodeum about twice the diameter of the spiracle (PSL 0.02–0.04), and distance to declivity same as the diameter of the spiracle. In dorsal view, promesonotum about 1.1 times longer than wide, anterior margin and sides rounded, narrowed posteriorly; sides of propodeum flat.

Petiole longer than high (PTL 0.13–0.14, PTH 0.10–0.11) and with relatively short peduncle, ventral face convex. Combined outline of dorsal surface of peduncle and anterior face of node concave, posterior face of node convex, anterodorsal and posterodorsal corner rounded, dorsum nearly rounded. Subpetiolar process produced as a small denticle. Postpetiolar node convex and lower than petiolar node. In dorsal view, postpetiolar node wider than petiolar node (PTW 0.07–0.09, PPW 0.11–0.12), and petiolar node as wide as long (PNL 0.07–0.08, PTW 0.07–0.09), anterior and posterior margins of petiole convex, and weakly concave in postpetiole, sides convex in petiole and rounded in postpetiole.

Dorsal surface of head, mandibles and clypeus smooth and shiny with scattered piligerous punctae on head and mandibles. Gena and frontal lobes with short longitudinal rugae. Mesosoma smooth and shiny, except for katepisternum and propodeum (areolate), and metapleuron (longitudinally and finely areolate-rugose). Petiole and ventral face of postpetiole areolate. In dorsal view, promesonotum, petiole, postpetiole and gaster smooth and shiny; propodeum areolate.

Lateral margins and posterior margin of head with subdecumbent hairs. Scapes with abundant decumbent to subdecumbent hairs. Outer margin of mandibles with few decumbent hairs. Mesosoma with short and long suberect hairs. Petiole and postpetiole with short decumbent hairs and long subdecumbent hairs. Tibia with decumbent hairs. Gaster with short decumbent to appressed hairs and long suberect to subdecumbent hairs. Color yellowish ferruginous.

#### Distribution and biology.

*Carebara salegi* is known only from the north of Madagascar (Figure 69) and occurs in the littoral rainforest, at an elevation of 90 m. Specimens were collected using maxi-Winkler traps and found in leaf litter, leaf mold and rotten wood.

#### Comments.

*Carebara salegi* is endemic to the north of Madagascar, and only one major worker of this species has been collected. Other six species were recorded at the same area: *C. bara*, *C. berivelo*, *C. grandidieri*, *C. kabosy*, *C. malagasy*, and *C. tana*.

*C. salegi* may be confused with *C. mahafaly* because of the form of the head. However, the posterior margin of the head of *C. mahafaly* is more concave than *C. salegi*, and the lateral margins of the head of *C. mahafaly* are more convex. The promesonotum is nearly rounded in *C. mahafaly* and convex in *C. salegi*. The posterodorsal corners of propodeum in *C. mahafaly* have a pair of triangular teeth directed slightly upward. In *C. salegi* the propodeum is armed with a pair of small triangular teeth that are not directed upward. The anterodorsal corner of the petiole in *C. mahafaly* is nearly rounded while *C. salegi* is convex. The gaster has short appressed hairs in *C. mahafaly*, and short decumbent hairs in *C. salegi*. In the workers, the main difference between these two species is the form of the propodeum, which is similar to the major worker subcaste.

*C. salegi* does not have intermediates in the major worker subcaste.

### Carebara sampi

Taxon classificationAnimaliaHymenopteraFormicidae

Azorsa & Fisher sp. n.

http://zoobank.org/779798F1-FB45-4E6C-A995-15A6EBC91F7C

#### Holotype.

(major worker), MADAGASCAR, Fianarantsoa, Parc National d’Isalo, Sahanafa River, 29.2 km 351° N Ranohira, -22.31333, 45.29167, 500 m, gallery forest, 10–13.ii.2003, (*Fisher, Griswold et al.*). Collection code BLF07651, (CASC: CASENT0031265). **Paratypes**: (9 major worker and 29 workers), with same data as holotype, 8 major workers (BMNH: CASENT0031260, CASC: CASENT0031268, CASENT0030374, CASENT0031267, CASENT0030481, CASENT0030407, CASENT0030441, MCZ: CASENT0030377), and 29 workers (BMNH: CASENT0030403, CASC: CASENT0030456, CASENT0031234, CASENT0031262, CASENT0031216, CASENT0030486, CASENT0031215, CASENT0031274, CASENT0030415, CASENT0030444, CASENT0030490, CASENT0030379, CASENT0030413, CASENT0030398, CASENT0030402, CASENT0030406, CASENT0030437, CASENT0030414, CASENT0030518, CASENT0030447, CASENT0030401, CASENT0030420, CASENT0031259, CASENT0030400, CASENT0031261, CASENT0030443, MCZ: CASENT0030409, MHNG: CASENT0030425, NHMB: CASENT0031272). 1 major worker with same data as holotype and collection code BLF07653, (CASC: CASENT0049730).

#### Diagnosis.

Antennae ten-segmented. **Major**: Head subrectangular, longer than wide, narrowed posteriorly, lateral margins slightly convex, posterolateral corners well developed, posterior margin of head deeply concave; dorsum of propodeum flat, posterodorsal corner with a pair of small triangular teeth; gaster with short appressed hairs, and longer, sparse suberect or subdecumbent hairs. **Minor**: Head subquadrate, slightly longer than wide, narrowed anteriorly, posterior margin of head nearly straight, lateral margins convex; dorsum of propodeum nearly flat, anterodorsal corner slightly convex, posterodorsal corner with a pair of triangular teeth; combined outline of dorsal surface of peduncle and anterior face of node nearly concave, and posterior face convex; gaster with short appressed hairs and dispersed and longer suberect or subdecumbent hairs.

**Figure 62.**
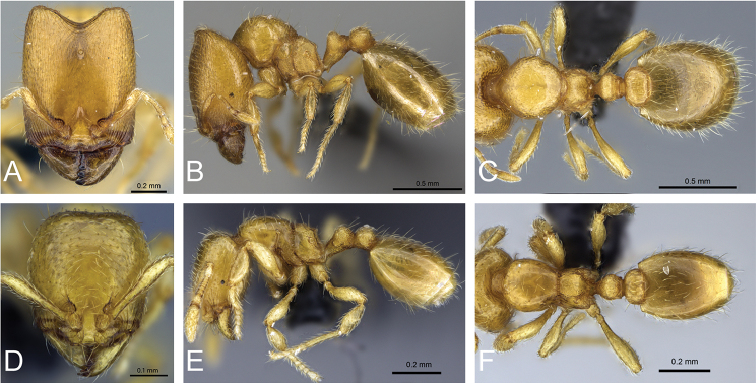
*Carebara sampi*–holotype. Major worker, CASENT0031265: **A** head in full-face view **B** body in profile view **C** body in dorsal view. Minor worker, CASENT0031214: **D** head in full-face view **E** body in profile view **F** body in dorsal view.

**Figure 63.**
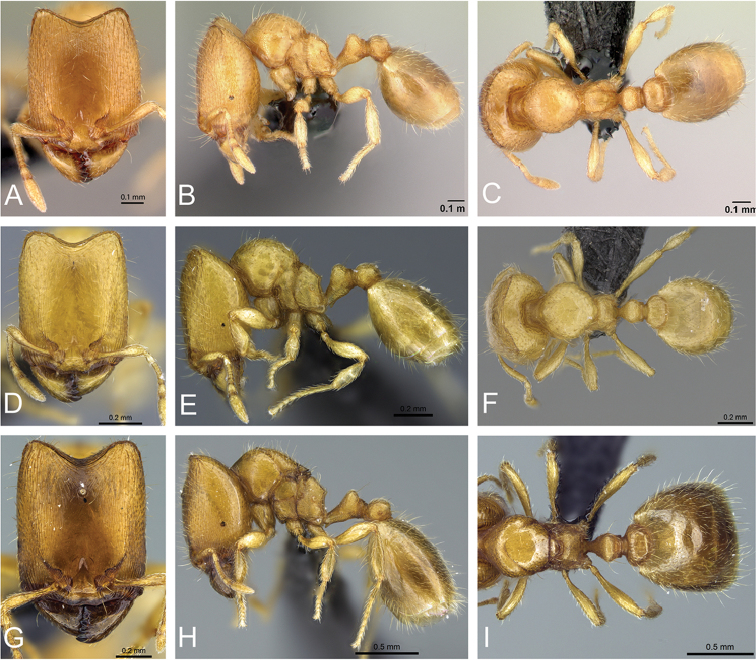
Intermediates of *Carebara sampi*. Major workers, CASENT0018045: **A** head in full-face view **B** body in profile view **C** body in dorsal view. CASENT0030374: **D** head in full-face view **E** body in profile view **F** body in dorsal view. CASENT0031267 **G** head in full-face view **H** body in profile view **I** body in dorsal view.

#### Description of major workers.

**Measurements** (n=13): HL 0.57–0.92 (0.82); HW 0.49–0.72 (0.64); SL 0.26–0.35 (0.31); ML 0.12–0.24 (0.21); EL 0.01–0.03 (0.02); EM 0.16–0.28 (0.25); HD 0.32–0.52 (0.45); WL 0.51–0.85 (0.70); PSL 0.05–0.11 (0.08); PW 0.30–0.47 (0.41); MFL 0.29–0.52 (0.41); MFW 0.07–0.12 (0.10); MTL 0.21–0.40 (0.30); PTL 0.19–0.32 (0.27); PNL 0.09–0.14 (0.12); PTH 0.14–0.24 (0.20); PTW 0.12–0.22 (0.17); PPL 0.11–0.22 (0.18); PPNL 0.11–0.20 (0.16); PPH 0.12–0.23 (0.18); PPW 0.18–0.32 (0.25); GL 0.43–1.10 (0.78); GW 0.39–0.82 (0.58); CI 76–86 (78); MI 21–26 (26); SI 37–46 (38); MLI 59–74 (64); PPLI 58–70 (67); PPI 131–152 (147); PSI 10–16 (13).

Head longer than wide (CI 76–86), in full-face view subrectangular, about 1.3 times longer than wide, slightly narrowed on the top. Posterior margin of head deeply concave in the middle, posterolateral corners well developed, rounded and narrowed forward, lateral margins slightly convex. Mandibles with six teeth. Anterior margin of clypeus slightly concave, and laterally convex. Antennae with ten segments. Scapes short (HL 0.57–0.92, SL 0.26–0.35, SI 37–46). Ocelli present or absent. Eyes present, consisting of two or three ommatidia (EL 0.01–0.03). Supraclypeal area triangular and well defined.

In profile view, posterolateral corner of head with a small angulate tooth resembling a horn. Promesonotum high and nearly rounded, metanotal groove deeply impressed. Propodeum lower than promesonotum, and about 1.4 times higher than long, dorsal face of propodeum flat and declining posteriorly, posterodorsal corners of propodeum each armed with a small triangular tooth, declivity concave, nearly flat with thin lateral laminae. Propodeal lobes convex. Propodeal spiracle nearly oval and situated above mid-height of sclerite, and beyond mid-length of sclerite by about half the diameter of the spiracle, (close to mid-height and mid-length in larger major workers), distance from propodeal spiracle to posterodorsal corner of propodeum about twice the diameter of the spiracle (PSL 0.05–0.11), and distance to declivity same as the diameter of the spiracle. In dorsal view, promesonotum about as long as wide, anterior and posterior margins, as well as sides rounded; sides of propodeum straight.

Petiole longer than high (PTL 0.19–0.32, PTH 0.14–0.24) and with relatively long peduncle, ventral face weakly convex in the middle. Combined outline of dorsal surface of peduncle and anterior face of node slightly concave, posterior face of node vertical and weakly concave, anterodorsal corner convex, posterodorsal corner rounded, dorsum nearly rounded. Subpetiolar process produced as a small denticle, almost as large as the diameter of the propodeal spiracle. Postpetiolar node rounded and slightly lower than petiolar node. In dorsal view, postpetiolar node wider than petiolar node (PTW 0.12–0.22, PPW 0.18–0.32) and petiolar node wider than long (PNL 0.09–0.14, PTW 0.12–0.22), anterior and posterior margins of petiole and postpetiole nearly straight, sides of petiole and postpetiole nearly rounded.

Dorsal surface of mandibles, clypeus and frons smooth and shiny with scattered piligerous punctae on head and mandibles. Head with longitudinal rugae directed to posterolateral corners of head, transverse rugae, and reticulate on median area of posterior margin of head, followed by short longitudinal rugae. Mesosoma smooth and shiny, except for katepisternum and propodeum (areolate), and metapleuron (areolate-rugose and with longitudinal rugae). Petiole, ventral face and sides of postpetiole areolate. In dorsal view, promesonotum, postpetiole and gaster smooth and shiny; propodeum and petiole areolate.

Lateral margins of head with short appressed hairs and long suberect hairs. Scapes with appressed hairs. Outer margin of mandibles with short and sparse appressed hairs. Mesosoma with short subdecumbent hairs and long suberect hairs. Petiole and postpetiole with short decumbent or appressed hairs and long subdecumbent hairs. Tibia with appressed hairs. Gaster with short appressed hairs and long suberect or subdecumbent hairs. Color yellowish ferruginous.

#### Description of minor workers.

**Measurements** (n=11): HL 0.32–0.40; HW 0.27–0.34; SL 0.20–0.26; ML 0.08–0.10; EL 0.01–0.02; EM 0.09–0.11; HD 0.20–0.22; WL 0.32–0.42; PSL 0.03–0.05; PW 0.10–0.21; MFL 0.20–0.26; MFW 0.05–0.07; MTL 0.14–0.20; PTL 0.11–0.15; PNL 0.06–0.08; PTH 0.09–0.12; PTW 0.08–0.10; PPL 0.06–0.09; PPNL 0.06–0.09; PPH 0.07–0.09; PPW 0.10–0.14; GL 0.26–0.48; GW 0.20–0.35; CI 83–91; MI 22–29; SI 61–68; MLI 69–81; PPLI 57–69; PPI 125–150; PSI 9–13.

Head longer than wide (CI 83–91), in full-face view nearly subquadrate, about 1.1 times longer than wide, narrowed anteriorly. Posterior margin of head nearly straight, weakly concave, posterolateral corners rounded, lateral margins convex. Mandibles with five teeth. Anterior margin of clypeus concave, and laterally angulate, almost triangular. Antennae with ten segments. Scape fails to reach posterior margin of head (HL 0.32–0.40, SL 0.20–0.26, SI 61–81). Eyes present, consisting of two ommatidia (EL 0.01–0.02). Supraclypeal area short and triangular, but poorly defined.

In profile, promesonotum weakly convex, nearly flat, metanotal groove deeply impressed. Propodeum 1.3 times higher than long, dorsal face of propodeum flat and barely declining posteriorly, posterodorsal corners of propodeum each armed with a triangular tooth, anterodorsal corner rounded, declivity concave with thin lateral laminae. Propodeal lobes convex. Propodeal spiracle rounded and situated above mid-height of sclerite by about half the diameter of the spiracle, and beyond mid-length of sclerite by about two times the diameter of the spiracle, distance from propodeal spiracle to posterodorsal corner of propodeum about twice the diameter of the spiracle (PSL 0.03–0.05), and distance to declivity about 0.5 times the diameter of the spiracle. In dorsal view, promesonotum almost as longer as wide, anterior margin rounded, sides convex and narrowed posteriorly; sides of propodeum convex.

Petiole longer than high (PTL 0.11–0.15, PTH 0.09–0.12) and with relatively long peduncle, ventral face medially convex. Combined outline of dorsal surface of peduncle and anterior face of node slightly concave in the middle, nearly straight, posterior face of node convex, anterodorsal and posterodorsal corner rounded, dorsum nearly rounded. Subpetiolar process produced as a small denticle, almost as large as the diameter of the propodeal spiracle. Postpetiolar node convex and slightly lower than petiolar node. In dorsal view, postpetiolar node wider than petiolar node (PTW 0.08–0.10, PPW 0.10–0.14), and petiolar node slightly wider than long (PNL 0.06–0.08, PTW 0.08–0.10), anterior and posterior margins of petiole weakly convex, and straight in postpetiole, sides rounded in petiole and postpetiole.

Dorsal surface of head, mandibles, and clypeus smooth and shiny with scattered piligerous punctae on head and mandibles. Gena and frontal lobes with short longitudinal rugae. Mesosoma smooth and shiny, except for katepisternum and propodeum (areolate), metapleuron (finely areolate). Petiole and ventral face of postpetiole areolate-rugose. In dorsal view, promesonotum, postpetiole and gaster smooth and shiny; propodeum and petiole areolate.

Lateral margins of head and scape with decumbent hairs. Outer margin of mandibles with appressed hairs. Mesosoma with short subdecumbent and decumbent hairs and long suberect hairs. Petiole and postpetiole with short decumbent hairs and long subdecumbent hairs. Tibia with appressed hairs. Gaster with short decumbent or appressed hairs and long suberect or subdecumbent hairs. Color yellowish ferruginous.

#### Distribution and biology.

*Carebara sampi* is known from south and southwestern Madagascar (Figure 69) where it was found in gallery forest, tropical dry forest, and gallery forest on sandy soil, at elevations ranging from 20 m to 525 m. This species was found in the following microhabitats: rotten logs, leaf mold, rotten wood, and in semi-deciduous vegetation. Specimens were collected using maxi-Winkler traps.

#### Comments.

*Carebara sampi* can be confused with *C. lova* but can be separated by the distance from the posterior border of propodeal spiracle to declivity, which is less than two times the diameter of the spiracle in *C. sampi*, while is same as or less than the diamater of the spiracle in *C. lova. C. sampi* is distributed from south and southwestern Madagascar, while *C. lova* is located in the northwestern of Madagascar. Other five species were recorded at the same area: *C. bara*, *C. dota*, *C. grandidieri*, *C. hiragasy*, and *C. omasi*.

*C. sampi* have three intermediates in the major worker subcaste (Figure 63). The posterolateral corner of head in intermediate 1 is narrowed posteriorly, and the posterior margin of the head is medially concave in all intermediates. Eyes are small and reduced to one ommatidium in all intermediates. Ocelli are absent in intermediate 1, slightly present in intermediate 2, present and well developed in intermediate 3 (one ocellus). Reduced flight sclerites are present in intermediate 3 and absent in intermediate 1 and 2. The dorsum of the mesosoma is convex anteriorly and gradually slopes to the declivity in intermediate 3, while in intermediate 1 and 2 the dorsum of the mesosoma is nearly rounded anteriorly, the propodeum is below the promesonotum. The propodeum is armed with a pair of small triangular teeth in all intermediates. The form of the petiole and postpetiole is the same among the intermediates. The sculpture is the same in all intermediates, with longitudinal rugae on the head with the frontal area smooth and shiny. The pilosity on the head and body follow the same pattern, except for intermediate 3, which has more abundant appressed hairs on the gaster than intermediates 1 and 2.

#### Additional material examined.

**MADAGASCAR**: ***Toliara***. Réserve Privé Berenty, Forêt de Malaza, Mandraré River, 8.6 km 314° NW Amboasary, -25.00778, 46.306, 40 m, gallery forest, 6.ii.2002, (*Fisher, Griswold et al.*); Toliara, Forêt de Mite, 20.7 km 29° WNW Tongobory, -23.52417, 44.12133, 75 m, gallery forest, 27.ii.-3.iii.2002, (*Fisher, Griswold et al.*); Toliara, Réserve Privé Berenty, Forêt de Bealoka, Mandraré River, 14.6 km 329° NNW Amboasary, -24.95694, 46.2715, 35 m, gallery forest, 3–8.ii.2002, (*Fisher, Griswold et al.*); Toliara, Fiherenana, -23.17698, 43.96142, gallery forest, 18–19.viii.2003, (*Frontier Wilderness Project*); Toliara, Beza-Mahafaly, 27 km E Betioky, -23.65, 44.63333, 135 m, tropical dry forest, 23.iv.1997, (*B.L. Fisher*); Toliara, Makay Mts., -21.21836, 45.3106, 510 m, gallery forest on sandy soil, 24–27.xi.2010, (*B.L. Fisher et al*.); Toliara, Makay Mts., -21.20978, 45.34184, 525 m, gallery forest on sandy soil, 27.xi-2.xii.2010, *(B.L. Fisher et al*.); Toliara, Makay, -21.22283, 45.32453, 525 m, gallery forest, 11.i.2010, (*J.M. Bichain*); Toliara, Makay, -21.22283, 45.32453, 525 m, gallery forest, 9.i.2010, (*J.M. Bichain*).

### Carebara tana

Taxon classificationAnimaliaHymenopteraFormicidae

Azorsa & Fisher sp. n.

http://zoobank.org/7FEAAB21-C3CA-4330-BFE0-C9CE8DF256DD

#### Holotype.

(major worker), MADAGASCAR, Antsiranana, Parc National Montagne d’Ambre, 3.6 km 235° SW Joffreville, -12.53444, 49.1795, 925 m, montane rainforest, 20–26.i.2001, (*Fisher, Griswold et al.*). Collection code BLF02564, (CASC: CASENT0437707). **Paratypes**: (3 major worker and 9 workers), with same data as holotype, 3 major workers (CASC: CASENT0438090, CASENT0437819, CASENT0437704), and 9 workers (BMNH: CASENT0437708, CASC: CASENT0438034, CASENT0438095, CASENT0438096, CASENT0438088, CASENT0438109, MCZ: CASENT0437825, MHNG: CASENT0437830, NHMB: CASENT0438076).

#### Diagnosis.

Antennae ten-segmented. **Major**: Head subrectangular, longer than wide, lateral margins straight and parallel, posterolateral corners rounded, posterior margin of head concave; dorsum of propodeum flat and declining posteriorly, posterodorsal corner with a pair of triangular teeth; gaster with short decumbent hairs and sparse and longer subdecumbent hairs. **Minor**: Head nearly subquadrate, slightly longer than wide, lateral margins weakly convex, posterior margin weakly convex; dorsum of propodeum nearly flat, posterodorsal corner with a pair of small triangular teeth; gaster with abundant decumbent hairs and sparse suberect to subdecumbent hairs.

**Figure 64.**
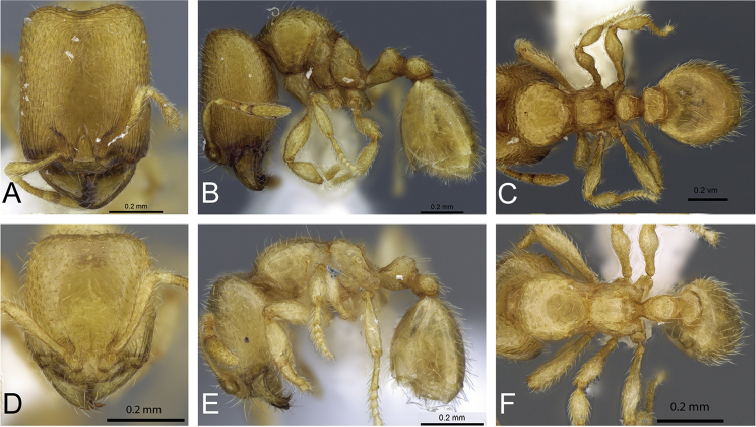
*Carebara tana*–holotype. Major worker, CASENT0437707: **A** head in full-face view **B** body in profile view **C** body in dorsal view. Minor worker, CASENT0438084: **D** head in full-face view **E** body in profile view **F** body in dorsal view.

#### Description of major workers.

**Measurements** (n=5): HL 0.61–0.65 (0.65); HW 0.47–0.51 (0.49); SL 0.23–0.26 (0.25); ML 0.12–0.14 (0.13); EL 0.02–0.03 (0.02); EM 0.14–0.20 (0.18); HD 0.29–0.33 (0.33); WL 0.50–0.54 (0.53); PSL 0.05–0.07 (0.06); PW 0.31–0.35 (0.32); MFL 0.29–0.32 (0.32); MFW 0.08–0.09 (0.08); MTL 0.20–0.27 (0.20), PTL 0.20–0.24 (0.20); PNL 0.11–0.12 (0.11); PTH 0.14–0.16 (0.16); PTW 0.13–0.15 (0.14); PPL 0.12–0.13 (0.12); PPNL 0.11–0.12 (0.12); PPH 0.11–0.12 (0.12); PPW 0.16–0.18 (0.18); GL 0.43–0.51 (0.51); GW 0.36–0.41 (0.41); CI 75–79 (75); MI 19–22 (20); SI 38–40 (38); MLI 60–65 (65); PPLI 50–65 (60); PPI 120–129 (129); PSI 10–15 (12).

Head longer than wide (CI 75–79), in full-face view nearly subrectangular, about 1.3 times longer than wide. Posterior margin of head medially concave, posterolateral corners rounded, lateral margins straight and parallel. Mandibles with five teeth. Anterior margin of clypeus slightly concave, and laterally convex. Antennae with ten segments. Scapes short (HL 0.61–0.65, SL 0.23–0.26, SI 38–40) not surpassing cephalic midlength. Ocelli absent. Eyes present, consisting of two or three ommatidia (EL 0.02–0.03). Supraclypeal area short and triangular.

In profile view, promesonotum high and nearly rounded, metanotal groove deeply impressed. Propodeum lower than promesonotum, and about 1.7 times higher than long, dorsal face of propodeum flat and declining posteriorly, posterodorsal corners of propodeum each armed with a triangular tooth, declivity concave, nearly flat, with thin lateral laminae. Propodeal lobes triangular. Propodeal spiracle rounded and situated slightly above mid-height of sclerite by about half the diameter of the spiracle, and beyond mid-length of sclerite by about the diameter of the spiracle, distance from propodeal spiracle to posterodorsal corner of propodeum about 2.5 times the diameter of the spiracle (PSL 0.05–0.07), and distance to declivity same as the diameter of the spiracle. In dorsal view, promesonotum about as long as wide, anterior margin and sides rounded, slightly narrowed posteriorly; sides of propodeum straight.

Petiole longer than high (PTL 0.20–0.24, PTH 0.14–0.16) and with relatively long peduncle, ventral face weakly convex at center. Combined outline of dorsal surface of peduncle and anterior face of node concave, posterior face of node weakly convex, anterodorsal corner convex, posterodorsal corner rounded, dorsum convex. Subpetiolar process produced as a small denticle, almost as large as the diameter of the propodeal spiracle. Postpetiolar node convex and lower than petiolar node. In dorsal view, postpetiolar node wider than petiolar node (PTW 0.13–0.15, PPW 0.16–0.18) and petiolar node wider than long (PNL 0.11–0.12, PTW 0.13–0.15), anterior margin of petiole convex, posterior margin weakly concave, anterior and posterior margins of postpetiole straight, sides of petiole rounded, and sides of postpetiole narrowed posteriorly and convex.

Dorsal surface of mandibles, clypeus and frons smooth and shiny with scattered piligerous punctae on head and mandibles. Head with longitudinal and parallel rugae, these joining near posterior margin of head, transverse rugae along posterior margin of head. Posterolateral portion of cephalic dorsum with longitudinal rugae. Mesosoma smooth and shiny, except for katepisternum (areolate), metapleuron (rugose and with longitudinal rugae), and dorsum of propodeum (finely areolate). Petiole and ventral face of postpetiole (areolate). In dorsal view, promesonotum, petiole, postpetiole and gaster smooth and shiny.

Lateral margins of head with short decumbent hairs and long suberect hairs, posterior margin with subdecumbent hairs. Scapes with decumbent hairs. Outer margin of mandibles with short appressed hairs. Mesosoma with short and long subdecumbent hairs. Petiole, and postpetiole with short decumbent hairs and long subdecumbent hairs. Tibia with appressed hairs. Gaster with short decumbent hairs and long subdecumbent or suberect hairs. Color yellowish ferruginous.

#### Description of minor workers.

**Measurements** (n=9): HL 0.35–0.38; HW 0.31–0.33; SL 0.21–0.23; ML 0.09–0.10; EL 0.01; EM 0.10–0.11; HD 0.21–0.23; WL 0.35–0.39; PSL 0.03–0.04; PW 0.19–0.21; MFL 0.22–0.24; MFW 0.05, MTL 0.16–0.18; PTL 0.12–0.15; PNL 0.06–0.07; PTH 0.10–0.11; PTW 0.07–0.08; PPL 0.07–0.08; PPNL 0.06–0.07; PPH 0.07–0.08; PPW 0.10–0.13; GL 0.31–0.35; GW 0.21–0.28; CI 86–89; MI 24–27; SI 57–63; MLI 69–75; PPLI 50–58; PPI 125–163; PSI 9–13.

Head slightly longer than wide (CI 86–89), in full-face view nearly subquadrate, about 1.1 times longer than wide. Posterior margin of head slightly concave, posterolateral corners rounded, lateral margins weakly convex. Mandibles with five teeth. Anterior margin of clypeus slightly concave in the middle, and laterally angulate. Antennae with ten segments. Scape fails to reach posterior margin of head (HL 0.35–0.38, SL 0.21–0.23, SI 57–63). Eyes present, consisting of one ommatidium (EL 0.01). Supraclypeal area triangular but not well defined.

In profile view, promesonotum weakly convex, metanotal groove deeply impressed. Propodeum about 1.1 times higher than long, dorsal face of propodeum slightly concave in the middle, nearly flat, declining posteriorly, posterodorsal corners each armed with a triangular tooth, anterodorsal corner rounded and higher than posterodorsal corner of promesonotum, declivity concave with thin lateral laminae. Propodeal lobes triangular. Propodeal spiracle rounded and situated above mid-height of sclerite by about the diameter of the spiracle, and beyond mid-length of sclerite by about two times the diameter of the spiracle, distance from propodeal spiracle to posterodorsal corner of propodeum about 2.5 times the diameter of the spiracle (PSL 0.03–0.04), and distance to declivity almost half the diameter of the spiracle. In dorsal view, promesonotum about as long as wide, anterior margin rounded, sides convex, narrowed posteriorly; sides of propodeum straight.

Petiole longer than high (PTL 0.12–0.15, PTH 0.10–0.11) and with a short peduncle, ventral face convex at center. Combined outine of dorsal surface of peduncle and anterior face of node slightly concave, posterior face of node vertical and slightly convex, anterodorsal corner convex, posterodorsal corner rounded, dorsum convex. Subpetiolar process produced as a small denticle. Postpetiolar node nearly rounded and lower than petiolar node. In dorsal view, postpetiolar node wider than petiolar node (PTW 0.07–0.08, PPW 0.10–0.13), and petiolar node almost as broad as long (PNL 0.06–0.07, PTW 0.07–0.08), anterior and posterior margins of petiole convex, anterior and posterior margins of postpetiole straight, sides rounded in petiole and postpetiole.

Head, mandible and clypeus smooth and shiny with scattered piligerous punctae on head and mandibles. Gena and frontal lobes with short longitudinal rugae. Mesosoma smooth and shiny, except for katepisternum (areolate), and metapleuron (finely areolate). Petiole and ventral face of postpetiole areolate. In dorsal view, mesosoma, petiole, postpetiole and gaster smooth and shiny.

Lateral margins and posterior margin of the head and scapes with subdecumbent hairs. Outer margin of mandibles with decumbent hairs. Mesosoma with short and long suberect hairs. Petiole and postpetiole with short decumbent hairs and long subdecumbent hairs. Gaster with abundant subdecumbent and decumbent hairs, and sparse suberect hairs. Tibia with appressed hairs. Color yellowish ferruginous.

#### Distribution and biology.

*Carebara tana* is known only from the north of Madagascar (Figure 69). This species was found in montane rainforest, at an elevation of 925 m, in leaf litter, leaf mold and rotten wood. This species was collected using maxi-Winkler traps.

#### Comments.

*Carebara tana* can be confused with *C. demeter* due to the form of the head. However, the two can be separated by the following combination of characters: in full-face view, the head sculpture of *C. tana* has nearly longitudinal and parallel rugae, while *C. demeter* has irregular longitudinal rugae, and is reticulate near the posterior margin of the head; the frontal area is smooth and shiny in *C. tana*; the petiolar node of *C. tana* is thicker than *C. demeter*; the combined outline of dorsal surface of peduncle and anterior face of node is nearly flat in *C. demeter*, and slightly concave in *C. tana*; the anteroventral corner has a well-developed triangular tooth in *C. demeter*, and a small, angulate, or triangular tooth in *C. tana*; the anterodorsal corner of the petiole is convex in *C. tana*, and rounded in *C. demeter*, the posterodorsal corner is convex in *C. demeter*, and slightly concave in *C. tana. C. tana* is endemic to the north of Madagascar and other six species were recorded at the same area: *C. bara*, *C. berivelo*, *C. grandidieri*, *C. kabosy*, *C. malagasy*, and *C. vazimba*.

*C. tana* does not have intermediates in the major worker subcaste.

### Carebara tanana

Taxon classificationAnimaliaHymenopteraFormicidae

Azorsa & Fisher sp. n.

http://zoobank.org/180C3905-077F-47F7-A2B3-AFA620DB28A9

#### Holotype.

(major worker), MADAGASCAR, Antananarivo, Antaponimanadala I Non Protected Area, 6.59 km E Manalalondo, -19.25528, 47.1771, 1984 m, savannah grassland, 13–16.v.2010, (*Andrianjaka Ravelomanana*). Collection code ARA1053, (CASC: CASENT0211683). **Paratypes**: (3 major worker and 6 workers), with same data as holotype, 3 major workers (CASC: CASENT0211708, CASENT0211736, CASENT0211696), and 6 workers (CASC: CASENT0211714, CASENT0211777, CASENT0211709, CASENT0211697, CASENT0211737, CASENT0211684).

#### Diagnosis.

Antennae ten-segmented. **Major**: Head subrectangular, longer than wide, lateral margins straight and parallel, posterolateral corners rounded, posterior margin of head concave; combined outline of dorsal surface of peduncle and anterior face of node nearly straight; dorsum of propodeum flat and declining posteriorly, posterodorsal corner unarmed, nearly angulate; gaster with abundant subdecumbent to suberect hairs. **Minor**: Head nearly subquadrate, slightly longer than wide, lateral margins weakly convex, posterior margin weakly convex; combined outline of dorsal surface of peduncle and anterior face of node nearly straight; dorsum of propodeum nearly flat, declining posteriorly, posterodorsal corner unarmed, nearly angulate; gaster with subdecumbent or suberect hairs.

**Figure 65.**
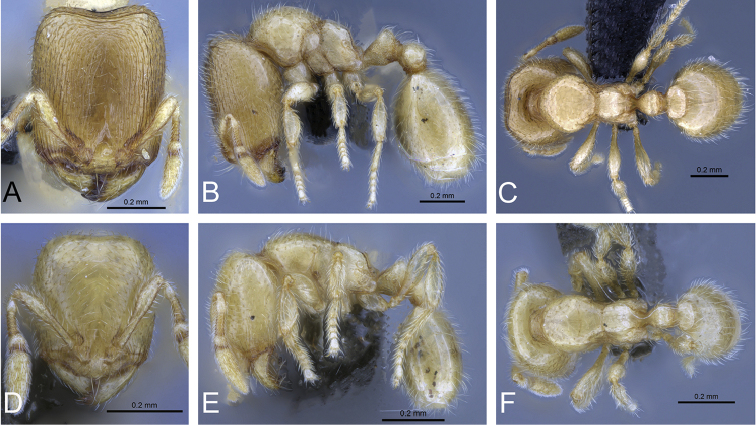
*Carebara tanana*–holotype. Major worker, CASENT0211683: **A** head in full-face view **B** body in profile view **C** body in dorsal view. Minor worker, CASENT0211714: **D** head in full-face view **E** body in profile view **F** body in dorsal view.

#### Description of major workers.

**Measurements** (n=1 (holotype)): HL 0.62; HW 0.48; SL 0.28; ML 0.15; EL 0.02; EM 0.18; HD 0.35; WL 0.54; PSL 0.06; PW 0.33; MFL 0.29; MFW 0.08; MTL 0.22, PTL 0.21; PNL 0.09; PTH 0.17; PTW 0.15; PPL 0.13; PPNL 0.13; PPH 0.13; PPW 0.20; GL 0.53; GW 0.43; CI 78; MI 24; SI 45; MLI 60; PPLI 60; PPI 133; PSI 12.

Head longer than wide (78), in full-face view weakly subrectangular, about 1.2 times longer than wide. Posterior margin of head medially concave, posterolateral corners rounded, lateral margins straight or weakly convex. Mandibles with five teeth. Anterior margin of clypeus nearly straight, and weakly convex laterally. Antennae with ten segments. Scapes short (HL 0.62, SL 0.28, SI 45) barely reaching cephalic midlength. Ocelli absent. Eyes present and short, consisting of only ommatidia (EL 0.02). Supraclypeal area short and triangular, not surpassing frontal carina.

In profile view, promesonotum high and convex, metanotal groove present and not deeply impressed. Propodeum lower than promesonotum, and about 1.3 times higher than long, dorsal face of propodeum flat and declining posteriorly, posterodorsal corners of propodeum unarmed, nearly angulate, declivity weakly convex, nearly flat. Propodeal lobes short and convex. Propodeal spiracle rounded and situated slightly above mid-height of sclerite, and beyond mid-length of sclerite by about half the diameter of the spiracle, distance from propodeal spiracle to posterodorsal corner of propodeum about same as the diameter of the spiracle (PSL 0.06), and distance to declivity less than the diameter of the spiracle. In dorsal view, promesonotum about as long as wide, anterior margin and sides rounded, slightly narrowed posteriorly; sides of propodeum nearly convex.

Petiole weakly longer than high (PTL 0.21, PTH 0.17) and with relatively short peduncle, ventral face weakly convex at center. Combined outline of dorsal surface of peduncle and anterior face of node straigth, posterior face of node weakly concave, anterodorsal corner convex, posterodorsal corner rounded, dorsum convex. Subpetiolar process produced as a small denticle, shorter than the diameter of the propodeal spiracle. Postpetiolar node weakly rounded and lower than petiolar node. In dorsal view, postpetiolar node wider than petiolar node (PTW 0.15, PPW 0.20) and petiolar node wider than long (PNL 0.09, PTW 0.15), anterior margin of petiole and postpetiole nearly straight, sides of petiole and postpetiole rounded.

Dorsal surface of mandibles, clypeus and median area of head smooth and shiny, with scattered piligerous punctae on head and mandibles. Head with longitudinal and parallel rugae, these joining near posterior margin of head, transverse rugae along posterior margin of head. Posterolateral portion of cephalic dorsum smooth and shiny. Mesosoma smooth and shiny, except for katepisternum (areolate), metapleuron (rugose and with longitudinal rugae), and anterior side of pronottum finely punctate. Petiole and ventral face of postpetiole (areolate). In dorsal view, mesosoma, petiole, postpetiole and gaster smooth and shiny.

Lateral and posterior margin of head with suberect to subdecumbent hairs. Scapes with decumbent hairs. Outer margin of mandibles with few and short appressed hairs. Mesosoma with short and long erect to subdecumbent hairs. Petiole and postpetiole with short decumbent hairs and long subdecumbent hairs. Tibia with appressed hairs. Gaster with abundant subdecumbent hairs. Color yellowish ferruginous.

#### Description of minor workers.

**Measurements** (n=1): HL 0.40; HW 0.34; SL 0.23; ML 0.10; EL 0.02; EM 0.11; HD 0.23; WL 0.41; PSL 0.03; PW 0.22; MFL 0.23; MFW 0.06; MTL 0.18, PTL 0.13; PNL 0.07; PTH 0.12; PTW 0.09; PPL 0.09; PPNL 0.08; PPH 0.08; PPW 0.13; GL 0.33; GW 0.28; CI 85; MI 25; SI 58; MLI 66; PPLI 69; PPI 136; PSI 10.

Head slightly longer than wide (CI 85), in full-face view nearly subquadrate, about 1.1 times longer than wide. Posterior margin of head slightly concave, posterolateral corners convex, lateral margins weakly convex. Mandibles with five teeth. Anterior margin of clypeus slightly straight, and laterally convex. Antennae with ten segments. Scape fails to reach posterior margin of head (HL 0.40, SL 0.23, SI 58). Eyes present, consisting of one ommatidium (EL 0.02). Supraclypeal area triangular but not well defined.

In profile view, promesonotum weakly convex, metanotal groove not well impressed. Propodeum about 1.2 times higher than long, dorsal face of propodeum slightly convex, declining posteriorly, posterodorsal corners unarmed or angulate, declivity nearly straight. Propodeal lobes short and convex. Propodeal spiracle rounded and situated above mid-height of sclerite, and beyond mid-length of sclerite by about half the diameter of the spiracle, distance from propodeal spiracle to posterodorsal corner of propodeum about the same as the diameter of the spiracle (PSL 0.03), and distance to declivity almost less than the diameter of the spiracle. In dorsal view, promesonotum about as long as wide, anterior margin rounded, sides nearly rounded, narrowed posteriorly; sides of propodeum straight.

Petiole weakly longer than high (PTL 0.13, PTH 0.12) and with a short peduncle, ventral face convex at center. Combined outine of dorsal surface of peduncle and anterior face of node straight, posterior face of node vertical and slightly concave, anterodorsal and posterodorsal corner rounded, dorsum convex. Subpetiolar process produced as a small denticle, same as the diameter of the propodeal spiracle. Postpetiolar node nearly rounded and lower than petiolar node. In dorsal view, postpetiolar node wider than petiolar node (PTW 0.09, PPW 0.13), and petiolar node wider than long (PNL 0.07, PTW 0.09), anterior and posterior margins of petiole and postpetiole straight, sides rounded in petiole and postpetiole.

Head, mandible and clypeus smooth and shiny with scattered piligerous punctae on head and mandibles. Gena and frontal lobes with short longitudinal rugae. Mesosoma smooth and shiny, except for katepisternum (areolate), and metapleuron (finely areolate combined with longitudinal rugae). Petiole and ventral face of postpetiole areolate. In dorsal view, mesosoma, petiole, postpetiole and gaster smooth and shiny.

Lateral margins, posterior margin of head and outer margin of mandibles with short decumbent hairs. Scapes with abundant subdecumbent hairs. Mesosoma with short and long suberect to subdecumbent hairs. Petiole and postpetiole with short and long decumbent hairs. Tibia with appressed hairs. Gaster with abundant suberect to subdecumbent hairs. Color yellowish ferruginous.

#### Distribution and biology.

*Carebara tanana* is known only from the center of Madagascar (Figure 69). This species was found in the savannah grassland, at an elevation of 1984 m, in leaf mold and rotten wood. This species was collected using maxi-Winkler traps.

#### Comments.

*Carebara tanana* can be confused with *C. tana* due to the form of the head. However, the two can be separated easily by the following combination of characters: in profile view, posterodorsal corner of propodeum of *C. tana* armed and unarmed in *C. tanana*; gaster with short and long and abundant suberect to subdecumbent hairs in *C. tanana*, while in *C. tana* the major worker has the gaster with short decumbent hairs and long subdecumbent or suberect hairs, and the minor worker with abundant subdecumbent and decumbent hairs, and sparse suberect hairs. Additional to this, *C. tanana* is known only from the center of Madagascar, while *C. tana* is known from the north of Madagascar. Only other two species were collected at the same area: *C. grandidieri*, and *C. nosindambo*.

*C. tanana* does not have intermediates in the major worker subcaste.

### Carebara vazimba

Taxon classificationAnimaliaHymenopteraFormicidae

Azorsa & Fisher sp. n.

http://zoobank.org/C6B92C61-0DA2-4873-AFD7-F2D00AB5B784

#### Holotype.

(major worker), MADAGASCAR, Antsiranana, Ambondrobe, 41.1 km 175° Vohemar, -13.71533, 50.10167, 10 m, littoral rainforest, 29.xi.2004, (*B.L. Fisher*). Collection code BLF11100, (CASC: CASENT0055532). **Paratypes**: (13 major workers and 17 workers), with same data as holotype, 10 major workers (BMNH: CASENT0055521, CASC: CASENT0055650, CASENT0055647, CASENT0055641, CASENT0055652, CASENT0055525, CASENT0055527, MCZ: CASENT0055536, MHNG: CASENT0055531, NHMB: CASENT0055643), and 15 workers (BMNH: CASENT0055524, CASC: CASENT0055636, CASENT0055535, CASENT0055640, CASENT0055518, CASENT0055526, CASENT0055529, CASENT0055528, CASENT0055644, CASENT0055538, CASENT0055530, CASENT0055651, MCZ: CASENT0055519, MHNG: CASENT0055523, NHMB: CASENT0055638). 2 major workers and 2 workers with same data as holotype but collected by 30.xi.2004: BLF11107, 1 major worker and 1 worker (CASC: CASENT0110667), BLF11123, 1 major worker and 1 worker (CASC: CASENT0052827).

#### Diagnosis.

Antennae ten-segmented. **Major**: Head nearly subrectangular, longer than wide, lateral margins straight and parallel, posterolateral corners well developed, posterior margin of head deeply concave in the middle, head with longitudinal rugae directed to the posterior margin; dorsum of propodeum flat, and declining posteriorly, posterodorsal corner with a pair of small triangular teeth; gaster with short decumbent hairs, and dispersed and longer subdecumbent hairs. **Minor**: Head nearly subrectangular, slightly longer than wide, posterior margin of head nearly straight, weakly concave, lateral margins convex; dorsum of propodeum nearly flat, posterodorsal corner with a pair of small triangular teeth, combined outline of dorsal surface of peduncle and anterior face of node concave, and posterior face slightly convex; gaster with short decumbent hairs, and longer and dispersed subdecumbent hairs.

**Figure 66.**
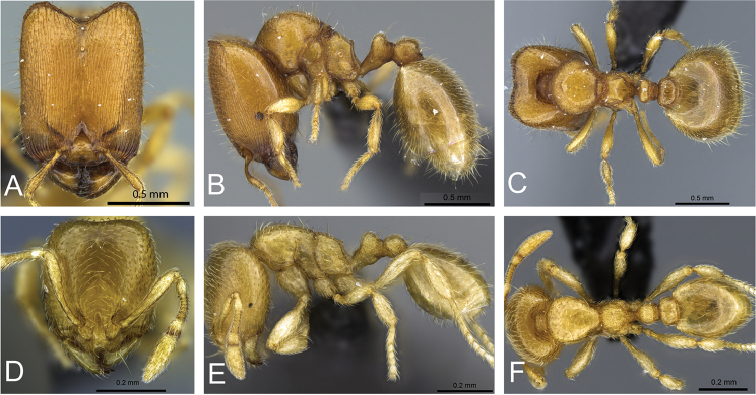
*Carebara vazimba*–holotype. Major worker, CASENT0055532: **A** head in full-face view **B** body in profile view **C** body in dorsal view. Minor worker, CASENT0055533: **D** head in full-face view **E** body in profile view **F** body in dorsal view.

**Figure 67.**
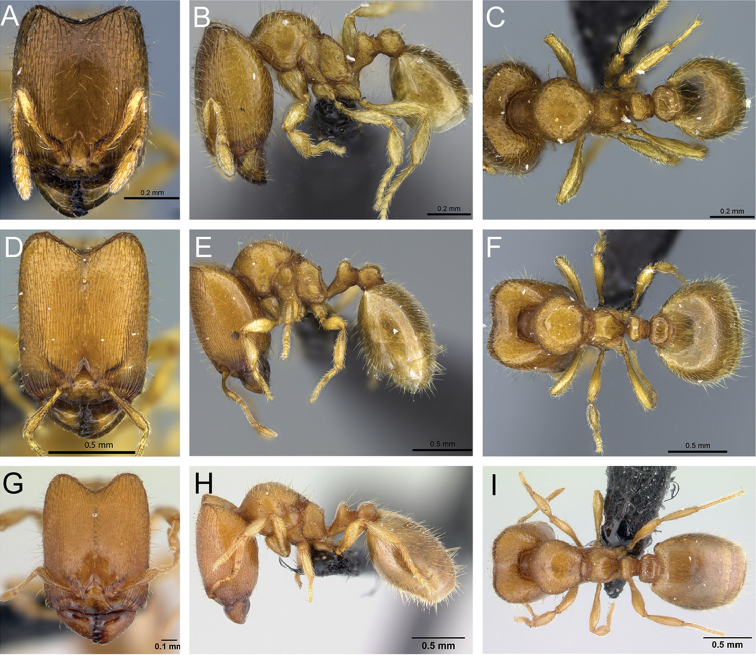
Intermediates of *Carebara vazimba*. Major workers, CASENT0055650: **A** head in full-face view **B** body in profile view **C** body in dorsal view. CASENT0055532: **D** head in full-face view **E** body in profile view **F** body in dorsal view. CASENT0110667 **G** head in full-face view **H** body in profile view **I** body in dorsal view.

#### Description of major workers.

**Measurements** (n=6): HL 0.64–1.02 (1.01); HW 0.50–0.78 (0.75); SL 0.26–0.35 (0.34); ML 0.14–0.24 (0.24); EL 0.01–0.05 (0.05); EM 0.16–0.26 (0.25); HD 0.34–0.57 (0.56); WL 0.52–0.86 (0.81); PSL 0.05–0.10 (0.10); PW 0.31–0.54 (0.52); MFL 0.30–0.47 (0.46); MFW 0.07–0.11 (0.10); MTL 0.23–0.38 (0.36); PTL 0.18–0.36 (0.36); PNL 0.09–0.14 (0.14); PTH 0.15–0.25 (0.24); PTW 0.14–0.23 (0.23); PPL 0.12–0.20 (0.19); PPNL 0.10–0.18 (0.18); PPH 0.12–0.24 (0.22); PPW 0.17–0.28 (0.27); GL 0.41–0.97 (0.92); GW 0.40–0.86 (0.86); CI 74–78 (74); MI 22–25 (24); SI 34–42 (34); MLI 58–64 (61); PPLI 53–67 (53); PPI 117–129 (117); PSI 9–13 (13).

Head longer than wide (CI 74–78), in full-face view nearly subrectangular, about 1.3 times longer than wide, slightly narrowed posteriorly. Posterior margin of head deeply concave in the middle, posterolateral corners well developed and rounded, lateral margins straight and parallel. Mandibles with six teeth. Anterior margin of clypeus nearly straight, and laterally convex. Antennae with ten segments. Scapes short (HL 0.64–1.02, SL 0.26–0.35, SI 34–42). Ocelli present or absent. Eyes present, consisting of one to twenty ommatidia (EL 0.01–0.05). Supraclypeal area triangular and well defined.

In profile view, posterolateral corner of head with an angulate and triangular tooth resembling a horn. Promesonotum high and nearly rounded. Promesonotal suture present or absent on dorsum, mesonotum absent or present, metanotal groove deeply impressed. Propodeum lower than promesonotum, about 1.5 times higher than long, dorsal face of propodeum flat and declining posteriorly, anterodorsal corner rounded, posterodorsal corners each armed with a triangular tooth, declivity concave with thin lateral laminae. Propodeal lobes convex. Propodeal spiracle rounded and situated slightly above mid-height of sclerite, and beyond mid-length of sclerite by about half the diameter of the spiracle, distance from propodeal spiracle to posterodorsal corner of propodeum about twice the diameter of the spiracle (PSL 0.05–0.10), and distance to declivity about 1.3 times the diameter of the spiracle. In dorsal view, promesonotum about as long as wide, anterior margin and sides rounded, narrowed posteriorly; sides of propodeum straight.

Petiole longer than high (PTL 0.18–0.36, PTH 0.15–0.25) and with relatively long peduncle, ventral face slightly convex in the middle. Combined outline of dorsal surface of peduncle and anterior face of node concave at center, posterior face of node vertical and straight, anterodorsal corner convex, posterodorsal corner rounded, and dorsum nearly rounded. Subpetiolar process produced as a small denticle, almost same as the diameter of the propodeal spiracle. Postpetiolar node convex and lower than petiolar node. In dorsal view, petiolar node almost as wide as postpetiolar node (PTW 0.14–0.23, PPW 0.17–0.28) and petiolar node wider than long (PNL 0.09–0.14, PTW 0.14–0.23), anterior and posterior margins of petiole and postpetiole almost straight, sides of petiole and postpetiole rounded.

Dorsal surface of mandibles, clypeus and frons smooth and shiny with scattered piligerous punctae on head and mandibles. Head with longitudinal and parallel rugae directed to posterior margin of head. Mesosoma smooth and shiny, except for katepisternum and propodeum (areolate), metapleuron (finely areolate and with longitudinal rugae), and sides of pronotum (finely longitudinal rugulose). Petiole, ventral face and sides of postpetiole areolate. In dorsal view, median area of promesonotum smooth and shiny, as well as postpetiole and gaster. Propodeum and petiole weakly sculptured in smaller major workers and smooth and shiny in larger major workers.

Lateral margins of the head with short appressed hairs and long erect to suberect hairs, posterior margin with subdecumbent hairs. Scapes with appressed hairs. Outer margin of mandibles with sparse and short appressed hairs. Mesosoma with short and long erect and suberect hairs. Petiole and postpetiole with short decumbent hairs and long subdecumbent hairs. Tibia with appressed hairs. Gaster with short decumbent hairs and long suberect to subdecumbent hairs. Color yellowish ferruginous.

#### Description of minor workers.

**Measurements** (n=10): HL 0.33–0.39; HW 0.29–0.34; SL 0.20–0.24; ML 0.08–0.09; EL 0.01–0.02; EM 0.09–0.11; HD 0.19–0.23; WL 0.32–0.39; PSL 0.02–0.04; PW 0.18–0.21; MFL 0.20–0.25; MFW 0.05–0.06; MTL 0.14–0.18; PTL 0.12–0.14; PNL 0.06–0.07; PTH 0.09–0.11; PTW 0.07–0.09; PPL 0.07–0.10; PPNL 0.06–0.08; PPH 0.06–0.08; PPW 0.10–0.12; GL 0.27–0.39; GW 0.21–0.26; CI 86–89; MI 22–27; SI 59–62; MLI 68–74; PPLI 54–71; PPI 122–147; PSI 6–13.

Head longer than wide (CI 86–89), in full-face view nearly subrectangular, about 1.2 times longer than wide. Posterior margin of head nearly straight, weakly concave, posterolateral corners rounded, lateral margins convex. Mandibles with five teeth. Anterior margin of clypeus nearly straight, and laterally convex. Antennae with ten segments. Scape fails to reach posterior margin of head (HL 0.33–0.39, SL 0.20–0.24, SI 59–62). Eyes present, consisting of one ommatidium (EL 0.01–0.02). Supraclypeal area short and triangular.

In profile view, promesonotum weakly convex, nearly flat, metanotal groove present. Propodeum about 1.3 times higher than long, dorsal face of propodeum nearly flat and declining posteriorly, anterodorsal corner rounded, posterodorsal corners each armed with a small triangular tooth, declivity concave with thin lateral laminae. Propodeal lobes convex. Propodeal spiracle small, rounded, and situated above mid-height of sclerite, and beyond mid-length of sclerite by about half the diameter of the spiracle, distance from propodeal spiracle to posterodorsal corner of propodeum about 2.2 times the diameter of the spiracle (PSL 0.02–0.04), and distance to declivity same as the diameter of the spiracle. In dorsal view, promesonotum about 1.1 times longer than wide, anterior margin rounded, sides convex and narrowed posteriorly; sides of propodeum straight.

Petiole longer than high (PTL 0.12–0.14, PTH 0.09–0.11) and with relatively short peduncle, ventral face convex at center. Combined outline of dorsal surface of peduncle and anterior face of node concave, posterior face of node vertical and slightly convex, anterodorsal and posterodorsal corner convex, dorsum rounded. Subpetiolar process produced as a small denticle, almost as large as the diameter of the propodeal spiracle. Postpetiolar node convex and lower than petiolar node. In dorsal view, postpetiolar node wider than petiolar node (PTW 0.07–09, PPW 0.10–0.12), and petiolar node wider than long (PNL 0.06–0.07, PTW 0.07–0.09), anterior and posterior margins of petiole and postpetiole nearly straight, sides nearly rounded in petiole and postpetiole.

Dorsal surface of head, mandibles and clypeus smooth and shiny with scattered piligerous punctae on head and mandibles. Gena and frontal lobes with longitudinal rugae. Mesosoma smooth and shiny, except for katepisternum and propodeum (areolate), and metapleuron (longitudinally areolate-rugose). Petiole and ventral face of postpetiole areolate-rugose. In dorsal view, promesonotum, postpetiole and gaster smooth and shiny; propodeum and petiole areolate.

Lateral margins and posterior margin of head with short decumbent hairs. Scapes with appressed hairs. Outer margin of mandible with sparse appressed hairs. Mesosoma with short and long suberect hairs. Petiole and postpetiole with short appressed hairs and long subdecumbent hairs. Tibia with appressed hairs. Gaster with short decumbent to appressed hairs and long subdecumbent hairs. Color yellowish ferruginous.

#### Distribution and biology.

*Carebara vazimba* is known from the north of Madagascar (Figure 69). This species was found in littoral rainforest and rainforest at 10 m and 240 m, respectively. Individuals of *C. vazimba* were found in rotten logs, leaf mold, and rotten wood. Specimens were collected using maxi-Winkler traps.

**Figure 68.**
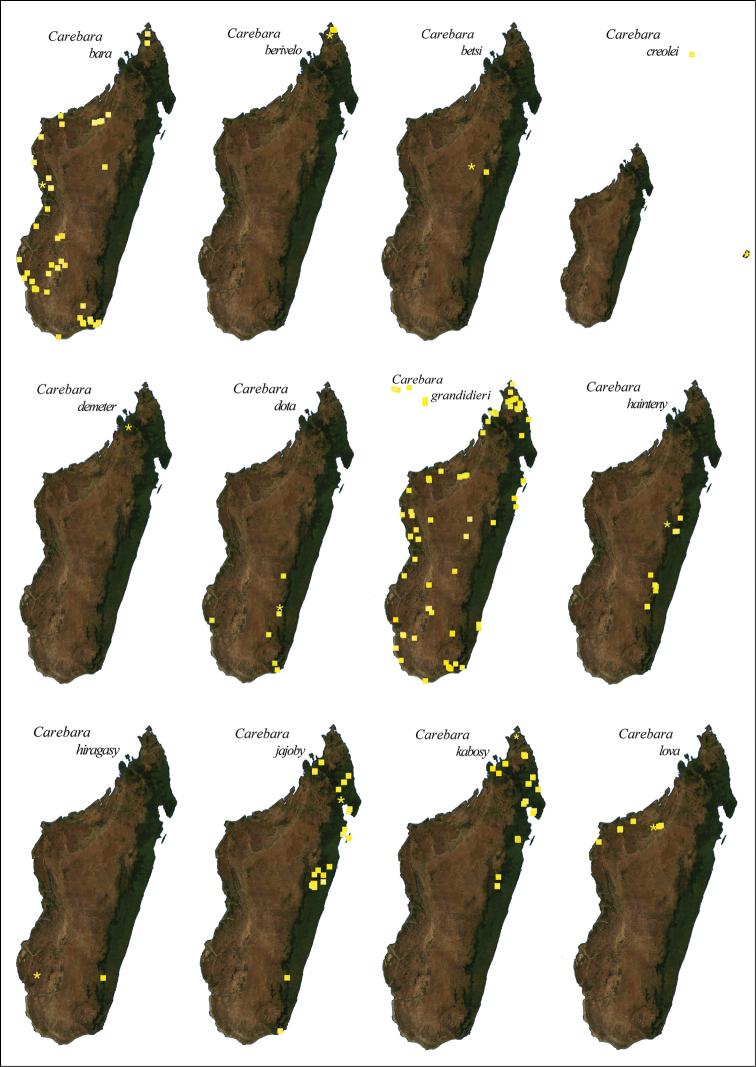
Distribution maps of Malagasy *Carebara* species: *C. bara. C. berivelo*, *C. betsi*, *C. creolei*, *C. demeter*, *C. dota*, *C. grandidieri*, *C. hainteny*, *C. hiragasy*, *C. jajoby*, *C. kabosy C lova*; type localities are indicated by stars.

**Figure 69.**
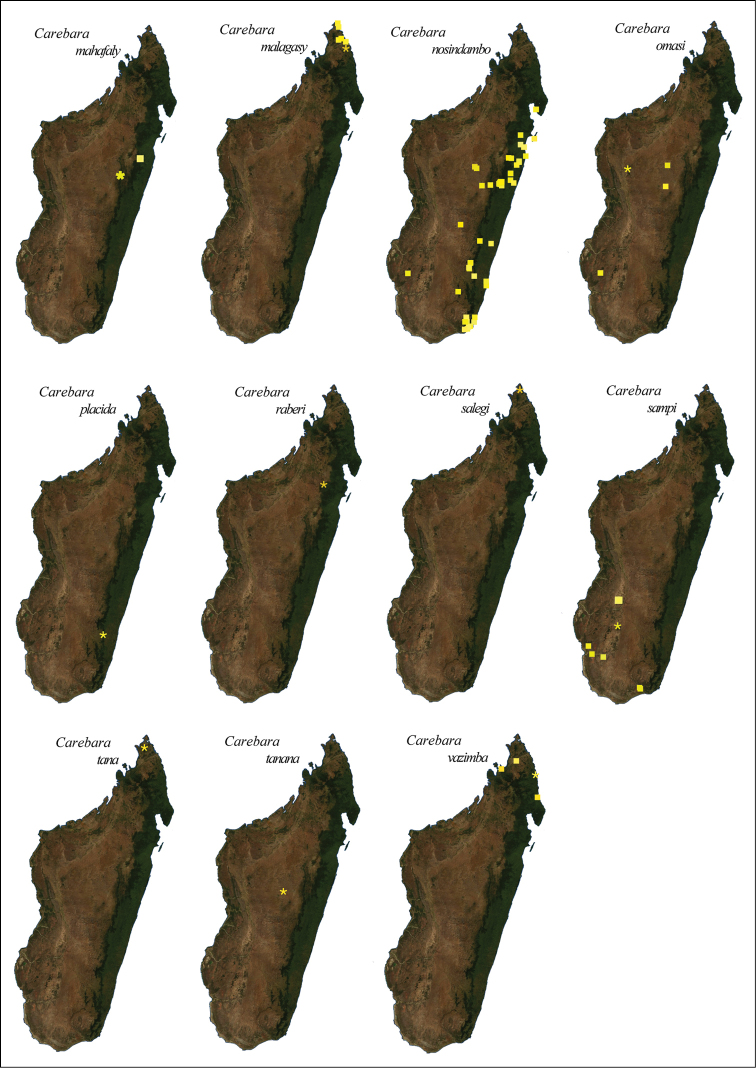
Distribution maps of Malagasy *Carebara* species: *C. mahafaly*, *C. malagasy*, *C. nosindambo*, *C. omasi*, *C. placida*, *C. raberi*, *C. salegi*, *C. sampi*, *C. tana*, *C. tanana*, *C. vazimba*; type localities are indicated by stars.

#### Comments.

*Carebara vazimba* can be confused with *C. kabosy* but can be separated by the length of the propodeal tooth, which is shorter than propodeal spiracle diameter in *C. vazimba* and slightly longer in *C. kabosy. C. vazimba* is distributed in the north of Madagascar, while *C. kabosy* is distributed in the north, northeastern and nortwestern areas of Madagascar. Other five species were collected at the same localities: *C. bara*, *C. berivelo*, *C. grandidieri*, *C. jajoby*, and *C. kabosy*.

*C. vazimba* have three intermediates in the major worker subcaste (Figure 67). The posterolateral corner of head in intermediate 1 is slightly narrowed posteriorly, the posterior margin of the head is medially concave in all intermediates. Eyes are small and reduced to one ommatidium in intermediate 1 but intermediate 3 has eyes made up of one to twelve ommatidia. Ocelli are absent in intermediates 1 and 2, but present in intermediate 3 (one ocellus). Reduced flight sclerites are present in intermediate 3, and absent in intermediates 1 and 2. The promesonotum is nearly rounded in intermediates 1 and 2, and strongly convex in intermediate 3. The dorsum of the mesosoma is convex anteriorly and gradually slopes to the declivity in intermediate 3, while in intermediates 1 and 2 the dorsum of the mesosoma is convex anteriorly, propodeum below the promesonotum. There is no variation in the form of the petiole. The sculpture of the head of intermediate 3 is more striking than that of intermediates 1 and 2, where rugae on the head are more finely marked and the frontal area is smooth and shiny. Pilosity on the head and body follows the same pattern in all intermediates.

#### Additional material examined.

**MADAGASCAR**: ***Antsiranana***: Antsiranana, Ambondrobe, 41.1 km 175° Vohemar, -13.71533, 50.10167, 10 m, littoral rainforest, 29.xi.2004, (*B.L. Fisher*); Antsiranana, Forêt Ambanitaza, 26.1 km 347° Antalaha, -14.67933, 50.18367, 240 m, rainforest, 26.xi.2004, (*B.L. Fisher*); *Antsiranana*: Forêt d’ Andavakoera, 21.4km 75° ENE Ambilobe; 4.6 km 356° N Betsiaka, -13.11833, 49.23, 425 m, rainforest, 15.xii.2003, (*B.L. Fisher*); Antsiranana, Forêt Ambato, 26.6 km 33° Ambanja, -13.4645, 48.55167, 150 m, rainforest, 8.xii.2004, (*B.L. Fisher*).

## Supplementary Material

XML Treatment for Carebara bara

XML Treatment for Carebara berivelo

XML Treatment for Carebara betsi

XML Treatment for Carebara creolei

XML Treatment for Carebara demeter

XML Treatment for Carebara dota

XML Treatment for Carebara grandidieri

XML Treatment for Carebara hainteny

XML Treatment for Carebara hiragasy

XML Treatment for Carebara jajoby

XML Treatment for Carebara kabosy

XML Treatment for Carebara lova

XML Treatment for Carebara mahafaly

XML Treatment for Carebara malagasy

XML Treatment for Carebara nosindambo

XML Treatment for Carebara omasi

XML Treatment for Carebara placida

XML Treatment for Carebara raberi

XML Treatment for Carebara salegi

XML Treatment for Carebara sampi

XML Treatment for Carebara tana

XML Treatment for Carebara tanana

XML Treatment for Carebara vazimba
